# A novel immune-related prognostic signature in epithelial ovarian carcinoma

**DOI:** 10.18632/aging.202792

**Published:** 2021-04-04

**Authors:** Tong Su, Panpan Zhang, Fujun Zhao, Shu Zhang

**Affiliations:** 1 Department of Gynecology and Obstetrics, Shanghai Key Laboratory of Gynecology Oncology, Renji Hospital, Shanghai Jiao Tong University School of Medicine, Shanghai 200127, China; 2 Department of Urology, Shanghai General Hospital, Shanghai Jiao Tong University School of Medicine, Shanghai 200080, China

**Keywords:** epithelial ovarian carcinoma, immune, prognostic signature, microenvironment

## Abstract

The immune response is associated with the progression and prognosis of epithelial ovarian cancer (EOC). However, the roles of infiltrated immune cells and immune-related genes (IRGs) in EOC have not been reported comprehensively. In the current study, the differentially expressed genes (DEGs) were filtered based on the integrated gene expression data acquired from The University of California at Santa Cruz (UCSC) Genome Browser. Then, IRGs and transcriptional factors (TFs) were screened based on the ImmPort database and Cistrome database. A total of 501 differentially expressed IRGs, and 76 TFs were detected. A TF-mediated network was constructed by univariate Cox analysis to reveal the potential regulatory mechanisms of IRGs. Next, a nine immune-based prognostic risk model using nine IRGs (PI3, CXCL10, CXCL11, LCN6, CCL17, CCL25, MIF, CX3CR1, and CSPG5) was established. Based on the risk score worked out from the signature, the EOC patients could be classified into low-risk and high-risk groups. Furthermore, the immune landscapes, elevated by the cell-type identification by estimating relative subsets of RNA transcripts (CIBERSORT) algorithm and the Tumor Immune Estimation Resource (TIMER) database, effectuated different patterns in two groups. Thus, an immune-based prognostic risk model of EOC elucidates the immune status in the tumor microenvironment, and hence, could be used for prognosis.

## INTRODUCTION

Epithelial ovarian cancer (EOC) has the highest mortality rate in gynecological malignant neoplasms in women [1]. The majority of the EOC patients have a poor clinical diagnosis and low 5-year overall survival rate due to unclear early symptoms and lack of effective early diagnostic measures [2]. The traditional therapeutic options exhibited subpar efficacy with high recurrence rates and chemoresistance [3]. Thus, it is essential to develop novel efficient therapeutics for EOC. Recently, with an improved understanding of the role of the immune system and the molecular mechanism underlying immune regulation, cancer immunotherapy has demonstrated major benefit in several types of solid tumors, such as melanoma, non-small-cell lung cancer, and renal-cell cancer. Also, several checkpoint inhibitors have been approved for the therapy of cancers. However, as an emerging treatment, the response rate of immunotherapy in EOC has been disappointing, and no immunotherapies have yet been approved [4].

Tumor microenvironment (TME) is a complex environment composed of tumor cells as well as various stromal cells, such as tumor-infiltrating immune cells (TIICs), endothelial cells, fibroblasts, and extracellular molecules [5]. These stromal cells are involved in the proliferation, invasion, migration, and drug resistance to improve tumorigenesis. The EOC is an immunogenic tumor. TIICs in the TME have essential effects on clinical outcome [6]. A large number of studies reported the interactions between TIICs and prognostic assessment in ovarian cancer [7, 8]. The roles of the TME have gained increasing attention in the field of cancer immunotherapy, especially the type and content of TIICs in TME [9]. The immunosuppressive TME is a major barrier for EOC immunotherapy, as evident by its intrinsic properties [4]. Thus, it is beneficial to assess the infiltration status of immune cells and explore the biomarkers that would evaluate the distribution of TIICs in the EOC. This would aid in understanding the immune microenvironment and provide novel insights for both prognosis prediction as well as therapeutic targets. Since there is rarely a prognostic model based on IRGs to systematically evaluate the tumor immune cell function and predict the overall survival of EOC patients, we conducted this bioinformatics analysis.

In the current study, based on a large-scale bioinformatic analysis of The Cancer Genome Atlas (TCGA) and the Genotype Tissue Expression (GTEx) database, we applied the CIBERSORT method to estimate the fraction of 22 immune cell types and investigate the correlation with the overall survival of EOC patients. Then, differentially expressed IRGs and transcription factors (TFs) were filtered, and a TF-regulated IRGs’ network was investigated. Furthermore, we filtered the prognostic IRGs and performed multivariate Cox regression analysis to construct a predictive risk prognostic model. According to the calculated immune-related risk score, the EOC patients could be classified into low-risk and high-risk groups, which exhibited a significant difference in immune infiltrations in the microenvironment and obviously various prognoses.

Although different patterns of immune infiltration are identified as hallmarks of various clinical outcomes of cancer, the specific IRGs in the microenvironment and their potential impact are less well-studied. Herein, we used bioinformatics to explore the differentially expressed IRGs and developed a potential TF-regulatory network. We identified a nine-gene signature; its expression could predict the prognosis of EOC. Thus, the present study provided a novel insight into the immune microenvironment of EOC and impetus to further study the genes with respect to cancer biology. These data provided a potential tool for clinical prognostication.

## RESULTS

### Differential composition of tumor-infiltrated immune cells in EOC tissues

First, we constructed a CIBERSORT algorithm to estimate the proportion of 22 infiltrated immune cells in EOC samples. Samples with P>0.05 were rejected, leaving 208 OC samples eligible for further analysis. The expression data showed varied cell content of each sample showed that macrophages were mainly infiltrating immune cells in the EOC tissue (Supplementary Figure 1A). Also, the correlation among infiltrating immune cells was analyzed (Supplementary Figure 1B), indicating that the content of activated NK cells was strongly correlated with the content of CD8^+^ T cells, and the content of M1 macrophages was positively related to the content of follicular helper T (Tfh) cells. On the other hand, the infiltration of M2 macrophages was negatively correlated to the infiltration of a series of immune cells, including M1 macrophages, CD8+ T cells, T follicular helper cells (Tfh cells), regulatory T cells (Tregs), activated natural killer (NK) cells as well as plasma cells, which hinted for immunosuppressive effects of M2 macrophages. Next, we investigated the correlation between tumor-infiltrating immune cells and the clinical outcomes by univariate Cox regression in 193 patients with a follow-up time of>90 days. The data showed that M1 macrophages, plasma cells, as well as Tfh cells, acted as protecting cells and were associated with improved prognosis. However, abundant neutrophils indicated a poor prognosis. Thus, different immune infiltration could be a signature evaluating clinical outcomes (Supplementary Figure 1C–1F).

### Differential expression profile of IRGs in EOCs

**Figure 1:**
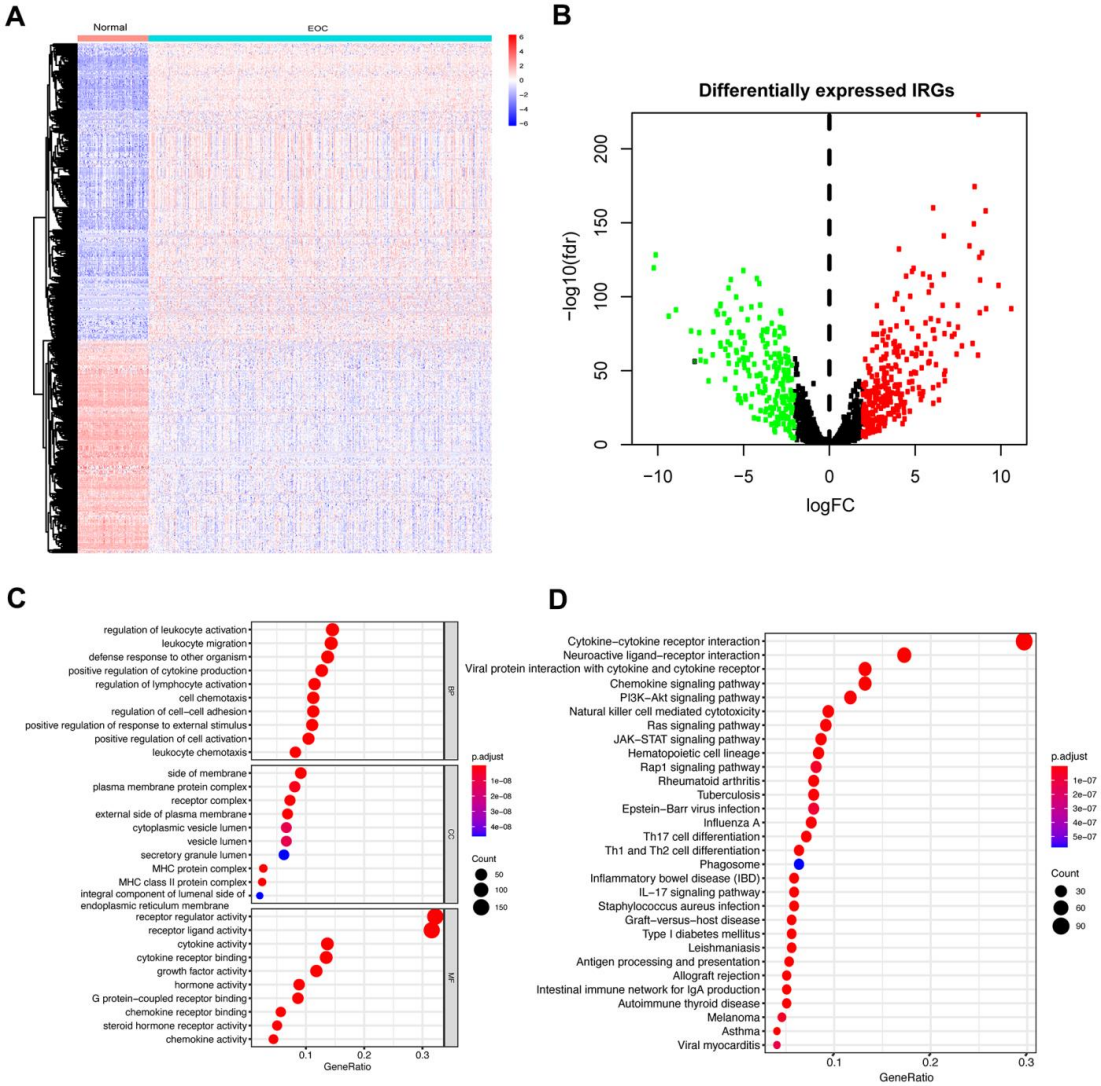
**Differentially expressed IRGs in EOC and functional enrichment analysis of differentially expressed IRGs.** (**A**) Heatmap of significantly differentially expressed IRGs in EOC. The color from blue to red represents the progression from low expression to high expression. (**B**) Volcano plot of differentially expressed IRGs. The red dots in the plot represent upregulated genes, and green dots represent downregulated genes. Black dots represent no differentially expressed genes. (**C**) GO analysis. From top to bottom, the figure represents BP, CC, and MF, respectively. (**D**) KEGG pathways analysis. The top 30 significant pathways are screened.

Furthermore, we investigated IRGs to reveal the immune landscape of the microenvironment. A total of 2498 IRGs were obtained from the ImmPort Database. Based on the expression profiles of ovarian tumors and normal tissues, a total of 501 IRGs (Figure 1A) were identified as DEGs in EOC samples compared to the normal samples (Supplementary Table 3). Among the differentially expressed IRGs, 292 were upregulated, and 209 were downregulated (Figure 1B).

To dissect the biological functions of these identified IRGs, we performed GO and KEGG analysis. Typically, the functional enrichment analysis revealed that the primary functional categories mainly enriched in these genes were related to immune and inflammatory responses. For BP, the primarily relevant processes were regulation of leukocyte activation and leukocyte migration in addition to positive regulation of cytokine production and regulation of lymphocyte activation. For CC, the side of membrane and plasma membrane protein complex were enriched terms. Receptor regulator activity, receptor-ligand activity, and cytokine activity were mainly MF categories (Figure 1C). The KEGG pathway analysis revealed that these genes were maximally involved in the cytokine-cytokine receptor interaction pathway (Figure 1D).

### Identification of prognostic IRGs in EOC

**Figure 2:**
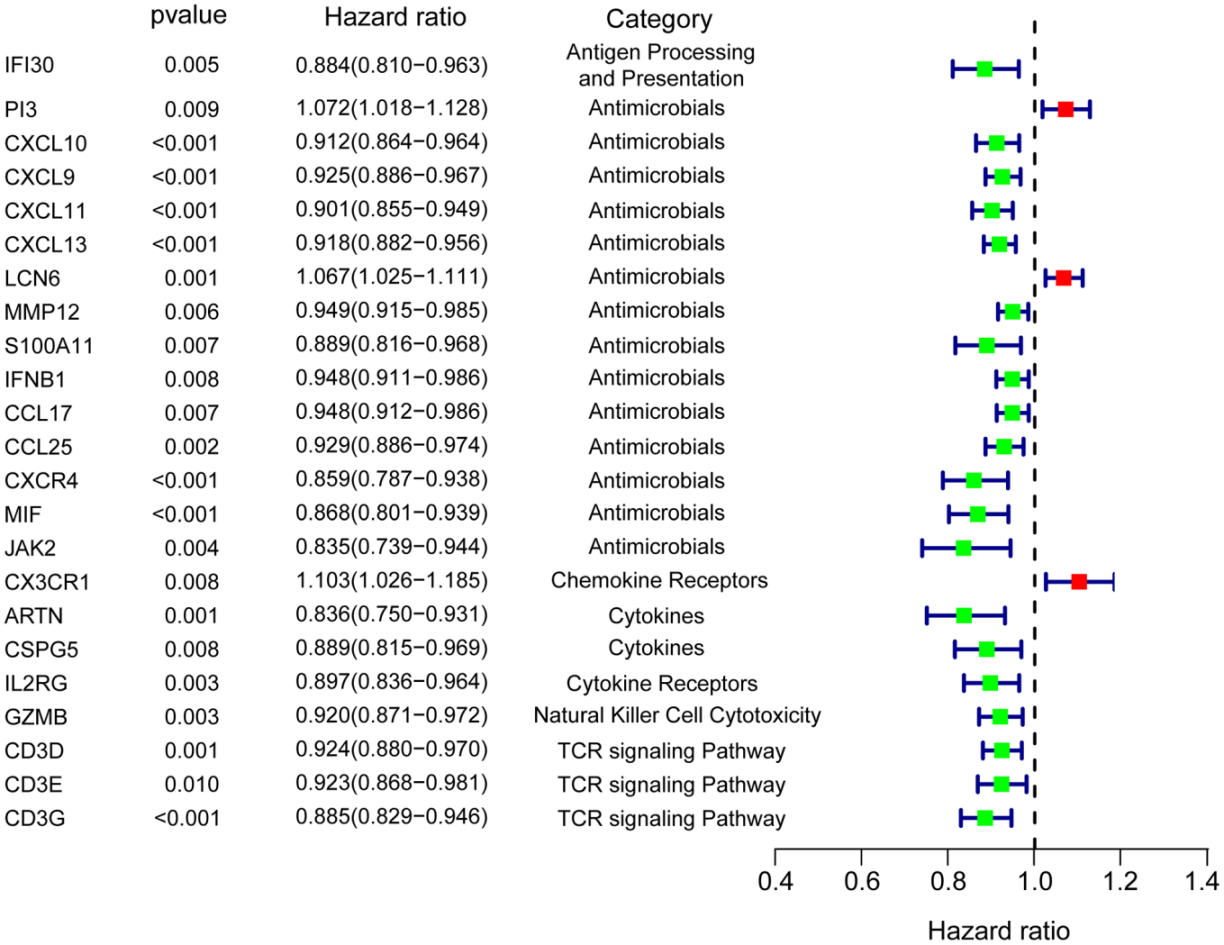
**Forest plot of hazard ratios showing the differentially expressed prognostic IRGs.**

**Figure 3:**
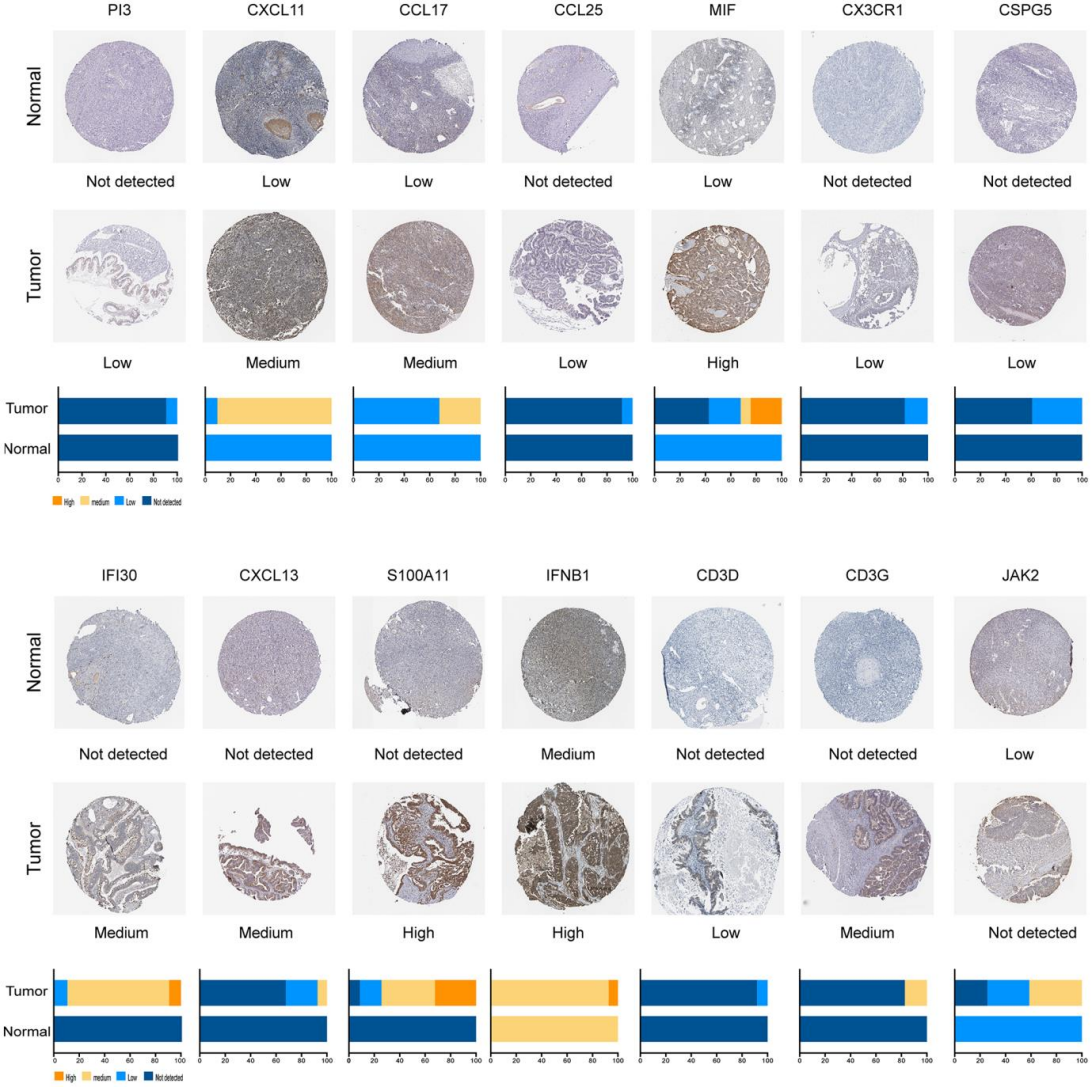
**Differential expression of prognostic IRGs in EOC in normal and EOC tissues.**

We performed univariate Cox analysis to evaluate the prognostic characteristics of the differentially expressed IRGs. The survival analysis was only performed on 396 patients who were followed up for >90 days. A total of 23 genes were significantly correlated with patient OS (P<0.01) and forest plot of the hazard ratios was illustrated in Figure 2, including genes involved in antigen processing and presentation (*IFI30*), antimicrobials (*PI3*, *CXCL9*, *CXCL10*, *CXCL11*, *CXCL13*, *LCN6*, *MMP12*, *S100A11*, *IFNB1*, *CCL17*, *CCL25*, *CXCR4*, *MIF*, and *JAK2*), chemokine receptor (*CX3CR1*), cytokines (*ARTN* and *CSPG5*), cytokine receptor (*IL2RG*), NK cell cytotoxicity (*GZMB*), and T cell antigen receptor (TCR) signaling pathway (*CD3D*, *CD3E*, and *CD3G*). The protein expression was also explored in the human atlas protein database (Figure 3).

### Identification transcriptional regulatory network for IRGs

**Figure 4:**
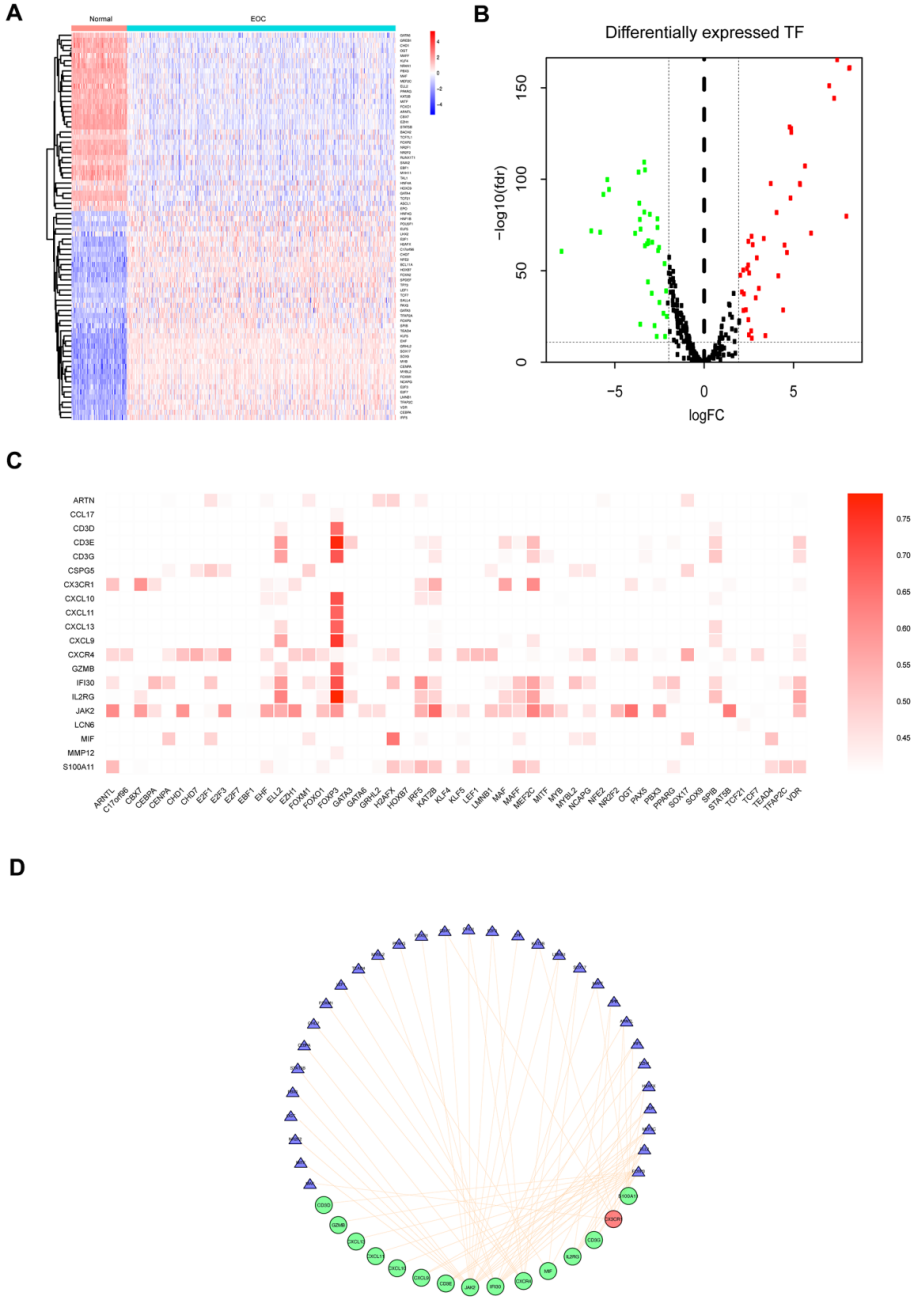
**Differentially expressed TFs in EOC, and transcriptional regulatory network constructed of TFs for prognostic IRGs.** (**A**) Heatmap of significantly differentially expressed TFs in EOC. (**B**) Volcano plot of differentially expressed TFs. (**C**) Correlations between differentially expressed TFs and 23 prognostic IRGs (P<0.05). (**D**) Regulatory network of TFs and the main prognostic IRGs (correlation coefficient >0.5 and P<0.05). The red circles represented upregulated expression of prognostic immune-related genes, the green circles represented downregulated expression of prognostic immune-related genes, the blue triangles represent prognosis-related TFs, and the orange lines represent positive regulatory correlations.

Subsequently, we explored the regulatory mechanism underlying the differentially expressed TFs and prognostic IRGs in tumor samples and established a TF-immune gene regulatory network. We obtained 318 TFs from the Cistrome Database, among which 76 TFs were identified as DEGs in EOC samples compared to the normal samples (Figure 4A, 4B, Supplementary Table 4). Correlation test function was utilized to test the correlations with cutoff criteria set as the correlation coefficient >0.5 and P<0.001 (Figure 4C), and a network was constructed (Figure 4D). The results revealed that forkhead box P3 (FOXP3) was regulated a large number of prognostic immunity genes. Also, MEF2C and ELL2 were TFs with a significant regulatory effect on the immunity genes, which was in agreement with the mRNA expression levels in the EOC tissue.

### Construction of an immune-related prognostic signature

**Figure 5:**
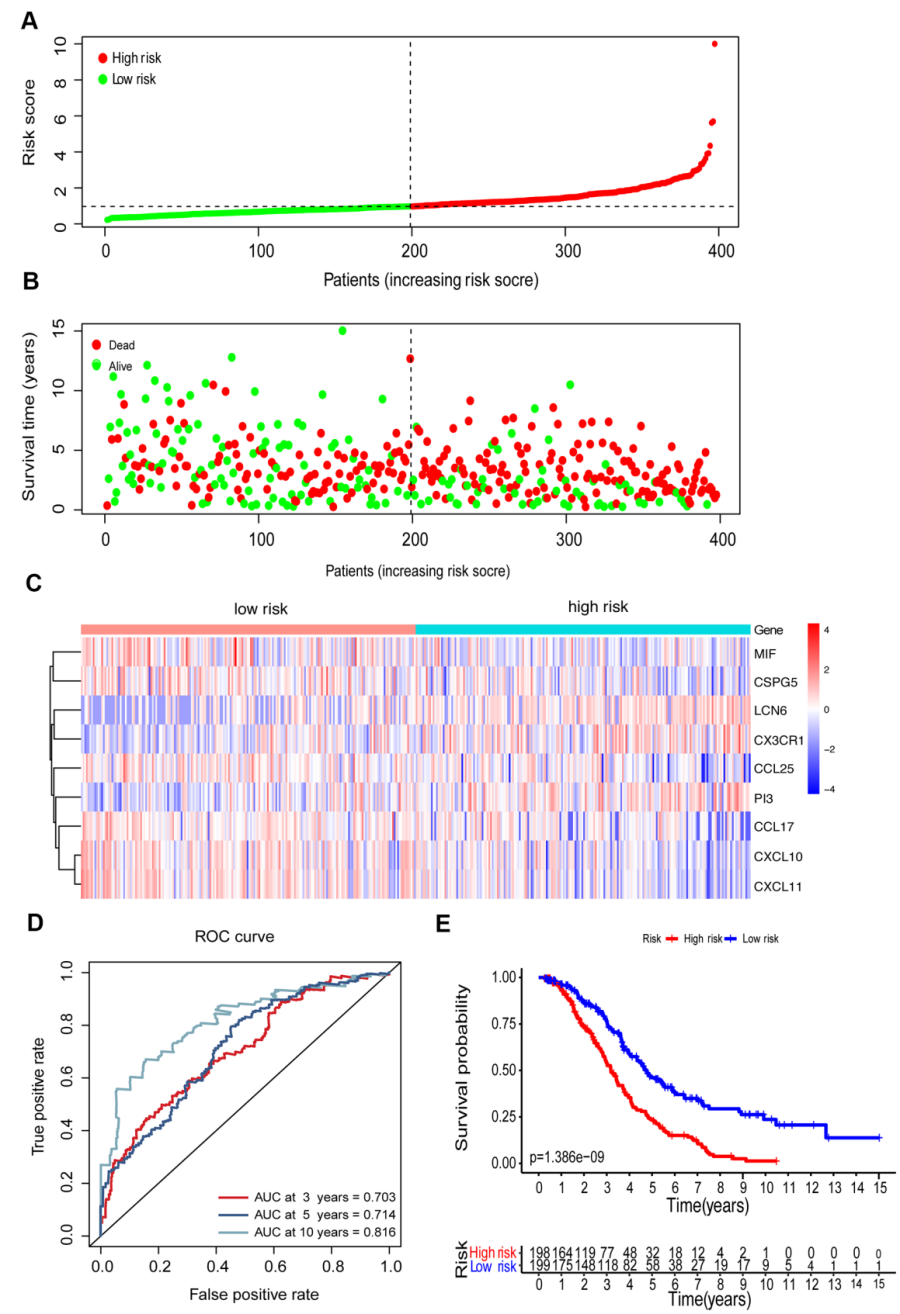
**Construction of the immune-related prognostic model for EOC.** (**A**) The risk score distribution of EOC patients in the prognostic model. (**B**) Survival status scatter plots for EOC patients in the prognostic model. (**C**) Heatmap of expression profiles of 9 prognostic genes in the high-risk and low-risk groups. (**D**) Time-dependent ROC curve analysis of the prognostic model (3, 5, and 10 years). (**E**) KM curve analysis of the high-risk and low-risk groups.

To establish an immune-related clinical prognostic signature, we further performed a multivariate Cox regression analysis of 23 candidate genes and selected 9 genes for modeling (Supplementary Table 3). A risk score was computed based on the mRNA expression level of the genes and their corresponding regression coefficients. The median risk score was set as a threshold to group the patients into high-risk groups (n=198) and low-risk groups (n=198). The distribution of risk score, the survival overview, and a heatmap exhibiting gene expression profiles in high- and low-risk OC groups were presented in Figure 5A–5C. The time-dependent receiver operating characteristic (ROC) analysis was performed, and the area under the curve (AUC) was 0.703 at 3 years and 0.714 at 5 years, indicating a good sensitivity and specificity of the immune-based prognostic model (Figure 5D). KM curves established a correlation between patients with high-risk scores and markedly poor overall survival, indicating a high prognostic performance of such prognostic index in clinical outcomes (P<0.001, Figure 5E).

**Figure 6:**
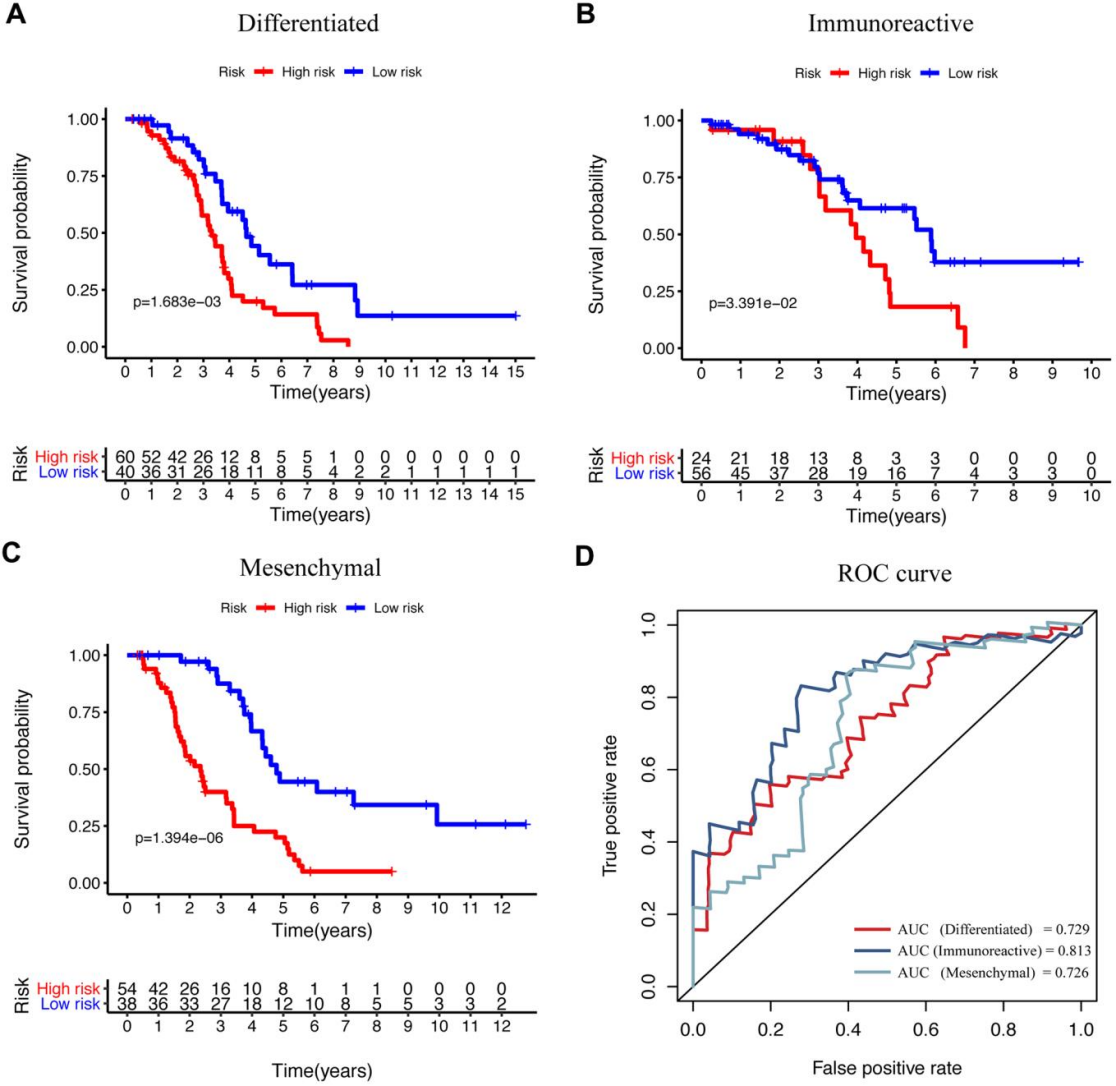
**The efficacy of signature in diverse subtypes according to the differentiated expressed genes.** (**A**–**C**) KM curve analysis of the high-risk and low-risk groups in “differentiated,” “immunoreactive,” and “mesenchymal” subtypes. (**D**) Time-dependent ROC curve analysis of the prognostic model in “differentiated,” “immunoreactive,” and “mesenchymal” subtypes.

EOC is a histologically and molecularly diverse disease that has been divided into different subtypes according to gene expression, which was correlated with specific characteristics and patient survival. A previous study described four molecular subtypes based on the gene expression in the clusters in the TCGA network study, which has also been validated in several other datasets [10, 11]. The subtypes were termed as “immunoreactive,” “differentiated,” “proliferative,” and “mesenchymal,” which provides new insights into the underlying biology and molecular pathogenesis and promoted treatment advancing. To examine the application of our signature, we performed KM survival analysis and ROC curve individually in four subtypes. The results showed that our model exhibited high prognostic performance in three subtypes except for the “proliferative” subtype (Figure 6A–6C). The ROC analysis also showed good sensitivity and specificity for “immunoreactive,” “differentiated,” and “mesenchymal” subtypes (Figure 6D). On the other hand, for the “proliferative” subtype, the P-value of survival analysis and the AUC of the ROC curve were not significant (Supplementary Figure 3). Furthermore, we also explored the efficacy of the immune-related prognosis model by classifying ovarian cancer according to three significant molecular aberrations in TP53, BRAC1, and BRAC2, respectively. The data revealed that the signature showed marked application in the group with TP53, BRAC1, and BRAC2 mutations (Supplementary Figure 4A–4C); also, the ROC curve showed a significant difference (Supplementary Figure 4D). These results indicated that our IRG-related signature could be used in combination with genotyping, which may show better prognostic performance.

### Differential distribution of tumor-infiltrating immune cells associated with immunity risk signature

**Figure 7:**
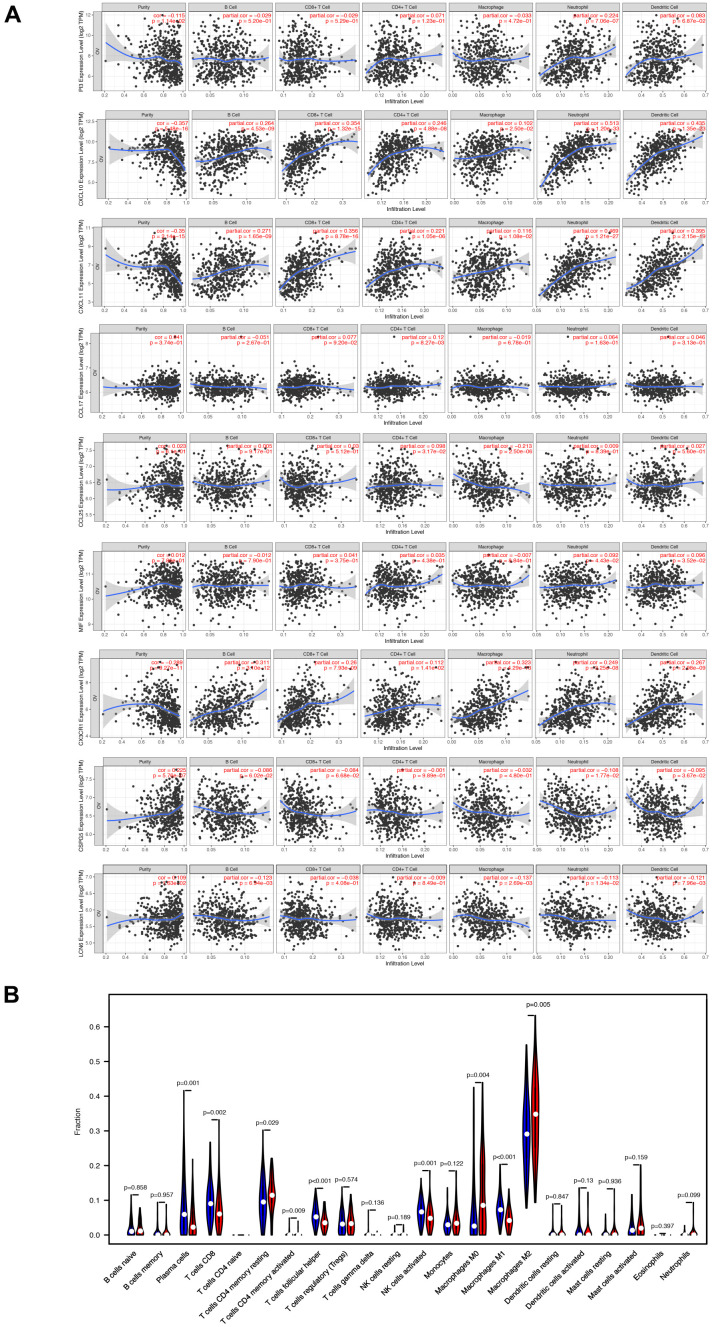
**The varied proportions of immune cells based on 9 immune-related prognostic signatures.** (**A**) The correlations of 9 signatures and 6 subtypes of immune cells. (**B**) The relative percentage of 22 subtypes of immune cells in high-risk and low-risk groups.

Subsequently, we examined the correlation between the differential abundance of immune cell infiltration and the immune-related prognostic signature. The TIMER database was utilized to explore the correlation of nine immune-related signatures with infiltration of six types of immune cells in EOC tissues (Figure 7A). Next, the CIBERSORT algorithm was applied to estimate the abundance of 22 immune cells in two groups separated by the overall nine-gene signature. Subsequently, 103 samples in the low-risk group and 76 samples in the high-risk group with CIBERSORT P-value <0.05 were extracted for further analysis. The heatmap of immune cell infiltration was exhibited in Supplementary Figure 2. The violin diagrams further visualized the differential abundance of infiltrated immune cells between high- and low-risk samples (Figure 7B). The results suggested heterogeneity between two groups of samples, with extremely higher fractions of resting memory CD4 T cells, M0 macrophages, M2 macrophages, and neutrophils in samples from patients with a high immune-related risk score, while plasma cells, CD8 T cells, activated memory CD4 T cells, Tfh cells, activated NK cells, and M1 macrophages existed abundantly in samples from the low-risk group.

## DISCUSSION

EOC is one of the most malignant cancers of the female reproductive system worldwide, with the highest morbidity and lethality rate. Therefore, precise prognostic strategies and effective new treatments are an urgent requirement for EOC patients. The immune cell infiltrations in TME play critical roles in the biological behavior of cancer, while immunotherapy has demonstrated major benefits in other types of cancers that have been disappointing in EOC [12]. The diversity of immune cells is one reason that leads to different prognoses and various effects towards immunotherapy. Thus, it is important to explore unique immune infiltration patterns and biomarkers of each EOC patient and develop tailored immunotherapy. Currently, the common strategy to identify the immune cell infiltration in the microenvironment is immunohistochemistry, with limitations, such as the limited markers of each type of cell. Recent studies have explicated the immune-related biomarkers of EOC and constructed models based on immune/stromal scores or immune-related gene pairs in order to elucidate the EOC immune microenvironment [13–15]. The current study focused on the construction of the prognosis model, and comprehensively explored the immune landscape of each EOC patient. Next, we compared the transcriptional expression profiles in EOC tissues and normal tissues to further explore the molecular mechanisms and derived a series of immune-related DEGs. The immune response and cancer-related pathway were the main functions of these genes. We also described the function of these differentially expressed IRGs and a TF regulatory network of these key IRGs. Next, we constructed a nine-gene signature, a simplified model that could be applied to estimate the immune status and predict prognosis. The current data showed that the immune infiltration in the low- and high-risk groups based on this nine-gene signature was significant, with protective immune cells in the microenvironment of patients in the low-risk group and harmful infiltrating immune cells in tissues in the high-risk group, and the clinical outcomes of patients with different risk score varied. Therefore, the nine-gene signature was an independent prognostic signature and could be utilized as guidance on prognosis and cancer therapy.

Ovarian cancer is a highly heterogeneous disease, and different cells of origin, including fallopian tube, peritoneum, or endometrial tissue, have been reported. Some benign diseases induce malignant epithelial ovarian tumors, such as ovarian endometriosis [16], due to altered local and systemic immunity. This indicated that the association between *in situ* immune microenvironments is a vital factor of ovarian tumor initiation and progression. A recent study reported that chronic inflammation in endometriosis promoted endometriosis-associated ovarian cancer, and a large proportion of endometriosis cases had a cancer-like immune gene signature [17]. As a typical feature of endometriosis, inflammation greatly modulated the progression of endometriosis and endometriosis-associated ovarian cancer. The immune system is also important for ovarian cancer clinical outcome. Therefore, the exploration of immune infiltration and immune-related signature could be used as a robust model.

Accumulating evidence established the prognostic value of IRGs in various types of cancer [18–20]. In the current study, a group of nine genes (*PI3*, *CXCL10*, *CXCL11*, *LCN6*, *CCL17*, *CCL25*, *MIF*, *CX3CR1*, and *CSPG5*) was used in the classifier which predicted the prognosis in EOC. Reportedly, these IRGs had correlations with patients’ OS. The peptidase inhibitor 3 (*PI3*)/elafin, a serine protease inhibitor with anti-microbial, anti-inflammatory, and immune-modulatory properties, was a key endogenous microbicide at female reproductive tract mucosal surfaces [21]. It could directly regulate the biological behavior of cancer cells as well as the immune microenvironment [22]. Recent studies identified PI3 as a biomarker of poor outcome of ovarian cancer and indicated recurrence [23]. Both CXCL10 and CXCL11 are ligands of CXCR3, with CXCL10 as a key driver chemokine [24]. This axis has been identified as an independent prognostic marker as well as a promising therapeutic target [25]. In addition, the axis regulates the proliferation and metastasis of cancer cells. By paracrine mechanisms, the activation of this axis could modulate the migration, differentiation, and activation of immune cells, including T cells, monocytes, dendritic cells, and NK cells [26]. Some studies also reported a strong crosslink between the PD-1/PDL-1 axis and other immune-related pathways [27, 28], and the function of promoting tumor progression [29–31]. CCL17 and CCL25 are both members of the CC chemokine family, in which CCL17 is released by macrophages, neutrophils, or dendritic cells. It is a high-affinity ligand of CCR4 and related to immunosuppression in the tumor microenvironment, especially by promoting the infiltration of regulatory T cells [32–35]. CCL25 is the specific ligand of CCR9, and both were highly expressed in various types of cancer and activate multiple signaling pathways, especially the pathways related to drug resistance as well as metastasis, thus related to poor prognosis [36–40]. The prevalence of certain cytokines linked endometriosis and ovarian cancer [16], and the CC-chemokine ligands and CXC-chemokine ligands are the two major classes of chemokines [41], indicating that these chemokines may be identified as biomarkers for prognosis. Furthermore, CX3CR1 is a major chemokine receptor in cytokines and chemokine networks preceding by macrophages in TME, followed by activating multiple downstream pathways: MAPK, PI3K, and JAK-STAT [40]. MIF is a key immunosuppressive cytokine produced by various cells and tissues that promote tumor progression mediated by macrophages in ovarian cancer and other cancer entities [42, 43]. The expression of PDL-1 was regulated, and MIF also facilitated the tumor cells’ escape immune surveillance [43]. Further, LCN6, a member of lipocalin protein family, participates in the regulation of cellular functions, such as cell homeostasis, substance transport, and immune response, and CSPG5 is a proteoglycan with an EGF-like module that may function as a growth and differentiation factor of neurons; these factors have not been previously reported to be related with ovarian cancer prognosis, and could serve as novel biomarkers [44–47]. Furthermore, the imbalance in gene expression plays a critical role in the immune microenvironment compared to that of the individual gene.

TFs are essential regulators of gene expression patterns, which in turn, regulate multiple BPs. Herein, we identified FoxP3 as the major TF involved in the regulation of IRGs and modulation of immune landscapes. FoxP3 is an essential regulator in immune system development and function. A recent study demonstrated FoxP3 as a marker of CD4^+^CD25^+^ Treg cells and illustrated its predominant role in the development and function of Tregs [48]. The negative regulation of cytokine genes facilitates FoxP3-mediated suppression of the immune response and induced immune tolerance [49]. In tumor immunity, FoxP3^+^ Treg cells are also related to immune escape. Several studies have reported the correlation between the high density of FoxP3^+^ Treg cell infiltration and poor prognosis [50–52]. Another study encompassing 104 EOC patients reported that FoxP3^+^ Treg cells suppressed tumor-specific T cells and induced the growth of tumors *in vivo*. Also, a high density of infiltrations of FoxP3^+^ Treg cells is a marker of reduced prognosis [53]. MEF2C and ELL2 were two other critical TFs that regulated the IRGs in the immune microenvironment, related to the development and function of plasma cells and other cells [54]. Rather than a single TF, these complicated regulatory networks regulate the complex community of the EOC immune microenvironment.

Emerging evidence suggested that the heterogeneity of immune cell composition, both innate and adaptive immune systems, plays a crucial role in the prognosis and treatment response of cancer patients [55, 56]. Differential immune infiltration responses lead to tumor rejection or progression. The recurrence after surgery and first-line chemotherapy is one of the major problems regarding ovarian cancers. In this scenario, reduced immunosurveillance is also one of the leading factors for ovarian cancer’s inherent chemoresistance [57–59]. The immune microenvironment influences the effect of chemotherapy. In turn, the chemotherapy drugs modulate the immune cell infiltrations by boosting tumor immunogenicity [60]. For instance, a series of germline or somatic mutations in BRCA1 or BRCA2 could exhibit various immune responses towards chemotherapy. Lo et al. reported that the platinum- and taxane-based chemotherapy showed a differential immune response in patients with the heterogeneous immune microenvironment, which could enhance antitumor immunity [61]. These studies also triggered an interest in the role of immune infiltration in the effect of chemotherapy. Therefore, in this study, we explored immune cell infiltration in the EOC microenvironment. Our results showed that M1 polarized macrophages, along with plasma cells and Tfh cells, were negatively related to the prognosis of EOC patients. On the other hand, a high density of neutrophils was a biomarker of poor prognosis, which indicated that the immune cells in TME were greatly related to clinical outcomes. Furthermore, 22 TIICs exhibited distinct infiltration patterns in the high-risk score group compared to the low-risk score group based on our nine-gene prognostic signature. The results identified macrophages as infiltrated immune cells with the highest proportion in cancer tissues, especially the M2 subtype. Macrophages are critical mediators in maintaining tissue homeostasis [62]. Substantial evidence indicated that the presence of tumor-associated macrophages is correlated with a poor prognosis [63]. A typical activation status described the classical (M1) and alternative (M2) polarization of macrophage, and M2 polarized macrophages are abundant in tumor tissues exhibiting poor prognosis. Furthermore, the control of other immune cells in the microenvironment, regulation of vascular structure or ECM, or direct regulation of tumor cell function revealed that M2 macrophages are significant regulators of the microenvironment that promote the malignant behavior of tumors [62]. These results demonstrated that M1 polarization of macrophages was lower and that of M2 macrophages was increased, with lower M0 macrophages—the inactivated subtype with neither inflammatory nor tumor-related function—in the high-risk group. Additionally, activated NK cells, the innate cytotoxic lymphocytes which surveil and eliminate cancer, effectuate a remarkably low infiltration in the high-risk group [64]. Previous evidence indicated that NK cells produce and release a variety of cytokines and chemokines to regulate immune responses [65, 66]. For example, dendritic cells are recruited, T cell response is regulated, and antitumor cell cytotoxicity is exerted; thus, the immunotherapy based on NK cells is also under intensive focus [67–69]. Additionally, as key mediators eliminating tumor cells and important targets for immune modulation in immunotherapy of tumor, T cells infiltrates were classified into two groups. The densities of most T cells decreased in high-risk group, whereas those of the CD4+ resting memory cells were higher [70]. Tfh cell is also a critical immune regulator in several solid tumors, which induced diminished immunosuppression that was positively correlated with favorable survival in breast cancer and colorectal cancer [71, 72]. Since the role of Tfh cells in EOC is unknown, we proposed a protective role of the cells in EOC microenvironment. Plasma cells have been considered to be predictors of a favorable clinical outcome in several solid tumors, which showed a higher fraction in the low-risk group patients [73]. Kroeger et al. reported that the plasma cells were associated with CD8^+^ tumor-infiltrating lymphocyte responses in EOC and facilitated antitumor immune responses [74]. Consistent with these findings, our results also identified the protective role of plasma cells in EOC tissues, with lesser infiltration in high-risk group patients.

In this study, multiple IRGs were identified, and a novel classifier based on IRGs was established, with CIBERSORT algorithms used to identify the immune infiltration. Our results provided preliminary insight into the role of immune infiltrations in EOC and provided ideas for further diagnosis, evaluation, and even targeted therapy. Nevertheless, the present study has some limitations. First, the biological functions and molecular functions of the classifier genes need further verification using *in vitro* and *vivo* experiments, especially on the correlation with immune infiltration and prognosis. Second, as a retrospective study, the results need to be substantiated further in other cohorts and verified by a prospective study.

In conclusion, we provided a comprehensive insight into TME and constructed a risk model utilizing the expression profile of 9 IRGs and clinical data acquired from the TCGA database to predict the prognosis of patients with EOC. The expression of each biomarker is related to the components in TME and affects the outcomes of patients. However, further prospective studies are required to explore the mechanism and verify future applicability.

## MATERIALS AND METHODS

### Data source and analysis

The gene expression profiles (TOIL RSEM expected count and fragments per kilobase million (FPKM)) of ovarian cancer samples from TCGA database (https://xenabrowser.net/datapages/) and normal ovarian tissue from GTEx database (https://www.gtexportal.org/home/) processed by the Toil pipeline were acquired from the UCSC Xena platform (https://xenabrowser.net/datapages/). After batch effects had been removed, we further extracted data by Perl script [75] (Perl software, version 5.24.3). Also, the clinical information, including age, tumor grade, tumor stage, and survival time, was collected from UCSC Xena website.

In addition, a list of IRGs containing a total of 2498 genes was obtained from the Immunology Database and Analysis Portal (ImmPort database, https://immport.niaid.nih.gov/home), which includes 17 immune categories based on various molecular functions in immune pathways (Supplementary Table 1) [76]. These IRGs were applied to select the related genes to construct the immune-related risk model. Furthermore, a total of 318 TFs were obtained from the Cistrome Cancer database (http://cistrome.org/CistromeCancer/CancerTarget/), a valuable data source to the cancer gene regulation community (Supplementary Table 2) [77].

### Evaluation of immune cell infiltration

The TIICs in EOC samples from the TCGA cohort and normal ovarian tissue from GTEx database were calculated using the CIBERSORT deconvolution algorithm [78]. CIBERSORT used the leukocyte gene signature matrix (LM22), which includes a set of barcode gene expression signature matrix of 547 marker genes for quantifying 22 TIICs (https://cibersortx.stanford.edu/) [79]. In order to improve the accuracy of the CIBERSORT algorithm, P-value and root mean squared error were enumerated for each sample. The default signature matrix was set to 100 permutations. Thus, only samples with a CIBERSORT P<0.05 were considered to be efficient, and hence, selected for subsequent analysis. Next, we analyzed a proportion matrix for the 22 TIICs in EOC tissues based on the high- and low-risk score groups of tumor tissues. Furthermore, the correlation between each prognostic IRGs and TIICs was also estimated according to the TIMER database (https://cistrome.shinyapps.io/timer/) [80].

### Differential expression analyses

The limma package in R was applied to identify differentially expressed genes (DEGs) between tumor and normal tissues [81]. The genes with logFC (log fold change) | ≥2 and false discovery rate (FDR) <0.05 were considered as the cutoff values to filter DEGs. Then, differentially expressed IRGs and TFs were extracted from DEGs. We found that 501 differentially expressed IRGs (292 upregulated genes and 209 downregulated genes) and 76 TFs (41 upregulated TFs and 35 downregulated TFs) were selected. Heatmaps were generated using pheatmap package, and volcano plots were constructed in R software.

To further understand the function of DEGs in EOC, we performed enrichment analyses through Kyoto Encyclopedia of Genes and Genomes (KEGG) and Gene Ontology (GO) databases, including biological process (BP), molecular function (MF), and cellular component (CC) [82, 83]. All the analyses were performed using the cluster Profiler package in R software [84].

### TF regulatory network of the differentially expressed IRGs

Differentially expressed IRGs were significantly associated with the prognosis of EOC patients and were identified as prognostic IRGs by univariate Cox analysis. Since TFs directly regulate gene expression, it is essential to explore their potential in regulating the prognostic IRGs. Herein, we investigated the potential inner TF regulatory network by selecting clinically relevant TFs from the cistrome project and explored the correlation via univariate COX regression. The Cytoscape (version 3.7.2) was used to construct and visualize the regulatory network, including TFs and IRGs [85].

### Construction of an immune prognostic model

An immune-related gene prognostic model was developed using the TCGA and ImmPort dataset [86]. A total of 23 candidate IRGs were further filtered by univariate Cox regression, and P-value <0.01 indicated statistical significance. Furthermore, 9 immune-related genes were ultimately selected for a risk prognosis model through multivariate Cox regression analysis. The expression of candidate IRGs on the translational level was explored using The Human Protein Atlas (HPA) Database (http://www.proteinatlas.org). The risk scores were acquired according to the computational formula encompassing related gene expression and regression coefficient. The EOC patients were divided into high-risk and low-risk groups according to the median risk score to perform subsequent analysis. The receiver operating characteristic (ROC) curve was generated using the R package “survival ROC” to evaluate the accuracy of the prognostic prediction model.

### Survival analysis

The survival time data of 396 patients followed for >90 days were collected from UCSC Xena website. The Kaplan–Meier (KM) survival analysis demonstrated the overall survival (OS) of patients with different risk groups of heterogeneous TIICs, which were stratified by our immune-related signature. The R package called “survival” was used to perform the survival analysis.

### Statistical analysis

R software (R version 3.6.0) was used to perform all statistical analyses, and P<0.05 was considered statistically significant without special instructions. KM curves and log-rank tests were used to analyze the survival data. The univariate Cox regression analysis was used to identify the survival of patients diagnosed with OC, while multivariate Cox regression analysis was used to identify independent prognostic factors.

## Supplementary Material

Supplementary Figures

Supplementary Table 1

Supplementary Table 2

Supplementary Table 3

Supplementary Table 4
