# Supplementary material for: A novel immune-related prognostic signature in epithelial ovarian carcinoma
Source: Aging (Albany NY). 2021 Apr 4;13(7):10289–311. doi: 10.18632/aging.202792 (PMC8064207; doi:10.18632/aging.202792)
Supplement: Supplementary Figures [file aging-13-202792-s001.pdf]

SUPPLEMENTARY FIGURES

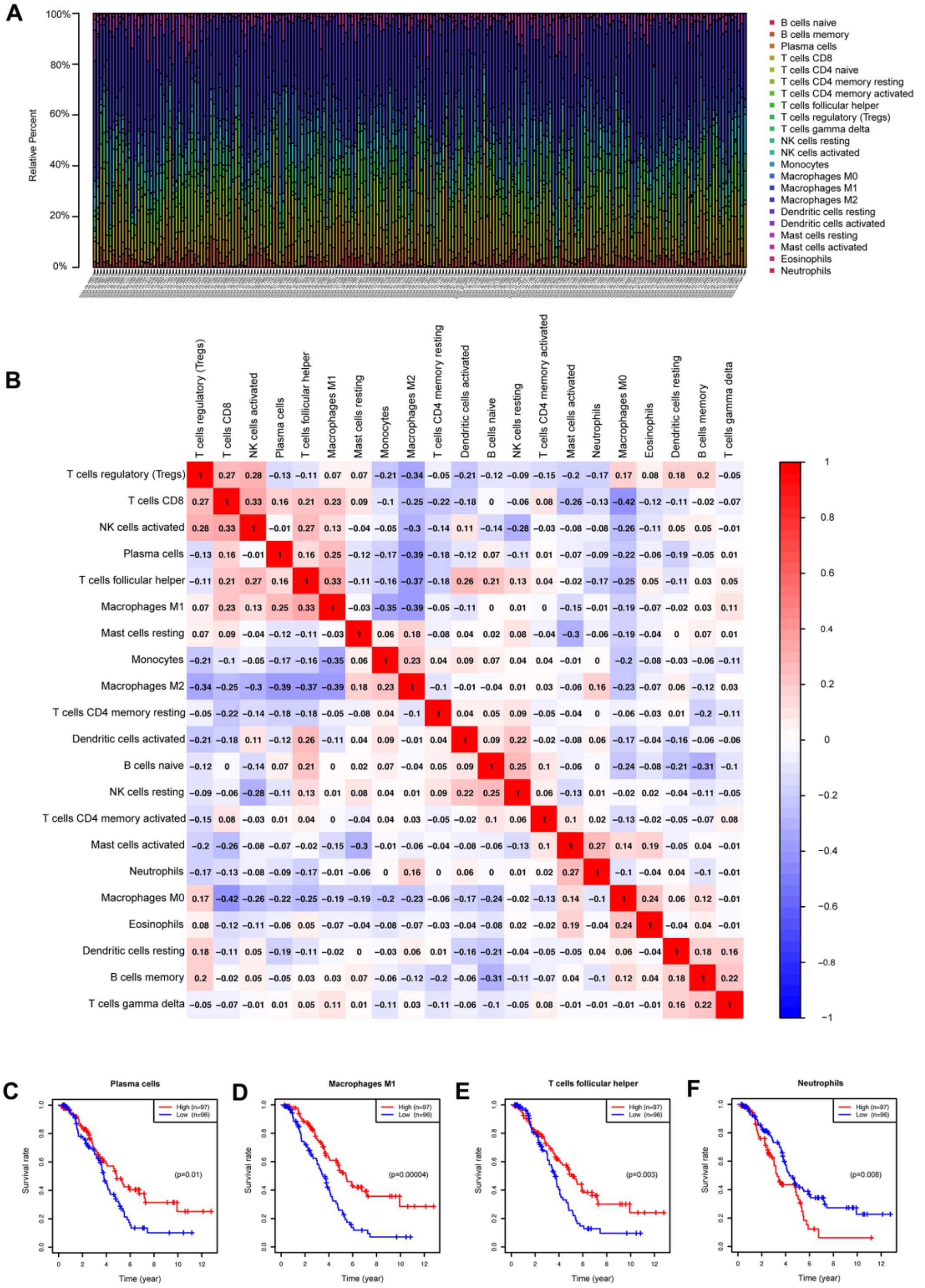

Supplementary Figure 1. The varied proportions of 22 subtypes of immune cells in the tumor and normal samples. (**A**) The relative percentage of 22 subtypes of immune cells. (**B**) The correlation between infiltrating immune cells in EOC tissues (**C**–**F**) KM curves to compare the OS of high expression and low expression of plasma cells, macrophages, Tfh, and neutrophils, respectively.

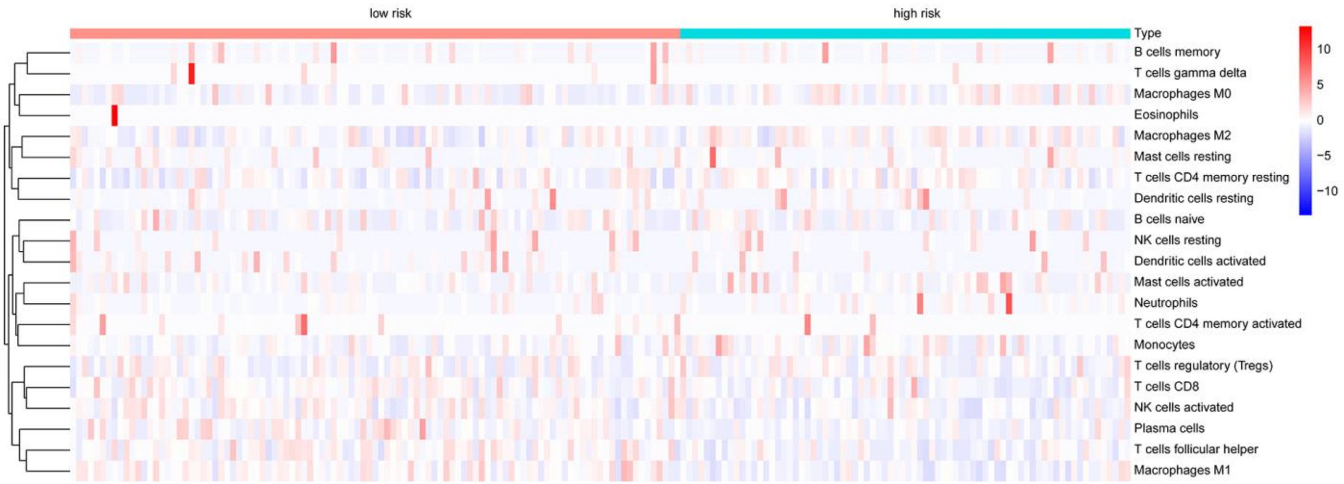

Supplementary Figure 2. Heatmap of 22 immune infiltration cells in the high- and low-risk groups of the EOC tissues.

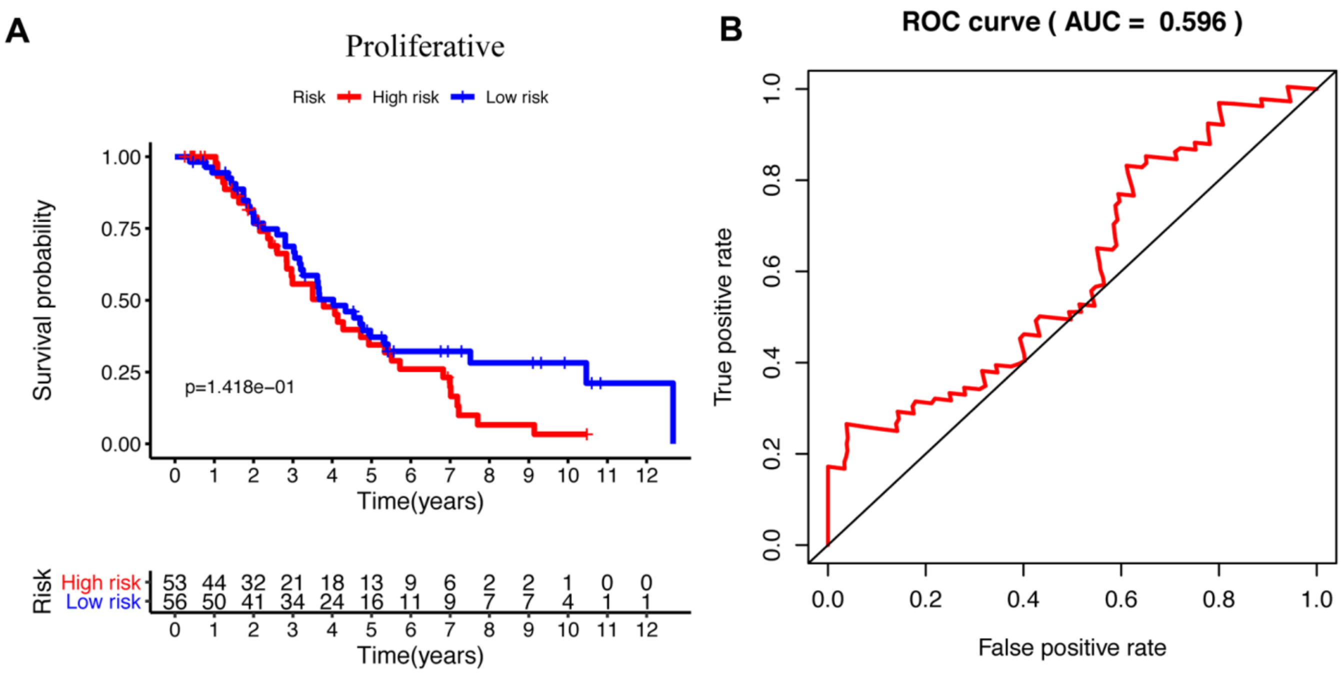

Supplementary Figure 3. The efficacy of signature in diverse subtypes according to the DEGs. (**A**) KM curve analysis of the high- and low- groups in the "proliferative" subtype. (**B**) Time-dependent ROC curves analysis of the prognostic model in the "proliferative" subtype.

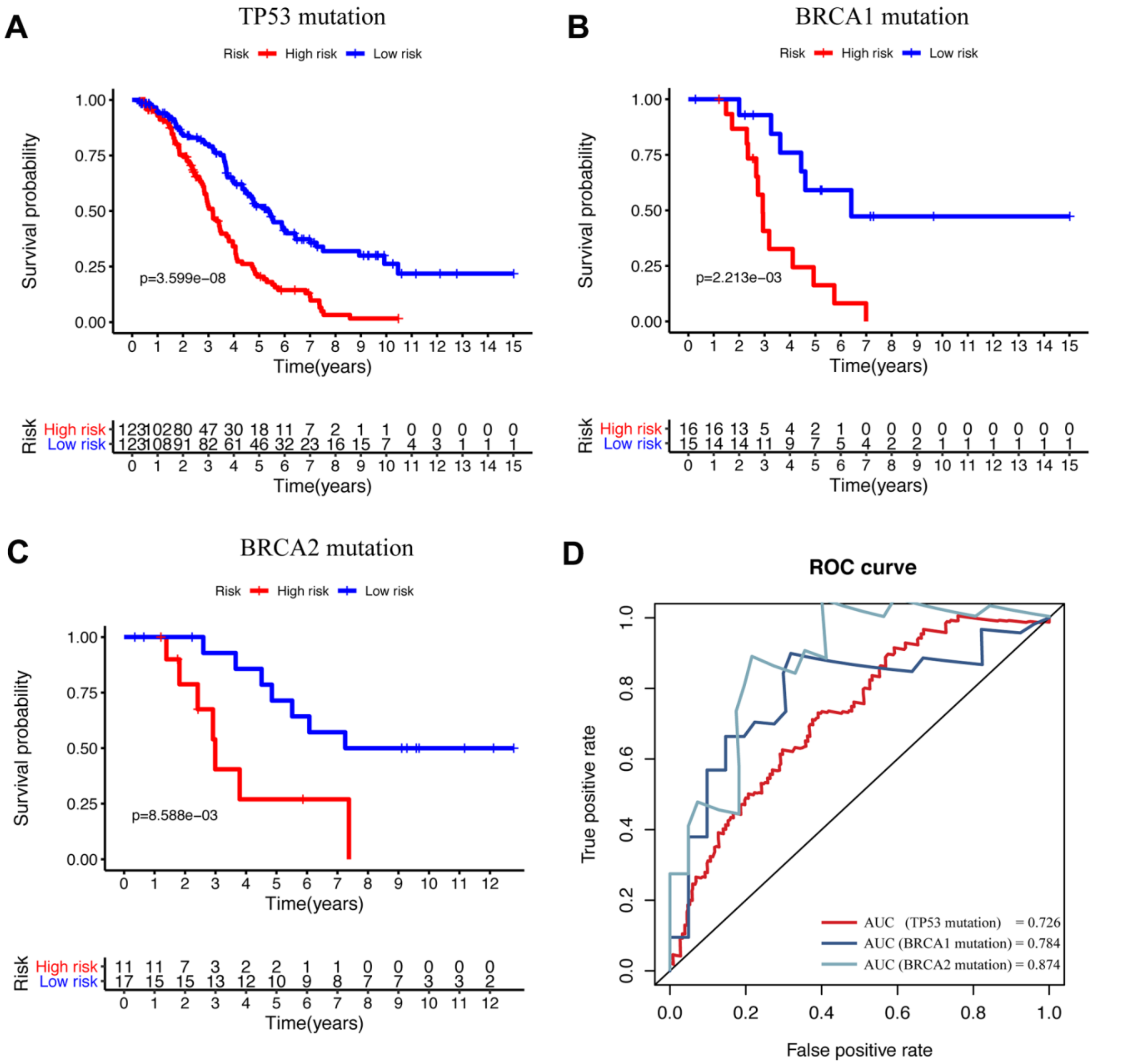

Supplementary Figure 4. The efficacy of signature in diverse subtypes according to the three significant genetic mutations. (**A**–**C**) KM curve analysis of the high- and low-risk groups in samples with TP53, BRCA1, and BRCA2 mutations. (**D**) Time-dependent ROC curves analysis of the prognostic model in samples with TP53, BRCA1, and BRCA2 mutations.
