# Supplementary material for: A novel immune-related prognostic signature in epithelial ovarian carcinoma
Source: Aging (Albany NY). 2021 Apr 4;13(7):10289–311. doi: 10.18632/aging.202792 (PMC8064207; doi:10.18632/aging.202792)
Supplement: Supplementary Table 1 [file aging-13-202792-s002.doc]

Supplementary Table 1. The table of immune-related genes from IMMPORT database.

| Symbol | Synonyms | Chromosome | Category |
| --- | --- | --- | --- |
| AZGP1 | ZA2G|ZAG | 7 | Antigen_Processing_and_Presentation |
| B2M | - | 15 | Antigen_Processing_and_Presentation |
| CALR | CRT|FLJ26680|RO|SSA|cC1qR | 19 | Antigen_Processing_and_Presentation |
| CANX | CNX|FLJ26570|IP90|P90 | 5 | Antigen_Processing_and_Presentation |
| CD1A | CD1|FCB6|HTA1|R4|T6 | 1 | Antigen_Processing_and_Presentation |
| CD1B | CD1|CD1A|MGC125990|MGC125991|R1 | 1 | Antigen_Processing_and_Presentation |
| CD1C | BDCA1|CD1|CD1A|R7 | 1 | Antigen_Processing_and_Presentation |
| CD1D | CD1A|MGC34622|R3 | 1 | Antigen_Processing_and_Presentation |
| CD1E | CD1A|R2 | 1 | Antigen_Processing_and_Presentation |
| CD4 | CD4mut | 12 | Antigen_Processing_and_Presentation |
| CD8A | CD8|Leu2|MAL|p32 | 2 | Antigen_Processing_and_Presentation |
| CD8B | CD8B1|LYT3|Leu2|Ly3|MGC119115 | 2 | Antigen_Processing_and_Presentation |
| CD74 | DHLAG|HLADG|Ia-GAMMA | 5 | Antigen_Processing_and_Presentation |
| CREB1 | CREB|MGC9284 | 2 | Antigen_Processing_and_Presentation |
| CTSB | APPS|CPSB | 8 | Antigen_Processing_and_Presentation |
| CTSE | CATE | 1 | Antigen_Processing_and_Presentation |
| CTSL1 | CATL|CTSL|FLJ31037|MEP | 9 | Antigen_Processing_and_Presentation |
| CTSS | MGC3886 | 1 | Antigen_Processing_and_Presentation |
| FCER1G | FCRG | 1 | Antigen_Processing_and_Presentation |
| FCGRT | FCRN|alpha-chain | 19 | Antigen_Processing_and_Presentation |
| PDIA3 | ER60|ERp57|ERp60|ERp61|GRP57|GRP58|HsT17083|P58|PI-PLC | 15 | Antigen_Processing_and_Presentation |
| HFE | HFE1|HH|HLA-H|MGC103790|MVCD7|dJ221C16.10.1 | 6 | Antigen_Processing_and_Presentation |
| HLA-A | FLJ26655|HLAA | 6 | Antigen_Processing_and_Presentation |
| HLA-B | AS|HLA-B-7301|HLA-B73|HLAB|HLAC|MGC111087|SPDA1 | 6 | Antigen_Processing_and_Presentation |
| HLA-C | D6S204|FLJ27082|HLA-Cw|HLA-Cw12|HLA-JY3|HLC-C|PSORS1 | 6 | Antigen_Processing_and_Presentation |
| HLA-DMA | D6S222E|DMA|HLADM|RING6 | 6 | Antigen_Processing_and_Presentation |
| HLA-DMB | D6S221E|RING7 | 6 | Antigen_Processing_and_Presentation |
| HLA-DOA | HLA-DNA|HLA-DZA|HLADZ | 6 | Antigen_Processing_and_Presentation |
| HLA-DOB | DOB | 6 | Antigen_Processing_and_Presentation |
| HLA-DPA1 | HLA-DP1A|HLADP|HLASB | 6 | Antigen_Processing_and_Presentation |
| HLA-DPB1 | DPB1|HLA-DP1B | 6 | Antigen_Processing_and_Presentation |
| HLA-DQA1 | CD|CELIAC1|DQ-A1|FLJ27088|FLJ27328|GSE|HLA-DQA|MGC149527 | 6 | Antigen_Processing_and_Presentation |
| HLA-DQA2 | HLA-DXA | 6 | Antigen_Processing_and_Presentation |
| HLA-DQB1 | CELIAC1|HLA-DQB|IDDM1 | 6 | Antigen_Processing_and_Presentation |
| HLA-DRA | HLA-DRA1 | 6 | Antigen_Processing_and_Presentation |
| HLA-DRB1 | DRB1|FLJ75017|FLJ76359|HLA-DR1B|HLA-DRB|HLA-DRB1*|SS1 | 6 | Antigen_Processing_and_Presentation |
| HLA-DRB3 | HLA-DR3B|HLA-DR52|MGC117330 | 6 | Antigen_Processing_and_Presentation |
| HLA-DRB4 | DRB4|HLA-DR4B | 6 | Antigen_Processing_and_Presentation |
| HLA-DRB5 | FLJ76359|HLA-DRB | 6 | Antigen_Processing_and_Presentation |
| HLA-E | DKFZp686P19218|EA1.2|EA2.1|HLA-6.2|MHC|QA1 | 6 | Antigen_Processing_and_Presentation |
| HLA-F | CDA12|HLA-5.4|HLA-CDA12|HLAF | 6 | Antigen_Processing_and_Presentation |
| HLA-G | MHC-G | 6 | Antigen_Processing_and_Presentation |
| HLA-H | HLAHP | 6 | Antigen_Processing_and_Presentation |
| MR1 | HLALS | 1 | Antigen_Processing_and_Presentation |
| HSPA1A | FLJ54303|FLJ54370|FLJ54392|FLJ54408|FLJ75127|HSP70-1|HSP70-1A|HSP70I|HSP72|HSPA1|HSPA1B | 6 | Antigen_Processing_and_Presentation |
| HSPA1B | FLJ54328|HSP70-1B|HSP70-2|HSPA1A | 6 | Antigen_Processing_and_Presentation |
| HSPA1L | HSP70-1L|HSP70-HOM|HSP70T|hum70t | 6 | Antigen_Processing_and_Presentation |
| HSPA2 | HSP70-2|HSP70-3 | 14 | Antigen_Processing_and_Presentation |
| HSPA4 | APG-2|HS24/P52|MGC131852|RY|hsp70|hsp70RY | 5 | Antigen_Processing_and_Presentation |
| HSPA5 | BIP|FLJ26106|GRP78|MIF2 | 9 | Antigen_Processing_and_Presentation |
| HSPA6 | - | 1 | Antigen_Processing_and_Presentation |
| HSPA8 | HSC54|HSC70|HSC71|HSP71|HSP73|HSPA10|LAP1|MGC131511|MGC29929|NIP71 | 11 | Antigen_Processing_and_Presentation |
| HSP90AA1 | FLJ31884|HSP86|HSP89A|HSP90A|HSP90N|HSPC1|HSPCA|HSPCAL1|HSPCAL4|HSPN|Hsp89|Hsp90|LAP2 | 14 | Antigen_Processing_and_Presentation |
| HSP90AB1 | D6S182|FLJ26984|HSP90-BETA|HSP90B|HSPC2|HSPCB | 6 | Antigen_Processing_and_Presentation |
| ICAM1 | BB2|CD54|P3.58 | 19 | Antigen_Processing_and_Presentation |
| IFNA1 | IFL|IFN|IFN-ALPHA|IFNA13|IFNA@|MGC138207|MGC138505|MGC138507 | 9 | Antigen_Processing_and_Presentation |
| IFNA2 | IFNA|INFA2|MGC125764|MGC125765 | 9 | Antigen_Processing_and_Presentation |
| IFNA4 | INFA4|MGC142200 | 9 | Antigen_Processing_and_Presentation |
| IFNA5 | INFA5 | 9 | Antigen_Processing_and_Presentation |
| IFNA6 | - | 9 | Antigen_Processing_and_Presentation |
| IFNA7 | IFNA-J | 9 | Antigen_Processing_and_Presentation |
| IFNA8 | - | 9 | Antigen_Processing_and_Presentation |
| IFNA10 | MGC119878|MGC119879 | 9 | Antigen_Processing_and_Presentation |
| IFNA13 | - | 9 | Antigen_Processing_and_Presentation |
| IFNA14 | LEIF2H|MGC125756|MGC125757 | 9 | Antigen_Processing_and_Presentation |
| IFNA16 | - | 9 | Antigen_Processing_and_Presentation |
| IFNA17 | IFNA|INFA|LEIF2C1 | 9 | Antigen_Processing_and_Presentation |
| IFNA21 | MGC126687|MGC126689 | 9 | Antigen_Processing_and_Presentation |
| IFNG | IFG|IFI | 12 | Antigen_Processing_and_Presentation |
| KIR2DL1 | CD158A|KIR-K64|KIR221|NKAT|NKAT1|p58.1 | 19 | Antigen_Processing_and_Presentation |
| KIR2DL2 | CD158B1|CD158b|NKAT6|p58.2 | 19 | Antigen_Processing_and_Presentation |
| KIR2DL3 | CD158B2|CD158b|GL183|KIR-023GB|KIR-K7b|KIR-K7c|KIRCL23|MGC129943|NKAT|NKAT2|NKAT2A|NKAT2B|p58 | 19 | Antigen_Processing_and_Presentation |
| KIR2DL4 | CD158D|G9P|KIR103|KIR103AS | 19 | Antigen_Processing_and_Presentation |
| KIR2DS1 | CD158H|CD158a|p50.1 | 19 | Antigen_Processing_and_Presentation |
| KIR2DS3 | NKAT7 | 19 | Antigen_Processing_and_Presentation |
| KIR2DS4 | CD158I|KIR1D|KIR412|KKA3|MGC120019|MGC125315|MGC125317|NKAT8 | 19 | Antigen_Processing_and_Presentation |
| KIR2DS5 | CD158G|NKAT9 | 19 | Antigen_Processing_and_Presentation |
| KIR3DL1 | CD158E1|KIR|MGC119726|MGC119728|MGC126589|MGC126591|NKAT3|NKB1|NKB1B | 19 | Antigen_Processing_and_Presentation |
| KIR3DL2 | CD158K|MGC125321|NKAT4|NKAT4B|p140 | 19 | Antigen_Processing_and_Presentation |
| KLRC1 | CD159A|MGC13374|MGC59791|NKG2|NKG2A | 12 | Antigen_Processing_and_Presentation |
| KLRC2 | CD159c|MGC138244|NKG2-C|NKG2C | 12 | Antigen_Processing_and_Presentation |
| KLRC3 | NKG2-E|NKG2E | 12 | Antigen_Processing_and_Presentation |
| KLRD1 | CD94 | 12 | Antigen_Processing_and_Presentation |
| LTA | LT|TNFB|TNFSF1 | 6 | Antigen_Processing_and_Presentation |
| CIITA | C2TA|CIITAIV|MHC2TA|NLRA | 16 | Antigen_Processing_and_Presentation |
| MICA | FLJ60820|MGC111087|PERB11.1 | 6 | Antigen_Processing_and_Presentation |
| MICB | PERB11.2 | 6 | Antigen_Processing_and_Presentation |
| NFYA | CBF-A|CBF-B|FLJ11236|HAP2|NF-YA | 6 | Antigen_Processing_and_Presentation |
| NFYB | CBF-A|CBF-B|HAP3|NF-YB | 12 | Antigen_Processing_and_Presentation |
| NFYC | CBF-C|CBFC|DKFZp667G242|FLJ45775|H1TF2A|HAP5|HSM|NF-YC | 1 | Antigen_Processing_and_Presentation |
| LGMN | AEP|LGMN1|PRSC1 | 14 | Antigen_Processing_and_Presentation |
| PSMB8 | D6S216|D6S216E|LMP7|MGC1491|PSMB5i|RING10|beta5i | 6 | Antigen_Processing_and_Presentation |
| PSMC1 | MGC24583|MGC8541|P26S4|S4|p56 | 14 | Antigen_Processing_and_Presentation |
| PSMC2 | MGC3004|MSS1|Nbla10058|S7 | 7 | Antigen_Processing_and_Presentation |
| PSMC3 | MGC8487|TBP1 | 11 | Antigen_Processing_and_Presentation |
| PSMC4 | MGC13687|MGC23214|MGC8570|MIP224|S6|TBP7 | 19 | Antigen_Processing_and_Presentation |
| PSMC5 | S8|SUG-1|SUG1|TBP10|TRIP1|p45|p45/SUG | 17 | Antigen_Processing_and_Presentation |
| PSMC6 | CADP44|MGC12520|P44|SUG2|p42 | 14 | Antigen_Processing_and_Presentation |
| PSMD1 | MGC133040|MGC133041|P112|Rpn2|S1 | 2 | Antigen_Processing_and_Presentation |
| PSMD2 | MGC14274|P97|Rpn1|S2|TRAP2 | 3 | Antigen_Processing_and_Presentation |
| PSMD3 | P58|RPN3|S3 | 17 | Antigen_Processing_and_Presentation |
| PSMD4 | AF|AF-1|ASF|MCB1|Rpn10|S5A|pUB-R5 | 1 | Antigen_Processing_and_Presentation |
| PSMD5 | KIAA0072|MGC23145|S5B | 9 | Antigen_Processing_and_Presentation |
| PSMD7 | MOV34|P40|Rpn8|S12 | 16 | Antigen_Processing_and_Presentation |
| PSMD8 | HIP6|HYPF|MGC1660|Nin1p|Rpn12|S14|p31 | 19 | Antigen_Processing_and_Presentation |
| PSMD10 | dJ889N15.2|p28 | X | Antigen_Processing_and_Presentation |
| PSMD11 | MGC3844|Rpn6|S9|p44.5 | 17 | Antigen_Processing_and_Presentation |
| PSMD13 | HSPC027|Rpn9|S11|p40.5 | 11 | Antigen_Processing_and_Presentation |
| PSME1 | IFI5111|MGC8628|PA28A|PA28alpha|REGalpha | 14 | Antigen_Processing_and_Presentation |
| PSME1 | IFI5111|MGC8628|PA28A|PA28alpha|REGalpha | 14 | Antigen_Processing_and_Presentation |
| PSME2 | PA28B|PA28beta|REGbeta | 14 | Antigen_Processing_and_Presentation |
| PSME2 | PA28B|PA28beta|REGbeta | 14 | Antigen_Processing_and_Presentation |
| RELB | I-REL|IREL | 19 | Antigen_Processing_and_Presentation |
| RFX5 | - | 1 | Antigen_Processing_and_Presentation |
| RFXAP | - | 13 | Antigen_Processing_and_Presentation |
| SLC10A2 | ASBT|ISBT|NTCP2 | 13 | Antigen_Processing_and_Presentation |
| TAP1 | ABC17|ABCB2|APT1|D6S114E|FLJ26666|FLJ41500|PSF1|RING4|TAP1*0102N|TAP1N | 6 | Antigen_Processing_and_Presentation |
| TAP2 | ABC18|ABCB3|APT2|D6S217E|PSF2|RING11 | 6 | Antigen_Processing_and_Presentation |
| TAPBP | NGS17|TAPA|TPN|TPSN|tapasin | 6 | Antigen_Processing_and_Presentation |
| THBS1 | THBS|THBS-1|TSP|TSP-1|TSP1 | 15 | Antigen_Processing_and_Presentation |
| SHFM1 | DSS1|ECD|SEM1|SHFD1|SHSF1|Shfdg1 | 7 | Antigen_Processing_and_Presentation |
| KLRC4 | FLJ17759|FLJ78582|NKG2-F|NKG2F | 12 | Antigen_Processing_and_Presentation |
| AP3B1 | ADTB3|ADTB3A|HPS|HPS2|PE | 5 | Antigen_Processing_and_Presentation |
| RFXANK | ANKRA1|BLS|F14150_1|MGC138628|RFX-B | 19 | Antigen_Processing_and_Presentation |
| PSMD6 | KIAA0107|Rpn7|S10|SGA-113M|p44S10 | 3 | Antigen_Processing_and_Presentation |
| PSME3 | Ki|PA28-gamma|PA28G|REG-GAMMA | 17 | Antigen_Processing_and_Presentation |
| PSMD14 | PAD1|POH1|rpn11 | 2 | Antigen_Processing_and_Presentation |
| CLEC4M | CD209L|CD299|DC-SIGN2|DC-SIGNR|DCSIGNR|HP10347|L-SIGN|LSIGN|MGC129964|MGC47866 | 19 | Antigen_Processing_and_Presentation |
| IFI30 | GILT|IFI-30|IP30|MGC32056 | 19 | Antigen_Processing_and_Presentation |
| PROCR | CCCA|CCD41|CD201|EPCR|MGC23024|bA42O4.2 | 20 | Antigen_Processing_and_Presentation |
| ADRM1 | ARM1|GP110|MGC29536|Rpn13 | 20 | Antigen_Processing_and_Presentation |
| KIAA0368 | ECM29|FLJ22036|KIAA1962|RP11-386D8.2 | 9 | Antigen_Processing_and_Presentation |
| TRPC4AP | C20orf188|TRRP4AP|TRUSS | 20 | Antigen_Processing_and_Presentation |
| CD209 | CDSIGN|CLEC4L|DC-SIGN|DC-SIGN1|MGC129965 | 19 | Antigen_Processing_and_Presentation |
| UBXN1 | 2B28|UBXD10 | 11 | Antigen_Processing_and_Presentation |
| ERAP1 | A-LAP|ALAP|APPILS|ARTS-1|ARTS1|ERAAP|ERAAP1|KIAA0525|PILS-AP|PILSAP | 5 | Antigen_Processing_and_Presentation |
| TAPBPL | FLJ10143|TAPBP-R|TAPBPR | 12 | Antigen_Processing_and_Presentation |
| KIR2DL5A | CD158F|KIR2DL5|KIR2DL5.1|KIR2DL5.3 | 19 | Antigen_Processing_and_Presentation |
| ERAP2 | FLJ23633|FLJ23701|FLJ23807|L-RAP|LRAP | 5 | Antigen_Processing_and_Presentation |
| ULBP3 | RAET1N | 6 | Antigen_Processing_and_Presentation |
| ULBP2 | N2DL2|RAET1H | 6 | Antigen_Processing_and_Presentation |
| ULBP1 | RAET1I | 6 | Antigen_Processing_and_Presentation |
| KIR3DL3 | CD158Z|KIR3DL7|KIR44|KIRC1 | 19 | Antigen_Processing_and_Presentation |
| RAET1E | LETAL|MGC125308|MGC125309|RAET1E2|ULBP4|bA350J20.7 | 6 | Antigen_Processing_and_Presentation |
| RAET1L | - | 6 | Antigen_Processing_and_Presentation |
| UBR1 | JBS|MGC142065|MGC142067 | 15 | Antigen_Processing_and_Presentation |
| RAET1G | ULBP5 | 6 | Antigen_Processing_and_Presentation |
| PDIA2 | PDA2|PDI|PDIP|PDIR | 16 | Antigen_Processing_and_Presentation |
| HAMP | HEPC|HEPCIDIN|HFE2B|LEAP-1|LEAP1|PLTR | 19 | Antimicrobials |
| PI3 | ESI|MGC13613|SKALP|WAP3|WFDC14|cementoin | 20 | Antimicrobials |
| CAMP | CAP18|CRAMP|FALL-39|FALL39|HSD26|LL37 | 3 | Antimicrobials |
| DEFB4 | DEFB-2|DEFB102|DEFB2|HBD-2|SAP1 | 8 | Antimicrobials |
| PPBP | B-TG1|Beta-TG|CTAP-III|CTAP3|CTAPIII|CXCL7|LA-PF4|LDGF|MDGF|NAP-2|PBP|SCYB7|TC1|TC2|TGB|TGB1|THBGB|THBGB1 | 4 | Antimicrobials |
| REG3G | MGC118998|MGC118999|MGC119001|PAP1B|PAPIB|REG-III|UNQ429 | 2 | Antimicrobials |
| CXCL14 | BMAC|BRAK|KS1|Kec|MGC10687|MIP-2g|NJAC|SCYB14|bolekine | 5 | Antimicrobials |
| CXCL16 | CXCLG16|SR-PSOX|SRPSOX | 17 | Antimicrobials |
| SLPI | ALK1|ALP|BLPI|HUSI|HUSI-I|MPI|WAP4|WFDC4 | 20 | Antimicrobials |
| IL8 | CXCL8|GCP-1|GCP1|LECT|LUCT|LYNAP|MDNCF|MONAP|NAF|NAP-1|NAP1 | 4 | Antimicrobials |
| CXCL10 | C7|IFI10|INP10|IP-10|SCYB10|crg-2|gIP-10|mob-1 | 4 | Antimicrobials |
| CXCL9 | CMK|Humig|MIG|SCYB9|crg-10 | 4 | Antimicrobials |
| CXCL5 | ENA-78|SCYB5 | 4 | Antimicrobials |
| CXCL11 | H174|I-TAC|IP-9|IP9|MGC102770|SCYB11|SCYB9B|b-R1 | 4 | Antimicrobials |
| CXCL6 | CKA-3|GCP-2|GCP2|SCYB6 | 4 | Antimicrobials |
| CXCL1 | FSP|GRO1|GROa|MGSA|MGSA-a|NAP-3|SCYB1 | 4 | Antimicrobials |
| CXCL12 | PBSF|SCYB12|SDF-1a|SDF-1b|SDF1|SDF1A|SDF1B|TLSF-a|TLSF-b|TPAR1 | 10 | Antimicrobials |
| CXCL13 | ANGIE|ANGIE2|BCA-1|BCA1|BLC|BLR1L|SCYB13 | 4 | Antimicrobials |
| CXCL2 | CINC-2a|GRO2|GROb|MGSA-b|MIP-2a|MIP2|MIP2A|SCYB2 | 4 | Antimicrobials |
| PF4 | CXCL4|MGC138298|SCYB4 | 4 | Antimicrobials |
| XCL1 | ATAC|LPTN|LTN|SCM-1|SCM-1a|SCM1|SCYC1 | 1 | Antimicrobials |
| CXCL3 | CINC-2b|GRO3|GROg|MIP-2b|MIP2B|SCYB3 | 4 | Antimicrobials |
| DEFB103A | DEFB103|DEFB3|HBD-3|HBD3|HBP-3|HBP3 | 8 | Antimicrobials |
| CCL13 | CKb10|MCP-4|MGC17134|NCC-1|NCC1|SCYA13|SCYL1 | 17 | Antimicrobials |
| CCL1 | I-309|P500|SCYA1|SISe|TCA3 | 17 | Antimicrobials |
| DEFB1 | BD1|DEFB-1|DEFB101|HBD1|MGC51822 | 8 | Antimicrobials |
| CCL8 | HC14|MCP-2|MCP2|SCYA10|SCYA8 | 17 | Antimicrobials |
| ELANE | ELA2|GE|HLE|HNE|NE|PMN-E | 19 | Antimicrobials |
| DEFB103B | - | 8 | Antimicrobials |
| DEFA3 | DEF3|HNP-3|HNP3|HP-3 | 8 | Antimicrobials |
| DEFA1 | DEF1|DEFA2|HNP-1|HP-1|MGC138393|MRS | 8 | Antimicrobials |
| TMSB10 | MIG12|TB10 | 2 | Antimicrobials |
| DEFA6 | DEF6|HD-6 | 8 | Antimicrobials |
| DEFA5 | DEF5|HD-5|MGC129728 | 8 | Antimicrobials |
| DEFA4 | DEF4|HNP-4|HP-4|HP4|MGC120099|MGC138296 | 8 | Antimicrobials |
| LCN2 | 24p3|NGAL | 9 | Antimicrobials |
| LCN1 | MGC71975|PMFA|TP|VEGP | 9 | Antimicrobials |
| COLEC10 | CLL1|MGC118794|MGC118795 | 8 | Antimicrobials |
| BPI | - | 20 | Antimicrobials |
| S100A9 | 60B8AG|CAGB|CFAG|CGLB|L1AG|LIAG|MAC387|MIF|MRP14|NIF|P14 | 1 | Antimicrobials |
| S100A8 | 60B8AG|CAGA|CFAG|CGLA|CP-10|L1Ag|MA387|MIF|MRP8|NIF|P8 | 1 | Antimicrobials |
| DCD | AIDD|DCD-1|DSEP|HCAP|MGC71930|PIF | 12 | Antimicrobials |
| LCN6 | LCN5|UNQ643|hLcn5 | 9 | Antimicrobials |
| S100A12 | CAAF1|CAGC|CGRP|ENRAGE|MRP6|p6 | 1 | Antimicrobials |
| HTN3 | HIS2|HTN2|HTN5 | 4 | Antimicrobials |
| LCN8 | EP17|LCN5 | 9 | Antimicrobials |
| LOC728358 | - | 8 | Antimicrobials |
| CCR10 | GPR2 | 17 | Antimicrobials |
| CELA1 | ELA1 | 12 | Antimicrobials |
| DEFB106A | BD-6|DEFB-6|DEFB106|MGC118938|MGC118939|MGC118940|MGC118941|MGC133011|MGC133012 | 8 | Antimicrobials |
| PENK | - | 8 | Antimicrobials |
| BPIL2 | - | 22 | Antimicrobials |
| MMP12 | HME|MGC138506|MME | 11 | Antimicrobials |
| BPIL3 | LPLUNC6 | 20 | Antimicrobials |
| LEAP2 | LEAP-2 | 5 | Antimicrobials |
| SFTPD | COLEC7|PSP-D|SFTP4|SP-D | 10 | Antimicrobials |
| LCN9 | 9230102I19Rik | 9 | Antimicrobials |
| BPIL1 | C20orf184|LPLUNC2|RYSR|dJ726C3.2 | 20 | Antimicrobials |
| PTGDS | LPGDS|PDS|PGD2|PGDS|PGDS2 | 9 | Antimicrobials |
| TMSB4X | FX|PTMB4|TB4X|TMSB4 | X | Antimicrobials |
| PGLYRP1 | MGC126894|MGC126896|PGLYRP|PGRP|PGRP-S|PGRPS|TAG7|TNFSF3L | 19 | Antimicrobials |
| ZC3HAV1 | DKFZp686F2052|DKFZp686H1869|DKFZp686O19171|FLB6421|FLJ13288|MGC48898|ZAP|ZC3H2|ZC3HDC2 | 7 | Antimicrobials |
| TMSB15A | TMSB15|TMSL8|TMSNB|Tb15|TbNB | X | Antimicrobials |
| S100B | NEF|S100|S100beta | 21 | Antimicrobials |
| S100A13 | - | 1 | Antimicrobials |
| S100A6 | 2A9|5B10|CABP|CACY|PRA | 1 | Antimicrobials |
| DEFB119 | DEFB-19|DEFB-20|DEFB120|ESC42-RELA|ESC42-RELB|MGC71893 | 20 | Antimicrobials |
| DEFB107A | BD-7|DEFB-7|DEFB107 | 8 | Antimicrobials |
| DEFB105A | BD-5|DEFB-5|DEFB105 | 8 | Antimicrobials |
| SERPIND1 | D22S673|HC2|HCF2|HCII|HLS2|LS2 | 22 | Antimicrobials |
| DEFB129 | C20orf87|DEFB-29|DEFB29|bA530N10.3|hBD-29 | 20 | Antimicrobials |
| DEFB127 | C20orf73|DEF-27|DEFB-27|DEFB27|bA530N10.2|hBD-27 | 20 | Antimicrobials |
| S100P | MIG9 | 4 | Antimicrobials |
| S100A7 | PSOR1|S100A7c | 1 | Antimicrobials |
| DEFB104A | BD-4|DEFB-4|DEFB104|DEFB4|MGC118942|MGC118944|MGC118945|hBD-4 | 8 | Antimicrobials |
| DEFB126 | C20orf8|DEFB-26|DEFB26|ESP13.2|bA530N10.1|hBD-26 | 20 | Antimicrobials |
| DEFB106B | - | 8 | Antimicrobials |
| DEFB104B | - | 8 | Antimicrobials |
| DEFB107B | HsT21816 | 8 | Antimicrobials |
| PGLYRP3 | MGC149197|PGRP-Ialpha|PGRPIA | 1 | Antimicrobials |
| PGLYRP2 | HMFT0141|PGLYRPL|PGRP-L|PGRPL|TAGL-like|tagL|tagL-alpha|tagl-beta | 19 | Antimicrobials |
| S100A10 | 42C|ANX2L|ANX2LG|CAL1L|CLP11|Ca[1]|GP11|MGC111133|P11|p10 | 1 | Antimicrobials |
| S100A2 | CAN19|MGC111539|S100L | 1 | Antimicrobials |
| DEFB125 | DEFB-25|MGC57449 | 20 | Antimicrobials |
| DEFB123 | DEFB-23|ESC42-RELD | 20 | Antimicrobials |
| DEFB105B | - | 8 | Antimicrobials |
| DEFB132 | DEFB32|UNQ827 | 20 | Antimicrobials |
| C20orf185 | LPLUNC3|RYA3|dJ726C3.4 | 20 | Antimicrobials |
| LCN12 | MGC34753|MGC48935 | 9 | Antimicrobials |
| PGLYRP4 | PGLYRPIbeta|PGRP-Ibeta|PGRPIB|SBBI67 | 1 | Antimicrobials |
| S100A11 | MLN70|S100C | 1 | Antimicrobials |
| S100A5 | S100D | 1 | Antimicrobials |
| S100A3 | S100E | 1 | Antimicrobials |
| S100A1 | S100|S100-alpha|S100A | 1 | Antimicrobials |
| DEFB128 | DEFB-28|DEFB28|hBD-28 | 20 | Antimicrobials |
| DEFB108B | - | 11 | Antimicrobials |
| HTN1 | HIS1 | 4 | Antimicrobials |
| LMBR1L | FLJ10494|FLJ36251|KIAA1174|LIMR | 12 | Antimicrobials |
| S100A7A | NICE-2|S100A15|S100A7L1|S100A7f | 1 | Antimicrobials |
| DEFB118 | C20orf63|DEFB-18|ESC42|dJ1018D12.3 | 20 | Antimicrobials |
| COLEC12 | CLP1|NSR2|SCARA4|SRCL | 18 | Antimicrobials |
| TMSB4Y | MGC26307|TB4Y | Y | Antimicrobials |
| DEFB131 | DEFB-31 | 4 | Antimicrobials |
| DEFB134 | MGC163333|MGC163335 | 8 | Antimicrobials |
| DEFB130 | DEFB-30 | 8 | Antimicrobials |
| DEFB124 | DEFB-24 | 20 | Antimicrobials |
| DEFB121 | DEFB-21|ESC42-RELC | 20 | Antimicrobials |
| DEFB116 | DEFB-16 | 20 | Antimicrobials |
| DEFB115 | DEFB-15 | 20 | Antimicrobials |
| DEFB114 | DEFB-14 | 6 | Antimicrobials |
| DEFB113 | DEFB-13 | 6 | Antimicrobials |
| DEFB112 | DEFB-12 | 6 | Antimicrobials |
| DEFB110 | DEFB-10 | 6 | Antimicrobials |
| TMSB15B | MGC39900|Tbeta15b | X | Antimicrobials |
| DEFB133 | - | 12 | Antimicrobials |
| S100Z | Gm625|S100-zeta | 5 | Antimicrobials |
| MAVS | CARDIF|DKFZp547C224|DKFZp666M015|FLJ27482|FLJ35386|FLJ38051|FLJ41962|IPS-1|IPS1|KIAA1271|MGC3260|VISA | 20 | Antimicrobials |
| TMSL3 | - | 4 | Antimicrobials |
| S100A14 | BCMP84|S100A15 | 1 | Antimicrobials |
| LCN10 | - | 9 | Antimicrobials |
| S100A16 | AAG13|DT1P1A7|MGC17528|S100F | 1 | Antimicrobials |
| DEFB137 | DEFB136 | 8 | Antimicrobials |
| DEFB136 | DEFB135 | 8 | Antimicrobials |
| DEFB117 | DEFB-17 | 20 | Antimicrobials |
| DEFB111 | DEFB-11 | 6 | Antimicrobials |
| ZC3HAV1L | C7orf39|MGC14289 | 7 | Antimicrobials |
| S100A7L2 | S100a7b | 1 | Antimicrobials |
| LOC731414 | - | 17 | Antimicrobials |
| LOC730963 | - | 8 | Antimicrobials |
| COLEC2 | MBL | - | Antimicrobials |
| DEFB4P | - | 8 | Antimicrobials |
| C20orf186 | LPLUNC4|RY2G5|dJ726C3.5 | 20 | Antimicrobials |
| IFNAR1 | AVP|IFN-alpha-REC|IFNAR|IFNBR|IFRC | 21 | Antimicrobials |
| AZU1 | AZAMP|AZU|CAP37|HBP|HUMAZUR|NAZC | 19 | Antimicrobials |
| LOC729523 | - | 11 | Antimicrobials |
| LOC100130154 | - | 2 | Antimicrobials |
| LOC100134379 | - | Un | Antimicrobials |
| LOC100134289 | - | 20 | Antimicrobials |
| LOC100129216 | - | 11 | Antimicrobials |
| DEFA1A3 | DEFA1|DEFA3|DEFT1P | 8 | Antimicrobials |
| LOC100131433 | - | 9 | Antimicrobials |
| LCN1L1 | bA430N14.2 | 9 | Antimicrobials |
| S100G | CABP1|CABP9K|CALB3|MGC138379 | X | Antimicrobials |
| LOC648637 | - | 8 | Antimicrobials |
| LOC100130969 | - | 9 | Antimicrobials |
| LOC100133267 | - | 8 | Antimicrobials |
| LOC100133128 | - | 4 | Antimicrobials |
| LOC100128174 | - | 8 | Antimicrobials |
| TCHHL1 | S100A17|THHL1|basalin | 1 | Antimicrobials |
| TINAGL1 | ARG1|LCN7|LIECG3|TINAGRP | 1 | Antimicrobials |
| IFNGR1 | CD119|FLJ45734|IFNGR | 6 | Antimicrobials |
| SLC22A17 | BOCT|BOIT|NGALR|hBOIT | 14 | Antimicrobials |
| WFIKKN1 | C16orf12|MGC126651|MGC126655|RJD2|WFIKKN | 16 | Antimicrobials |
| WFDC2 | HE4|MGC57529|WAP5|dJ461P17.6 | 20 | Antimicrobials |
| IL6 | BSF2|HGF|HSF|IFNB2|IL-6 | 7 | Antimicrobials |
| UMODL1 | - | 21 | Antimicrobials |
| TGFB1 | CED|DPD1|TGFB|TGFbeta | 19 | Antimicrobials |
| PF4V1 | CXCL4L1|CXCL4V1|PF4-ALT|PF4A|SCYB4V1 | 4 | Antimicrobials |
| MMP9 | CLG4B|GELB|MMP-9 | 20 | Antimicrobials |
| KAL1 | ADMLX|HHA|KAL|KALIG-1|KMS | X | Antimicrobials |
| TLR4 | ARMD10|CD284|TOLL|hToll | 9 | Antimicrobials |
| IFNG | IFG|IFI | 12 | Antimicrobials |
| SPAG11B | EP2|EP2C|EP2D|HE2|HE2C|MGC61846|SPAG11 | 8 | Antimicrobials |
| A2M | CPAMD5|DKFZp779B086|FWP007|S863-7 | 12 | Antimicrobials |
| CTSL1 | CATL|CTSL|FLJ31037|MEP | 9 | Antimicrobials |
| NFKB1 | DKFZp686C01211|EBP-1|KBF1|MGC54151|NF-kappa-B|NFKB-p105|NFKB-p50|p105|p50 | 4 | Antimicrobials |
| APOBEC3G | ARP9|CEM15|FLJ12740|MDS019|bK150C2.7|dJ494G10.1 | 22 | Antimicrobials |
| FABP6 | I-15P|I-BABP|I-BALB|I-BAP|ILBP|ILBP3|ILLBP | 5 | Antimicrobials |
| NOD2 | ACUG|BLAU|CARD15|CD|CLR16.3|IBD1|NLRC2|NOD2B|PSORAS1 | 16 | Antimicrobials |
| MBL2 | COLEC1|HSMBPC|MBL|MBP|MBP1|MGC116832|MGC116833 | 10 | Antimicrobials |
| SFTPA1B | AC068139.6|MGC133365|PSAP|PSPA|SFTP1|SFTPA1 | 10 | Antimicrobials |
| RBP1 | CRABP-I|CRBP|CRBP1|CRBPI|RBPC | 3 | Antimicrobials |
| TLR2 | CD282|TIL4 | 4 | Antimicrobials |
| SLC40A1 | FPN1|HFE4|IREG1|MST079|MSTP079|MTP1|SLC11A3 | 2 | Antimicrobials |
| PLAU | ATF|UPA|URK|u-PA | 10 | Antimicrobials |
| IL1B | IL-1|IL1-BETA|IL1F2 | 2 | Antimicrobials |
| PAEP | GD|GdA|GdF|GdS|MGC138509|MGC142288|PAEG|PEP|PP14 | 9 | Antimicrobials |
| HFE2 | HFE2A|HJV|JH|MGC23953|RGMC | 1 | Antimicrobials |
| MUC5AC | MUC5 | 11 | Antimicrobials |
| CTSS | MGC3886 | 1 | Antimicrobials |
| OBP2A | OBP|OBP2C|OBPIIa|hOBPIIa | 9 | Antimicrobials |
| PLTP | HDLCQ9 | 20 | Antimicrobials |
| MX1 | IFI-78K|IFI78|MX|MxA | 21 | Antimicrobials |
| DDX58 | DKFZp434J1111|DKFZp686N19181|FLJ13599|RIG-I | 9 | Antimicrobials |
| IL29 | IFNL1|IL-29 | 19 | Antimicrobials |
| IRF3 | - | 19 | Antimicrobials |
| SFTPA2 | COLEC5|MGC189761|SP-A2|SPA2|SPAII | 10 | Antimicrobials |
| SFTPA2B | AC068139.3|SFTPA2|SP-2A|SP-A1|SP-A2|SPAII | 10 | Antimicrobials |
| LPA | AK38|APOA|LP | 6 | Antimicrobials |
| LBP | MGC22233 | 20 | Antimicrobials |
| RBP4 | - | 10 | Antimicrobials |
| SFTPA1 | COLEC4|FLJ51913|SFTP1|SP-A|SP-A1 | 10 | Antimicrobials |
| NOX4 | KOX|KOX-1|RENOX | 11 | Antimicrobials |
| LTF | GIG12|HLF2|LF | 3 | Antimicrobials |
| IFNB1 | IFB|IFF|IFNB|MGC96956 | 9 | Antimicrobials |
| RBP5 | CRBP-III|CRBP3|CRBPIII | 12 | Antimicrobials |
| FABP7 | B-FABP|BLBP|DKFZp547J2313|FABPB|MRG | 6 | Antimicrobials |
| FABP5 | E-FABP|EFABP|PA-FABP|PAFABP | 8 | Antimicrobials |
| FABP3 | FABP11|H-FABP|MDGI|O-FABP | 1 | Antimicrobials |
| FABP2 | FABPI|I-FABP|MGC133132 | 4 | Antimicrobials |
| FABP4 | A-FABP|aP2 | 8 | Antimicrobials |
| R3HDML | MGC129564|dJ881L22.3 | 20 | Antimicrobials |
| C20orf71 | MGC44525|SPLUNC3 | 20 | Antimicrobials |
| C20orf114 | LPLUNC1|MGC14597 | 20 | Antimicrobials |
| OASL | TRIP14|p59OASL | 12 | Antimicrobials |
| CRABP2 | CRABP-II|RBP6 | 1 | Antimicrobials |
| CRABP1 | CRABP|CRABP-I|CRABPI|RBP5 | 15 | Antimicrobials |
| RBP7 | CRBP4|CRBPIV|MGC70641 | 1 | Antimicrobials |
| DUOX1 | LNOX1|MGC138840|MGC138841|NOXEF1|THOX1 | 15 | Antimicrobials |
| OBP2B | MGC119022|hOBPIIb | 9 | Antimicrobials |
| RBP2 | CRABP-II|CRBP2|CRBPII|RBPC2 | 3 | Antimicrobials |
| LCN15 | PRO6093|UNQ2541 | 9 | Antimicrobials |
| CETP | HDLCQ10 | 16 | Antimicrobials |
| FABP12 | - | 8 | Antimicrobials |
| FABP9 | PERF|PERF15|T-FABP | 8 | Antimicrobials |
| PLUNC | LPLUNC3|LUNX|NASG|SPLUNC1|SPURT|bA49G10.5 | 20 | Antimicrobials |
| LCNL1 | FLJ45224 | 9 | Antimicrobials |
| C8G | C8C|MGC142186 | 9 | Antimicrobials |
| SPAG11A | HE2 | 8 | Antimicrobials |
| PI15 | CRISP8|DKFZp686F0366|P24TI|P25TI | 8 | Antimicrobials |
| NOX1 | GP91-2|MOX1|NOH-1|NOH1 | X | Antimicrobials |
| PMP2 | FABP8|M-FABP|MP2|P2 | 8 | Antimicrobials |
| APOD | - | 3 | Antimicrobials |
| ORM2 | AGP-B|AGP-B'|AGP2 | 9 | Antimicrobials |
| ORM1 | AGP-A|AGP1|ORM | 9 | Antimicrobials |
| TNF | DIF|TNF-alpha|TNFA|TNFSF2 | 6 | Antimicrobials |
| CTSG | CG|MGC23078 | 14 | Antimicrobials |
| PRTN3 | ACPA|AGP7|C-ANCA|MBT|P29|PR-3 | 19 | Antimicrobials |
| MAPK1 | ERK|ERK2|ERT1|MAPK2|P42MAPK|PRKM1|PRKM2|p38|p40|p41|p41mapk | 22 | Antimicrobials |
| PML | MYL|PP8675|RNF71|TRIM19 | 15 | Antimicrobials |
| AEN | FLJ12484|FLJ12562|ISG20L1|pp12744 | 15 | Antimicrobials |
| CYBB | CGD|GP91-1|GP91-PHOX|GP91PHOX|NOX2|p91-PHOX | X | Antimicrobials |
| C20orf70 | PSP|SPLUNC2|bA49G10.1 | 20 | Antimicrobials |
| ISG20 | CD25|HEM45 | 15 | Antimicrobials |
| BCL3 | BCL4|D19S37 | 19 | Antimicrobials |
| ISG20L2 | FLJ12671 | 1 | Antimicrobials |
| NOX5 | MGC149776|MGC149777|NOX5A|NOX5B | 15 | Antimicrobials |
| NOX3 | GP91-3 | 6 | Antimicrobials |
| DUOX2 | LNOX2|NOXEF2|P138-TOX|THOX2 | 15 | Antimicrobials |
| TLR3 | CD283 | 4 | Antimicrobials |
| TFRC | CD71|TFR|TFR1|TRFR | 3 | Antimicrobials |
| IFIH1 | Hlcd|IDDM19|MDA-5|MDA5|MGC133047 | 2 | Antimicrobials |
| LRP1 | A2MR|APOER|APR|CD91|FLJ16451|IGFBP3R|LRP|MGC88725|TGFBR5 | 12 | Antimicrobials |
| TRIM5 | RNF88|TRIM5alpha | 11 | Antimicrobials |
| IDO1 | CD107B|IDO|INDO | 8 | Antimicrobials |
| GDF15 | GDF-15|MIC-1|MIC1|NAG-1|PDF|PLAB|PTGFB | 19 | Antimicrobials |
| NEDD4 | KIAA0093|MGC176705|NEDD4-1|RPF1 | 15 | Antimicrobials |
| ADIPOQ | ACDC|ACRP30|ADIPQTL1|ADPN|APM-1|APM1|GBP28|adiponectin | 3 | Antimicrobials |
| STAT3 | APRF|FLJ20882|HIES|MGC16063 | 17 | Antimicrobials |
| STAT1 | DKFZp686B04100|ISGF-3|STAT91 | 2 | Antimicrobials |
| IL28A | IFNL2|IL-28A | 19 | Antimicrobials |
| SOCS3 | ATOD4|CIS3|Cish3|MGC71791|SOCS-3|SSI-3|SSI3 | 17 | Antimicrobials |
| SEMG1 | MGC14719|SEMG|SGI | 20 | Antimicrobials |
| TNFSF10 | APO2L|Apo-2L|CD253|TL2|TRAIL | 3 | Antimicrobials |
| CCL20 | CKb4|LARC|MIP-3a|MIP3A|SCYA20|ST38 | 2 | Antimicrobials |
| SOCS1 | CIS1|CISH1|JAB|SOCS-1|SSI-1|SSI1|TIP3 | 16 | Antimicrobials |
| RNASEL | DKFZp781D08126|MGC104972|MGC133329|PRCA1|RNS4 | 1 | Antimicrobials |
| IRF1 | IRF-1|MAR | 5 | Antimicrobials |
| IL15 | IL-15|MGC9721 | 4 | Antimicrobials |
| APOBEC3F | ARP8|BK150C2.4.MRNA|KA6|MGC74891 | 22 | Antimicrobials |
| RARRES3 | HRASLS4|MGC8906|PLA1/2-3|RIG1|TIG3 | 11 | Antimicrobials |
| CHIT1 | CHI3|CHIT|FLJ00314|MGC125322 | 1 | Antimicrobials |
| IFNA1 | IFL|IFN|IFN-ALPHA|IFNA13|IFNA@|MGC138207|MGC138505|MGC138507 | 9 | Antimicrobials |
| CD40 | Bp50|CDW40|MGC9013|TNFRSF5|p50 | 20 | Antimicrobials |
| TLR7 | - | X | Antimicrobials |
| PPIA | CYPA|CYPH|MGC117158|MGC12404|MGC23397 | 7 | Antimicrobials |
| HFE | HFE1|HH|HLA-H|MGC103790|MVCD7|dJ221C16.10.1 | 6 | Antimicrobials |
| ZYX | ESP-2|HED-2 | 7 | Antimicrobials |
| NLRX1 | CLR11.3|DLNB26|FLJ21478|MGC131937|MGC21025|NOD26|NOD5|NOD9 | 11 | Antimicrobials |
| PGC | - | 6 | Antimicrobials |
| VEGFA | MGC70609|MVCD1|VEGF|VEGF-A|VPF | 6 | Antimicrobials |
| IKBKE | IKK-i|IKKE|IKKI|KIAA0151|MGC125294|MGC125295|MGC125297 | 1 | Antimicrobials |
| ISG15 | G1P2|IFI15|UCRP | 1 | Antimicrobials |
| DHX58 | D11LGP2|D11lgp2e|LGP2 | 17 | Antimicrobials |
| TNFAIP3 | A20|MGC104522|MGC138687|MGC138688|OTUD7C|TNFA1P2 | 6 | Antimicrobials |
| TFR2 | HFE3|MGC126368|TFRC2 | 7 | Antimicrobials |
| FCN2 | EBP-37|FCNL|P35|ficolin-2 | 9 | Antimicrobials |
| MUC4 | HSA276359 | 3 | Antimicrobials |
| F2R | CF2R|HTR|PAR1|TR | 5 | Antimicrobials |
| ELN | FLJ38671|FLJ43523|SVAS|WBS|WS | 7 | Antimicrobials |
| IL27 | IL-27|IL-27A|IL27p28|IL30|MGC71873|p28 | 16 | Antimicrobials |
| MAPT | DDPAC|FLJ31424|FTDP-17|MAPTL|MGC138549|MSTD|MTBT1|MTBT2|PPND|TAU | 17 | Antimicrobials |
| LYZ | LZM|lysozyme | 12 | Antimicrobials |
| CCL5 | D17S136E|MGC17164|RANTES|SCYA5|SISd|TCP228 | 17 | Antimicrobials |
| LEP | FLJ94114|OB|OBS | 7 | Antimicrobials |
| CYLD | CDMT|CYLD1|CYLDI|EAC|FLJ20180|FLJ31664|FLJ78684|HSPC057|KIAA0849|MFT|MFT1|SBS|TEM|USPL2 | 16 | Antimicrobials |
| KLKB1 | KLK3|PPK | 4 | Antimicrobials |
| CST4 | MGC71923 | 20 | Antimicrobials |
| CSRP1 | CRP|CRP1|CSRP|CYRP|D1S181E|DKFZp686M148 | 1 | Antimicrobials |
| MAPK14 | CSBP1|CSBP2|CSPB1|EXIP|Mxi2|PRKM14|PRKM15|RK|SAPK2A|p38|p38ALPHA | 6 | Antimicrobials |
| JUN | AP-1|AP1|c-Jun | 1 | Antimicrobials |
| ITGAV | CD51|DKFZp686A08142|MSK8|VNRA | 2 | Antimicrobials |
| IRF5 | SLEB10 | 7 | Antimicrobials |
| CCR6 | BN-1|CD196|CKR-L3|CKR6|CKRL3|CMKBR6|DCR2|DRY-6|GPR-CY4|GPR29|GPRCY4|STRL22 | 6 | Antimicrobials |
| IL12B | CLMF|CLMF2|IL-12B|NKSF|NKSF2 | 5 | Antimicrobials |
| TLR8 | CD288|MGC119599|MGC119600 | X | Antimicrobials |
| GNLY | 519|D2S69E|LAG-2|LAG2|NKG5|TLA519 | 2 | Antimicrobials |
| CD81 | S5.7|TAPA1|TSPAN28 | 11 | Antimicrobials |
| EIF2AK2 | EIF2AK1|MGC126524|PKR|PRKR | 2 | Antimicrobials |
| APOM | G3a|HSPC336|MGC22400|NG20 | 6 | Antimicrobials |
| CACYBP | GIG5|MGC87971|PNAS-107|RP1-102G20.6|S100A6BP|SIP | 1 | Antimicrobials |
| NOD1 | CARD4|CLR7.1|NLRC1 | 7 | Antimicrobials |
| MAPK8 | JNK|JNK1|JNK1A2|JNK21B1/2|PRKM8|SAPK1 | 10 | Antimicrobials |
| MAPK3 | ERK1|HS44KDAP|HUMKER1A|MGC20180|P44ERK1|P44MAPK|PRKM3 | 16 | Antimicrobials |
| BST2 | CD317 | 19 | Antimicrobials |
| BPHL | BPH-RP|MCNAA|MGC125930|MGC41865|VACVASE | 6 | Antimicrobials |
| PLA2G2A | MOM1|PLA2|PLA2B|PLA2L|PLA2S|PLAS1|sPLA2 | 1 | Antimicrobials |
| GRN | GEP|GP88|PCDGF|PEPI|PGRN | 17 | Antimicrobials |
| NEWENTRY | - | - | Antimicrobials |
| PDGFRA | CD140A|MGC74795|PDGFR2|Rhe-PDGFRA | 4 | Antimicrobials |
| GNAI1 | Gi | 7 | Antimicrobials |
| WNT5A | hWNT5A | 3 | Antimicrobials |
| FURIN | FUR|PACE|PCSK3|SPC1 | 15 | Antimicrobials |
| ADAR | ADAR1|DRADA|DSH|DSRAD|G1P1|IFI-4|IFI4|K88dsRBP|p136 | 1 | Antimicrobials |
| TYK2 | JTK1 | 19 | Antimicrobials |
| NOS2 | HEP-NOS|INOS|NOS|NOS2A | 17 | Antimicrobials |
| TRAF3 | CAP-1|CD40bp|CRAF1|LAP1 | 14 | Antimicrobials |
| TPT1 | FLJ27337|HRF|TCTP|p02 | 13 | Antimicrobials |
| TPM2 | AMCD1|DA1|DA2B|TMSB | 9 | Antimicrobials |
| NEO1 | DKFZp547A066|DKFZp547B146|HsT17534|IGDCC2|NGN | 15 | Antimicrobials |
| AHNAK | AHNAKRS|MGC5395 | 11 | Antimicrobials |
| TLR1 | CD281|DKFZp547I0610|DKFZp564I0682|KIAA0012|MGC104956|MGC126311|MGC126312|TIL|rsc786 | 4 | Antimicrobials |
| TK2 | - | 16 | Antimicrobials |
| PRDX2 | MGC4104|NKEFB|PRP|PRX2|PRXII|TDPX1|TSA | 19 | Antimicrobials |
| MX2 | MXB | 21 | Antimicrobials |
| FGF2 | BFGF|FGFB|HBGF-2 | 4 | Antimicrobials |
| FGA | Fib2|MGC119422|MGC119423|MGC119425 | 4 | Antimicrobials |
| TCF7L2 | TCF-4|TCF4 | 10 | Antimicrobials |
| F2RL1 | GPR11|PAR2 | 5 | Antimicrobials |
| DAK | DKFZp586B1621|MGC5621 | 11 | Antimicrobials |
| MSR1 | CD204|SCARA1|SR-A|phSR1|phSR2 | 8 | Antimicrobials |
| NFKBIZ | FLJ30225|FLJ34463|IKBZ|INAP|MAIL | 3 | Antimicrobials |
| LMBR1 | ACHP|C7orf2|DIF14|FLJ11665|PPD2|TPT | 7 | Antimicrobials |
| SPINLW1 | EPPIN|EPPIN1|EPPIN2|EPPIN3|WAP7|WFDC7|dJ461P17.2 | 20 | Antimicrobials |
| SRC | ASV|SRC1|c-SRC|p60-Src | 20 | Antimicrobials |
| MPO | - | 17 | Antimicrobials |
| ELAVL1 | ELAV1|HUR|Hua|MelG | 19 | Antimicrobials |
| ROBO3 | FLJ21044|HGPPS|HGPS|RBIG1|RIG1 | 11 | Antimicrobials |
| SP1 | - | 12 | Antimicrobials |
| SOD1 | ALS|ALS1|IPOA|SOD|homodimer | 21 | Antimicrobials |
| PDF | - | 16 | Antimicrobials |
| DLL4 | MGC126344|hdelta2 | 15 | Antimicrobials |
| ECD | GCR2|HSGT1 | 10 | Antimicrobials |
| SLC11A1 | LSH|NRAMP|NRAMP1 | 2 | Antimicrobials |
| DMBT1 | GP340|MGC164738|muclin | 10 | Antimicrobials |
| TMEM173 | FLJ38577|MITA|MPYS|STING | 5 | Antimicrobials |
| SKIV2L | 170A|DDX13|HLP|SKI2|SKI2W|SKIV2 | 6 | Antimicrobials |
| SEMG2 | SGII | 20 | Antimicrobials |
| LTA | LT|TNFB|TNFSF1 | 6 | Antimicrobials |
| DES | CMD1I|CSM1|CSM2|FLJ12025|FLJ39719|FLJ41013|FLJ41793 | 2 | Antimicrobials |
| DCK | MGC117410|MGC138632 | 4 | Antimicrobials |
| DAXX | BING2|DAP6|EAP1|MGC126245|MGC126246 | 6 | Antimicrobials |
| TNFRSF10A | APO2|CD261|DR4|MGC9365|TRAILR-1|TRAILR1 | 8 | Antimicrobials |
| TNFRSF10B | CD262|DR5|KILLER|KILLER/DR5|TRAIL-R2|TRAILR2|TRICK2|TRICK2A|TRICK2B|TRICKB|ZTNFR9 | 8 | Antimicrobials |
| EED | HEED|WAIT1 | 11 | Antimicrobials |
| CCL4 | ACT2|AT744.1|G-26|LAG1|MGC104418|MGC126025|MGC126026|MIP-1-beta|MIP1B|MIP1B1|SCYA2|SCYA4 | 17 | Antimicrobials |
| LIMS1 | PINCH|PINCH1 | 2 | Antimicrobials |
| LALBA | MGC138521|MGC138523 | 12 | Antimicrobials |
| APOBEC3H | ARP10|dJ742C19.2 | 22 | Antimicrobials |
| TMPRSS6 | IRIDA | 22 | Antimicrobials |
| SPINK5 | DKFZp686K19184|FLJ21544|FLJ97536|FLJ97596|FLJ99794|LEKTI|LETKI|NETS|NS|VAKTI | 5 | Antimicrobials |
| MARCO | SCARA2 | 2 | Antimicrobials |
| BECN1 | ATG6|VPS30|beclin1 | 17 | Antimicrobials |
| TNFSF11 | CD254|ODF|OPGL|OPTB2|RANKL|TRANCE|hRANKL2|sOdf | 13 | Antimicrobials |
| KNG1 | BDK|KNG | 3 | Antimicrobials |
| CSK | MGC117393 | 15 | Antimicrobials |
| KLRK1 | CD314|D12S2489E|FLJ17759|FLJ75772|KLR|NKG2-D|NKG2D | 12 | Antimicrobials |
| KCNH2 | ERG1|HERG|HERG1|Kv11.1|LQT2|SQT1 | 7 | Antimicrobials |
| JUND | AP-1 | 19 | Antimicrobials |
| JAK1 | JAK1A|JAK1B|JTK3 | 1 | Antimicrobials |
| CREB1 | CREB|MGC9284 | 2 | Antimicrobials |
| CLDN4 | CPE-R|CPER|CPETR|CPETR1|WBSCR8|hCPE-R | 7 | Antimicrobials |
| CCL28 | CCK1|MEC|MGC71902|SCYA28 | 5 | Antimicrobials |
| RNASE3 | ECP|RNS3 | 14 | Antimicrobials |
| RN7SL1 | 7L1a|7SL|RN7SL|RNSRP1 | 14 | Antimicrobials |
| IRF7 | IRF-7H|IRF7A | 11 | Antimicrobials |
| IREB2 | ACO3|FLJ23381|IRP2|IRP2AD | 15 | Antimicrobials |
| ILK | DKFZp686F1765|P59 | 11 | Antimicrobials |
| IL18 | IGIF|IL-18|IL-1g|IL1F4|MGC12320 | 11 | Antimicrobials |
| IL17A | CTLA8|IL-17|IL-17A|IL17 | 6 | Antimicrobials |
| LTB4R | BLT1|BLTR|CMKRL1|GPR16|LTB4R1|LTBR1|P2RY7|P2Y7 | 14 | Antimicrobials |
| APOBEC3A | ARP3|PHRBN|bK150C2.1 | 22 | Antimicrobials |
| MASP2 | MAP19|MASP-2|sMAP | 1 | Antimicrobials |
| TRIM27 | RFP|RNF76 | 6 | Antimicrobials |
| RELA | MGC131774|NFKB3|p65 | 11 | Antimicrobials |
| IL7R | CD127|CDW127|IL-7R-alpha|IL7RA|ILRA | 5 | Antimicrobials |
| IL1A | IL-1A|IL1|IL1-ALPHA|IL1F1 | 2 | Antimicrobials |
| PTX3 | TNFAIP5|TSG-14 | 3 | Antimicrobials |
| IFNAR2 | IFN-R|IFN-alpha-REC|IFNABR|IFNARB | 21 | Antimicrobials |
| IFN1@ | IFNA | 9 | Antimicrobials |
| SYTL1 | FLJ14996|JFC1|SLP1 | 1 | Antimicrobials |
| APOBEC3C | APOBEC1L|ARDC2|ARDC4|ARP5|MGC19485|PBI|bK150C2.3 | 22 | Antimicrobials |
| DDX17 | DKFZp761H2016|P72|RH70 | 22 | Antimicrobials |
| PTGS2 | COX-2|COX2|GRIPGHS|PGG/HS|PGHS-2|PHS-2|hCox-2 | 1 | Antimicrobials |
| HTR1A | 5-HT1A|5HT1a|ADRB2RL1|ADRBRL1 | 5 | Antimicrobials |
| SEPT-7 | CDC10|CDC3|Nbla02942|SEPT7A | 7 | Antimicrobials |
| CD40LG | CD154|CD40L|HIGM1|IGM|IMD3|T-BAM|TNFSF5|TRAP|gp39|hCD40L | X | Antimicrobials |
| CD14 | - | 5 | Antimicrobials |
| CD8A | CD8|Leu2|MAL|p32 | 2 | Antimicrobials |
| CD4 | CD4mut | 12 | Antimicrobials |
| MASP1 | CRARF|CRARF1|DKFZp686I01199|FLJ26383|MASP|MGC126283|MGC126284|PRSS5|RaRF | 3 | Antimicrobials |
| PROC | PC|PROC1 | 2 | Antimicrobials |
| MAP2K2 | FLJ26075|MAPKK2|MEK2|MKK2|PRKMK2 | 19 | Antimicrobials |
| MAP2K1 | MAPKK1|MEK1|MKK1|PRKMK1 | 15 | Antimicrobials |
| HRG | DKFZp779H1622|HPRG|HRGP | 3 | Antimicrobials |
| NDRG1 | CAP43|CMT4D|DRG1|GC4|HMSNL|NDR1|NMSL|PROXY1|RIT42|RTP|TARG1|TDD5 | 8 | Antimicrobials |
| IRF9 | IRF-9|ISGF3|ISGF3G|p48 | 14 | Antimicrobials |
| TRIM22 | GPSTAF50|RNF94|STAF50 | 11 | Antimicrobials |
| LANCL1 | GPR69A|p40 | 2 | Antimicrobials |
| PPP4C | PP4|PPH3|PPX | 16 | Antimicrobials |
| HMOX1 | HO-1|HSP32|bK286B10 | 22 | Antimicrobials |
| HMGB1 | DKFZp686A04236|HMG1|HMG3|SBP-1 | 13 | Antimicrobials |
| HLA-B | AS|HLA-B-7301|HLA-B73|HLAB|HLAC|MGC111087|SPDA1 | 6 | Antimicrobials |
| RNASE7 | MGC133220 | 14 | Antimicrobials |
| ABCC4 | EST170205|MOAT-B|MOATB|MRP4 | 13 | Antimicrobials |
| HGF | F-TCF|HGFB|HPTA|SF | 7 | Antimicrobials |
| HDAC1 | DKFZp686H12203|GON-10|HD1|RPD3|RPD3L1 | 1 | Antimicrobials |
| IL28RA | CRF2/12|IFNLR|IFNLR1|IL-28R1|LICR2 | 1 | Antimicrobials |
| PLSCR1 | MMTRA1B | 3 | Antimicrobials |
| B2M | - | 15 | Antimicrobials |
| BACH2 | - | 6 | Antimicrobials |
| TANK | I-TRAF|TRAF2 | 2 | Antimicrobials |
| PIK3CG | PI3CG|PI3K|PI3Kgamma|PIK3 | 7 | Antimicrobials |
| ARRB1 | ARB1|ARR1 | 11 | Antimicrobials |
| RSAD2 | 2510004L01Rik|cig33|cig5|vig1 | 2 | Antimicrobials |
| STAB2 | DKFZp434E0321|FEEL-2|FELE-2|FELL|FELL-2|FEX2|HARE|STAB-2 | 12 | Antimicrobials |
| TBK1 | FLJ11330|NAK|T2K | 12 | Antimicrobials |
| PDYN | MGC26418|PENKB | 20 | Antimicrobials |
| PDGFRB | CD140B|JTK12|PDGF-R-beta|PDGFR|PDGFR1 | 5 | Antimicrobials |
| PDCD1 | CD279|PD1|SLEB2|hPD-1|hPD-l | 2 | Antimicrobials |
| PCSK2 | NEC2|PC2|SPC2 | 20 | Antimicrobials |
| PCSK1 | BMIQ12|NEC1|PC1|PC3|SPC3 | 5 | Antimicrobials |
| ARG2 | - | 14 | Antimicrobials |
| AQP9 | HsT17287|SSC1 | 15 | Antimicrobials |
| FASLG | APT1LG1|CD178|CD95L|FASL|TNFSF6 | 1 | Antimicrobials |
| APOH | B2G1|BG | 17 | Antimicrobials |
| BIRC5 | API4|EPR-1 | 17 | Antimicrobials |
| ANXA6 | ANX6|CBP68 | 5 | Antimicrobials |
| IL22 | IL-21|IL-22|IL-D110|IL-TIF|IL21|ILTIF|MGC79382|MGC79384|TIFIL-23|TIFa|zcyto18 | 12 | Antimicrobials |
| VTN | V75|VN|VNT | 17 | Antimicrobials |
| VIM | FLJ36605 | 10 | Antimicrobials |
| VCAM1 | CD106|DKFZp779G2333|INCAM-100|MGC99561 | 1 | Antimicrobials |
| PRDX1 | MSP23|NKEFA|PAG|PAGA|PAGB|PRX1|PRXI|TDPX2 | 1 | Antimicrobials |
| GFAP | FLJ45472 | 17 | Antimicrobials |
| GBP2 | - | 1 | Antimicrobials |
| ALB | DKFZp779N1935|PRO0883|PRO0903|PRO1341 | 4 | Antimicrobials |
| SLC29A3 | ENT3|FLJ11160 | 10 | Antimicrobials |
| OAS1 | IFI-4|OIAS|OIASI | 12 | Antimicrobials |
| AGER | MGC22357|RAGE | 6 | Antimicrobials |
| UNC93B1 | MGC126617|UNC93|UNC93B | 11 | Antimicrobials |
| TNFSF4 | CD134L|CD252|GP34|OX-40L|OX4OL|TXGP1 | 1 | Antimicrobials |
| NOS1 | IHPS1|NOS|nNOS | 12 | Antimicrobials |
| ACTG1 | ACT|ACTG|DFNA20|DFNA26 | 17 | Antimicrobials |
| ACTA1 | ACTA|ASMA|CFTD|CFTD1|CFTDM|MPFD|NEM1|NEM2|NEM3 | 1 | Antimicrobials |
| ACO1 | ACONS|IREB1|IREBP|IREBP1|IRP1 | 9 | Antimicrobials |
| SERPINA3 | AACT|ACT|GIG24|GIG25|MGC88254 | 14 | Antimicrobials |
| IL8RA | C-C|C-C-CKR-1|CD128|CD181|CDw128a|CKR-1|CMKAR1|CXCR1|IL8R1|IL8RBA | 2 | Antimicrobials |
| CCL15 | HCC-2|HMRP-2B|LKN1|Lkn-1|MIP-1d|MIP-5|NCC-3|NCC3|SCYA15|SCYL3|SY15 | 17 | Antimicrobials |
| CCL14 | CC-1|CC-3|CKb1|FLJ16015|HCC-1|HCC-3|MCIF|NCC-2|NCC2|SCYA14|SCYL2|SY14 | 17 | Antimicrobials |
| CCL4 | ACT2|AT744.1|G-26|LAG1|MGC104418|MGC126025|MGC126026|MIP-1-beta|MIP1B|MIP1B1|SCYA2|SCYA4 | 17 | Antimicrobials |
| CCL16 | CKb12|HCC-4|ILINCK|LCC-1|LEC|LMC|MGC117051|Mtn-1|NCC-4|NCC4|SCYA16|SCYL4 | 17 | Antimicrobials |
| CCL19 | CKb11|ELC|MGC34433|MIP-3b|MIP3B|SCYA19 | 9 | Antimicrobials |
| CCL13 | CKb10|MCP-4|MGC17134|NCC-1|NCC1|SCYA13|SCYL1 | 17 | Antimicrobials |
| CCL18 | AMAC-1|AMAC1|CKb7|DC-CK1|DCCK1|MIP-4|PARC|SCYA18 | 17 | Antimicrobials |
| CCL17 | A-152E5.3|ABCD-2|MGC138271|MGC138273|SCYA17|TARC | 16 | Antimicrobials |
| CCL26 | IMAC|MGC126714|MIP-4a|MIP-4alpha|SCYA26|TSC-1 | 7 | Antimicrobials |
| CCL22 | A-152E5.1|ABCD-1|DC/B-CK|MDC|MGC34554|SCYA22|STCP-1 | 16 | Antimicrobials |
| CCR3 | CC-CKR-3|CD193|CKR3|CMKBR3|MGC102841 | 3 | Antimicrobials |
| CCL28 | CCK1|MEC|MGC71902|SCYA28 | 5 | Antimicrobials |
| CCL4L2 | AT744.2|CCL4L|SCYA4L | 17 | Antimicrobials |
| CCBP2 | CCR10|CCR9|CMKBR9|D6|MGC126678|MGC138250|hD6 | 3 | Antimicrobials |
| CCR7 | BLR2|CD197|CDw197|CMKBR7|EBI1 | 17 | Antimicrobials |
| CCL27 | ALP|CTACK|CTAK|ESKINE|ILC|PESKY|SCYA27 | 9 | Antimicrobials |
| CCR8 | CDw198|CKR-L1|CKRL1|CMKBR8|CMKBRL2|CY6|GPR-CY6|MGC129966|MGC129973|TER1 | 3 | Antimicrobials |
| CCRL1 | CC-CKR-11|CCBP2|CCR10|CCR11|CCX-CKR|CKR-11|PPR1|VSHK1 | 3 | Antimicrobials |
| CCR10 | GPR2 | 17 | Antimicrobials |
| CCL2 | GDCF-2|HC11|HSMCR30|MCAF|MCP-1|MCP1|MGC9434|SCYA2|SMC-CF | 17 | Antimicrobials |
| CCL21 | 6Ckine|CKb9|ECL|MGC34555|SCYA21|SLC|TCA4 | 9 | Antimicrobials |
| CCL7 | FIC|MARC|MCP-3|MCP3|MGC138463|MGC138465|NC28|SCYA6|SCYA7 | 17 | Antimicrobials |
| CCL5 | D17S136E|MGC17164|RANTES|SCYA5|SISd|TCP228 | 17 | Antimicrobials |
| CCL3 | G0S19-1|LD78ALPHA|MIP-1-alpha|MIP1A|SCYA3 | 17 | Antimicrobials |
| CCL20 | CKb4|LARC|MIP-3a|MIP3A|SCYA20|ST38 | 2 | Antimicrobials |
| CCL11 | MGC22554|SCYA11 | 17 | Antimicrobials |
| CCR5 | CC-CKR-5|CCCKR5|CD195|CKR-5|CKR5|CMKBR5|FLJ78003|IDDM22 | 3 | Antimicrobials |
| CCL23 | CK-BETA-8|CKb8|Ckb-8|Ckb-8-1|MIP-3|MIP3|MPIF-1|SCYA23 | 17 | Antimicrobials |
| CCL25 | Ckb15|MGC150327|SCYA25|TECK | 19 | Antimicrobials |
| CCL1 | I-309|P500|SCYA1|SISe|TCA3 | 17 | Antimicrobials |
| CCL3L3 | 464.2|D17S1718|LD78|LD78BETA|MGC12815|SCYA3L|SCYA3L1 | 17 | Antimicrobials |
| CCL4L1 | AT744.2|CCL4L|LAG-1|LAG1|SCYA4L | 17 | Antimicrobials |
| CXCL12 | PBSF|SCYB12|SDF-1a|SDF-1b|SDF1|SDF1A|SDF1B|TLSF-a|TLSF-b|TPAR1 | 10 | Antimicrobials |
| XCL1 | ATAC|LPTN|LTN|SCM-1|SCM-1a|SCM1|SCYC1 | 1 | Antimicrobials |
| CCL8 | HC14|MCP-2|MCP2|SCYA10|SCYA8 | 17 | Antimicrobials |
| CCL3L1 | 464.2|D17S1718|G0S19-2|LD78|LD78BETA|MGC104178|MGC12815|MGC182017|MIP1AP|SCYA3L|SCYA3L1 | 17 | Antimicrobials |
| CCR1 | CD191|CKR-1|CKR1|CMKBR1|HM145|MIP1aR|SCYAR1 | 3 | Antimicrobials |
| CCL24 | Ckb-6|MPIF-2|MPIF2|SCYA24 | 7 | Antimicrobials |
| XCL2 | SCM-1b|SCM1B|SCYC2 | 1 | Antimicrobials |
| CXCL1 | FSP|GRO1|GROa|MGSA|MGSA-a|NAP-3|SCYB1 | 4 | Antimicrobials |
| CXCL10 | C7|IFI10|INP10|IP-10|SCYB10|crg-2|gIP-10|mob-1 | 4 | Antimicrobials |
| CXCR4 | CD184|D2S201E|FB22|HM89|HSY3RR|LAP3|LCR1|LESTR|NPY3R|NPYR|NPYRL|NPYY3R|WHIM | 2 | Antimicrobials |
| CXCL2 | CINC-2a|GRO2|GROb|MGSA-b|MIP-2a|MIP2|MIP2A|SCYB2 | 4 | Antimicrobials |
| CXCR6 | BONZO|CD186|STRL33|TYMSTR | 3 | Antimicrobials |
| CCR4 | CC-CKR-4|CD194|CKR4|CMKBR4|ChemR13|HGCN:14099|K5-5|MGC88293 | 3 | Antimicrobials |
| CXCL11 | H174|I-TAC|IP-9|IP9|MGC102770|SCYB11|SCYB9B|b-R1 | 4 | Antimicrobials |
| FAM19A5 | QLLK5208|TAFA-5|TAFA5|UNQ5208 | 22 | Antimicrobials |
| FAM19A3 | MGC138473|TAFA-3|TAFA3 | 1 | Antimicrobials |
| FAM19A4 | FLJ25161|TAFA-4|TAFA4 | 3 | Antimicrobials |
| FAM19A1 | TAFA-1|TAFA1 | 3 | Antimicrobials |
| FAM19A2 | DKFZp761E1217|DKFZp781P0552|MGC42403|TAFA-2|TAFA2 | 12 | Antimicrobials |
| CCL14-CCL15 | - | 17 | Antimicrobials |
| IL6 | BSF2|HGF|HSF|IFNB2|IL-6 | 7 | Antimicrobials |
| TNF | DIF|TNF-alpha|TNFA|TNFSF2 | 6 | Antimicrobials |
| IL1B | IL-1|IL1-BETA|IL1F2 | 2 | Antimicrobials |
| IL18 | IGIF|IL-18|IL-1g|IL1F4|MGC12320 | 11 | Antimicrobials |
| PTK2B | CADTK|CAKB|FADK2|FAK2|FRNK|PKB|PTK|PYK2|RAFTK | 8 | Antimicrobials |
| VEGFA | MGC70609|MVCD1|VEGF|VEGF-A|VPF | 6 | Antimicrobials |
| IL4 | BCGF-1|BCGF1|BSF1|IL-4|MGC79402 | 5 | Antimicrobials |
| CDH1 | Arc-1|CD324|CDHE|ECAD|LCAM|UVO | 16 | Antimicrobials |
| CD40 | Bp50|CDW40|MGC9013|TNFRSF5|p50 | 20 | Antimicrobials |
| DEFB103A | DEFB103|DEFB3|HBD-3|HBD3|HBP-3|HBP3 | 8 | Antimicrobials |
| F2RL1 | GPR11|PAR2 | 5 | Antimicrobials |
| MMP9 | CLG4B|GELB|MMP-9 | 20 | Antimicrobials |
| LTBP1 | MGC163161 | 2 | Antimicrobials |
| DEFB4 | DEFB-2|DEFB102|DEFB2|HBD-2|SAP1 | 8 | Antimicrobials |
| TNFSF10 | APO2L|Apo-2L|CD253|TL2|TRAIL | 3 | Antimicrobials |
| IL13 | ALRH|BHR1|IL-13|MGC116786|MGC116788|MGC116789|P600 | 5 | Antimicrobials |
| IL10 | CSIF|IL-10|IL10A|MGC126450|MGC126451|TGIF | 1 | Antimicrobials |
| IL2 | IL-2|TCGF|lymphokine | 4 | Antimicrobials |
| PPARG | CIMT1|NR1C3|PPARG1|PPARG2|PPARgamma | 3 | Antimicrobials |
| FGR | FLJ43153|MGC75096|SRC2|c-fgr|c-src2|p55c-fgr|p58c-fgr | 1 | Antimicrobials |
| MIF | GIF|GLIF|MMIF | 22 | Antimicrobials |
| CRP | MGC149895|MGC88244|PTX1 | 1 | Antimicrobials |
| JAK2 | JTK10 | 9 | Antimicrobials |
| IL1A | IL-1A|IL1|IL1-ALPHA|IL1F1 | 2 | Antimicrobials |
| PTK2 | FADK|FAK|FAK1|pp125FAK | 8 | Antimicrobials |
| PTGDR | AS1|ASRT1|DP|DP1|MGC49004 | 14 | Antimicrobials |
| CD86 | B7-2|B70|CD28LG2|LAB72|MGC34413 | 3 | Antimicrobials |
| HCK | JTK9 | 20 | Antimicrobials |
| ARRB1 | ARB1|ARR1 | 11 | Antimicrobials |
| GNAI1 | Gi | 7 | Antimicrobials |
| VDR | NR1I1 | 12 | Antimicrobials |
| OLR1 | CLEC8A|LOX1|SCARE1 | 12 | Antimicrobials |
| ADRBK1 | BARK1|BETA-ARK1|FLJ16718|GRK2 | 11 | Antimicrobials |
| TXK | BTKL|MGC22473|PSCTK5|PTK4|RLK|TKL | 4 | Antimicrobials |
| RNASE2 | EDN|RNS2 | 14 | Antimicrobials |
| CD79A | IGA|MB-1 | 19 | BCRSignalingPathway |
| CD79B | B29|IGB | 17 | BCRSignalingPathway |
| LYN | FLJ26625|JTK8 | 8 | BCRSignalingPathway |
| SYK | DKFZp313N1010|FLJ25043|FLJ37489 | 9 | BCRSignalingPathway |
| BTK | AGMX1|AT|ATK|BPK|IMD1|MGC126261|MGC126262|PSCTK1|XLA | X | BCRSignalingPathway |
| BLNK | BASH|BLNK-S|LY57|MGC111051|SLP-65|SLP65 | 10 | BCRSignalingPathway |
| VAV3 | FLJ40431 | 1 | BCRSignalingPathway |
| VAV1 | VAV | 19 | BCRSignalingPathway |
| VAV2 | - | 9 | BCRSignalingPathway |
| RAC1 | MGC111543|MIG5|TC-25|p21-Rac1 | 7 | BCRSignalingPathway |
| RAC2 | EN-7|Gx|HSPC022 | 22 | BCRSignalingPathway |
| RAC3 | - | 17 | BCRSignalingPathway |
| PPP3CA | CALN|CALNA|CALNA1|CCN1|CNA1|PPP2B | 4 | BCRSignalingPathway |
| PPP3CB | CALNA2|CALNB | 10 | BCRSignalingPathway |
| PPP3CC | CALNA3 | 8 | BCRSignalingPathway |
| CHP | SLC9A1BP | 15 | BCRSignalingPathway |
| PPP3R1 | CALNB1|CNB|CNB1 | 2 | BCRSignalingPathway |
| PPP3R2 | PPP3RL | 9 | BCRSignalingPathway |
| CHP2 | - | 16 | BCRSignalingPathway |
| NFAT5 | KIAA0827|NF-AT5|NFATL1|NFATZ|OREBP|TONEBP | 16 | BCRSignalingPathway |
| NFATC1 | MGC138448|NF-ATC|NFAT2|NFATc | 18 | BCRSignalingPathway |
| NFATC2 | NFAT1|NFATP | 20 | BCRSignalingPathway |
| NFATC3 | NFAT4|NFATX | 16 | BCRSignalingPathway |
| NFATC4 | NF-ATc4|NFAT3 | 14 | BCRSignalingPathway |
| HRAS | C-BAS/HAS|C-H-RAS|C-HA-RAS1|CTLO|H-RASIDX|HAMSV|HRAS1|K-RAS|N-RAS|RASH1 | 11 | BCRSignalingPathway |
| KRAS | C-K-RAS|K-RAS2A|K-RAS2B|K-RAS4A|K-RAS4B|KI-RAS|KRAS1|KRAS2|NS3|RASK2 | 12 | BCRSignalingPathway |
| NRAS | ALPS4|N-ras|NRAS1 | 1 | BCRSignalingPathway |
| FOS | AP-1|C-FOS | 14 | BCRSignalingPathway |
| JUN | AP-1|AP1|c-Jun | 1 | BCRSignalingPathway |
| CARD11 | BIMP3|CARMA1|MGC133069 | 7 | BCRSignalingPathway |
| BCL10 | CARMEN|CIPER|CLAP|c-E10|mE10 | 1 | BCRSignalingPathway |
| MALT1 | DKFZp434L132|MLT|MLT1 | 18 | BCRSignalingPathway |
| CHUK | IKBKA|IKK-alpha|IKK1|IKKA|NFKBIKA|TCF16 | 10 | BCRSignalingPathway |
| IKBKB | FLJ40509|IKK-beta|IKK2|IKKB|MGC131801|NFKBIKB | 8 | BCRSignalingPathway |
| IKBKG | AMCBX1|FIP-3|FIP3|Fip3p|IKK-gamma|IP|IP1|IP2|IPD2|NEMO | X | BCRSignalingPathway |
| NFKB1 | DKFZp686C01211|EBP-1|KBF1|MGC54151|NF-kappa-B|NFKB-p105|NFKB-p50|p105|p50 | 4 | BCRSignalingPathway |
| RELA | MGC131774|NFKB3|p65 | 11 | BCRSignalingPathway |
| NFKBIA | IKBA|MAD-3|NFKBI | 14 | BCRSignalingPathway |
| NFKBIB | IKBB|TRIP9 | 19 | BCRSignalingPathway |
| NFKBIE | IKBE | 6 | BCRSignalingPathway |
| CD81 | S5.7|TAPA1|TSPAN28 | 11 | BCRSignalingPathway |
| CD19 | B4|MGC12802 | 16 | BCRSignalingPathway |
| CR2 | C3DR|CD21|SLEB9 | 1 | BCRSignalingPathway |
| PIK3R5 | F730038I15Rik|FOAP-2|P101-PI3K|p101 | 17 | BCRSignalingPathway |
| PIK3R1 | GRB1|p85|p85-ALPHA | 5 | BCRSignalingPathway |
| PIK3R2 | P85B|p85|p85-BETA | 19 | BCRSignalingPathway |
| PIK3R3 | DKFZp686P05226|FLJ41892|p55|p55-GAMMA | 1 | BCRSignalingPathway |
| PIK3CA | MGC142161|MGC142163|PI3K|p110-alpha | 3 | BCRSignalingPathway |
| PIK3CB | DKFZp779K1237|MGC133043|PI3K|PI3KCB|PI3Kbeta|PIK3C1|p110-BETA | 3 | BCRSignalingPathway |
| PIK3CD | p110D | 1 | BCRSignalingPathway |
| PIK3CG | PI3CG|PI3K|PI3Kgamma|PIK3 | 7 | BCRSignalingPathway |
| AKT3 | DKFZp434N0250|PKB-GAMMA|PKBG|PRKBG|RAC-PK-gamma|RAC-gamma|STK-2 | 1 | BCRSignalingPathway |
| AKT1 | AKT|MGC99656|PKB|PKB-ALPHA|PRKBA|RAC|RAC-ALPHA | 14 | BCRSignalingPathway |
| AKT2 | PKBB|PKBBETA|PRKBB|RAC-BETA | 19 | BCRSignalingPathway |
| GSK3B | - | 3 | BCRSignalingPathway |
| INPP5D | MGC104855|MGC142140|MGC142142|SHIP|SHIP1|SIP-145|hp51CN | 2 | BCRSignalingPathway |
| CD22 | FLJ22814|MGC130020|SIGLEC-2|SIGLEC2 | 19 | BCRSignalingPathway |
| CD72 | CD72b|LYB2 | 9 | BCRSignalingPathway |
| PTPN6 | HCP|HCPH|HPTP1C|PTP-1C|SH-PTP1|SHP-1|SHP-1L|SHP1 | 12 | BCRSignalingPathway |
| LILRB3 | CD85A|HL9|ILT5|LIR-3|LIR3|MGC138403|PIRB | 19 | BCRSignalingPathway |
| FCGR2B | CD32|CD32B|FCG2|FCGR2|IGFR2 | 1 | BCRSignalingPathway |
| RASGRP3 | GRP3|KIAA0846 | 2 | BCRSignalingPathway |
| PLCG2 | - | 16 | BCRSignalingPathway |
| PRKCB | MGC41878|PKC-beta|PKCB|PRKCB1|PRKCB2 | 16 | BCRSignalingPathway |
| IFITM1 | 9-27|CD225|IFI17|LEU13 | 11 | BCRSignalingPathway |
| IGH@ | DKFZp686C15213|IGH|IGH.1@|IGHDY1|MGC72071|MGC88774 | 14 | BCRSignalingPathway |
| IGHA1 | FLJ14473|FLJ35065|FLJ35500|FLJ36402|FLJ39698|FLJ40001|FLJ41548|FLJ41552|FLJ41789|FLJ43248|FLJ43594|FLJ44293|FLJ46028|FLJ46621|FLJ46724|FLJ46811|FLJ46824|FLJ90170|IgA1|MGC102857 | 14 | BCRSignalingPathway |
| IGHA2 | - | 14 | BCRSignalingPathway |
| IGHD | FLJ00382|FLJ46727|MGC29633 | 14 | BCRSignalingPathway |
| IGHD@ | IGD1|IGHDY1 | 14 | BCRSignalingPathway |
| IGHD1-1 | IGHD11 | 14 | BCRSignalingPathway |
| IGHD1-14 | DM2|IGHD114 | 14 | BCRSignalingPathway |
| IGHD1-20 | IGHD120 | 14 | BCRSignalingPathway |
| IGHD1-26 | IGHD126 | 14 | BCRSignalingPathway |
| IGHD1-7 | DM1|IGHD17 | 14 | BCRSignalingPathway |
| IGHD2-15 | D2|IGHD215 | 14 | BCRSignalingPathway |
| IGHD2-2 | IGHD22 | 14 | BCRSignalingPathway |
| IGHD2-21 | IGHD221 | 14 | BCRSignalingPathway |
| IGHD2-8 | DLR1|IGHD28 | 14 | BCRSignalingPathway |
| IGHD3-10 | DXP'1|IGHD310 | 14 | BCRSignalingPathway |
| IGHD3-16 | IGHD316 | 14 | BCRSignalingPathway |
| IGHD3-22 | IGHD322 | 14 | BCRSignalingPathway |
| IGHD3-3 | DXP4|IGHD33 | 14 | BCRSignalingPathway |
| IGHD3-9 | DXP1|IGHD39 | 14 | BCRSignalingPathway |
| IGHD4-11 | DA1|IGHD411 | 14 | BCRSignalingPathway |
| IGHD4-17 | IGHD417 | 14 | BCRSignalingPathway |
| IGHD4-23 | IGHD423 | 14 | BCRSignalingPathway |
| IGHD4-4 | DA4|IGHD44 | 14 | BCRSignalingPathway |
| IGHD5-12 | DK1|IGHD512 | 14 | BCRSignalingPathway |
| IGHD5-18 | IGHD518 | 14 | BCRSignalingPathway |
| IGHD5-24 | IGHD524 | 14 | BCRSignalingPathway |
| IGHD5-5 | DK4|IGHD55 | 14 | BCRSignalingPathway |
| IGHD6-13 | DN1|IGHD613 | 14 | BCRSignalingPathway |
| IGHD6-19 | IGHD619 | 14 | BCRSignalingPathway |
| IGHD6-25 | IGHD625 | 14 | BCRSignalingPathway |
| IGHD6-6 | D(N4)|IGHD66 | 14 | BCRSignalingPathway |
| IGHD7-27 | DHQ52|IGHD727 | 14 | BCRSignalingPathway |
| IGHE | IgE | 14 | BCRSignalingPathway |
| IGHG1 | - | 14 | BCRSignalingPathway |
| IGHG2 | DKFZp686I04196 | 14 | BCRSignalingPathway |
| IGHG3 | DKFZp686H11213|FLJ39988|FLJ40036|FLJ40253|FLJ40587|FLJ40789|FLJ40834|IgG3|MGC45809 | 14 | BCRSignalingPathway |
| IGHG4 | MGC117419 | 14 | BCRSignalingPathway |
| IGHJ@ | IGHJ | 14 | BCRSignalingPathway |
| IGHJ1 | JH1 | 14 | BCRSignalingPathway |
| IGHJ2 | JH2 | 14 | BCRSignalingPathway |
| IGHJ3 | JH3b | 14 | BCRSignalingPathway |
| IGHJ4 | JH4b | 14 | BCRSignalingPathway |
| IGHJ5 | JH5b | 14 | BCRSignalingPathway |
| IGHJ6 | JH6b | 14 | BCRSignalingPathway |
| IGHM | DKFZp686I15196|DKFZp686I15212|FLJ00385|MGC104996|MGC52291|MU|VH | 14 | BCRSignalingPathway |
| IGHV@ | IGHV | 14 | BCRSignalingPathway |
| IGHV1-18 | IGHV118 | 14 | BCRSignalingPathway |
| IGHV1-2 | IGHV12 | 14 | BCRSignalingPathway |
| IGHV1-24 | IGHV124|VH | 14 | BCRSignalingPathway |
| IGHV1-3 | IGHV13 | 14 | BCRSignalingPathway |
| IGHV1-45 | IGHV145|VH | 14 | BCRSignalingPathway |
| IGHV1-46 | IGHV146 | 14 | BCRSignalingPathway |
| IGHV1-58 | IGHV158|VH | 14 | BCRSignalingPathway |
| IGHV1-69 | IGHV1-E|IGHV169|IGHV1E | 14 | BCRSignalingPathway |
| IGHV1-8 | IGHV18 | 14 | BCRSignalingPathway |
| IGHV1-C | IGHV1C | 14 | BCRSignalingPathway |
| IGHV1-F | IGHV1F | 14 | BCRSignalingPathway |
| IGHV2-26 | IGHV226|VH | 14 | BCRSignalingPathway |
| IGHV2-5 | IGHV25|VH | 14 | BCRSignalingPathway |
| IGHV2-70 | IGHV270|VH | 14 | BCRSignalingPathway |
| IGHV3-11 | IGHV311|VH | 14 | BCRSignalingPathway |
| IGHV3-13 | IGHV313 | 14 | BCRSignalingPathway |
| IGHV3-15 | IGHV315|VH | 14 | BCRSignalingPathway |
| IGHV3-16 | IGHV316|VH | 14 | BCRSignalingPathway |
| IGHV3-20 | IGHV320|VH | 14 | BCRSignalingPathway |
| IGHV3-21 | IGHV321|VH | 14 | BCRSignalingPathway |
| IGHV3-23 | DP47|IGHV323|V3-23|VH26 | 14 | BCRSignalingPathway |
| IGHV3-30 | IGHV330|VH | 14 | BCRSignalingPathway |
| IGHV3-30-3 | IGHV3-3|IGHV3303 | 14 | BCRSignalingPathway |
| IGHV3-30-5 | IGHV3-3|IGHV3305 | 14 | BCRSignalingPathway |
| IGHV3-33 | IGHV333|VH | 14 | BCRSignalingPathway |
| IGHV3-35 | IGHV335|VH | 14 | BCRSignalingPathway |
| IGHV3-38 | IGHV338|VH | 14 | BCRSignalingPathway |
| IGHV3-43 | IGHV343|VH | 14 | BCRSignalingPathway |
| IGHV3-48 | IGHV348|VH | 14 | BCRSignalingPathway |
| IGHV3-49 | IGHV349|VH | 14 | BCRSignalingPathway |
| IGHV3-53 | IGHV353|VH | 14 | BCRSignalingPathway |
| IGHV3-64 | IGHV364|VH | 14 | BCRSignalingPathway |
| IGHV3-66 | IGHV366|VH | 14 | BCRSignalingPathway |
| IGHV3-7 | IGHV37|VH | 14 | BCRSignalingPathway |
| IGHV3-72 | IGHV372|VH | 14 | BCRSignalingPathway |
| IGHV3-73 | IGHV373|VH | 14 | BCRSignalingPathway |
| IGHV3-74 | IGHV374|VH | 14 | BCRSignalingPathway |
| IGHV3-9 | IGHV39|VH | 14 | BCRSignalingPathway |
| IGHV3-D | IGHV3D | 14 | BCRSignalingPathway |
| IGHV3-H | IGHV3H | 14 | BCRSignalingPathway |
| IGHV4-28 | IGHV428|VH | 14 | BCRSignalingPathway |
| IGHV4-30-1 | IGHV4-3 | 14 | BCRSignalingPathway |
| IGHV4-30-2 | IGHV4-3|IGHV4302 | 14 | BCRSignalingPathway |
| IGHV4-30-4 | IGHV4-3|IGHV4304 | 14 | BCRSignalingPathway |
| IGHV4-31 | FLJ45507|IGHV431 | 14 | BCRSignalingPathway |
| IGHV4-34 | IGHV434|VH | 14 | BCRSignalingPathway |
| IGHV4-39 | IGHV439|VH | 14 | BCRSignalingPathway |
| IGHV4-4 | IGHV44|VH | 14 | BCRSignalingPathway |
| IGHV4-59 | IGHV459|VH | 14 | BCRSignalingPathway |
| IGHV4-61 | IGHV461|VH | 14 | BCRSignalingPathway |
| IGHV4-B | IGHV4B | 14 | BCRSignalingPathway |
| IGHV5-51 | IGHV551|VH | 14 | BCRSignalingPathway |
| IGHV5-A | IGHV5A | 14 | BCRSignalingPathway |
| IGHV6-1 | IGHV61|VH | 14 | BCRSignalingPathway |
| IGHV7-4-1 | IGHV7-41|IGHV741 | 14 | BCRSignalingPathway |
| IGHV7-81 | IGHV781 | 14 | BCRSignalingPathway |
| IGK@ | FLJ26296|IGK|IGKC|MGC22645|MGC27376|MGC40426|MGC71990 | 2 | BCRSignalingPathway |
| IGKC | HCAK1|Km|MGC111575|MGC62011|MGC72072|MGC88770|MGC88771|MGC88809 | 2 | BCRSignalingPathway |
| IGKDEL | IGKDE | 2 | BCRSignalingPathway |
| IGKJ@ | IGKJ | 2 | BCRSignalingPathway |
| IGKJ1 | J1 | 2 | BCRSignalingPathway |
| IGKJ2 | J2 | 2 | BCRSignalingPathway |
| IGKJ3 | J3 | 2 | BCRSignalingPathway |
| IGKJ4 | J4 | 2 | BCRSignalingPathway |
| IGKJ5 | J5 | 2 | BCRSignalingPathway |
| IGKV@ | IGKV|IGKV1|IGKV1@|IGKV2|IGKV2@|IGKV3|IGKV3@ | 2 | BCRSignalingPathway |
| IGKV1-12 | IGKV112|L19 | 2 | BCRSignalingPathway |
| IGKV1-13 | IGKV113|L18 | 2 | BCRSignalingPathway |
| IGKV1-16 | IGKV116|L1 | 2 | BCRSignalingPathway |
| IGKV1-17 | A30|IGKV117 | 2 | BCRSignalingPathway |
| IGKV1-27 | A20|IGKV127 | 2 | BCRSignalingPathway |
| IGKV1-33 | IGKV133|O18 | 2 | BCRSignalingPathway |
| IGKV1-37 | IGKV137|O14 | 2 | BCRSignalingPathway |
| IGKV1-39 | IGKV139|O12|O12a | 2 | BCRSignalingPathway |
| IGKV1-5 | IGKV|IGKV15|L12|L12a|MGC22745|MGC32715|MGC88810|V1 | 2 | BCRSignalingPathway |
| IGKV1-6 | IGKV16|L11 | 2 | BCRSignalingPathway |
| IGKV1-8 | IGKV18|L9 | 2 | BCRSignalingPathway |
| IGKV1-9 | IGKV19|L8 | 2 | BCRSignalingPathway |
| IGKV1D-12 | IGKV1D12|L19 | 2 | BCRSignalingPathway |
| IGKV1D-13 | IGKV1D13|L18 | 2 | BCRSignalingPathway |
| IGKV1D-16 | IGKV1D16|L15|L15a | 2 | BCRSignalingPathway |
| IGKV1D-17 | IGKV1D17|L14 | 2 | BCRSignalingPathway |
| IGKV1D-33 | IGKV1D33|O8 | 2 | BCRSignalingPathway |
| IGKV1D-37 | IGKV1D37|O4 | 2 | BCRSignalingPathway |
| IGKV1D-39 | IGKV1D39|O2 | 2 | BCRSignalingPathway |
| IGKV1D-42 | IGKV1D42|L22 | 2 | BCRSignalingPathway |
| IGKV1D-43 | IGKV1D43|L23|L23a | 2 | BCRSignalingPathway |
| IGKV1D-8 | IGKV1D8|L24|L24a | 2 | BCRSignalingPathway |
| IGKV2-24 | A23|IGKV224 | 2 | BCRSignalingPathway |
| IGKV2-28 | A19|IGKV228 | 2 | BCRSignalingPathway |
| IGKV2-30 | A17|IGKV230 | 2 | BCRSignalingPathway |
| IGKV2-40 | IGKV240|O11|O11a | 2 | BCRSignalingPathway |
| IGKV2D-24 | A7|IGKV2D24 | 2 | BCRSignalingPathway |
| IGKV2D-28 | A3|IGKV2D28 | 2 | BCRSignalingPathway |
| IGKV2D-29 | A2a|A2c|IGKV2D29 | 2 | BCRSignalingPathway |
| IGKV2D-30 | A1|IGKV2D30 | 2 | BCRSignalingPathway |
| IGKV2D-40 | IGKV2D40|O1 | 2 | BCRSignalingPathway |
| IGKV3-11 | IGKV311|L6 | 2 | BCRSignalingPathway |
| IGKV3-15 | IGKV315|L2 | 2 | BCRSignalingPathway |
| IGKV3-20 | 13K18|A27|IGKV320 | 2 | BCRSignalingPathway |
| IGKV3-7 | IGKV37|L10|L10a|Vh | 2 | BCRSignalingPathway |
| IGKV3D-11 | IGKV3D11|L20 | 2 | BCRSignalingPathway |
| IGKV3D-15 | IGKV3D15|L16|L16a|L16b|L16c | 2 | BCRSignalingPathway |
| IGKV3D-20 | A11|A11a|IGKV3D20 | 2 | BCRSignalingPathway |
| IGKV3D-7 | IGKV3D7|L25 | 2 | BCRSignalingPathway |
| IGKV4-1 | B3|IGKV41 | 2 | BCRSignalingPathway |
| IGKV5-2 | B2|IGKV52 | 2 | BCRSignalingPathway |
| IGKV6-21 | A26|IGKV621 | 2 | BCRSignalingPathway |
| IGKV6D-21 | A10|IGKV6D21 | 2 | BCRSignalingPathway |
| IGKV6D-41 | A14 | 2 | BCRSignalingPathway |
| IGL@ | IGL|MGC88804 | 22 | BCRSignalingPathway |
| IGLC@ | IGLC | 22 | BCRSignalingPathway |
| IGLC1 | IGLC | 22 | BCRSignalingPathway |
| IGLC2 | IGLC|MGC20392|MGC45681 | 22 | BCRSignalingPathway |
| IGLC3 | IGLC | 22 | BCRSignalingPathway |
| IGLC6 | IGLC | 22 | BCRSignalingPathway |
| IGLC7 | C7 | 22 | BCRSignalingPathway |
| IGLJ@ | IGLJ | 22 | BCRSignalingPathway |
| IGLJ1 | J1 | 22 | BCRSignalingPathway |
| IGLJ2 | J2 | 22 | BCRSignalingPathway |
| IGLJ3 | J3 | 22 | BCRSignalingPathway |
| IGLJ4 | - | 22 | BCRSignalingPathway |
| IGLJ5 | - | 22 | BCRSignalingPathway |
| IGLJ6 | - | 22 | BCRSignalingPathway |
| IGLJ7 | J7 | 22 | BCRSignalingPathway |
| IGLV@ | IGLV | 22 | BCRSignalingPathway |
| IGLV1-36 | IGLV136|V1-11 | 22 | BCRSignalingPathway |
| IGLV1-40 | IGLV140|V1-13 | 22 | BCRSignalingPathway |
| IGLV1-44 | IGLV144|V1-16 | 22 | BCRSignalingPathway |
| IGLV1-47 | IGLV147|V1-17 | 22 | BCRSignalingPathway |
| IGLV1-50 | IGLV150|V1-18 | 22 | BCRSignalingPathway |
| IGLV1-51 | IGLV151|V1-19 | 22 | BCRSignalingPathway |
| IGLV10-54 | IGLV1054|V1-20 | 22 | BCRSignalingPathway |
| IGLV11-55 | IGLV1155|V4-6 | 22 | BCRSignalingPathway |
| IGLV2-11 | IGLV211|V1-3 | 22 | BCRSignalingPathway |
| IGLV2-14 | IGLV214|V1-4 | 22 | BCRSignalingPathway |
| IGLV2-18 | IGLV218|V1-5 | 22 | BCRSignalingPathway |
| IGLV2-23 | IGLV223|V1-7 | 22 | BCRSignalingPathway |
| IGLV2-33 | IGLV233|V1-9 | 22 | BCRSignalingPathway |
| IGLV2-8 | IGLV28|V1-2 | 22 | BCRSignalingPathway |
| IGLV3-1 | IGLV31|V2-1 | 22 | BCRSignalingPathway |
| IGLV3-10 | IGLV310|V2-7 | 22 | BCRSignalingPathway |
| IGLV3-12 | IGLV312|V2-8 | 22 | BCRSignalingPathway |
| IGLV3-16 | IGLV316|V2-11 | 22 | BCRSignalingPathway |
| IGLV3-19 | IGLV319|V2-13|VL3L | 22 | BCRSignalingPathway |
| IGLV3-21 | IGLV321|V2-14 | 22 | BCRSignalingPathway |
| IGLV3-22 | IGLV322|V2-15 | 22 | BCRSignalingPathway |
| IGLV3-25 | IGLV325|MGC105005|V2-17 | 22 | BCRSignalingPathway |
| IGLV3-27 | IGLV327|V2-19 | 22 | BCRSignalingPathway |
| IGLV3-32 | IGLV332|V2-23P | 22 | BCRSignalingPathway |
| IGLV3-9 | IGLV39|V2-6 | 22 | BCRSignalingPathway |
| IGLV4-3 | IGLV43|V5-1 | 22 | BCRSignalingPathway |
| IGLV4-60 | IGLV460|V5-4 | 22 | BCRSignalingPathway |
| IGLV4-69 | IGLV469|V5-6 | 22 | BCRSignalingPathway |
| IGLV5-37 | IGLV537|V4-1 | 22 | BCRSignalingPathway |
| IGLV5-39 | IGLV539 | 22 | BCRSignalingPathway |
| IGLV5-45 | IGLV545|V4-2 | 22 | BCRSignalingPathway |
| IGLV5-48 | IGLV548|V4-3 | 22 | BCRSignalingPathway |
| IGLV5-52 | IGLV552|V4-4 | 22 | BCRSignalingPathway |
| IGLV6-57 | IGLV657|MGC34845|V1-22 | 22 | BCRSignalingPathway |
| IGLV7-43 | IGLV743|V3-2 | 22 | BCRSignalingPathway |
| IGLV7-46 | IGLV746|V3-3 | 22 | BCRSignalingPathway |
| IGLV8-61 | IGLV861|V3-4 | 22 | BCRSignalingPathway |
| IGLV9-49 | IGLV949|V5-2 | 22 | BCRSignalingPathway |
| C3 | ARMD9|ASP|CPAMD1 | 19 | Chemokines |
| C5 | CPAMD4|FLJ17816|FLJ17822|MGC142298 | 9 | Chemokines |
| CAMP | CAP18|CRAMP|FALL-39|FALL39|HSD26|LL37 | 3 | Chemokines |
| CCL1 | I-309|P500|SCYA1|SISe|TCA3 | 17 | Chemokines |
| CCL11 | MGC22554|SCYA11 | 17 | Chemokines |
| CCL13 | CKb10|MCP-4|MGC17134|NCC-1|NCC1|SCYA13|SCYL1 | 17 | Chemokines |
| CCL14 | CC-1|CC-3|CKb1|FLJ16015|HCC-1|HCC-3|MCIF|NCC-2|NCC2|SCYA14|SCYL2|SY14 | 17 | Chemokines |
| CCL14-CCL15 | - | 17 | Chemokines |
| CCL15 | HCC-2|HMRP-2B|LKN1|Lkn-1|MIP-1d|MIP-5|NCC-3|NCC3|SCYA15|SCYL3|SY15 | 17 | Chemokines |
| CCL16 | CKb12|HCC-4|ILINCK|LCC-1|LEC|LMC|MGC117051|Mtn-1|NCC-4|NCC4|SCYA16|SCYL4 | 17 | Chemokines |
| CCL17 | A-152E5.3|ABCD-2|MGC138271|MGC138273|SCYA17|TARC | 16 | Chemokines |
| CCL18 | AMAC-1|AMAC1|CKb7|DC-CK1|DCCK1|MIP-4|PARC|SCYA18 | 17 | Chemokines |
| CCL19 | CKb11|ELC|MGC34433|MIP-3b|MIP3B|SCYA19 | 9 | Chemokines |
| CCL2 | GDCF-2|HC11|HSMCR30|MCAF|MCP-1|MCP1|MGC9434|SCYA2|SMC-CF | 17 | Chemokines |
| CCL20 | CKb4|LARC|MIP-3a|MIP3A|SCYA20|ST38 | 2 | Chemokines |
| CCL21 | 6Ckine|CKb9|ECL|MGC34555|SCYA21|SLC|TCA4 | 9 | Chemokines |
| CCL22 | A-152E5.1|ABCD-1|DC/B-CK|MDC|MGC34554|SCYA22|STCP-1 | 16 | Chemokines |
| CCL23 | CK-BETA-8|CKb8|Ckb-8|Ckb-8-1|MIP-3|MIP3|MPIF-1|SCYA23 | 17 | Chemokines |
| CCL24 | Ckb-6|MPIF-2|MPIF2|SCYA24 | 7 | Chemokines |
| CCL25 | Ckb15|MGC150327|SCYA25|TECK | 19 | Chemokines |
| CCL26 | IMAC|MGC126714|MIP-4a|MIP-4alpha|SCYA26|TSC-1 | 7 | Chemokines |
| CCL27 | ALP|CTACK|CTAK|ESKINE|ILC|PESKY|SCYA27 | 9 | Chemokines |
| CCL28 | CCK1|MEC|MGC71902|SCYA28 | 5 | Chemokines |
| CCL3 | G0S19-1|LD78ALPHA|MIP-1-alpha|MIP1A|SCYA3 | 17 | Chemokines |
| CCL3L1 | 464.2|D17S1718|G0S19-2|LD78|LD78BETA|MGC104178|MGC12815|MGC182017|MIP1AP|SCYA3L|SCYA3L1 | 17 | Chemokines |
| CCL3L2 | G0S19-3|LD78gamma|SCYA3L2 | 17 | Chemokines |
| CCL3L3 | 464.2|D17S1718|LD78|LD78BETA|MGC12815|SCYA3L|SCYA3L1 | 17 | Chemokines |
| CCL4 | ACT2|AT744.1|G-26|LAG1|MGC104418|MGC126025|MGC126026|MIP-1-beta|MIP1B|MIP1B1|SCYA2|SCYA4 | 17 | Chemokines |
| CCL4L1 | AT744.2|CCL4L|LAG-1|LAG1|SCYA4L | 17 | Chemokines |
| CCL4L2 | AT744.2|CCL4L|SCYA4L | 17 | Chemokines |
| CCL5 | D17S136E|MGC17164|RANTES|SCYA5|SISd|TCP228 | 17 | Chemokines |
| CCL7 | FIC|MARC|MCP-3|MCP3|MGC138463|MGC138465|NC28|SCYA6|SCYA7 | 17 | Chemokines |
| CCL8 | HC14|MCP-2|MCP2|SCYA10|SCYA8 | 17 | Chemokines |
| CKLF | C32|CKLF1|CKLF2|CKLF3|CKLF4|HSPC224|UCK-1 | 16 | Chemokines |
| CMA1 | CYH|MCT1|MGC119890|MGC119891|chymase | 14 | Chemokines |
| CTSG | CG|MGC23078 | 14 | Chemokines |
| CX3CL1 | ABCD-3|C3Xkine|CXC3|CXC3C|NTN|NTT|SCYD1|fractalkine|neurotactin | 16 | Chemokines |
| CXCL1 | FSP|GRO1|GROa|MGSA|MGSA-a|NAP-3|SCYB1 | 4 | Chemokines |
| CXCL10 | C7|IFI10|INP10|IP-10|SCYB10|crg-2|gIP-10|mob-1 | 4 | Chemokines |
| CXCL11 | H174|I-TAC|IP-9|IP9|MGC102770|SCYB11|SCYB9B|b-R1 | 4 | Chemokines |
| CXCL12 | PBSF|SCYB12|SDF-1a|SDF-1b|SDF1|SDF1A|SDF1B|TLSF-a|TLSF-b|TPAR1 | 10 | Chemokines |
| CXCL13 | ANGIE|ANGIE2|BCA-1|BCA1|BLC|BLR1L|SCYB13 | 4 | Chemokines |
| CXCL14 | BMAC|BRAK|KS1|Kec|MGC10687|MIP-2g|NJAC|SCYB14|bolekine | 5 | Chemokines |
| CXCL16 | CXCLG16|SR-PSOX|SRPSOX | 17 | Chemokines |
| CXCL17 | DMC|Dcip1|MGC138300|UNQ473|VCC-1|VCC1 | 19 | Chemokines |
| CXCL2 | CINC-2a|GRO2|GROb|MGSA-b|MIP-2a|MIP2|MIP2A|SCYB2 | 4 | Chemokines |
| CXCL3 | CINC-2b|GRO3|GROg|MIP-2b|MIP2B|SCYB3 | 4 | Chemokines |
| CXCL5 | ENA-78|SCYB5 | 4 | Chemokines |
| CXCL6 | CKA-3|GCP-2|GCP2|SCYB6 | 4 | Chemokines |
| CXCL9 | CMK|Humig|MIG|SCYB9|crg-10 | 4 | Chemokines |
| CYR61 | CCN1|GIG1|IGFBP10 | 1 | Chemokines |
| DEFA1 | DEF1|DEFA2|HNP-1|HP-1|MGC138393|MRS | 8 | Chemokines |
| DEFA3 | DEF3|HNP-3|HNP3|HP-3 | 8 | Chemokines |
| DEFA5 | DEF5|HD-5|MGC129728 | 8 | Chemokines |
| DEFB1 | BD1|DEFB-1|DEFB101|HBD1|MGC51822 | 8 | Chemokines |
| DEFB103A | DEFB103|DEFB3|HBD-3|HBD3|HBP-3|HBP3 | 8 | Chemokines |
| DEFB104A | BD-4|DEFB-4|DEFB104|DEFB4|MGC118942|MGC118944|MGC118945|hBD-4 | 8 | Chemokines |
| DEFB4 | DEFB-2|DEFB102|DEFB2|HBD-2|SAP1 | 8 | Chemokines |
| EDN1 | ET1|HDLCQ7 | 6 | Chemokines |
| EDN2 | ET2|PPET2 | 1 | Chemokines |
| EDN3 | ET3|MGC15067|MGC61498 | 20 | Chemokines |
| FGF10 | - | 5 | Chemokines |
| FGF2 | BFGF|FGFB|HBGF-2 | 4 | Chemokines |
| HTN3 | HIS2|HTN2|HTN5 | 4 | Chemokines |
| IL8 | CXCL8|GCP-1|GCP1|LECT|LUCT|LYNAP|MDNCF|MONAP|NAF|NAP-1|NAP1 | 4 | Chemokines |
| LECT2 | MGC126628|chm-II|chm2 | 5 | Chemokines |
| PF4 | CXCL4|MGC138298|SCYB4 | 4 | Chemokines |
| PF4V1 | CXCL4L1|CXCL4V1|PF4-ALT|PF4A|SCYB4V1 | 4 | Chemokines |
| PLAU | ATF|UPA|URK|u-PA | 10 | Chemokines |
| PPBP | B-TG1|Beta-TG|CTAP-III|CTAP3|CTAPIII|CXCL7|LA-PF4|LDGF|MDGF|NAP-2|PBP|SCYB7|TC1|TC2|TGB|TGB1|THBGB|THBGB1 | 4 | Chemokines |
| PPBPL1 | TGB2 | 4 | Chemokines |
| PROK2 | BV8|KAL4|MIT1|PK2 | 3 | Chemokines |
| RNASE2 | EDN|RNS2 | 14 | Chemokines |
| SAA1 | MGC111216|PIG4|SAA|TP53I4 | 11 | Chemokines |
| SAA2 | - | 11 | Chemokines |
| SBDS | CGI-97|FLJ10917|SDS|SWDS | 7 | Chemokines |
| SEMA3A | Hsema-I|Hsema-III|MGC133243|SEMA1|SEMAD|SEMAIII|SEMAL|SemD|coll-1 | 7 | Chemokines |
| SEMA3B | FLJ34863|LUCA-1|SEMA5|SEMAA|SemA|semaV | 3 | Chemokines |
| SEMA3C | SEMAE|SemE | 7 | Chemokines |
| SEMA3D | MGC39708|Sema-Z2|coll-2 | 7 | Chemokines |
| SEMA3E | KIAA0331|M-SEMAH|M-SemaK|SEMAH|coll-5 | 7 | Chemokines |
| SEMA3F | SEMA-IV|SEMA4|SEMAK | 3 | Chemokines |
| SEMA3G | FLJ00014|MGC119473|sem2 | 3 | Chemokines |
| SEMA4A | CORD10|FLJ12287|RP35|SEMAB|SEMB | 1 | Chemokines |
| SEMA4B | KIAA1745|MGC131831|SEMAC|SemC | 15 | Chemokines |
| SEMA4C | FLJ20369|KIAA1739|M-SEMA-F|MGC126382|MGC126383|SEMACL1|SEMAF|SEMAI | 2 | Chemokines |
| SEMA4D | C9orf164|CD100|FLJ33485|FLJ34282|FLJ39737|FLJ46484|M-sema-G|MGC169138|MGC169141|SEMAJ|coll-4 | 9 | Chemokines |
| SEMA4F | M-SEMA|PRO2353|SEMAM|SEMAW|m-Sema-M | 2 | Chemokines |
| SEMA4G | FLJ20590|KIAA1619|MGC102867 | 10 | Chemokines |
| SEMA5A | FLJ12815|SEMAF|semF | 5 | Chemokines |
| SEMA5B | FLJ10372|KIAA1445|SEMAG|SemG | 3 | Chemokines |
| SEMA6A | HT018|KIAA1368|SEMA|SEMA6A1|SEMAQ|VIA | 5 | Chemokines |
| SEMA6B | SEM-SEMA-Y|SEMA-VIB|SEMAN|semaZ | 19 | Chemokines |
| SEMA6C | SEMAY|m-SemaY|m-SemaY2 | 1 | Chemokines |
| SEMA6D | FLJ11598|KIAA1479 | 15 | Chemokines |
| SEMA7A | CD108|CDw108|H-SEMA-K1|H-Sema-L|JMH|MGC126692|MGC126696|SEMAK1|SEMAL | 15 | Chemokines |
| SLIT1 | MEGF4|MGC164811|SLIL1|SLIT3|Slit-1 | 10 | Chemokines |
| SLIT2 | FLJ14420|SLIL3|Slit-2 | 4 | Chemokines |
| TNC | HXB|MGC167029|TN | 9 | Chemokines |
| TYMP | ECGF1|MNGIE|PDECGF|TP|hPD-ECGF | 22 | Chemokines |
| XCL1 | ATAC|LPTN|LTN|SCM-1|SCM-1a|SCM1|SCYC1 | 1 | Chemokines |
| XCL2 | SCM-1b|SCM1B|SCYC2 | 1 | Chemokines |
| C5AR1 | C5A|C5AR|C5R1|CD88 | 19 | Chemokine_Receptors |
| CCBP2 | CCR10|CCR9|CMKBR9|D6|MGC126678|MGC138250|hD6 | 3 | Chemokine_Receptors |
| CCR1 | CD191|CKR-1|CKR1|CMKBR1|HM145|MIP1aR|SCYAR1 | 3 | Chemokine_Receptors |
| CCR10 | GPR2 | 17 | Chemokine_Receptors |
| CCR3 | CC-CKR-3|CD193|CKR3|CMKBR3|MGC102841 | 3 | Chemokine_Receptors |
| CCR4 | CC-CKR-4|CD194|CKR4|CMKBR4|ChemR13|HGCN:14099|K5-5|MGC88293 | 3 | Chemokine_Receptors |
| CCR5 | CC-CKR-5|CCCKR5|CD195|CKR-5|CKR5|CMKBR5|FLJ78003|IDDM22 | 3 | Chemokine_Receptors |
| CCR6 | BN-1|CD196|CKR-L3|CKR6|CKRL3|CMKBR6|DCR2|DRY-6|GPR-CY4|GPR29|GPRCY4|STRL22 | 6 | Chemokine_Receptors |
| CCR7 | BLR2|CD197|CDw197|CMKBR7|EBI1 | 17 | Chemokine_Receptors |
| CCR8 | CDw198|CKR-L1|CKRL1|CMKBR8|CMKBRL2|CY6|GPR-CY6|MGC129966|MGC129973|TER1 | 3 | Chemokine_Receptors |
| CCR9 | CDw199|GPR-9-6|GPR28 | 3 | Chemokine_Receptors |
| CCRL1 | CC-CKR-11|CCBP2|CCR10|CCR11|CCX-CKR|CKR-11|PPR1|VSHK1 | 3 | Chemokine_Receptors |
| CCRL2 | CKRX|CRAM-A|CRAM-B|FLJ55815|HCR|MGC116710|MGC34104 | 3 | Chemokine_Receptors |
| CMKLR1 | CHEMERINR|ChemR23|DEZ|MGC126105|MGC126106 | 12 | Chemokine_Receptors |
| CX3CR1 | CCRL1|CMKBRL1|CMKDR1|GPR13|GPRV28|V28 | 3 | Chemokine_Receptors |
| CXCR3 | CD182|CD183|CKR-L2|CMKAR3|GPR9|IP10-R|Mig-R|MigR | X | Chemokine_Receptors |
| CXCR4 | CD184|D2S201E|FB22|HM89|HSY3RR|LAP3|LCR1|LESTR|NPY3R|NPYR|NPYRL|NPYY3R|WHIM | 2 | Chemokine_Receptors |
| CXCR5 | BLR1|CD185|MDR15|MGC117347 | 11 | Chemokine_Receptors |
| CXCR6 | BONZO|CD186|STRL33|TYMSTR | 3 | Chemokine_Receptors |
| CXCR7 | CMKOR1|GPR159|RDC1 | 2 | Chemokine_Receptors |
| CYSLTR1 | CYSLT1|CYSLT1R|CYSLTR|HG55|HMTMF81|MGC46139 | X | Chemokine_Receptors |
| CYSLTR2 | CYSLT2|CYSLT2R|GPCR|HG57|HPN321|KPG_011|PSEC0146|hGPCR21 | 13 | Chemokine_Receptors |
| DARC | CCBP1|CD234|Dfy|FY|GPD|GpFy|WBCQ1 | 1 | Chemokine_Receptors |
| EDNRA | ETA|ETRA | 4 | Chemokine_Receptors |
| EDNRB | ABCDS|ETB|ETBR|ETRB|HSCR|HSCR2 | 13 | Chemokine_Receptors |
| FPR1 | FMLP|FPR | 19 | Chemokine_Receptors |
| FPR2 | ALXR|FMLP-R-II|FMLPX|FPR2A|FPRH1|FPRH2|FPRL1|HM63|LXA4R | 19 | Chemokine_Receptors |
| FPR2 | ALXR|FMLP-R-II|FMLPX|FPR2A|FPRH1|FPRH2|FPRL1|HM63|LXA4R | 19 | Chemokine_Receptors |
| GPR17 | DKFZp686M18273 | 2 | Chemokine_Receptors |
| GPR32 | - | 19 | Chemokine_Receptors |
| GPR33 | - | 14 | Chemokine_Receptors |
| GPR44 | CD294|CRTH2|DP2 | 11 | Chemokine_Receptors |
| GPR77 | C5L2|GPF77 | 19 | Chemokine_Receptors |
| IL8RA | C-C|C-C-CKR-1|CD128|CD181|CDw128a|CKR-1|CMKAR1|CXCR1|IL8R1|IL8RBA | 2 | Chemokine_Receptors |
| IL8RB | CD182|CDw128b|CMKAR2|CXCR2|IL8R2|IL8RA | 2 | Chemokine_Receptors |
| LTB4R | BLT1|BLTR|CMKRL1|GPR16|LTB4R1|LTBR1|P2RY7|P2Y7 | 14 | Chemokine_Receptors |
| LTB4R2 | BLT2|BLTR2|JULF2|KPG_004|NOP9 | 14 | Chemokine_Receptors |
| PLAUR | CD87|UPAR|URKR | 19 | Chemokine_Receptors |
| PLXNA1 | NOV|NOVP|PLEXIN-A1|PLXN1 | 3 | Chemokine_Receptors |
| PLXNA2 | FLJ11751|FLJ30634|KIAA0463|OCT|PLXN2 | 1 | Chemokine_Receptors |
| PLXNA3 | 6.3|HSSEXGENE|PLEXIN-A3|PLXN3|PLXN4|SEX|XAP-6 | X | Chemokine_Receptors |
| PLXNA4 | DKFZp434G0625|DKFZp566O0546|FAYV2820|FLJ35026|FLJ38287|KIAA1550|PLEXA4|PLXNA4A|PLXNA4B|PRO34003 | 7 | Chemokine_Receptors |
| PLXNB1 | KIAA0407|MGC149167|PLEXIN-B1|PLXN5|SEP | 3 | Chemokine_Receptors |
| PLXNB2 | KIAA0315|MM1|Nbla00445|PLEXB2|dJ402G11.3 | 22 | Chemokine_Receptors |
| PLXNB3 | FLJ76953|PLEXB3|PLEXR|PLXN6 | X | Chemokine_Receptors |
| PLXNC1 | CD232|PLXN-C1|VESPR | 12 | Chemokine_Receptors |
| PLXND1 | KIAA0620|MGC75353|PLEXD1 | 3 | Chemokine_Receptors |
| PTAFR | PAFR | 1 | Chemokine_Receptors |
| ROBO1 | DUTT1|FLJ21882|MGC131599|MGC133277|SAX3 | 3 | Chemokine_Receptors |
| ROBO2 | KIAA1568|SAX3 | 3 | Chemokine_Receptors |
| ROBO3 | FLJ21044|HGPPS|HGPS|RBIG1|RIG1 | 11 | Chemokine_Receptors |
| RXFP3 | GPCR135|MGC141998|MGC142000|RLN3R1|RXFPR3|SALPR | 5 | Chemokine_Receptors |
| XCR1 | CCXCR1|GPR5 | 3 | Chemokine_Receptors |
| ADIPOQ | ACDC|ACRP30|ADIPQTL1|ADPN|APM-1|APM1|GBP28|adiponectin | 3 | Cytokines |
| ADM | AM | 11 | Cytokines |
| ADM2 | AM2|FLJ21135|dJ579N16.4 | 22 | Cytokines |
| AGRP | AGRT|ART|ASIP2|MGC118963 | 16 | Cytokines |
| AGT | ANHU|FLJ92595|FLJ97926|SERPINA8 | 1 | Cytokines |
| AMBN | - | 4 | Cytokines |
| AMELX | AIH1|ALGN|AMG|AMGL|AMGX | X | Cytokines |
| AMH | MIF|MIS | 19 | Cytokines |
| ANGPTL5 | - | 11 | Cytokines |
| ANGPTL7 | AngX|CDT6|RP4-647M16.2|dJ647M16.1 | 1 | Cytokines |
| APLN | XNPEP2 | X | Cytokines |
| AREG | AR|CRDGF|MGC13647|SDGF | 4 | Cytokines |
| ARMET | ARP|MANF|MGC142148|MGC142150 | 3 | Cytokines |
| ARMETL1 | cdnf | 10 | Cytokines |
| ARTN | ENOVIN|EVN|NBN | 1 | Cytokines |
| AVP | ADH|ARVP|AVP-NPII|AVRP|VP | 20 | Cytokines |
| AZU1 | AZAMP|AZU|CAP37|HBP|HUMAZUR|NAZC | 19 | Cytokines |
| BDNF | MGC34632 | 11 | Cytokines |
| BMP1 | FLJ44432|PCOLC|PCP|TLD|pCP-2 | 8 | Cytokines |
| BMP10 | MGC126783 | 2 | Cytokines |
| BMP15 | GDF9B|ODG2|POF4 | X | Cytokines |
| BMP2 | BMP2A | 20 | Cytokines |
| BMP3 | BMP-3A | 4 | Cytokines |
| BMP4 | BMP2B|BMP2B1|MCOPS6|OFC11|ZYME | 14 | Cytokines |
| BMP5 | MGC34244 | 6 | Cytokines |
| BMP6 | VGR|VGR1 | 6 | Cytokines |
| BMP7 | OP-1 | 20 | Cytokines |
| BMP8A | FLJ14351|FLJ45264 | 1 | Cytokines |
| BMP8B | BMP8|MGC131757|OP2 | 1 | Cytokines |
| BTC | - | 4 | Cytokines |
| C19orf10 | EUROIMAGE1875335|IL25|IL27|IL27w|R33729_1|SF20 | 19 | Cytokines |
| C3 | ARMD9|ASP|CPAMD1 | 19 | Cytokines |
| C5 | CPAMD4|FLJ17816|FLJ17822|MGC142298 | 9 | Cytokines |
| CALCA | CALC1|CGRP|CGRP-I|CGRP1|CT|KC|MGC126648 | 11 | Cytokines |
| CALCB | CALC2|CGRP-II|CGRP2|FLJ30166 | 11 | Cytokines |
| CAMP | CAP18|CRAMP|FALL-39|FALL39|HSD26|LL37 | 3 | Cytokines |
| CAT | MGC138422|MGC138424 | 11 | Cytokines |
| CCK | MGC117187 | 3 | Cytokines |
| CCL1 | I-309|P500|SCYA1|SISe|TCA3 | 17 | Cytokines |
| CCL11 | MGC22554|SCYA11 | 17 | Cytokines |
| CCL13 | CKb10|MCP-4|MGC17134|NCC-1|NCC1|SCYA13|SCYL1 | 17 | Cytokines |
| CCL14 | CC-1|CC-3|CKb1|FLJ16015|HCC-1|HCC-3|MCIF|NCC-2|NCC2|SCYA14|SCYL2|SY14 | 17 | Cytokines |
| CCL14-CCL15 | - | 17 | Cytokines |
| CCL15 | HCC-2|HMRP-2B|LKN1|Lkn-1|MIP-1d|MIP-5|NCC-3|NCC3|SCYA15|SCYL3|SY15 | 17 | Cytokines |
| CCL16 | CKb12|HCC-4|ILINCK|LCC-1|LEC|LMC|MGC117051|Mtn-1|NCC-4|NCC4|SCYA16|SCYL4 | 17 | Cytokines |
| CCL17 | A-152E5.3|ABCD-2|MGC138271|MGC138273|SCYA17|TARC | 16 | Cytokines |
| CCL18 | AMAC-1|AMAC1|CKb7|DC-CK1|DCCK1|MIP-4|PARC|SCYA18 | 17 | Cytokines |
| CCL19 | CKb11|ELC|MGC34433|MIP-3b|MIP3B|SCYA19 | 9 | Cytokines |
| CCL2 | GDCF-2|HC11|HSMCR30|MCAF|MCP-1|MCP1|MGC9434|SCYA2|SMC-CF | 17 | Cytokines |
| CCL20 | CKb4|LARC|MIP-3a|MIP3A|SCYA20|ST38 | 2 | Cytokines |
| CCL21 | 6Ckine|CKb9|ECL|MGC34555|SCYA21|SLC|TCA4 | 9 | Cytokines |
| CCL22 | A-152E5.1|ABCD-1|DC/B-CK|MDC|MGC34554|SCYA22|STCP-1 | 16 | Cytokines |
| CCL23 | CK-BETA-8|CKb8|Ckb-8|Ckb-8-1|MIP-3|MIP3|MPIF-1|SCYA23 | 17 | Cytokines |
| CCL24 | Ckb-6|MPIF-2|MPIF2|SCYA24 | 7 | Cytokines |
| CCL25 | Ckb15|MGC150327|SCYA25|TECK | 19 | Cytokines |
| CCL26 | IMAC|MGC126714|MIP-4a|MIP-4alpha|SCYA26|TSC-1 | 7 | Cytokines |
| CCL27 | ALP|CTACK|CTAK|ESKINE|ILC|PESKY|SCYA27 | 9 | Cytokines |
| CCL28 | CCK1|MEC|MGC71902|SCYA28 | 5 | Cytokines |
| CCL3 | G0S19-1|LD78ALPHA|MIP-1-alpha|MIP1A|SCYA3 | 17 | Cytokines |
| CCL3L1 | 464.2|D17S1718|G0S19-2|LD78|LD78BETA|MGC104178|MGC12815|MGC182017|MIP1AP|SCYA3L|SCYA3L1 | 17 | Cytokines |
| CCL3L2 | G0S19-3|LD78gamma|SCYA3L2 | 17 | Cytokines |
| CCL3L3 | 464.2|D17S1718|LD78|LD78BETA|MGC12815|SCYA3L|SCYA3L1 | 17 | Cytokines |
| CCL4 | ACT2|AT744.1|G-26|LAG1|MGC104418|MGC126025|MGC126026|MIP-1-beta|MIP1B|MIP1B1|SCYA2|SCYA4 | 17 | Cytokines |
| CCL4L1 | AT744.2|CCL4L|LAG-1|LAG1|SCYA4L | 17 | Cytokines |
| CCL4L2 | AT744.2|CCL4L|SCYA4L | 17 | Cytokines |
| CCL5 | D17S136E|MGC17164|RANTES|SCYA5|SISd|TCP228 | 17 | Cytokines |
| CCL7 | FIC|MARC|MCP-3|MCP3|MGC138463|MGC138465|NC28|SCYA6|SCYA7 | 17 | Cytokines |
| CCL8 | HC14|MCP-2|MCP2|SCYA10|SCYA8 | 17 | Cytokines |
| CD320 | 8D6|8D6A | 19 | Cytokines |
| CD40LG | CD154|CD40L|HIGM1|IGM|IMD3|T-BAM|TNFSF5|TRAP|gp39|hCD40L | X | Cytokines |
| CD70 | CD27L|CD27LG|TNFSF7 | 19 | Cytokines |
| CECR1 | ADGF|IDGFL | 22 | Cytokines |
| CER1 | DAND4|MGC119894|MGC119895|MGC96951 | 9 | Cytokines |
| CGA | CG-ALPHA|FSHA|GPHA1|GPHa|HCG|LHA|TSHA | 6 | Cytokines |
| CGB | CGB3|hCGB | 19 | Cytokines |
| CGB1 | - | 19 | Cytokines |
| CGB2 | - | 19 | Cytokines |
| CGB5 | HCG|MGC119822 | 19 | Cytokines |
| CGB7 | CG-beta-a|FLJ35403|FLJ43118 | 19 | Cytokines |
| CGB8 | - | 19 | Cytokines |
| CHGA | CGA | 14 | Cytokines |
| CHGB | SCG1 | 20 | Cytokines |
| CKLF | C32|CKLF1|CKLF2|CKLF3|CKLF4|HSPC224|UCK-1 | 16 | Cytokines |
| CLCF1 | BSF3|CISS2|CLC|NNT1|NR6 | 11 | Cytokines |
| CLEC11A | CLECSF3|LSLCL|P47|SCGF | 19 | Cytokines |
| CMA1 | CYH|MCT1|MGC119890|MGC119891|chymase | 14 | Cytokines |
| CMTM1 | CKLFH|CKLFH1|CKLFSF1|MGC71870 | 16 | Cytokines |
| CMTM2 | CKLFSF2|MGC39436 | 16 | Cytokines |
| CMTM3 | BNAS2|CKLFSF3|FLJ31762|MGC51956 | 16 | Cytokines |
| CMTM4 | CKLFSF4 | 16 | Cytokines |
| CMTM5 | CKLFSF5|FLJ37521 | 14 | Cytokines |
| CMTM6 | CKLFSF6|FLJ20396|PRO2219 | 3 | Cytokines |
| CMTM7 | CKLFSF7|FLJ30992 | 3 | Cytokines |
| CMTM8 | CKLFSF8|CKLFSF8-V2 | 3 | Cytokines |
| CNTF | HCNTF | 11 | Cytokines |
| CORT | CST-14|CST-17|CST-29 | 1 | Cytokines |
| CRH | CRF | 8 | Cytokines |
| CSF1 | MCSF|MGC31930 | 1 | Cytokines |
| CSF2 | GMCSF|MGC131935|MGC138897 | 5 | Cytokines |
| CSF3 | G-CSF|GCSF|MGC45931 | 17 | Cytokines |
| CSH1 | CSA|CSMT|FLJ75407|PL | 17 | Cytokines |
| CSH2 | CS-2|CSB|hCS-B | 17 | Cytokines |
| CSHL1 | CS-5|CSHP1|CSL|MGC149868|hCS-L | 17 | Cytokines |
| CSPG5 | MGC44034|NGC | 3 | Cytokines |
| CTF1 | CT-1|CT1 | 16 | Cytokines |
| CTGF | CCN2|HCS24|IGFBP8|MGC102839|NOV2 | 6 | Cytokines |
| CTSG | CG|MGC23078 | 14 | Cytokines |
| CX3CL1 | ABCD-3|C3Xkine|CXC3|CXC3C|NTN|NTT|SCYD1|fractalkine|neurotactin | 16 | Cytokines |
| CXCL1 | FSP|GRO1|GROa|MGSA|MGSA-a|NAP-3|SCYB1 | 4 | Cytokines |
| CXCL10 | C7|IFI10|INP10|IP-10|SCYB10|crg-2|gIP-10|mob-1 | 4 | Cytokines |
| CXCL11 | H174|I-TAC|IP-9|IP9|MGC102770|SCYB11|SCYB9B|b-R1 | 4 | Cytokines |
| CXCL12 | PBSF|SCYB12|SDF-1a|SDF-1b|SDF1|SDF1A|SDF1B|TLSF-a|TLSF-b|TPAR1 | 10 | Cytokines |
| CXCL13 | ANGIE|ANGIE2|BCA-1|BCA1|BLC|BLR1L|SCYB13 | 4 | Cytokines |
| CXCL14 | BMAC|BRAK|KS1|Kec|MGC10687|MIP-2g|NJAC|SCYB14|bolekine | 5 | Cytokines |
| CXCL16 | CXCLG16|SR-PSOX|SRPSOX | 17 | Cytokines |
| CXCL17 | DMC|Dcip1|MGC138300|UNQ473|VCC-1|VCC1 | 19 | Cytokines |
| CXCL2 | CINC-2a|GRO2|GROb|MGSA-b|MIP-2a|MIP2|MIP2A|SCYB2 | 4 | Cytokines |
| CXCL3 | CINC-2b|GRO3|GROg|MIP-2b|MIP2B|SCYB3 | 4 | Cytokines |
| CXCL5 | ENA-78|SCYB5 | 4 | Cytokines |
| CXCL6 | CKA-3|GCP-2|GCP2|SCYB6 | 4 | Cytokines |
| CXCL9 | CMK|Humig|MIG|SCYB9|crg-10 | 4 | Cytokines |
| CYR61 | CCN1|GIG1|IGFBP10 | 1 | Cytokines |
| DEFA1 | DEF1|DEFA2|HNP-1|HP-1|MGC138393|MRS | 8 | Cytokines |
| DEFA3 | DEF3|HNP-3|HNP3|HP-3 | 8 | Cytokines |
| DEFA5 | DEF5|HD-5|MGC129728 | 8 | Cytokines |
| DEFB1 | BD1|DEFB-1|DEFB101|HBD1|MGC51822 | 8 | Cytokines |
| DEFB103A | DEFB103|DEFB3|HBD-3|HBD3|HBP-3|HBP3 | 8 | Cytokines |
| DEFB104A | BD-4|DEFB-4|DEFB104|DEFB4|MGC118942|MGC118944|MGC118945|hBD-4 | 8 | Cytokines |
| DEFB4 | DEFB-2|DEFB102|DEFB2|HBD-2|SAP1 | 8 | Cytokines |
| DKK1 | DKK-1|SK | 10 | Cytokines |
| EBI3 | IL27B | 19 | Cytokines |
| EDN1 | ET1|HDLCQ7 | 6 | Cytokines |
| EDN2 | ET2|PPET2 | 1 | Cytokines |
| EDN3 | ET3|MGC15067|MGC61498 | 20 | Cytokines |
| EGF | HOMG4|URG | 4 | Cytokines |
| EPGN | ALGV3072|EPG|FLJ75542|PRO9904|epigen | 4 | Cytokines |
| EPO | EP|MGC138142|MVCD2 | 7 | Cytokines |
| EREG | ER | 4 | Cytokines |
| ESM1 | endocan | 5 | Cytokines |
| FAM3B | 2-21|C21orf11|C21orf76|ORF9|PANDER|PRED44 | 21 | Cytokines |
| FAM3C | GS3786|ILEI | 7 | Cytokines |
| FAM3D | EF7|OIT1 | 3 | Cytokines |
| FASLG | APT1LG1|CD178|CD95L|FASL|TNFSF6 | 1 | Cytokines |
| FGF1 | AFGF|ECGF|ECGF-beta|ECGFA|ECGFB|FGF-alpha|FGFA|GLIO703|HBGF1 | 5 | Cytokines |
| FGF10 | - | 5 | Cytokines |
| FGF11 | FHF3|FLJ16061|MGC102953|MGC45269 | 17 | Cytokines |
| FGF12 | FGF12B|FHF1 | 3 | Cytokines |
| FGF13 | FGF2|FHF-2|FHF2 | X | Cytokines |
| FGF14 | FHF4|MGC119129|SCA27 | 13 | Cytokines |
| FGF16 | - | X | Cytokines |
| FGF17 | FGF-13 | 8 | Cytokines |
| FGF18 | FGF-18|ZFGF5 | 5 | Cytokines |
| FGF19 | - | 11 | Cytokines |
| FGF2 | BFGF|FGFB|HBGF-2 | 4 | Cytokines |
| FGF20 | - | 8 | Cytokines |
| FGF21 | - | 19 | Cytokines |
| FGF22 | - | 19 | Cytokines |
| FGF23 | ADHR|HPDR2|HYPF|PHPTC | 12 | Cytokines |
| FGF3 | HBGF-3|INT2 | 11 | Cytokines |
| FGF4 | HBGF-4|HST|HST-1|HSTF1|K-FGF|KFGF | 11 | Cytokines |
| FGF5 | HBGF-5|Smag-82 | 4 | Cytokines |
| FGF6 | HBGF-6|HST2 | 12 | Cytokines |
| FGF7 | HBGF-7|KGF | 15 | Cytokines |
| FGF8 | AIGF|HBGF-8|KAL6|MGC149376 | 10 | Cytokines |
| FGF9 | GAF|HBFG-9|MGC119914|MGC119915 | 13 | Cytokines |
| FIGF | VEGF-D|VEGFD | X | Cytokines |
| FIGNL2 | - | 12 | Cytokines |
| FLT3LG | FL | 19 | Cytokines |
| FSHB | - | 11 | Cytokines |
| GAL | GALN|GLNN|GMAP|MGC40167 | 11 | Cytokines |
| GALP | - | 19 | Cytokines |
| GAST | GAS | 17 | Cytokines |
| GCG | GLP1|GLP2|GRPP | 2 | Cytokines |
| GDF1 | - | 19 | Cytokines |
| GDF10 | BMP-3b|BMP3B | 10 | Cytokines |
| GDF11 | BMP-11|BMP11 | 12 | Cytokines |
| GDF15 | GDF-15|MIC-1|MIC1|NAG-1|PDF|PLAB|PTGFB | 19 | Cytokines |
| GDF2 | BMP-9|BMP9 | 10 | Cytokines |
| GDF3 | - | 12 | Cytokines |
| GDF5 | BMP14|CDMP1|LAP4|OS5|SYNS2 | 20 | Cytokines |
| GDF6 | BMP13|CDMP2|KFS|KFSL|MGC158100|MGC158101|SGM1 | 8 | Cytokines |
| GDF7 | BMP12 | 2 | Cytokines |
| GDF9 | - | 5 | Cytokines |
| GDNF | ATF1|ATF2|HFB1-GDNF | 5 | Cytokines |
| GH1 | GH|GH-N|GHN|hGH-N | 17 | Cytokines |
| GH2 | GH-V|GHL|GHV|hGH-V | 17 | Cytokines |
| GHRH | GHRF|GRF|MGC119781 | 20 | Cytokines |
| GHRL | MTLRP|obestatin | 3 | Cytokines |
| GIP | - | 17 | Cytokines |
| GKN1 | AMP18|BRICD1|CA11|FOV|MGC70354|foveolin | 2 | Cytokines |
| GMFB | GMF | 14 | Cytokines |
| GMFG | GMF-GAMMA|MGC126867 | 19 | Cytokines |
| GNRH1 | GNRH|GRH|LHRH|LNRH | 8 | Cytokines |
| GNRH2 | GnRH-II|LH-RHII | 20 | Cytokines |
| GPHA2 | A2|GPA2|MGC126572|ZSIG51 | 11 | Cytokines |
| GPHB5 | B5|GPB5|ZLUT1 | 14 | Cytokines |
| GPI | AMF|GNPI|NLK|PGI|PHI|SA-36 | 19 | Cytokines |
| GREM1 | CKTSF1B1|DAND2|DRM|GREMLIN|IHG-2|MGC126660|PIG2 | 15 | Cytokines |
| GREM2 | CKTSF1B2|DAND3|PRDC | 1 | Cytokines |
| GRN | GEP|GP88|PCDGF|PEPI|PGRN | 17 | Cytokines |
| GRP | BN|GRP-10|preproGRP|proGRP | 18 | Cytokines |
| GUCA2A | GUANYLIN|GUCA2|STARA | 1 | Cytokines |
| HAMP | HEPC|HEPCIDIN|HFE2B|LEAP-1|LEAP1|PLTR | 19 | Cytokines |
| HBEGF | DTR|DTS|DTSF|HEGFL | 5 | Cytokines |
| HDGF | DKFZp686J1764|FLJ96580|HMG1L2 | 1 | Cytokines |
| HDGFRP3 | CGI-142|HDGF2 | 15 | Cytokines |
| HGF | F-TCF|HGFB|HPTA|SF | 7 | Cytokines |
| HTN3 | HIS2|HTN2|HTN5 | 4 | Cytokines |
| IAPP | AMYLIN|DAP|IAP | 12 | Cytokines |
| IFNA1 | IFL|IFN|IFN-ALPHA|IFNA13|IFNA@|MGC138207|MGC138505|MGC138507 | 9 | Cytokines |
| IFNA10 | MGC119878|MGC119879 | 9 | Cytokines |
| IFNA13 | - | 9 | Cytokines |
| IFNA14 | LEIF2H|MGC125756|MGC125757 | 9 | Cytokines |
| IFNA16 | - | 9 | Cytokines |
| IFNA17 | IFNA|INFA|LEIF2C1 | 9 | Cytokines |
| IFNA2 | IFNA|INFA2|MGC125764|MGC125765 | 9 | Cytokines |
| IFNA21 | MGC126687|MGC126689 | 9 | Cytokines |
| IFNA4 | INFA4|MGC142200 | 9 | Cytokines |
| IFNA5 | INFA5 | 9 | Cytokines |
| IFNA6 | - | 9 | Cytokines |
| IFNA7 | IFNA-J | 9 | Cytokines |
| IFNA8 | - | 9 | Cytokines |
| IFNB1 | IFB|IFF|IFNB|MGC96956 | 9 | Cytokines |
| IFNE | IFN-E|IFNE1|IFNT1|MGC119018|MGC119020|PRO655 | 9 | Cytokines |
| IFNG | IFG|IFI | 12 | Cytokines |
| IFNK | RP11-27J8.1 | 9 | Cytokines |
| IFNW1 | - | 9 | Cytokines |
| IGF1 | IGF1A|IGFI | 12 | Cytokines |
| IGF2 | C11orf43|FLJ22066|FLJ44734|INSIGF|pp9974 | 11 | Cytokines |
| IL10 | CSIF|IL-10|IL10A|MGC126450|MGC126451|TGIF | 1 | Cytokines |
| IL11 | AGIF|IL-11 | 19 | Cytokines |
| IL12A | CLMF|IL-12A|NFSK|NKSF1|P35 | 3 | Cytokines |
| IL12B | CLMF|CLMF2|IL-12B|NKSF|NKSF2 | 5 | Cytokines |
| IL13 | ALRH|BHR1|IL-13|MGC116786|MGC116788|MGC116789|P600 | 5 | Cytokines |
| IL15 | IL-15|MGC9721 | 4 | Cytokines |
| IL16 | FLJ16806|FLJ42735|FLJ44234|HsT19289|IL-16|LCF|prIL-16 | 15 | Cytokines |
| IL17A | CTLA8|IL-17|IL-17A|IL17 | 6 | Cytokines |
| IL17B | IL-17B|IL-20|MGC138900|MGC138901|ZCYTO7 | 5 | Cytokines |
| IL17C | CX2|IL-17C|IL-21|MGC126884|MGC138401 | 16 | Cytokines |
| IL17D | FLJ30846|IL-17D|IL-22|IL-27|IL27 | 13 | Cytokines |
| IL17F | IL-17F|ML-1|ML1 | 6 | Cytokines |
| IL18 | IGIF|IL-18|IL-1g|IL1F4|MGC12320 | 11 | Cytokines |
| IL19 | IL-10C|MDA1|NG.1|ZMDA1 | 1 | Cytokines |
| IL1A | IL-1A|IL1|IL1-ALPHA|IL1F1 | 2 | Cytokines |
| IL1B | IL-1|IL1-BETA|IL1F2 | 2 | Cytokines |
| IL1F10 | FIL1-theta|FKSG75|IL-1HY2|IL1-theta|MGC119831|MGC119832|MGC119833 | 2 | Cytokines |
| IL1F5 | FIL1|FIL1(DELTA)|FIL1D|IL1HY1|IL1L1|IL1RP3|MGC29840 | 2 | Cytokines |
| IL1F6 | FIL1|FIL1(EPSILON)|FIL1E|IL-1F6|IL1(EPSILON)|MGC129552|MGC129553 | 2 | Cytokines |
| IL1F7 | FIL1|FIL1(ZETA)|FIL1Z|IL-1F7|IL-1H4|IL-1RP1|IL1H4|IL1RP1 | 2 | Cytokines |
| IL1F8 | FIL1|FIL1-(ETA)|FIL1H|IL-1F8|IL-1H2|IL1-ETA|IL1H2|MGC126880|MGC126882 | 2 | Cytokines |
| IL1F9 | IL-1F9|IL-1H1|IL-1RP2|IL1E|IL1H1|IL1RP2 | 2 | Cytokines |
| IL1RN | ICIL-1RA|IL-1ra3|IL1F3|IL1RA|IRAP|MGC10430 | 2 | Cytokines |
| IL2 | IL-2|TCGF|lymphokine | 4 | Cytokines |
| IL20 | IL-20|IL10D|MGC96907|ZCYTO10 | 1 | Cytokines |
| IL21 | IL-21|Za11 | 4 | Cytokines |
| IL22 | IL-21|IL-22|IL-D110|IL-TIF|IL21|ILTIF|MGC79382|MGC79384|TIFIL-23|TIFa|zcyto18 | 12 | Cytokines |
| IL23A | IL-23|IL-23A|IL23P19|MGC79388|P19|SGRF | 12 | Cytokines |
| IL24 | C49A|FISP|IL-24|IL10B|MDA7|Mob-5|ST16|mda-7 | 1 | Cytokines |
| IL25 | IL-17E|IL-25|IL17E | 14 | Cytokines |
| IL26 | AK155|IL-26 | 12 | Cytokines |
| IL27 | IL-27|IL-27A|IL27p28|IL30|MGC71873|p28 | 16 | Cytokines |
| IL28A | IFNL2|IL-28A | 19 | Cytokines |
| IL28B | IFNL3|IL-28B|IL28C | 19 | Cytokines |
| IL29 | IFNL1|IL-29 | 19 | Cytokines |
| IL3 | IL-3|MCGF|MGC79398|MGC79399|MULTI-CSF | 5 | Cytokines |
| IL31 | IL-31 | 12 | Cytokines |
| IL32 | IL-32alpha|IL-32beta|IL-32delta|IL-32gamma|NK4|TAIF|TAIFa|TAIFb|TAIFc|TAIFd | 16 | Cytokines |
| IL33 | C9orf26|DKFZp586H0523|DVS27|NF-HEV|NFEHEV|RP11-575C20.2 | 9 | Cytokines |
| IL34 | C16orf77|IL-34|MGC34647 | 16 | Cytokines |
| IL4 | BCGF-1|BCGF1|BSF1|IL-4|MGC79402 | 5 | Cytokines |
| IL5 | EDF|IL-5|TRF | 5 | Cytokines |
| IL6 | BSF2|HGF|HSF|IFNB2|IL-6 | 7 | Cytokines |
| IL6ST | CD130|CDw130|GP130|GP130-RAPS|IL6R-beta | 5 | Cytokines |
| IL7 | IL-7 | 8 | Cytokines |
| IL8 | CXCL8|GCP-1|GCP1|LECT|LUCT|LYNAP|MDNCF|MONAP|NAF|NAP-1|NAP1 | 4 | Cytokines |
| IL9 | HP40|IL-9|P40 | 5 | Cytokines |
| INHA | - | 2 | Cytokines |
| INHBA | EDF|FRP | 7 | Cytokines |
| INHBB | MGC157939 | 2 | Cytokines |
| INHBC | IHBC | 12 | Cytokines |
| INHBE | MGC4638 | 12 | Cytokines |
| INS | ILPR|IRDN | 11 | Cytokines |
| INS-IGF2 | - | 11 | Cytokines |
| INSL3 | MGC119818|MGC119819|RLF|RLNL | 19 | Cytokines |
| INSL4 | EPIL|PLACENTIN | 9 | Cytokines |
| INSL5 | MGC126695|MGC126697|PRO182|UNQ156 | 1 | Cytokines |
| INSL6 | RIF1 | 9 | Cytokines |
| JAG1 | AGS|AHD|AWS|CD339|HJ1|JAGL1|MGC104644 | 20 | Cytokines |
| JAG2 | HJ2|SER2 | 14 | Cytokines |
| KGFLP1 | MGC125746|MGC125747|MGC126891 | 9 | Cytokines |
| KGFLP2 | - | 9 | Cytokines |
| KITLG | DKFZp686F2250|KL-1|Kitl|MGF|SCF|SF|SHEP7 | 12 | Cytokines |
| KL | - | 13 | Cytokines |
| LACRT | MGC71934 | 12 | Cytokines |
| LECT2 | MGC126628|chm-II|chm2 | 5 | Cytokines |
| LEFTY1 | LEFTB|LEFTYB | 1 | Cytokines |
| LEFTY2 | EBAF|LEFTA|LEFTYA|MGC46222|TGFB4 | 1 | Cytokines |
| LEP | FLJ94114|OB|OBS | 7 | Cytokines |
| LHB | CGB4|LSH-B|hLHB | 19 | Cytokines |
| LIF | CDF|DIA|HILDA | 22 | Cytokines |
| LRSAM1 | FLJ31641|RIFLE|TAL | 9 | Cytokines |
| LTA | LT|TNFB|TNFSF1 | 6 | Cytokines |
| LTB | TNFC|TNFSF3|p33 | 6 | Cytokines |
| LTBP1 | MGC163161 | 2 | Cytokines |
| LTBP2 | C14orf141|LTBP3|MSTP031 | 14 | Cytokines |
| LTBP3 | DKFZp586M2123|FLJ33431|FLJ39893|FLJ42533|FLJ44138|FLJ45576|LTBP-3|LTBP2|pp6425 | 11 | Cytokines |
| LTBP4 | FLJ46318|FLJ90018|LTBP-4|LTBP-4L | 19 | Cytokines |
| MDK | FLJ27379|MK|NEGF2 | 11 | Cytokines |
| MIA | CD-RAP | 19 | Cytokines |
| MIF | GIF|GLIF|MMIF | 22 | Cytokines |
| MLN | MGC138519 | 6 | Cytokines |
| MSTN | GDF8 | 2 | Cytokines |
| NAMPT | 1110035O14Rik|DKFZp666B131|MGC117256|PBEF|PBEF1|VF|VISFATIN | 7 | Cytokines |
| NDP | EVR2|FEVR|ND | X | Cytokines |
| NENF | CIR2|NEUDESIN|SCIRP10|SPUF | 1 | Cytokines |
| NGF | Beta-NGF|HSAN5|MGC161426|MGC161428|NGFB | 1 | Cytokines |
| NMB | MGC17211|MGC2277|MGC3936 | 15 | Cytokines |
| NODAL | MGC138230 | 10 | Cytokines |
| NOV | CCN3|IGFBP9 | 8 | Cytokines |
| NPFF | FMRFAL | 12 | Cytokines |
| NPPA | ANF|ANP|ATFB6|CDD-ANF|PND | 1 | Cytokines |
| NPPB | BNP | 1 | Cytokines |
| NPPC | CNP | 2 | Cytokines |
| NPY | PYY4 | 7 | Cytokines |
| NRG1 | ARIA|GGF|GGF2|HGL|HRG|HRG1|HRGA|NDF|SMDF | 8 | Cytokines |
| NRG2 | Don-1|HRG2|NTAK | 5 | Cytokines |
| NRG3 | HRG3|pro-NRG3 | 10 | Cytokines |
| NRG4 | DKFZp779N0541|DKFZp779N1944|HRG4 | 15 | Cytokines |
| NRTN | NTN | 19 | Cytokines |
| NTF3 | HDNF|MGC129711|NGF-2|NGF2|NT3 | 12 | Cytokines |
| NTF4 | NT-4/5|NT4|NT5|NTF5 | 19 | Cytokines |
| NTS | NMN-125|NN|NT|NT/N|NTS1 | 12 | Cytokines |
| NUDT6 | ASFGF2|FGF-2|FGF-AS|FGF2AS|bFGF|gfg|gfg-1 | 4 | Cytokines |
| OGN | DKFZp586P2421|OG|OIF|SLRR3A | 9 | Cytokines |
| OSGIN1 | BDGI|OKL38 | 16 | Cytokines |
| OSM | MGC20461 | 22 | Cytokines |
| OSTN | MUSCLIN | 3 | Cytokines |
| OXT | MGC126890|MGC126892|OT|OT-NPI | 20 | Cytokines |
| P11 | MGC133268|PP11|PRSS26 | 12 | Cytokines |
| PDGFA | PDGF-A|PDGF1 | 7 | Cytokines |
| PDGFB | FLJ12858|PDGF2|SIS|SSV|c-sis | 22 | Cytokines |
| PDGFC | FALLOTEIN|SCDGF | 4 | Cytokines |
| PDGFD | IEGF|MGC26867|MSTP036|SCDGF-B|SCDGFB | 11 | Cytokines |
| PDGFRA | CD140A|MGC74795|PDGFR2|Rhe-PDGFRA | 4 | Cytokines |
| PDGFRB | CD140B|JTK12|PDGF-R-beta|PDGFR|PDGFR1 | 5 | Cytokines |
| PDGFRL | PDGRL|PRLTS | 8 | Cytokines |
| PDYN | MGC26418|PENKB | 20 | Cytokines |
| PENK | - | 8 | Cytokines |
| PF4 | CXCL4|MGC138298|SCYB4 | 4 | Cytokines |
| PF4V1 | CXCL4L1|CXCL4V1|PF4-ALT|PF4A|SCYB4V1 | 4 | Cytokines |
| PGF | D12S1900|PGFL|PLGF|PlGF-2|SHGC-10760 | 14 | Cytokines |
| PLAU | ATF|UPA|URK|u-PA | 10 | Cytokines |
| PMCH | MCH | 12 | Cytokines |
| PNOC | PPNOC | 8 | Cytokines |
| POMC | ACTH|CLIP|LPH|MSH|NPP|POC | 2 | Cytokines |
| PPBP | B-TG1|Beta-TG|CTAP-III|CTAP3|CTAPIII|CXCL7|LA-PF4|LDGF|MDGF|NAP-2|PBP|SCYB7|TC1|TC2|TGB|TGB1|THBGB|THBGB1 | 4 | Cytokines |
| PPBPL1 | TGB2 | 4 | Cytokines |
| PPBPL2 | SPBPBP | 4 | Cytokines |
| PPY | PNP | 17 | Cytokines |
| PRL | - | 6 | Cytokines |
| PRLH | PRH|PRRP | 2 | Cytokines |
| PROK1 | EGVEGF|PK1|PRK1 | 1 | Cytokines |
| PROK2 | BV8|KAL4|MIT1|PK2 | 3 | Cytokines |
| PSPN | PSP | 19 | Cytokines |
| PTH | PTH1 | 11 | Cytokines |
| PTH2 | TIP39 | 19 | Cytokines |
| PTHLH | HHM|MGC14611|PLP|PTHR|PTHRP | 12 | Cytokines |
| PTN | HARP|HBGF8|HBNF|NEGF1 | 7 | Cytokines |
| PYY | PYY1 | 17 | Cytokines |
| QRFP | 26RFa|MGC119794|P518 | 9 | Cytokines |
| RABEP1 | RAB5EP|RABPT5 | 17 | Cytokines |
| RABEP2 | FLJ23282|FRA | 16 | Cytokines |
| REG1A | ICRF|MGC12447|P19|PSP|PSPS|PSPS1|PTP|REG | 2 | Cytokines |
| RETN | ADSF|FIZZ3|MGC126603|MGC126609|RETN1|RSTN|XCP1 | 19 | Cytokines |
| RETNLB | FIZZ1|FIZZ2|HXCP2|RELM-beta|RELMb|RELMbeta|XCP2 | 3 | Cytokines |
| RLN1 | H1|RLXH1|bA12D24.3.1|bA12D24.3.2 | 9 | Cytokines |
| RLN2 | H2|RLXH2|bA12D24.1.1|bA12D24.1.2 | 9 | Cytokines |
| RLN3 | H3|RXN3|ZINS4|insl7 | 19 | Cytokines |
| RNASE2 | EDN|RNS2 | 14 | Cytokines |
| S100A6 | 2A9|5B10|CABP|CACY|PRA | 1 | Cytokines |
| SAA1 | MGC111216|PIG4|SAA|TP53I4 | 11 | Cytokines |
| SAA2 | - | 11 | Cytokines |
| SBDS | CGI-97|FLJ10917|SDS|SWDS | 7 | Cytokines |
| SCG2 | CHGC|SN|SgII | 2 | Cytokines |
| SCGB3A1 | HIN-1|HIN1|LU105|MGC87867|PnSP-2|UGRP2 | 5 | Cytokines |
| SCT | - | 11 | Cytokines |
| SCYE1 | AIMP1|EMAP2|EMAPII|p43 | 4 | Cytokines |
| SECTM1 | K12 | 17 | Cytokines |
| SEMA3A | Hsema-I|Hsema-III|MGC133243|SEMA1|SEMAD|SEMAIII|SEMAL|SemD|coll-1 | 7 | Cytokines |
| SEMA3B | FLJ34863|LUCA-1|SEMA5|SEMAA|SemA|semaV | 3 | Cytokines |
| SEMA3C | SEMAE|SemE | 7 | Cytokines |
| SEMA3D | MGC39708|Sema-Z2|coll-2 | 7 | Cytokines |
| SEMA3E | KIAA0331|M-SEMAH|M-SemaK|SEMAH|coll-5 | 7 | Cytokines |
| SEMA3F | SEMA-IV|SEMA4|SEMAK | 3 | Cytokines |
| SEMA3G | FLJ00014|MGC119473|sem2 | 3 | Cytokines |
| SEMA4A | CORD10|FLJ12287|RP35|SEMAB|SEMB | 1 | Cytokines |
| SEMA4B | KIAA1745|MGC131831|SEMAC|SemC | 15 | Cytokines |
| SEMA4C | FLJ20369|KIAA1739|M-SEMA-F|MGC126382|MGC126383|SEMACL1|SEMAF|SEMAI | 2 | Cytokines |
| SEMA4D | C9orf164|CD100|FLJ33485|FLJ34282|FLJ39737|FLJ46484|M-sema-G|MGC169138|MGC169141|SEMAJ|coll-4 | 9 | Cytokines |
| SEMA4F | M-SEMA|PRO2353|SEMAM|SEMAW|m-Sema-M | 2 | Cytokines |
| SEMA4G | FLJ20590|KIAA1619|MGC102867 | 10 | Cytokines |
| SEMA5A | FLJ12815|SEMAF|semF | 5 | Cytokines |
| SEMA5B | FLJ10372|KIAA1445|SEMAG|SemG | 3 | Cytokines |
| SEMA6A | HT018|KIAA1368|SEMA|SEMA6A1|SEMAQ|VIA | 5 | Cytokines |
| SEMA6B | SEM-SEMA-Y|SEMA-VIB|SEMAN|semaZ | 19 | Cytokines |
| SEMA6C | SEMAY|m-SemaY|m-SemaY2 | 1 | Cytokines |
| SEMA6D | FLJ11598|KIAA1479 | 15 | Cytokines |
| SEMA7A | CD108|CDw108|H-SEMA-K1|H-Sema-L|JMH|MGC126692|MGC126696|SEMAK1|SEMAL | 15 | Cytokines |
| SLIT1 | MEGF4|MGC164811|SLIL1|SLIT3|Slit-1 | 10 | Cytokines |
| SLIT2 | FLJ14420|SLIL3|Slit-2 | 4 | Cytokines |
| SLURP1 | ANUP|ARS|ArsB|LY6LS|MDM | 8 | Cytokines |
| SPP1 | BNSP|BSPI|ETA-1|MGC110940|OPN | 4 | Cytokines |
| SST | SMST | 3 | Cytokines |
| STC1 | STC | 8 | Cytokines |
| STC2 | STC-2|STCRP | 5 | Cytokines |
| TAC1 | Hs.2563|NK2|NKNA|NPK|TAC2 | 7 | Cytokines |
| TDGF1 | CR|CRGF|CRIPTO|Cripto-1 | 3 | Cytokines |
| TDGF3 | CR-3|CRIPTO|CRIPTO-3|TDGF1|TDGF2 | X | Cytokines |
| TG | AITD3|TGN | 8 | Cytokines |
| TGFA | TFGA | 2 | Cytokines |
| TGFB1 | CED|DPD1|TGFB|TGFbeta | 19 | Cytokines |
| TGFB2 | MGC116892|TGF-beta2 | 1 | Cytokines |
| TGFB3 | ARVD|FLJ16571|TGF-beta3 | 14 | Cytokines |
| THPO | MGC163194|MGDF|MKCSF|ML|MPLLG|TPO | 3 | Cytokines |
| TNC | HXB|MGC167029|TN | 9 | Cytokines |
| TNF | DIF|TNF-alpha|TNFA|TNFSF2 | 6 | Cytokines |
| TNFRSF11B | MGC29565|OCIF|OPG|TR1 | 8 | Cytokines |
| TNFSF10 | APO2L|Apo-2L|CD253|TL2|TRAIL | 3 | Cytokines |
| TNFSF11 | CD254|ODF|OPGL|OPTB2|RANKL|TRANCE|hRANKL2|sOdf | 13 | Cytokines |
| TNFSF12 | APO3L|DR3LG|MGC129581|MGC20669|TWEAK | 17 | Cytokines |
| TNFSF13 | APRIL|CD256|TALL2|TRDL-1|UNQ383/PRO715|ligand | 17 | Cytokines |
| TNFSF13B | BAFF|BLYS|CD257|DTL|TALL-1|TALL1|THANK|TNFSF20|ZTNF4 | 13 | Cytokines |
| TNFSF14 | CD258|HVEML|LIGHT|LTg|TR2 | 19 | Cytokines |
| TNFSF15 | MGC129934|MGC129935|TL1|TL1A|VEGI|VEGI192A | 9 | Cytokines |
| TNFSF18 | AITRL|GITRL|MGC138237|TL6|hGITRL | 1 | Cytokines |
| TNFSF4 | CD134L|CD252|GP34|OX-40L|OX4OL|TXGP1 | 1 | Cytokines |
| TNFSF8 | CD153|CD30L|CD30LG|MGC138144 | 9 | Cytokines |
| TNFSF9 | 4-1BB-L|CD137L | 19 | Cytokines |
| TOR2A | FLJ14771|MGC99558|TORP1 | 9 | Cytokines |
| TRH | MGC125964|MGC125965 | 3 | Cytokines |
| TSHB | CHNG4|TSH-BETA | 1 | Cytokines |
| TSLP | - | 5 | Cytokines |
| TXLNA | DKFZp451J0118|IL14|MGC118870|MGC118871|RP4-622L5.4|TXLN | 1 | Cytokines |
| TYMP | ECGF1|MNGIE|PDECGF|TP|hPD-ECGF | 22 | Cytokines |
| UCN | MGC129974|MGC129975|UI|UROC | 2 | Cytokines |
| UCN2 | SRP|UCN-II|UCNI|UR|URP | 3 | Cytokines |
| UCN3 | MGC119002|SCP|SPC|UCNIII | 10 | Cytokines |
| UTS2 | PRO1068|U-II|UCN2|UII | 1 | Cytokines |
| UTS2D | MGC138371|U2B|URP | 3 | Cytokines |
| VEGFA | MGC70609|MVCD1|VEGF|VEGF-A|VPF | 6 | Cytokines |
| VEGFB | VEGFL|VRF | 11 | Cytokines |
| VEGFC | Flt4-L|VRP | 4 | Cytokines |
| VGF | - | 7 | Cytokines |
| VIP | MGC13587|PHM27 | 6 | Cytokines |
| XCL1 | ATAC|LPTN|LTN|SCM-1|SCM-1a|SCM1|SCYC1 | 1 | Cytokines |
| XCL2 | SCM-1b|SCM1B|SCYC2 | 1 | Cytokines |
| ACVR1B | ACTRIB|ACVRLK4|ALK4|SKR2 | 12 | Cytokine_Receptors |
| ACVR1C | ACVRLK7|ALK7 | 2 | Cytokine_Receptors |
| ACVR2A | ACTRII|ACVR2 | 2 | Cytokine_Receptors |
| ACVR2B | ACTRIIB|ActR-IIB|MGC116908 | 3 | Cytokine_Receptors |
| ACVRL1 | ACVRLK1|ALK-1|ALK1|HHT|HHT2|ORW2|SKR3|TSR-I | 12 | Cytokine_Receptors |
| ADCYAP1R1 | PAC1|PACAPR|PACAPRI | 7 | Cytokine_Receptors |
| ADIPOR1 | ACDCR1|CGI-45|CGI45|FLJ25385|FLJ42464|PAQR1|TESBP1A | 1 | Cytokine_Receptors |
| ADIPOR2 | ACDCR2|FLJ21432|MGC4640|PAQR2 | 12 | Cytokine_Receptors |
| ADRB1 | ADRB1R|B1AR|BETA1AR|RHR | 10 | Cytokine_Receptors |
| ADRB2 | ADRB2R|ADRBR|B2AR|BAR|BETA2AR | 5 | Cytokine_Receptors |
| AGTR1 | AG2S|AGTR1A|AGTR1B|AT1|AT1B|AT1R|AT2R1|AT2R1A|AT2R1B|HAT1R | 3 | Cytokine_Receptors |
| AGTR2 | AT2|ATGR2|MRX88 | X | Cytokine_Receptors |
| AMHR2 | AMHR|MISR2|MISRII | 12 | Cytokine_Receptors |
| ANGPT1 | AGP1|AGPT|ANG1 | 8 | Cytokine_Receptors |
| ANGPT4 | AGP4|ANG-3|ANG4|MGC138181|MGC138183 | 20 | Cytokine_Receptors |
| ANGPTL1 | ANG3|ANGPT3|ARP1|AngY|KIAA0351|UNQ162|dJ595C2.2 | 1 | Cytokine_Receptors |
| ANGPTL2 | ARP2|HARP|MGC8889 | 9 | Cytokine_Receptors |
| ANGPTL3 | ANGPT5 | 1 | Cytokine_Receptors |
| ANGPTL4 | ANGPTL2|ARP4|FIAF|HFARP|NL2|PGAR|pp1158 | 19 | Cytokine_Receptors |
| ANGPTL6 | AGF|ARP5 | 19 | Cytokine_Receptors |
| APLNR | AGTRL1|APJ|APJR|FLJ90771|MGC45246 | 11 | Cytokine_Receptors |
| AR | AIS|DHTR|HUMARA|HYSP1|KD|NR3C4|SBMA|SMAX1|TFM | X | Cytokine_Receptors |
| AVPR1A | AVPR1 | 12 | Cytokine_Receptors |
| AVPR1B | AVPR3 | 1 | Cytokine_Receptors |
| AVPR2 | ADHR|DI1|DIR|DIR3|MGC126533|MGC138386|NDI|V2R | X | Cytokine_Receptors |
| BMPR1A | 10q23del|ACVRLK3|ALK3|CD292|SKR5 | 10 | Cytokine_Receptors |
| BMPR1B | ALK-6|ALK6|CDw293 | 4 | Cytokine_Receptors |
| BMPR2 | BMPR-II|BMPR3|BMR2|BRK-3|FLJ41585|FLJ76945|PPH1|T-ALK | 2 | Cytokine_Receptors |
| BRD8 | SMAP|SMAP2|p120 | 5 | Cytokine_Receptors |
| C3AR1 | AZ3B|C3AR|HNFAG09 | 12 | Cytokine_Receptors |
| C5AR1 | C5A|C5AR|C5R1|CD88 | 19 | Cytokine_Receptors |
| CALCR | CRT|CTR|CTR1 | 7 | Cytokine_Receptors |
| CALCRL | CGRPR|CRLR | 2 | Cytokine_Receptors |
| CCBP2 | CCR10|CCR9|CMKBR9|D6|MGC126678|MGC138250|hD6 | 3 | Cytokine_Receptors |
| CCR1 | CD191|CKR-1|CKR1|CMKBR1|HM145|MIP1aR|SCYAR1 | 3 | Cytokine_Receptors |
| CCR10 | GPR2 | 17 | Cytokine_Receptors |
| CCR3 | CC-CKR-3|CD193|CKR3|CMKBR3|MGC102841 | 3 | Cytokine_Receptors |
| CCR4 | CC-CKR-4|CD194|CKR4|CMKBR4|ChemR13|HGCN:14099|K5-5|MGC88293 | 3 | Cytokine_Receptors |
| CCR5 | CC-CKR-5|CCCKR5|CD195|CKR-5|CKR5|CMKBR5|FLJ78003|IDDM22 | 3 | Cytokine_Receptors |
| CCR6 | BN-1|CD196|CKR-L3|CKR6|CKRL3|CMKBR6|DCR2|DRY-6|GPR-CY4|GPR29|GPRCY4|STRL22 | 6 | Cytokine_Receptors |
| CCR7 | BLR2|CD197|CDw197|CMKBR7|EBI1 | 17 | Cytokine_Receptors |
| CCR8 | CDw198|CKR-L1|CKRL1|CMKBR8|CMKBRL2|CY6|GPR-CY6|MGC129966|MGC129973|TER1 | 3 | Cytokine_Receptors |
| CCR9 | CDw199|GPR-9-6|GPR28 | 3 | Cytokine_Receptors |
| CCRL1 | CC-CKR-11|CCBP2|CCR10|CCR11|CCX-CKR|CKR-11|PPR1|VSHK1 | 3 | Cytokine_Receptors |
| CCRL2 | CKRX|CRAM-A|CRAM-B|FLJ55815|HCR|MGC116710|MGC34104 | 3 | Cytokine_Receptors |
| CD40 | Bp50|CDW40|MGC9013|TNFRSF5|p50 | 20 | Cytokine_Receptors |
| CMKLR1 | CHEMERINR|ChemR23|DEZ|MGC126105|MGC126106 | 12 | Cytokine_Receptors |
| CNTFR | MGC1774 | 9 | Cytokine_Receptors |
| CRHR1 | CRF-R|CRF1|CRFR1|CRH-R1h|CRHR|CRHR1f | 17 | Cytokine_Receptors |
| CRHR2 | CRFR2 | 7 | Cytokine_Receptors |
| CRIM1 | MGC138194|S52 | 2 | Cytokine_Receptors |
| CRLF1 | CISS|CISS1|CLF|CLF-1|NR6 | 19 | Cytokine_Receptors |
| CRLF2 | CRL2|CRLF2Y|TSLPR | X|Y | Cytokine_Receptors |
| CRLF3 | CREME9|CYTOR4|FRWS|MGC20661 | 17 | Cytokine_Receptors |
| CSF1R | C-FMS|CD115|CSFR|FIM2|FMS | 5 | Cytokine_Receptors |
| CSF2RA | CD116|CDw116|CSF2R|CSF2RAX|CSF2RAY|CSF2RX|CSF2RY|GM-CSF-R-alpha|GMCSFR|GMR|MGC3848|MGC4838 | X|Y | Cytokine_Receptors |
| CSF2RB | CD131|CDw131|IL3RB|IL5RB | 22 | Cytokine_Receptors |
| CSF3R | CD114|GCSFR | 1 | Cytokine_Receptors |
| CX3CR1 | CCRL1|CMKBRL1|CMKDR1|GPR13|GPRV28|V28 | 3 | Cytokine_Receptors |
| CXCR3 | CD182|CD183|CKR-L2|CMKAR3|GPR9|IP10-R|Mig-R|MigR | X | Cytokine_Receptors |
| CXCR4 | CD184|D2S201E|FB22|HM89|HSY3RR|LAP3|LCR1|LESTR|NPY3R|NPYR|NPYRL|NPYY3R|WHIM | 2 | Cytokine_Receptors |
| CXCR5 | BLR1|CD185|MDR15|MGC117347 | 11 | Cytokine_Receptors |
| CXCR6 | BONZO|CD186|STRL33|TYMSTR | 3 | Cytokine_Receptors |
| CXCR7 | CMKOR1|GPR159|RDC1 | 2 | Cytokine_Receptors |
| CYSLTR1 | CYSLT1|CYSLT1R|CYSLTR|HG55|HMTMF81|MGC46139 | X | Cytokine_Receptors |
| CYSLTR2 | CYSLT2|CYSLT2R|GPCR|HG57|HPN321|KPG_011|PSEC0146|hGPCR21 | 13 | Cytokine_Receptors |
| DARC | CCBP1|CD234|Dfy|FY|GPD|GpFy|WBCQ1 | 1 | Cytokine_Receptors |
| EDNRA | ETA|ETRA | 4 | Cytokine_Receptors |
| EDNRB | ABCDS|ETB|ETBR|ETRB|HSCR|HSCR2 | 13 | Cytokine_Receptors |
| EGFR | ERBB|ERBB1|HER1|PIG61|mENA | 7 | Cytokine_Receptors |
| ENG | CD105|END|FLJ41744|HHT1|ORW|ORW1 | 9 | Cytokine_Receptors |
| EPOR | MGC138358 | 19 | Cytokine_Receptors |
| ESR1 | DKFZp686N23123|ER|ESR|ESRA|Era|NR3A1 | 6 | Cytokine_Receptors |
| ESR2 | ER-BETA|ESR-BETA|ESRB|ESTRB|Erb|NR3A2 | 14 | Cytokine_Receptors |
| ESRRA | ERR1|ERRa|ERRalpha|ESRL1|NR3B1 | 11 | Cytokine_Receptors |
| ESRRB | DFNB35|ERR2|ERRb|ERRbeta|ERRbeta-2|ESRL2|NR3B2 | 14 | Cytokine_Receptors |
| ESRRG | DKFZp781L1617|ERR3|FLJ16023|KIAA0832|NR3B3 | 1 | Cytokine_Receptors |
| FGFR1 | BFGFR|CD331|CEK|FGFBR|FLG|FLJ99988|FLT2|HBGFR|KAL2|N-SAM|OGD | 8 | Cytokine_Receptors |
| FGFR2 | BEK|BFR-1|CD332|CEK3|CFD1|ECT1|FLJ98662|JWS|K-SAM|KGFR|TK14|TK25 | 10 | Cytokine_Receptors |
| FGFR3 | ACH|CD333|CEK2|HSFGFR3EX|JTK4 | 4 | Cytokine_Receptors |
| FGFR4 | CD334|JTK2|MGC20292|TKF | 5 | Cytokine_Receptors |
| FGFRL1 | FGFR5|FHFR | 4 | Cytokine_Receptors |
| FLT1 | FLT|VEGFR1 | 13 | Cytokine_Receptors |
| FLT3 | CD135|FLK2|STK1 | 13 | Cytokine_Receptors |
| FLT4 | FLT41|LMPH1A|PCL|VEGFR3 | 5 | Cytokine_Receptors |
| FPR1 | FMLP|FPR | 19 | Cytokine_Receptors |
| FPR2 | ALXR|FMLP-R-II|FMLPX|FPR2A|FPRH1|FPRH2|FPRL1|HM63|LXA4R | 19 | Cytokine_Receptors |
| FPR2 | ALXR|FMLP-R-II|FMLPX|FPR2A|FPRH1|FPRH2|FPRL1|HM63|LXA4R | 19 | Cytokine_Receptors |
| FSHR | FSHRO|LGR1|MGC141667|MGC141668|ODG1 | 2 | Cytokine_Receptors |
| GALR2 | GALNR2|MGC125983|MGC125984 | 17 | Cytokine_Receptors |
| GALR3 | - | 22 | Cytokine_Receptors |
| GCGR | GGR|MGC138246 | 17 | Cytokine_Receptors |
| GHR | GHBP | 5 | Cytokine_Receptors |
| GHRHR | GHRFR|GHRHRpsv|GRFR | 7 | Cytokine_Receptors |
| GHSR | - | 3 | Cytokine_Receptors |
| GIPR | MGC126722 | 19 | Cytokine_Receptors |
| GLP1R | MGC138331 | 6 | Cytokine_Receptors |
| GLP2R | - | 17 | Cytokine_Receptors |
| GNRHR | GNRHR1|GRHR|LHRHR|LRHR | 4 | Cytokine_Receptors |
| GPER | CEPR|CMKRL2|DRY12|FEG-1|GPCR-Br|GPR30|LERGU|LERGU2|LyGPR|MGC99678 | 7 | Cytokine_Receptors |
| GPR17 | DKFZp686M18273 | 2 | Cytokine_Receptors |
| GPR32 | - | 19 | Cytokine_Receptors |
| GPR33 | - | 14 | Cytokine_Receptors |
| GPR44 | CD294|CRTH2|DP2 | 11 | Cytokine_Receptors |
| GPR77 | C5L2|GPF77 | 19 | Cytokine_Receptors |
| HNF4A | FLJ39654|HNF4|HNF4a7|HNF4a8|HNF4a9|HNF4alpha|MODY|MODY1|NR2A1|NR2A21|TCF|TCF14 | 20 | Cytokine_Receptors |
| HNF4G | NR2A2|NR2A3 | 8 | Cytokine_Receptors |
| HTR3A | 5-HT-3|5-HT3A|5-HT3R|5HT3R|HTR3 | 11 | Cytokine_Receptors |
| HTR3B | 5-HT3B | 11 | Cytokine_Receptors |
| HTR3C | - | 3 | Cytokine_Receptors |
| HTR3D | MGC119636|MGC119637 | 3 | Cytokine_Receptors |
| HTR3E | 5-HT3c1|MGC120035|MGC120036|MGC120037 | 3 | Cytokine_Receptors |
| IFNAR1 | AVP|IFN-alpha-REC|IFNAR|IFNBR|IFRC | 21 | Cytokine_Receptors |
| IFNAR2 | IFN-R|IFN-alpha-REC|IFNABR|IFNARB | 21 | Cytokine_Receptors |
| IFNGR1 | CD119|FLJ45734|IFNGR | 6 | Cytokine_Receptors |
| IFNGR2 | AF-1|IFGR2|IFNGT1 | 21 | Cytokine_Receptors |
| IGF1R | CD221|IGFIR|IGFR|JTK13|MGC142170|MGC142172|MGC18216 | 15 | Cytokine_Receptors |
| IGF2R | CD222|CIMPR|M6P-R|MPR1|MPRI | 6 | Cytokine_Receptors |
| IL10RA | CDW210A|HIL-10R|IL-10R1|IL10R | 11 | Cytokine_Receptors |
| IL10RB | CDW210B|CRF2-4|CRFB4|D21S58|D21S66|IL-10R2 | 21 | Cytokine_Receptors |
| IL11RA | MGC2146 | 9 | Cytokine_Receptors |
| IL11RB | - | - | Cytokine_Receptors |
| IL12RB1 | CD212|IL-12R-BETA1|IL12RB|MGC34454 | 19 | Cytokine_Receptors |
| IL12RB2 | - | 1 | Cytokine_Receptors |
| IL13RA1 | CD213A1|IL-13Ra|NR4 | X | Cytokine_Receptors |
| IL13RA2 | CD213A2|CT19|IL-13R|IL13BP | X | Cytokine_Receptors |
| IL15RA | MGC104179 | 10 | Cytokine_Receptors |
| IL15RB | - | - | Cytokine_Receptors |
| IL17RA | CD217|CDw217|IL-17RA|IL17R|MGC10262|hIL-17R | 22 | Cytokine_Receptors |
| IL17RB | CRL4|EVI27|IL17BR|IL17RH1|MGC5245 | 3 | Cytokine_Receptors |
| IL17RC | FLJ95963|FLJ96005|IL17-RL|IL17RL|MGC10763 | 3 | Cytokine_Receptors |
| IL17RD | DKFZp434N1928|FLJ35755|IL-17RD|IL17RLM|MGC133309|SEF | 3 | Cytokine_Receptors |
| IL17RE | FLJ23658|MGC71884 | 3 | Cytokine_Receptors |
| IL18R1 | CD218a|CDw218a|IL-1Rrp|IL18RA|IL1RRP | 2 | Cytokine_Receptors |
| IL18RAP | ACPL|CD218b|CDw218b|IL18RB|MGC120589|MGC120590 | 2 | Cytokine_Receptors |
| IL1R1 | CD121A|D2S1473|IL-1R-alpha|IL1R|IL1RA|P80 | 2 | Cytokine_Receptors |
| IL1R2 | CD121b|IL1RB|MGC47725 | 2 | Cytokine_Receptors |
| IL1RAP | C3orf13|FLJ37788|IL-1RAcP|IL1R3 | 3 | Cytokine_Receptors |
| IL1RL1 | DER4|FIT-1|MGC32623|ST2|ST2L|ST2V|T1 | 2 | Cytokine_Receptors |
| IL1RL2 | IL1R-rp2|IL1RRP2 | 2 | Cytokine_Receptors |
| IL20RA | FLJ40993|IL-20R1|ZCYTOR7 | 6 | Cytokine_Receptors |
| IL20RB | DIRS1|FNDC6|IL-20R2|MGC34923 | 3 | Cytokine_Receptors |
| IL21R | MGC10967|NILR | 16 | Cytokine_Receptors |
| IL22RA1 | CRF2-9|IL22R|IL22R1 | 1 | Cytokine_Receptors |
| IL22RA2 | CRF2-10|CRF2-S1|CRF2X|IL-22BP|MGC150509|MGC150510 | 6 | Cytokine_Receptors |
| IL23R | - | 1 | Cytokine_Receptors |
| IL27RA | CRL1|IL27R|TCCR|WSX1|zcytor1 | 19 | Cytokine_Receptors |
| IL28RA | CRF2/12|IFNLR|IFNLR1|IL-28R1|LICR2 | 1 | Cytokine_Receptors |
| IL2RA | CD25|IDDM10|IL2R|TCGFR | 10 | Cytokine_Receptors |
| IL2RB | CD122|P70-75 | 22 | Cytokine_Receptors |
| IL2RG | CD132|IMD4|SCIDX|SCIDX1 | X | Cytokine_Receptors |
| IL31RA | CRL|CRL3|GLM-R|GLMR|GPL|IL-31RA|MGC125346|PRO21384 | 5 | Cytokine_Receptors |
| IL3RA | CD123|IL3R|IL3RAY|IL3RX|IL3RY|MGC34174|hIL-3Ra | X|Y | Cytokine_Receptors |
| IL4R | CD124|IL4RA | 16 | Cytokine_Receptors |
| IL5RA | CD125|CDw125|HSIL5R3|IL5R|MGC26560 | 3 | Cytokine_Receptors |
| IL6R | CD126|IL-6R-1|IL-6R-alpha|IL6RA|MGC104991 | 1 | Cytokine_Receptors |
| IL7R | CD127|CDW127|IL-7R-alpha|IL7RA|ILRA | 5 | Cytokine_Receptors |
| IL8RA | C-C|C-C-CKR-1|CD128|CD181|CDw128a|CKR-1|CMKAR1|CXCR1|IL8R1|IL8RBA | 2 | Cytokine_Receptors |
| IL8RB | CD182|CDw128b|CMKAR2|CXCR2|IL8R2|IL8RA | 2 | Cytokine_Receptors |
| IL9R | CD129 | X|Y | Cytokine_Receptors |
| INSR | CD220|HHF5 | 19 | Cytokine_Receptors |
| KDR | CD309|FLK1|VEGFR|VEGFR2 | 4 | Cytokine_Receptors |
| LEPR | CD295|OBR | 1 | Cytokine_Receptors |
| LGR4 | GPR48 | 11 | Cytokine_Receptors |
| LGR5 | FEX|GPR49|GPR67|GRP49|HG38|MGC117008 | 12 | Cytokine_Receptors |
| LGR6 | FLJ14471|GPCR|VTS20631 | 1 | Cytokine_Receptors |
| LHCGR | FLJ41504|LCGR|LGR2|LH/CG-R|LH/CGR|LHR|LHRHR|LSH-R | 2 | Cytokine_Receptors |
| LIFR | CD118|FLJ98106|FLJ99923|LIF-R|SJS2|STWS|SWS | 5 | Cytokine_Receptors |
| LTB4R | BLT1|BLTR|CMKRL1|GPR16|LTB4R1|LTBR1|P2RY7|P2Y7 | 14 | Cytokine_Receptors |
| LTB4R2 | BLT2|BLTR2|JULF2|KPG_004|NOP9 | 14 | Cytokine_Receptors |
| LTBR | CD18|D12S370|LT-BETA-R|TNF-R-III|TNFCR|TNFR-RP|TNFR2-RP|TNFRSF3 | 12 | Cytokine_Receptors |
| MC1R | MGC14337|MSH-R|SHEP2 | 16 | Cytokine_Receptors |
| MC2R | ACTHR|MGC125798 | 18 | Cytokine_Receptors |
| MC3R | BMIQ9|MC3|MC3-R|OB20|OQTL | 20 | Cytokine_Receptors |
| MC4R | MGC126851|MGC138197 | 18 | Cytokine_Receptors |
| MCHR1 | GPR24|MCH1R|MGC32129|SLC1 | 22 | Cytokine_Receptors |
| MCHR2 | GPR145|MCH2|MCH2R|SLT | 6 | Cytokine_Receptors |
| MET | AUTS9|HGFR|RCCP2|c-Met | 7 | Cytokine_Receptors |
| MLNR | GPR38|MTLR1 | 13 | Cytokine_Receptors |
| MPL | C-MPL|CD110|MPLV|TPOR | 1 | Cytokine_Receptors |
| MTNR1A | MEL-1A-R|MT1 | 4 | Cytokine_Receptors |
| MTNR1B | MEL-1B-R|MT2 | 11 | Cytokine_Receptors |
| NGFR | CD271|Gp80-LNGFR|TNFRSF16|p75(NTR)|p75NTR | 17 | Cytokine_Receptors |
| NMBR | - | 6 | Cytokine_Receptors |
| NPR1 | ANPRA|ANPa|GUC2A|GUCY2A|NPRA | 1 | Cytokine_Receptors |
| NPR3 | ANPRC|GUCY2B|NPRC | 5 | Cytokine_Receptors |
| NR0B1 | AHC|AHCH|AHX|DAX-1|DAX1|DSS|GTD|HHG|NROB1 | X | Cytokine_Receptors |
| NR0B2 | FLJ17090|SHP|SHP1 | 1 | Cytokine_Receptors |
| NR1D1 | EAR1|THRA1|THRAL|ear-1|hRev | 17 | Cytokine_Receptors |
| NR1D2 | BD73|EAR-1R|RVR | 3 | Cytokine_Receptors |
| NR1H2 | LXR-b|LXRB|NER|NER-I|RIP15|UNR | 19 | Cytokine_Receptors |
| NR1H3 | LXR-a|LXRA|RLD-1 | 11 | Cytokine_Receptors |
| NR1H4 | BAR|FXR|HRR-1|HRR1|MGC163445|RIP14 | 12 | Cytokine_Receptors |
| NR1I2 | BXR|ONR1|PAR|PAR1|PAR2|PARq|PRR|PXR|SAR|SXR | 3 | Cytokine_Receptors |
| NR1I3 | CAR|CAR1|MB67|MGC150433|MGC97144|MGC97209 | 1 | Cytokine_Receptors |
| NR2C1 | TR2 | 12 | Cytokine_Receptors |
| NR2C2 | TAK1|TR2R1|TR4|hTAK1 | 3 | Cytokine_Receptors |
| NR2E1 | TLL|TLX|XTLL | 6 | Cytokine_Receptors |
| NR2E3 | ESCS|MGC49976|PNR|RNR|RP37|rd7 | 15 | Cytokine_Receptors |
| NR2F1 | COUP-TFI|EAR-3|EAR3|ERBAL3|NR2F2|SVP44|TCFCOUP1|TFCOUP1 | 5 | Cytokine_Receptors |
| NR2F2 | ARP1|COUP-TFII|COUPTFB|MGC117452|SVP40|TFCOUP2 | 15 | Cytokine_Receptors |
| NR2F6 | EAR-2|EAR2|ERBAL2 | 19 | Cytokine_Receptors |
| NR3C1 | GCCR|GCR|GR|GRL | 5 | Cytokine_Receptors |
| NR3C2 | MCR|MGC133092|MLR|MR | 4 | Cytokine_Receptors |
| NR4A1 | GFRP1|HMR|MGC9485|N10|NAK-1|NGFIB|NP10|NUR77|TR3 | 12 | Cytokine_Receptors |
| NR4A2 | HZF-3|NOT|NURR1|RNR1|TINUR | 2 | Cytokine_Receptors |
| NR4A3 | CHN|CSMF|MINOR|NOR1|TEC | 9 | Cytokine_Receptors |
| NR5A1 | AD4BP|ELP|FTZ1|FTZF1|SF-1|SF1 | 9 | Cytokine_Receptors |
| NR5A2 | B1F|B1F2|CPF|FTF|FTZ-F1|FTZ-F1beta|LRH-1|hB1F|hB1F-2 | 1 | Cytokine_Receptors |
| NR6A1 | GCNF|GCNF1|NR61|RTR | 9 | Cytokine_Receptors |
| NRP1 | BDCA4|CD304|DKFZp686A03134|DKFZp781F1414|NP1|NRP|VEGF165R | 10 | Cytokine_Receptors |
| NRP2 | MGC126574|NP2|NPN2|PRO2714|VEGF165R2 | 2 | Cytokine_Receptors |
| OGFR | - | 20 | Cytokine_Receptors |
| OPRD1 | OPRD | 1 | Cytokine_Receptors |
| OPRK1 | KOR|OPRK | 8 | Cytokine_Receptors |
| OPRL1 | KOR-3|MGC34578|NOCIR|OOR|ORL1 | 20 | Cytokine_Receptors |
| OPRM1 | KIAA0403|LMOR|MOR|MOR1|OPRM | 6 | Cytokine_Receptors |
| OSMR | MGC150626|MGC150627|MGC75127|OSMRB | 5 | Cytokine_Receptors |
| OXTR | OT-R | 3 | Cytokine_Receptors |
| PGR | NR3C3|PR | 11 | Cytokine_Receptors |
| PGRMC2 | DG6|PMBP | 4 | Cytokine_Receptors |
| PLAUR | CD87|UPAR|URKR | 19 | Cytokine_Receptors |
| PLXNA1 | NOV|NOVP|PLEXIN-A1|PLXN1 | 3 | Cytokine_Receptors |
| PLXNA2 | FLJ11751|FLJ30634|KIAA0463|OCT|PLXN2 | 1 | Cytokine_Receptors |
| PLXNA3 | 6.3|HSSEXGENE|PLEXIN-A3|PLXN3|PLXN4|SEX|XAP-6 | X | Cytokine_Receptors |
| PLXNA4 | DKFZp434G0625|DKFZp566O0546|FAYV2820|FLJ35026|FLJ38287|KIAA1550|PLEXA4|PLXNA4A|PLXNA4B|PRO34003 | 7 | Cytokine_Receptors |
| PLXNB1 | KIAA0407|MGC149167|PLEXIN-B1|PLXN5|SEP | 3 | Cytokine_Receptors |
| PLXNB2 | KIAA0315|MM1|Nbla00445|PLEXB2|dJ402G11.3 | 22 | Cytokine_Receptors |
| PLXNB3 | FLJ76953|PLEXB3|PLEXR|PLXN6 | X | Cytokine_Receptors |
| PLXNC1 | CD232|PLXN-C1|VESPR | 12 | Cytokine_Receptors |
| PLXND1 | KIAA0620|MGC75353|PLEXD1 | 3 | Cytokine_Receptors |
| PPARA | MGC2237|MGC2452|NR1C1|PPAR|hPPAR | 22 | Cytokine_Receptors |
| PPARD | FAAR|MGC3931|NR1C2|NUC1|NUCI|NUCII|PPAR-beta|PPARB | 6 | Cytokine_Receptors |
| PPARG | CIMT1|NR1C3|PPARG1|PPARG2|PPARgamma | 3 | Cytokine_Receptors |
| PRLHR | GPR10|GR3|MGC126539|MGC126541|PrRPR | 10 | Cytokine_Receptors |
| PRLR | hPRLrI | 5 | Cytokine_Receptors |
| PTAFR | PAFR | 1 | Cytokine_Receptors |
| PTGDR | AS1|ASRT1|DP|DP1|MGC49004 | 14 | Cytokine_Receptors |
| PTGDS | LPGDS|PDS|PGD2|PGDS|PGDS2 | 9 | Cytokine_Receptors |
| PTGER1 | EP1 | 19 | Cytokine_Receptors |
| PTGER2 | EP2 | 14 | Cytokine_Receptors |
| PTGER3 | EP3|EP3-I|EP3-II|EP3-III|EP3-IV|EP3e|MGC141828|MGC141829|MGC27302 | 1 | Cytokine_Receptors |
| PTGER4 | EP4|EP4R|MGC126583 | 5 | Cytokine_Receptors |
| PTGFR | FP|MGC120498|MGC46203 | 1 | Cytokine_Receptors |
| PTH1R | MGC138426|MGC138452|PFE|PTHR|PTHR1 | 3 | Cytokine_Receptors |
| PTH2R | PTHR2 | 2 | Cytokine_Receptors |
| RARA | NR1B1|RAR | 17 | Cytokine_Receptors |
| RARB | HAP|NR1B2|RRB2 | 3 | Cytokine_Receptors |
| RARG | NR1B3|RARC | 12 | Cytokine_Receptors |
| ROBO1 | DUTT1|FLJ21882|MGC131599|MGC133277|SAX3 | 3 | Cytokine_Receptors |
| ROBO2 | KIAA1568|SAX3 | 3 | Cytokine_Receptors |
| ROBO3 | FLJ21044|HGPPS|HGPS|RBIG1|RIG1 | 11 | Cytokine_Receptors |
| RORA | DKFZp686M2414|MGC119326|MGC119329|NR1F1|ROR1|ROR2|ROR3|RZR-ALPHA|RZRA | 15 | Cytokine_Receptors |
| RORB | NR1F2|ROR-BETA|RZR-BETA|RZRB|bA133M9.1 | 9 | Cytokine_Receptors |
| RORC | MGC129539|NR1F3|RORG|RZR-GAMMA|RZRG|TOR | 1 | Cytokine_Receptors |
| RXFP1 | LGR7|LGR7.1|LGR7.10|LGR7.2|MGC138347|MGC142177|RXFPR1 | 4 | Cytokine_Receptors |
| RXFP2 | GPR106|GREAT|INSL3R|LGR8|LGR8.1|RXFPR2 | 13 | Cytokine_Receptors |
| RXFP3 | GPCR135|MGC141998|MGC142000|RLN3R1|RXFPR3|SALPR | 5 | Cytokine_Receptors |
| RXRA | FLJ00280|FLJ00318|FLJ16020|FLJ16733|MGC102720|NR2B1 | 9 | Cytokine_Receptors |
| RXRB | DAUDI6|H-2RIIBP|MGC1831|NR2B2|RCoR-1 | 6 | Cytokine_Receptors |
| RXRG | NR2B3|RXRC | 1 | Cytokine_Receptors |
| S1PR1 | CHEDG1|D1S3362|ECGF1|EDG-1|EDG1|FLJ58121|S1P1 | 1 | Cytokine_Receptors |
| S1PR2 | AGR16|EDG-5|EDG5|Gpcr13|H218|LPB2|S1P2 | 19 | Cytokine_Receptors |
| SCTR | SR | 2 | Cytokine_Receptors |
| SDC1 | CD138|SDC|SYND1|syndecan | 2 | Cytokine_Receptors |
| SDC2 | HSPG|HSPG1|SYND2 | 8 | Cytokine_Receptors |
| SDC3 | N-syndecan|SDCN|SYND3 | 1 | Cytokine_Receptors |
| SDC4 | MGC22217|SYND4 | 20 | Cytokine_Receptors |
| SORT1 | Gp95|NT3 | 1 | Cytokine_Receptors |
| SSTR1 | SRIF-2 | 14 | Cytokine_Receptors |
| SSTR2 | - | 17 | Cytokine_Receptors |
| SSTR5 | - | 16 | Cytokine_Receptors |
| ST2 | - | 11 | Cytokine_Receptors |
| TACR1 | NK1R|NKIR|SPR|TAC1R | 2 | Cytokine_Receptors |
| TEK | CD202B|TIE-2|TIE2|VMCM|VMCM1 | 9 | Cytokine_Receptors |
| TGFBR1 | AAT5|ACVRLK4|ALK-5|ALK5|LDS1A|LDS2A|SKR4|TGFR-1 | 9 | Cytokine_Receptors |
| TGFBR2 | AAT3|FAA3|LDS1B|LDS2B|MFS2|RIIC|TAAD2|TGFR-2|TGFbeta-RII | 3 | Cytokine_Receptors |
| TGFBR3 | BGCAN|betaglycan | 1 | Cytokine_Receptors |
| THRA | AR7|EAR7|ERB-T-1|ERBA|ERBA1|MGC000261|MGC43240|NR1A1|THRA1|THRA2|c-ERBA-1 | 17 | Cytokine_Receptors |
| THRB | ERBA-BETA|ERBA2|GRTH|MGC126109|MGC126110|NR1A2|PRTH|THR1|THRB1|THRB2 | 3 | Cytokine_Receptors |
| TIE1 | JTK14|TIE | 1 | Cytokine_Receptors |
| TNFRSF10A | APO2|CD261|DR4|MGC9365|TRAILR-1|TRAILR1 | 8 | Cytokine_Receptors |
| TNFRSF10B | CD262|DR5|KILLER|KILLER/DR5|TRAIL-R2|TRAILR2|TRICK2|TRICK2A|TRICK2B|TRICKB|ZTNFR9 | 8 | Cytokine_Receptors |
| TNFRSF10C | CD263|DCR1|LIT|MGC149501|MGC149502|TRAILR3|TRID | 8 | Cytokine_Receptors |
| TNFRSF10D | CD264|DCR2|TRAILR4|TRUNDD | 8 | Cytokine_Receptors |
| TNFRSF11A | CD265|FEO|LOH18CR1|ODFR|OFE|OPTB7|OSTS|PDB2|RANK|TRANCER | 18 | Cytokine_Receptors |
| TNFRSF12A | CD266|FN14|TWEAKR | 16 | Cytokine_Receptors |
| TNFRSF13B | CD267|CVID|FLJ39942|MGC133214|MGC39952|TACI|TNFRSF14B | 17 | Cytokine_Receptors |
| TNFRSF13C | BAFF-R|BAFFR|CD268|MGC138235 | 22 | Cytokine_Receptors |
| TNFRSF14 | ATAR|HVEA|HVEM|LIGHTR|TR2 | 1 | Cytokine_Receptors |
| TNFRSF17 | BCM|BCMA|CD269 | 16 | Cytokine_Receptors |
| TNFRSF18 | AITR|GITR|GITR-D | 1 | Cytokine_Receptors |
| TNFRSF19 | TAJ|TAJ-alpha|TRADE|TROY | 13 | Cytokine_Receptors |
| TNFRSF1A | CD120a|FPF|MGC19588|TBP1|TNF-R|TNF-R-I|TNF-R55|TNFAR|TNFR1|TNFR55|TNFR60|p55|p55-R|p60 | 12 | Cytokine_Receptors |
| TNFRSF1B | CD120b|TBPII|TNF-R-II|TNF-R75|TNFBR|TNFR1B|TNFR2|TNFR80|p75|p75TNFR | 1 | Cytokine_Receptors |
| TNFRSF21 | BM-018|DR6|MGC31965 | 6 | Cytokine_Receptors |
| TNFRSF25 | APO-3|DDR3|DR3|LARD|TNFRSF12|TR3|TRAMP|WSL-1|WSL-LR | 1 | Cytokine_Receptors |
| TNFRSF4 | ACT35|CD134|OX40|TXGP1L | 1 | Cytokine_Receptors |
| TNFRSF6B | DCR3|DJ583P15.1.1|M68|TR6 | 20 | Cytokine_Receptors |
| TNFRSF8 | CD30|D1S166E|Ki-1 | 1 | Cytokine_Receptors |
| TNFRSF9 | 4-1BB|CD137|CDw137|ILA|MGC2172 | 1 | Cytokine_Receptors |
| TRHR | MGC141920 | 8 | Cytokine_Receptors |
| TSHR | CHNG1|LGR3|MGC75129|hTSHR-I | 14 | Cytokine_Receptors |
| TUBB3 | MC1R|TUBB4|beta-4 | 16 | Cytokine_Receptors |
| VDR | NR1I1 | 12 | Cytokine_Receptors |
| VIPR1 | FLJ41949|HVR1|II|PACAP-R-2|RDC1|VAPC1|VIPR|VIRG|VPAC1|VPCAP1R | 3 | Cytokine_Receptors |
| VIPR2 | FLJ16511|VPAC2|VPCAP2R | 7 | Cytokine_Receptors |
| XCR1 | CCXCR1|GPR5 | 3 | Cytokine_Receptors |
| IFNA10 | MGC119878|MGC119879 | 9 | Interferons |
| IFNA13 | - | 9 | Interferons |
| IFNA14 | LEIF2H|MGC125756|MGC125757 | 9 | Interferons |
| IFNA16 | - | 9 | Interferons |
| IFNA17 | IFNA|INFA|LEIF2C1 | 9 | Interferons |
| IFNA2 | IFNA|INFA2|MGC125764|MGC125765 | 9 | Interferons |
| IFNA21 | MGC126687|MGC126689 | 9 | Interferons |
| IFNA4 | INFA4|MGC142200 | 9 | Interferons |
| IFNA5 | INFA5 | 9 | Interferons |
| IFNA6 | - | 9 | Interferons |
| IFNA7 | IFNA-J | 9 | Interferons |
| IFNA8 | - | 9 | Interferons |
| IFNB1 | IFB|IFF|IFNB|MGC96956 | 9 | Interferons |
| IFNE | IFN-E|IFNE1|IFNT1|MGC119018|MGC119020|PRO655 | 9 | Interferons |
| IFNG | IFG|IFI | 12 | Interferons |
| IFNK | RP11-27J8.1 | 9 | Interferons |
| IFNW1 | - | 9 | Interferons |
| IFNAR2 | IFN-R|IFN-alpha-REC|IFNABR|IFNARB | 21 | Interferon_Receptor |
| IFNGR1 | CD119|FLJ45734|IFNGR | 6 | Interferon_Receptor |
| IFNGR2 | AF-1|IFGR2|IFNGT1 | 21 | Interferon_Receptor |
| IL11 | AGIF|IL-11 | 19 | Interleukins |
| IL12A | CLMF|IL-12A|NFSK|NKSF1|P35 | 3 | Interleukins |
| IL12B | CLMF|CLMF2|IL-12B|NKSF|NKSF2 | 5 | Interleukins |
| IL13 | ALRH|BHR1|IL-13|MGC116786|MGC116788|MGC116789|P600 | 5 | Interleukins |
| IL15 | IL-15|MGC9721 | 4 | Interleukins |
| IL16 | FLJ16806|FLJ42735|FLJ44234|HsT19289|IL-16|LCF|prIL-16 | 15 | Interleukins |
| IL17A | CTLA8|IL-17|IL-17A|IL17 | 6 | Interleukins |
| IL17B | IL-17B|IL-20|MGC138900|MGC138901|ZCYTO7 | 5 | Interleukins |
| IL17C | CX2|IL-17C|IL-21|MGC126884|MGC138401 | 16 | Interleukins |
| IL17D | FLJ30846|IL-17D|IL-22|IL-27|IL27 | 13 | Interleukins |
| IL17F | IL-17F|ML-1|ML1 | 6 | Interleukins |
| IL18 | IGIF|IL-18|IL-1g|IL1F4|MGC12320 | 11 | Interleukins |
| IL19 | IL-10C|MDA1|NG.1|ZMDA1 | 1 | Interleukins |
| IL1A | IL-1A|IL1|IL1-ALPHA|IL1F1 | 2 | Interleukins |
| IL1B | IL-1|IL1-BETA|IL1F2 | 2 | Interleukins |
| IL1F10 | FIL1-theta|FKSG75|IL-1HY2|IL1-theta|MGC119831|MGC119832|MGC119833 | 2 | Interleukins |
| IL1F5 | FIL1|FIL1(DELTA)|FIL1D|IL1HY1|IL1L1|IL1RP3|MGC29840 | 2 | Interleukins |
| IL1F6 | FIL1|FIL1(EPSILON)|FIL1E|IL-1F6|IL1(EPSILON)|MGC129552|MGC129553 | 2 | Interleukins |
| IL1F7 | FIL1|FIL1(ZETA)|FIL1Z|IL-1F7|IL-1H4|IL-1RP1|IL1H4|IL1RP1 | 2 | Interleukins |
| IL1F8 | FIL1|FIL1-(ETA)|FIL1H|IL-1F8|IL-1H2|IL1-ETA|IL1H2|MGC126880|MGC126882 | 2 | Interleukins |
| IL1F9 | IL-1F9|IL-1H1|IL-1RP2|IL1E|IL1H1|IL1RP2 | 2 | Interleukins |
| IL1RN | ICIL-1RA|IL-1ra3|IL1F3|IL1RA|IRAP|MGC10430 | 2 | Interleukins |
| IL2 | IL-2|TCGF|lymphokine | 4 | Interleukins |
| IL20 | IL-20|IL10D|MGC96907|ZCYTO10 | 1 | Interleukins |
| IL21 | IL-21|Za11 | 4 | Interleukins |
| IL22 | IL-21|IL-22|IL-D110|IL-TIF|IL21|ILTIF|MGC79382|MGC79384|TIFIL-23|TIFa|zcyto18 | 12 | Interleukins |
| IL23A | IL-23|IL-23A|IL23P19|MGC79388|P19|SGRF | 12 | Interleukins |
| IL24 | C49A|FISP|IL-24|IL10B|MDA7|Mob-5|ST16|mda-7 | 1 | Interleukins |
| IL25 | IL-17E|IL-25|IL17E | 14 | Interleukins |
| IL26 | AK155|IL-26 | 12 | Interleukins |
| IL27 | IL-27|IL-27A|IL27p28|IL30|MGC71873|p28 | 16 | Interleukins |
| IL28A | IFNL2|IL-28A | 19 | Interleukins |
| IL28B | IFNL3|IL-28B|IL28C | 19 | Interleukins |
| IL29 | IFNL1|IL-29 | 19 | Interleukins |
| IL3 | IL-3|MCGF|MGC79398|MGC79399|MULTI-CSF | 5 | Interleukins |
| IL31 | IL-31 | 12 | Interleukins |
| IL32 | IL-32alpha|IL-32beta|IL-32delta|IL-32gamma|NK4|TAIF|TAIFa|TAIFb|TAIFc|TAIFd | 16 | Interleukins |
| IL33 | C9orf26|DKFZp586H0523|DVS27|NF-HEV|NFEHEV|RP11-575C20.2 | 9 | Interleukins |
| IL34 | C16orf77|IL-34|MGC34647 | 16 | Interleukins |
| IL4 | BCGF-1|BCGF1|BSF1|IL-4|MGC79402 | 5 | Interleukins |
| IL5 | EDF|IL-5|TRF | 5 | Interleukins |
| IL6 | BSF2|HGF|HSF|IFNB2|IL-6 | 7 | Interleukins |
| IL6ST | CD130|CDw130|GP130|GP130-RAPS|IL6R-beta | 5 | Interleukins |
| IL7 | IL-7 | 8 | Interleukins |
| IL8 | CXCL8|GCP-1|GCP1|LECT|LUCT|LYNAP|MDNCF|MONAP|NAF|NAP-1|NAP1 | 4 | Interleukins |
| IL9 | HP40|IL-9|P40 | 5 | Interleukins |
| TXLNA | DKFZp451J0118|IL14|MGC118870|MGC118871|RP4-622L5.4|TXLN | 1 | Interleukins |
| IL10RA | CDW210A|HIL-10R|IL-10R1|IL10R | 11 | Interleukins_Receptor |
| IL10RB | CDW210B|CRF2-4|CRFB4|D21S58|D21S66|IL-10R2 | 21 | Interleukins_Receptor |
| IL11RA | MGC2146 | 9 | Interleukins_Receptor |
| IL11RB | - | - | Interleukins_Receptor |
| IL12RB1 | CD212|IL-12R-BETA1|IL12RB|MGC34454 | 19 | Interleukins_Receptor |
| IL12RB2 | - | 1 | Interleukins_Receptor |
| IL13RA1 | CD213A1|IL-13Ra|NR4 | X | Interleukins_Receptor |
| IL13RA2 | CD213A2|CT19|IL-13R|IL13BP | X | Interleukins_Receptor |
| IL15RA | MGC104179 | 10 | Interleukins_Receptor |
| IL15RB | - | - | Interleukins_Receptor |
| IL17RA | CD217|CDw217|IL-17RA|IL17R|MGC10262|hIL-17R | 22 | Interleukins_Receptor |
| IL17RB | CRL4|EVI27|IL17BR|IL17RH1|MGC5245 | 3 | Interleukins_Receptor |
| IL17RC | FLJ95963|FLJ96005|IL17-RL|IL17RL|MGC10763 | 3 | Interleukins_Receptor |
| IL17RD | DKFZp434N1928|FLJ35755|IL-17RD|IL17RLM|MGC133309|SEF | 3 | Interleukins_Receptor |
| IL17RE | FLJ23658|MGC71884 | 3 | Interleukins_Receptor |
| IL18R1 | CD218a|CDw218a|IL-1Rrp|IL18RA|IL1RRP | 2 | Interleukins_Receptor |
| IL18RAP | ACPL|CD218b|CDw218b|IL18RB|MGC120589|MGC120590 | 2 | Interleukins_Receptor |
| IL1R1 | CD121A|D2S1473|IL-1R-alpha|IL1R|IL1RA|P80 | 2 | Interleukins_Receptor |
| IL1R2 | CD121b|IL1RB|MGC47725 | 2 | Interleukins_Receptor |
| IL1RAP | C3orf13|FLJ37788|IL-1RAcP|IL1R3 | 3 | Interleukins_Receptor |
| IL1RL1 | DER4|FIT-1|MGC32623|ST2|ST2L|ST2V|T1 | 2 | Interleukins_Receptor |
| IL1RL2 | IL1R-rp2|IL1RRP2 | 2 | Interleukins_Receptor |
| IL20RA | FLJ40993|IL-20R1|ZCYTOR7 | 6 | Interleukins_Receptor |
| IL20RB | DIRS1|FNDC6|IL-20R2|MGC34923 | 3 | Interleukins_Receptor |
| IL21R | MGC10967|NILR | 16 | Interleukins_Receptor |
| IL22RA1 | CRF2-9|IL22R|IL22R1 | 1 | Interleukins_Receptor |
| IL22RA2 | CRF2-10|CRF2-S1|CRF2X|IL-22BP|MGC150509|MGC150510 | 6 | Interleukins_Receptor |
| IL23R | - | 1 | Interleukins_Receptor |
| IL27RA | CRL1|IL27R|TCCR|WSX1|zcytor1 | 19 | Interleukins_Receptor |
| IL28RA | CRF2/12|IFNLR|IFNLR1|IL-28R1|LICR2 | 1 | Interleukins_Receptor |
| IL2RA | CD25|IDDM10|IL2R|TCGFR | 10 | Interleukins_Receptor |
| IL2RB | CD122|P70-75 | 22 | Interleukins_Receptor |
| IL2RG | CD132|IMD4|SCIDX|SCIDX1 | X | Interleukins_Receptor |
| IL31RA | CRL|CRL3|GLM-R|GLMR|GPL|IL-31RA|MGC125346|PRO21384 | 5 | Interleukins_Receptor |
| IL3RA | CD123|IL3R|IL3RAY|IL3RX|IL3RY|MGC34174|hIL-3Ra | X|Y | Interleukins_Receptor |
| IL4R | CD124|IL4RA | 16 | Interleukins_Receptor |
| IL5RA | CD125|CDw125|HSIL5R3|IL5R|MGC26560 | 3 | Interleukins_Receptor |
| IL6R | CD126|IL-6R-1|IL-6R-alpha|IL6RA|MGC104991 | 1 | Interleukins_Receptor |
| IL7R | CD127|CDW127|IL-7R-alpha|IL7RA|ILRA | 5 | Interleukins_Receptor |
| IL8RA | C-C|C-C-CKR-1|CD128|CD181|CDw128a|CKR-1|CMKAR1|CXCR1|IL8R1|IL8RBA | 2 | Interleukins_Receptor |
| IL8RB | CD182|CDw128b|CMKAR2|CXCR2|IL8R2|IL8RA | 2 | Interleukins_Receptor |
| IL9R | CD129 | X|Y | Interleukins_Receptor |
| ST2 | - | 11 | Interleukins_Receptor |
| HLA-A | FLJ26655|HLAA | 6 | NaturalKiller_Cell_Cytotoxicity |
| HLA-B | AS|HLA-B-7301|HLA-B73|HLAB|HLAC|MGC111087|SPDA1 | 6 | NaturalKiller_Cell_Cytotoxicity |
| HLA-C | D6S204|FLJ27082|HLA-Cw|HLA-Cw12|HLA-JY3|HLC-C|PSORS1 | 6 | NaturalKiller_Cell_Cytotoxicity |
| HLA-E | DKFZp686P19218|EA1.2|EA2.1|HLA-6.2|MHC|QA1 | 6 | NaturalKiller_Cell_Cytotoxicity |
| HLA-G | MHC-G | 6 | NaturalKiller_Cell_Cytotoxicity |
| KIR3DL1 | CD158E1|KIR|MGC119726|MGC119728|MGC126589|MGC126591|NKAT3|NKB1|NKB1B | 19 | NaturalKiller_Cell_Cytotoxicity |
| KIR3DL2 | CD158K|MGC125321|NKAT4|NKAT4B|p140 | 19 | NaturalKiller_Cell_Cytotoxicity |
| KIR2DL1 | CD158A|KIR-K64|KIR221|NKAT|NKAT1|p58.1 | 19 | NaturalKiller_Cell_Cytotoxicity |
| KIR2DL2 | CD158B1|CD158b|NKAT6|p58.2 | 19 | NaturalKiller_Cell_Cytotoxicity |
| KIR2DL3 | CD158B2|CD158b|GL183|KIR-023GB|KIR-K7b|KIR-K7c|KIRCL23|MGC129943|NKAT|NKAT2|NKAT2A|NKAT2B|p58 | 19 | NaturalKiller_Cell_Cytotoxicity |
| KIR2DL4 | CD158D|G9P|KIR103|KIR103AS | 19 | NaturalKiller_Cell_Cytotoxicity |
| KIR2DL5A | CD158F|KIR2DL5|KIR2DL5.1|KIR2DL5.3 | 19 | NaturalKiller_Cell_Cytotoxicity |
| KLRC1 | CD159A|MGC13374|MGC59791|NKG2|NKG2A | 12 | NaturalKiller_Cell_Cytotoxicity |
| KLRC2 | CD159c|MGC138244|NKG2-C|NKG2C | 12 | NaturalKiller_Cell_Cytotoxicity |
| KLRC3 | NKG2-E|NKG2E | 12 | NaturalKiller_Cell_Cytotoxicity |
| KLRD1 | CD94 | 12 | NaturalKiller_Cell_Cytotoxicity |
| PTPN6 | HCP|HCPH|HPTP1C|PTP-1C|SH-PTP1|SHP-1|SHP-1L|SHP1 | 12 | NaturalKiller_Cell_Cytotoxicity |
| PTPN11 | BPTP3|CFC|MGC14433|NS1|PTP-1D|PTP2C|SH-PTP2|SH-PTP3|SHP2 | 12 | NaturalKiller_Cell_Cytotoxicity |
| ICAM1 | BB2|CD54|P3.58 | 19 | NaturalKiller_Cell_Cytotoxicity |
| ICAM2 | CD102 | 17 | NaturalKiller_Cell_Cytotoxicity |
| ITGAL | CD11A|LFA-1|LFA1A | 16 | NaturalKiller_Cell_Cytotoxicity |
| ITGB2 | CD18|LAD|LCAMB|LFA-1|MAC-1|MF17|MFI7 | 21 | NaturalKiller_Cell_Cytotoxicity |
| PTK2B | CADTK|CAKB|FADK2|FAK2|FRNK|PKB|PTK|PYK2|RAFTK | 8 | NaturalKiller_Cell_Cytotoxicity |
| VAV3 | FLJ40431 | 1 | NaturalKiller_Cell_Cytotoxicity |
| VAV1 | VAV | 19 | NaturalKiller_Cell_Cytotoxicity |
| VAV2 | - | 9 | NaturalKiller_Cell_Cytotoxicity |
| RAC1 | MGC111543|MIG5|TC-25|p21-Rac1 | 7 | NaturalKiller_Cell_Cytotoxicity |
| RAC2 | EN-7|Gx|HSPC022 | 22 | NaturalKiller_Cell_Cytotoxicity |
| RAC3 | - | 17 | NaturalKiller_Cell_Cytotoxicity |
| PAK1 | MGC130000|MGC130001|PAKalpha | 11 | NaturalKiller_Cell_Cytotoxicity |
| MAP2K1 | MAPKK1|MEK1|MKK1|PRKMK1 | 15 | NaturalKiller_Cell_Cytotoxicity |
| MAP2K2 | FLJ26075|MAPKK2|MEK2|MKK2|PRKMK2 | 19 | NaturalKiller_Cell_Cytotoxicity |
| MAPK1 | ERK|ERK2|ERT1|MAPK2|P42MAPK|PRKM1|PRKM2|p38|p40|p41|p41mapk | 22 | NaturalKiller_Cell_Cytotoxicity |
| MAPK3 | ERK1|HS44KDAP|HUMKER1A|MGC20180|P44ERK1|P44MAPK|PRKM3 | 16 | NaturalKiller_Cell_Cytotoxicity |
| TNF | DIF|TNF-alpha|TNFA|TNFSF2 | 6 | NaturalKiller_Cell_Cytotoxicity |
| CSF2 | GMCSF|MGC131935|MGC138897 | 5 | NaturalKiller_Cell_Cytotoxicity |
| IFNG | IFG|IFI | 12 | NaturalKiller_Cell_Cytotoxicity |
| KIR2DS1 | CD158H|CD158a|p50.1 | 19 | NaturalKiller_Cell_Cytotoxicity |
| KIR2DS3 | NKAT7 | 19 | NaturalKiller_Cell_Cytotoxicity |
| KIR2DS4 | CD158I|KIR1D|KIR412|KKA3|MGC120019|MGC125315|MGC125317|NKAT8 | 19 | NaturalKiller_Cell_Cytotoxicity |
| KIR2DS5 | CD158G|NKAT9 | 19 | NaturalKiller_Cell_Cytotoxicity |
| NCR2 | CD336|LY95|NK-p44|NKP44|dJ149M18.1 | 6 | NaturalKiller_Cell_Cytotoxicity |
| TYROBP | DAP12|KARAP|PLOSL | 19 | NaturalKiller_Cell_Cytotoxicity |
| LCK | YT16|p56lck|pp58lck | 1 | NaturalKiller_Cell_Cytotoxicity |
| FCGR3A | CD16|CD16A|FCG3|FCGR3|FCGRIII|FCR-10|FCRIII|FCRIIIA|IGFR3 | 1 | NaturalKiller_Cell_Cytotoxicity |
| FCGR3B | CD16|CD16b|FCG3|FCGR3 | 1 | NaturalKiller_Cell_Cytotoxicity |
| LOC652578 | - | Un | NaturalKiller_Cell_Cytotoxicity |
| NCR1 | CD335|FLJ99094|LY94|NK-p46|NKP46 | 19 | NaturalKiller_Cell_Cytotoxicity |
| NCR3 | 1C7|CD337|LY117|MALS|NKp30 | 6 | NaturalKiller_Cell_Cytotoxicity |
| FCER1G | FCRG | 1 | NaturalKiller_Cell_Cytotoxicity |
| CD247 | CD3-ZETA|CD3H|CD3Q|CD3Z|T3Z|TCRZ | 1 | NaturalKiller_Cell_Cytotoxicity |
| ZAP70 | FLJ17670|FLJ17679|SRK|STD|TZK|ZAP-70 | 2 | NaturalKiller_Cell_Cytotoxicity |
| SYK | DKFZp313N1010|FLJ25043|FLJ37489 | 9 | NaturalKiller_Cell_Cytotoxicity |
| LCP2 | SLP-76|SLP76 | 5 | NaturalKiller_Cell_Cytotoxicity |
| LAT | LAT1|pp36 | 16 | NaturalKiller_Cell_Cytotoxicity |
| PLCG1 | PLC-II|PLC1|PLC148|PLCgamma1 | 20 | NaturalKiller_Cell_Cytotoxicity |
| PLCG2 | - | 16 | NaturalKiller_Cell_Cytotoxicity |
| SH3BP2 | 3BP2|CRBM|CRPM|FLJ42079|FLJ54978|RES4-23 | 4 | NaturalKiller_Cell_Cytotoxicity |
| PIK3CA | MGC142161|MGC142163|PI3K|p110-alpha | 3 | NaturalKiller_Cell_Cytotoxicity |
| PIK3CB | DKFZp779K1237|MGC133043|PI3K|PI3KCB|PI3Kbeta|PIK3C1|p110-BETA | 3 | NaturalKiller_Cell_Cytotoxicity |
| PIK3CD | p110D | 1 | NaturalKiller_Cell_Cytotoxicity |
| PIK3CG | PI3CG|PI3K|PI3Kgamma|PIK3 | 7 | NaturalKiller_Cell_Cytotoxicity |
| PIK3R5 | F730038I15Rik|FOAP-2|P101-PI3K|p101 | 17 | NaturalKiller_Cell_Cytotoxicity |
| PIK3R1 | GRB1|p85|p85-ALPHA | 5 | NaturalKiller_Cell_Cytotoxicity |
| PIK3R2 | P85B|p85|p85-BETA | 19 | NaturalKiller_Cell_Cytotoxicity |
| PIK3R3 | DKFZp686P05226|FLJ41892|p55|p55-GAMMA | 1 | NaturalKiller_Cell_Cytotoxicity |
| FYN | MGC45350|SLK|SYN | 6 | NaturalKiller_Cell_Cytotoxicity |
| SHC2 | SCK|SHCB|SLI | 19 | NaturalKiller_Cell_Cytotoxicity |
| SHC4 | MGC34023|RaLP|SHCD | 15 | NaturalKiller_Cell_Cytotoxicity |
| SHC3 | N-Shc|NSHC|RAI|SHCC | 9 | NaturalKiller_Cell_Cytotoxicity |
| SHC1 | FLJ26504|SHC|SHCA | 1 | NaturalKiller_Cell_Cytotoxicity |
| GRB2 | ASH|EGFRBP-GRB2|Grb3-3|MST084|MSTP084 | 17 | NaturalKiller_Cell_Cytotoxicity |
| SOS1 | GF1|GGF1|GINGF|HGF|NS4 | 2 | NaturalKiller_Cell_Cytotoxicity |
| SOS2 | FLJ25596 | 14 | NaturalKiller_Cell_Cytotoxicity |
| HRAS | C-BAS/HAS|C-H-RAS|C-HA-RAS1|CTLO|H-RASIDX|HAMSV|HRAS1|K-RAS|N-RAS|RASH1 | 11 | NaturalKiller_Cell_Cytotoxicity |
| KRAS | C-K-RAS|K-RAS2A|K-RAS2B|K-RAS4A|K-RAS4B|KI-RAS|KRAS1|KRAS2|NS3|RASK2 | 12 | NaturalKiller_Cell_Cytotoxicity |
| NRAS | ALPS4|N-ras|NRAS1 | 1 | NaturalKiller_Cell_Cytotoxicity |
| ARAF | A-RAF|ARAF1|PKS2|RAFA1 | X | NaturalKiller_Cell_Cytotoxicity |
| BRAF | B-RAF1|BRAF1|FLJ95109|MGC126806|MGC138284|RAFB1 | 7 | NaturalKiller_Cell_Cytotoxicity |
| RAF1 | CRAF|NS5|Raf-1|c-Raf | 3 | NaturalKiller_Cell_Cytotoxicity |
| MICA | FLJ60820|MGC111087|PERB11.1 | 6 | NaturalKiller_Cell_Cytotoxicity |
| MICB | PERB11.2 | 6 | NaturalKiller_Cell_Cytotoxicity |
| ULBP3 | RAET1N | 6 | NaturalKiller_Cell_Cytotoxicity |
| ULBP2 | N2DL2|RAET1H | 6 | NaturalKiller_Cell_Cytotoxicity |
| ULBP1 | RAET1I | 6 | NaturalKiller_Cell_Cytotoxicity |
| KLRK1 | CD314|D12S2489E|FLJ17759|FLJ75772|KLR|NKG2-D|NKG2D | 12 | NaturalKiller_Cell_Cytotoxicity |
| HCST | DAP10|DKFZP586C1522|KAP10|PIK3AP | 19 | NaturalKiller_Cell_Cytotoxicity |
| CD48 | BCM1|BLAST|BLAST1|MEM-102|SLAMF2|hCD48|mCD48 | 1 | NaturalKiller_Cell_Cytotoxicity |
| CD244 | 2B4|NAIL|NKR2B4|Nmrk|SLAMF4 | 1 | NaturalKiller_Cell_Cytotoxicity |
| PPP3CA | CALN|CALNA|CALNA1|CCN1|CNA1|PPP2B | 4 | NaturalKiller_Cell_Cytotoxicity |
| PPP3CB | CALNA2|CALNB | 10 | NaturalKiller_Cell_Cytotoxicity |
| PPP3CC | CALNA3 | 8 | NaturalKiller_Cell_Cytotoxicity |
| CHP | SLC9A1BP | 15 | NaturalKiller_Cell_Cytotoxicity |
| PPP3R1 | CALNB1|CNB|CNB1 | 2 | NaturalKiller_Cell_Cytotoxicity |
| PPP3R2 | PPP3RL | 9 | NaturalKiller_Cell_Cytotoxicity |
| CHP2 | - | 16 | NaturalKiller_Cell_Cytotoxicity |
| NFAT5 | KIAA0827|NF-AT5|NFATL1|NFATZ|OREBP|TONEBP | 16 | NaturalKiller_Cell_Cytotoxicity |
| NFATC1 | MGC138448|NF-ATC|NFAT2|NFATc | 18 | NaturalKiller_Cell_Cytotoxicity |
| NFATC2 | NFAT1|NFATP | 20 | NaturalKiller_Cell_Cytotoxicity |
| NFATC3 | NFAT4|NFATX | 16 | NaturalKiller_Cell_Cytotoxicity |
| NFATC4 | NF-ATc4|NFAT3 | 14 | NaturalKiller_Cell_Cytotoxicity |
| PRKCA | AAG6|MGC129900|MGC129901|PKC-alpha|PKCA|PRKACA | 17 | NaturalKiller_Cell_Cytotoxicity |
| PRKCB | MGC41878|PKC-beta|PKCB|PRKCB1|PRKCB2 | 16 | NaturalKiller_Cell_Cytotoxicity |
| PRKCG | MGC57564|PKC-gamma|PKCC|PKCG|SCA14 | 19 | NaturalKiller_Cell_Cytotoxicity |
| SH2D1B | EAT2 | 1 | NaturalKiller_Cell_Cytotoxicity |
| SH2D1A | DSHP|EBVS|FLJ18687|FLJ92177|IMD5|LYP|MTCP1|SAP|XLP|XLPD | X | NaturalKiller_Cell_Cytotoxicity |
| IFNGR1 | CD119|FLJ45734|IFNGR | 6 | NaturalKiller_Cell_Cytotoxicity |
| IFNGR2 | AF-1|IFGR2|IFNGT1 | 21 | NaturalKiller_Cell_Cytotoxicity |
| IFNA1 | IFL|IFN|IFN-ALPHA|IFNA13|IFNA@|MGC138207|MGC138505|MGC138507 | 9 | NaturalKiller_Cell_Cytotoxicity |
| IFNA2 | IFNA|INFA2|MGC125764|MGC125765 | 9 | NaturalKiller_Cell_Cytotoxicity |
| IFNA4 | INFA4|MGC142200 | 9 | NaturalKiller_Cell_Cytotoxicity |
| IFNA5 | INFA5 | 9 | NaturalKiller_Cell_Cytotoxicity |
| IFNA6 | - | 9 | NaturalKiller_Cell_Cytotoxicity |
| IFNA7 | IFNA-J | 9 | NaturalKiller_Cell_Cytotoxicity |
| IFNA8 | - | 9 | NaturalKiller_Cell_Cytotoxicity |
| IFNA10 | MGC119878|MGC119879 | 9 | NaturalKiller_Cell_Cytotoxicity |
| IFNA13 | - | 9 | NaturalKiller_Cell_Cytotoxicity |
| IFNA14 | LEIF2H|MGC125756|MGC125757 | 9 | NaturalKiller_Cell_Cytotoxicity |
| IFNA16 | - | 9 | NaturalKiller_Cell_Cytotoxicity |
| IFNA17 | IFNA|INFA|LEIF2C1 | 9 | NaturalKiller_Cell_Cytotoxicity |
| IFNA21 | MGC126687|MGC126689 | 9 | NaturalKiller_Cell_Cytotoxicity |
| IFNB1 | IFB|IFF|IFNB|MGC96956 | 9 | NaturalKiller_Cell_Cytotoxicity |
| IFNAR1 | AVP|IFN-alpha-REC|IFNAR|IFNBR|IFRC | 21 | NaturalKiller_Cell_Cytotoxicity |
| IFNAR2 | IFN-R|IFN-alpha-REC|IFNABR|IFNARB | 21 | NaturalKiller_Cell_Cytotoxicity |
| TNFSF10 | APO2L|Apo-2L|CD253|TL2|TRAIL | 3 | NaturalKiller_Cell_Cytotoxicity |
| TNFRSF10D | CD264|DCR2|TRAILR4|TRUNDD | 8 | NaturalKiller_Cell_Cytotoxicity |
| TNFRSF10C | CD263|DCR1|LIT|MGC149501|MGC149502|TRAILR3|TRID | 8 | NaturalKiller_Cell_Cytotoxicity |
| TNFRSF10B | CD262|DR5|KILLER|KILLER/DR5|TRAIL-R2|TRAILR2|TRICK2|TRICK2A|TRICK2B|TRICKB|ZTNFR9 | 8 | NaturalKiller_Cell_Cytotoxicity |
| TNFRSF10A | APO2|CD261|DR4|MGC9365|TRAILR-1|TRAILR1 | 8 | NaturalKiller_Cell_Cytotoxicity |
| FASLG | APT1LG1|CD178|CD95L|FASL|TNFSF6 | 1 | NaturalKiller_Cell_Cytotoxicity |
| FAS | ALPS1A|APO-1|APT1|CD95|FAS1|FASTM|TNFRSF6 | 10 | NaturalKiller_Cell_Cytotoxicity |
| GZMB | CCPI|CGL-1|CGL1|CSP-B|CSPB|CTLA1|CTSGL1|HLP|SECT | 14 | NaturalKiller_Cell_Cytotoxicity |
| PRF1 | FLH2|HPLH2|MGC65093|P1|PFN1|PFP | 10 | NaturalKiller_Cell_Cytotoxicity |
| CASP3 | CPP32|CPP32B|SCA-1 | 4 | NaturalKiller_Cell_Cytotoxicity |
| BID | FP497|MGC15319|MGC42355 | 22 | NaturalKiller_Cell_Cytotoxicity |
| CD3D | CD3-DELTA|T3D | 11 | TCRsignalingPathway |
| CD3E | FLJ18683|T3E|TCRE | 11 | TCRsignalingPathway |
| CD3G | CD3-GAMMA|FLJ17620|FLJ17664|FLJ79544|FLJ94613|MGC138597|T3G | 11 | TCRsignalingPathway |
| CD247 | CD3-ZETA|CD3H|CD3Q|CD3Z|T3Z|TCRZ | 1 | TCRsignalingPathway |
| CD4 | CD4mut | 12 | TCRsignalingPathway |
| CD8A | CD8|Leu2|MAL|p32 | 2 | TCRsignalingPathway |
| CD8B | CD8B1|LYT3|Leu2|Ly3|MGC119115 | 2 | TCRsignalingPathway |
| PTPRC | B220|CD45|CD45R|GP180|LCA|LY5|T200 | 1 | TCRsignalingPathway |
| LCK | YT16|p56lck|pp58lck | 1 | TCRsignalingPathway |
| FYN | MGC45350|SLK|SYN | 6 | TCRsignalingPathway |
| ZAP70 | FLJ17670|FLJ17679|SRK|STD|TZK|ZAP-70 | 2 | TCRsignalingPathway |
| LCP2 | SLP-76|SLP76 | 5 | TCRsignalingPathway |
| LAT | LAT1|pp36 | 16 | TCRsignalingPathway |
| ITK | EMT|LYK|MGC126257|MGC126258|PSCTK2 | 5 | TCRsignalingPathway |
| TEC | MGC126760|MGC126762|PSCTK4 | 4 | TCRsignalingPathway |
| NCK1 | MGC12668|NCK|NCKalpha | 3 | TCRsignalingPathway |
| NCK2 | GRB4|NCKbeta | 2 | TCRsignalingPathway |
| VAV3 | FLJ40431 | 1 | TCRsignalingPathway |
| VAV1 | VAV | 19 | TCRsignalingPathway |
| VAV2 | - | 9 | TCRsignalingPathway |
| GRAP2 | GADS|GRAP-2|GRB2L|GRBLG|GRID|GRPL|GrbX|Grf40|Mona|P38 | 22 | TCRsignalingPathway |
| GRB2 | ASH|EGFRBP-GRB2|Grb3-3|MST084|MSTP084 | 17 | TCRsignalingPathway |
| PAK1 | MGC130000|MGC130001|PAKalpha | 11 | TCRsignalingPathway |
| PAK2 | PAK65|PAKgamma | 3 | TCRsignalingPathway |
| PAK3 | CDKN1A|MRX30|MRX47|OPHN3|PAK3beta|bPAK|hPAK3 | X | TCRsignalingPathway |
| PAK4 | - | 19 | TCRsignalingPathway |
| PAK6 | PAK5 | 15 | TCRsignalingPathway |
| PAK7 | KIAA1264|MGC26232|PAK5 | 20 | TCRsignalingPathway |
| RHOA | ARH12|ARHA|RHO12|RHOH12 | 3 | TCRsignalingPathway |
| CDC42 | CDC42Hs|G25K | 1 | TCRsignalingPathway |
| PPP3CA | CALN|CALNA|CALNA1|CCN1|CNA1|PPP2B | 4 | TCRsignalingPathway |
| PPP3CB | CALNA2|CALNB | 10 | TCRsignalingPathway |
| PPP3CC | CALNA3 | 8 | TCRsignalingPathway |
| CHP | SLC9A1BP | 15 | TCRsignalingPathway |
| PPP3R1 | CALNB1|CNB|CNB1 | 2 | TCRsignalingPathway |
| PPP3R2 | PPP3RL | 9 | TCRsignalingPathway |
| CHP2 | - | 16 | TCRsignalingPathway |
| NFAT5 | KIAA0827|NF-AT5|NFATL1|NFATZ|OREBP|TONEBP | 16 | TCRsignalingPathway |
| NFATC1 | MGC138448|NF-ATC|NFAT2|NFATc | 18 | TCRsignalingPathway |
| NFATC2 | NFAT1|NFATP | 20 | TCRsignalingPathway |
| NFATC3 | NFAT4|NFATX | 16 | TCRsignalingPathway |
| NFATC4 | NF-ATc4|NFAT3 | 14 | TCRsignalingPathway |
| SOS1 | GF1|GGF1|GINGF|HGF|NS4 | 2 | TCRsignalingPathway |
| SOS2 | FLJ25596 | 14 | TCRsignalingPathway |
| HRAS | C-BAS/HAS|C-H-RAS|C-HA-RAS1|CTLO|H-RASIDX|HAMSV|HRAS1|K-RAS|N-RAS|RASH1 | 11 | TCRsignalingPathway |
| KRAS | C-K-RAS|K-RAS2A|K-RAS2B|K-RAS4A|K-RAS4B|KI-RAS|KRAS1|KRAS2|NS3|RASK2 | 12 | TCRsignalingPathway |
| NRAS | ALPS4|N-ras|NRAS1 | 1 | TCRsignalingPathway |
| FOS | AP-1|C-FOS | 14 | TCRsignalingPathway |
| JUN | AP-1|AP1|c-Jun | 1 | TCRsignalingPathway |
| CARD11 | BIMP3|CARMA1|MGC133069 | 7 | TCRsignalingPathway |
| BCL10 | CARMEN|CIPER|CLAP|c-E10|mE10 | 1 | TCRsignalingPathway |
| MALT1 | DKFZp434L132|MLT|MLT1 | 18 | TCRsignalingPathway |
| CHUK | IKBKA|IKK-alpha|IKK1|IKKA|NFKBIKA|TCF16 | 10 | TCRsignalingPathway |
| IKBKB | FLJ40509|IKK-beta|IKK2|IKKB|MGC131801|NFKBIKB | 8 | TCRsignalingPathway |
| IKBKG | AMCBX1|FIP-3|FIP3|Fip3p|IKK-gamma|IP|IP1|IP2|IPD2|NEMO | X | TCRsignalingPathway |
| NFKB1 | DKFZp686C01211|EBP-1|KBF1|MGC54151|NF-kappa-B|NFKB-p105|NFKB-p50|p105|p50 | 4 | TCRsignalingPathway |
| RELA | MGC131774|NFKB3|p65 | 11 | TCRsignalingPathway |
| NFKBIA | IKBA|MAD-3|NFKBI | 14 | TCRsignalingPathway |
| NFKBIB | IKBB|TRIP9 | 19 | TCRsignalingPathway |
| NFKBIE | IKBE | 6 | TCRsignalingPathway |
| CD28 | MGC138290|Tp44 | 2 | TCRsignalingPathway |
| ICOS | AILIM|CD278|MGC39850 | 2 | TCRsignalingPathway |
| CD40LG | CD154|CD40L|HIGM1|IGM|IMD3|T-BAM|TNFSF5|TRAP|gp39|hCD40L | X | TCRsignalingPathway |
| PIK3R5 | F730038I15Rik|FOAP-2|P101-PI3K|p101 | 17 | TCRsignalingPathway |
| PIK3R1 | GRB1|p85|p85-ALPHA | 5 | TCRsignalingPathway |
| PIK3R2 | P85B|p85|p85-BETA | 19 | TCRsignalingPathway |
| PIK3R3 | DKFZp686P05226|FLJ41892|p55|p55-GAMMA | 1 | TCRsignalingPathway |
| PIK3CA | MGC142161|MGC142163|PI3K|p110-alpha | 3 | TCRsignalingPathway |
| PIK3CB | DKFZp779K1237|MGC133043|PI3K|PI3KCB|PI3Kbeta|PIK3C1|p110-BETA | 3 | TCRsignalingPathway |
| PIK3CD | p110D | 1 | TCRsignalingPathway |
| PIK3CG | PI3CG|PI3K|PI3Kgamma|PIK3 | 7 | TCRsignalingPathway |
| AKT3 | DKFZp434N0250|PKB-GAMMA|PKBG|PRKBG|RAC-PK-gamma|RAC-gamma|STK-2 | 1 | TCRsignalingPathway |
| AKT1 | AKT|MGC99656|PKB|PKB-ALPHA|PRKBA|RAC|RAC-ALPHA | 14 | TCRsignalingPathway |
| AKT2 | PKBB|PKBBETA|PRKBB|RAC-BETA | 19 | TCRsignalingPathway |
| MAP3K8 | COT|EST|ESTF|FLJ10486|TPL2|Tpl-2|c-COT | 10 | TCRsignalingPathway |
| MAP3K14 | FTDCR1B|HS|HSNIK|NIK | 17 | TCRsignalingPathway |
| PDCD1 | CD279|PD1|SLEB2|hPD-1|hPD-l | 2 | TCRsignalingPathway |
| CTLA4 | CD|CD152|CELIAC3|CTLA-4|GSE|ICOS|IDDM12 | 2 | TCRsignalingPathway |
| PTPN6 | HCP|HCPH|HPTP1C|PTP-1C|SH-PTP1|SHP-1|SHP-1L|SHP1 | 12 | TCRsignalingPathway |
| CBLC | CBL-3|CBL-SL|RNF57 | 19 | TCRsignalingPathway |
| CBL | C-CBL|CBL2|RNF55 | 11 | TCRsignalingPathway |
| CBLB | DKFZp686J10223|DKFZp779A0729|DKFZp779F1443|FLJ36865|FLJ41152|Nbla00127|RNF56 | 3 | TCRsignalingPathway |
| IL2 | IL-2|TCGF|lymphokine | 4 | TCRsignalingPathway |
| IL4 | BCGF-1|BCGF1|BSF1|IL-4|MGC79402 | 5 | TCRsignalingPathway |
| IL5 | EDF|IL-5|TRF | 5 | TCRsignalingPathway |
| IL10 | CSIF|IL-10|IL10A|MGC126450|MGC126451|TGIF | 1 | TCRsignalingPathway |
| IFNG | IFG|IFI | 12 | TCRsignalingPathway |
| CSF2 | GMCSF|MGC131935|MGC138897 | 5 | TCRsignalingPathway |
| TNF | DIF|TNF-alpha|TNFA|TNFSF2 | 6 | TCRsignalingPathway |
| CDK4 | CMM3|MGC14458|PSK-J3 | 12 | TCRsignalingPathway |
| RASGRP1 | CALDAG-GEFI|CALDAG-GEFII|MGC129998|MGC129999|RASGRP|V|hRasGRP1 | 15 | TCRsignalingPathway |
| PDK1 | - | 2 | TCRsignalingPathway |
| PLCG1 | PLC-II|PLC1|PLC148|PLCgamma1 | 20 | TCRsignalingPathway |
| PRKCQ | MGC126514|MGC141919|PRKCT|nPKC-theta | 10 | TCRsignalingPathway |
| TRAC | - | 14 | TCRsignalingPathway |
| TRAJ1 | - | 14 | TCRsignalingPathway |
| TRAJ2 | - | 14 | TCRsignalingPathway |
| TRAJ3 | - | 14 | TCRsignalingPathway |
| TRAJ4 | - | 14 | TCRsignalingPathway |
| TRAJ5 | - | 14 | TCRsignalingPathway |
| TRAJ6 | - | 14 | TCRsignalingPathway |
| TRAJ7 | - | 14 | TCRsignalingPathway |
| TRAJ8 | - | 14 | TCRsignalingPathway |
| TRAJ9 | - | 14 | TCRsignalingPathway |
| TRAJ10 | - | 14 | TCRsignalingPathway |
| TRAJ11 | - | 14 | TCRsignalingPathway |
| TRAJ12 | - | 14 | TCRsignalingPathway |
| TRAJ13 | - | 14 | TCRsignalingPathway |
| TRAJ14 | - | 14 | TCRsignalingPathway |
| TRAJ15 | - | 14 | TCRsignalingPathway |
| TRAJ16 | - | 14 | TCRsignalingPathway |
| TRAJ17 | - | 14 | TCRsignalingPathway |
| TRAJ18 | - | 14 | TCRsignalingPathway |
| TRAJ19 | - | 14 | TCRsignalingPathway |
| TRAJ20 | - | 14 | TCRsignalingPathway |
| TRAJ21 | - | 14 | TCRsignalingPathway |
| TRAJ22 | - | 14 | TCRsignalingPathway |
| TRAJ23 | - | 14 | TCRsignalingPathway |
| TRAJ24 | - | 14 | TCRsignalingPathway |
| TRAJ25 | - | 14 | TCRsignalingPathway |
| TRAJ26 | - | 14 | TCRsignalingPathway |
| TRAJ27 | - | 14 | TCRsignalingPathway |
| TRAJ28 | - | 14 | TCRsignalingPathway |
| TRAJ29 | - | 14 | TCRsignalingPathway |
| TRAJ30 | - | 14 | TCRsignalingPathway |
| TRAJ31 | - | 14 | TCRsignalingPathway |
| TRAJ32 | - | 14 | TCRsignalingPathway |
| TRAJ33 | - | 14 | TCRsignalingPathway |
| TRAJ34 | - | 14 | TCRsignalingPathway |
| TRAJ35 | - | 14 | TCRsignalingPathway |
| TRAJ36 | - | 14 | TCRsignalingPathway |
| TRAJ37 | - | 14 | TCRsignalingPathway |
| TRAJ38 | - | 14 | TCRsignalingPathway |
| TRAJ39 | - | 14 | TCRsignalingPathway |
| TRAJ40 | - | 14 | TCRsignalingPathway |
| TRAJ41 | - | 14 | TCRsignalingPathway |
| TRAJ42 | - | 14 | TCRsignalingPathway |
| TRAJ43 | - | 14 | TCRsignalingPathway |
| TRAJ44 | - | 14 | TCRsignalingPathway |
| TRAJ45 | - | 14 | TCRsignalingPathway |
| TRAJ46 | - | 14 | TCRsignalingPathway |
| TRAJ47 | - | 14 | TCRsignalingPathway |
| TRAJ48 | - | 14 | TCRsignalingPathway |
| TRAJ49 | - | 14 | TCRsignalingPathway |
| TRAJ50 | - | 14 | TCRsignalingPathway |
| TRAJ52 | - | 14 | TCRsignalingPathway |
| TRAJ53 | - | 14 | TCRsignalingPathway |
| TRAJ54 | - | 14 | TCRsignalingPathway |
| TRAJ56 | - | 14 | TCRsignalingPathway |
| TRAJ57 | - | 14 | TCRsignalingPathway |
| TRAJ58 | - | 14 | TCRsignalingPathway |
| TRAJ59 | - | 14 | TCRsignalingPathway |
| TRAJ61 | - | 14 | TCRsignalingPathway |
| TRAV1-1 | TCRAV1S1|TCRAV7S1|TRAV11 | 14 | TCRsignalingPathway |
| TRAV1-2 | TCRAV1S2|TCRAV7S2|TRAV12 | 14 | TCRsignalingPathway |
| TRAV2 | TCRAV11S1|TCRAV2S1 | 14 | TCRsignalingPathway |
| TRAV3 | TCRAV16S1|TCRAV3S1 | 14 | TCRsignalingPathway |
| TRAV4 | TCRAV20S1|TCRAV4S1 | 14 | TCRsignalingPathway |
| TRAV5 | TCRAV15S1|TCRAV5S1 | 14 | TCRsignalingPathway |
| TRAV7 | TCRAV7S1 | 14 | TCRsignalingPathway |
| TRAV8-1 | TCRAV1S1|TCRAV8S1|TRAV81 | 14 | TCRsignalingPathway |
| TRAV8-2 | TCRAV1S5|TCRAV8S2|TRAV82 | 14 | TCRsignalingPathway |
| TRAV8-3 | TCRAV1S4|TCRAV8S3|TRAV83 | 14 | TCRsignalingPathway |
| TRAV8-4 | TCRAV1S2|TCRAV8S4|TRAV84 | 14 | TCRsignalingPathway |
| TRAV8-6 | TCRAV1S3|TCRAV8S6|TRAV86 | 14 | TCRsignalingPathway |
| TRAV8-7 | TCRAV8S7|TRAV87 | 14 | TCRsignalingPathway |
| TRAV9-1 | TCRAV9S1|TRAV91 | 14 | TCRsignalingPathway |
| TRAV9-2 | TCRAV22S1|TCRAV9S2|TRAV92 | 14 | TCRsignalingPathway |
| TRAV10 | TCRAV10S1|TCRAV24S1 | 14 | TCRsignalingPathway |
| TRAV12-1 | TCRAV12S1|TCRAV2S3|TRAV121 | 14 | TCRsignalingPathway |
| TRAV12-2 | TCRAV12S2|TCRAV2S1|TRAV122 | 14 | TCRsignalingPathway |
| TRAV12-3 | TCRAV12S3|TCRAV2S2|TRAV123 | 14 | TCRsignalingPathway |
| TRAV13-1 | TCRAV13S1|TCRAV8S1|TRAV131 | 14 | TCRsignalingPathway |
| TRAV13-2 | TCRAV13S2|TCRAV8S2|TRAV132 | 14 | TCRsignalingPathway |
| TRAV14DV4 | TCRAV6S1-hDV104S1|TRAV14/DV4|hADV14S1 | 14 | TCRsignalingPathway |
| TRAV16 | TCRAV16S1|TCRAV9S1 | 14 | TCRsignalingPathway |
| TRAV17 | TCRAV17S1|TCRAV3S1 | 14 | TCRsignalingPathway |
| TRAV18 | TCRAV18S1 | 14 | TCRsignalingPathway |
| TRAV19 | TCRAV12S1|TCRAV19S1 | 14 | TCRsignalingPathway |
| TRAV20 | TCRAV20S1|TCRAV30S1 | 14 | TCRsignalingPathway |
| TRAV21 | TCRAV21S1|TCRAV23S1 | 14 | TCRsignalingPathway |
| TRAV22 | TCRAV13S1|TCRAV22S1 | 14 | TCRsignalingPathway |
| TRAV23DV6 | TCRAV17S1|TRAV23/DV6|hADV23S1 | 14 | TCRsignalingPathway |
| TRAV24 | TCRAV18S1|TCRAV24S1 | 14 | TCRsignalingPathway |
| TRAV25 | TCRAV25S1|TCRAV32S1 | 14 | TCRsignalingPathway |
| TRAV26-1 | TCRAV26S1|TCRAV4S2|TRAV261 | 14 | TCRsignalingPathway |
| TRAV26-2 | TCRAV26S2|TCRAV4S1|TRAV262 | 14 | TCRsignalingPathway |
| TRAV27 | TCRAV10S1|TCRAV27S1 | 14 | TCRsignalingPathway |
| TRAV29DV5 | TCRAV21S1|TRAV29/DV5|hADV29S1 | 14 | TCRsignalingPathway |
| TRAV30 | TCRAV29S1|TCRAV30S1 | 14 | TCRsignalingPathway |
| TRAV34 | TCRAV26S1|TCRAV34S1 | 14 | TCRsignalingPathway |
| TRAV35 | TCRAV25S1|TCRAV35S1 | 14 | TCRsignalingPathway |
| TRAV36DV7 | TCRAV28S1|TRAV36/DV7|hADV36S1 | 14 | TCRsignalingPathway |
| TRAV38-1 | TCRAV14S2|TCRAV38S1|TRAV381 | 14 | TCRsignalingPathway |
| TRAV38-2DV8 | TCRAV14S1|TRAV382DV8|hADV38S2 | 14 | TCRsignalingPathway |
| TRAV39 | TCRAV27S1|TCRAV39S1 | 14 | TCRsignalingPathway |
| TRAV40 | TCRAV31S1|TCRAV40S1 | 14 | TCRsignalingPathway |
| TRAV41 | TCRAV19S1|TCRAV41S1 | 14 | TCRsignalingPathway |
| TRBC1 | BV05S1J2.2|MGC88817|TCRBC1 | 7 | TCRsignalingPathway |
| TRBC2 | TCRBC2 | 7 | TCRsignalingPathway |
| TRBD1 | TCRBD1 | 7 | TCRsignalingPathway |
| TRBD2 | TCRBD2 | 7 | TCRsignalingPathway |
| TRBJ1-1 | TCRBJ1S1|TRBJ11 | 7 | TCRsignalingPathway |
| TRBJ1-2 | TCRBJ1S2|TRBJ12 | 7 | TCRsignalingPathway |
| TRBJ1-3 | TCRBJ1S3|TRBJ13 | 7 | TCRsignalingPathway |
| TRBJ1-4 | TCRBJ1S4|TRBJ14 | 7 | TCRsignalingPathway |
| TRBJ1-5 | TCRBJ1S5|TRBJ15 | 7 | TCRsignalingPathway |
| TRBJ1-6 | TCRBJ1S6|TRBJ16 | 7 | TCRsignalingPathway |
| TRBJ2-1 | TCRBJ2S1|TRBJ21 | 7 | TCRsignalingPathway |
| TRBJ2-2 | TCRBJ2S2|TRBJ22 | 7 | TCRsignalingPathway |
| TRBJ2-3 | TCRBJ2S3|TRBJ23 | 7 | TCRsignalingPathway |
| TRBJ2-4 | TCRBJ2S4|TRBJ24 | 7 | TCRsignalingPathway |
| TRBJ2-5 | TCRBJ2S5|TRBJ25 | 7 | TCRsignalingPathway |
| TRBJ2-6 | TCRBJ2S6|TRBJ26 | 7 | TCRsignalingPathway |
| TRBJ2-7 | TCRBJ2S7|TRBJ27 | 7 | TCRsignalingPathway |
| TRBV2 | TCRBV22S1A2N1T|TCRBV2S1 | 7 | TCRsignalingPathway |
| TRBV3-1 | TCRBV3S1|TCRBV9S1A1T|TRBV31 | 7 | TCRsignalingPathway |
| TRBV4-1 | BV07S1J2.7|TCRBV4S1|TCRBV7S1A1N2T|TRBV41 | 7 | TCRsignalingPathway |
| TRBV4-2 | TCRBV4S2|TCRBV7S3A2T|TRBV42 | 7 | TCRsignalingPathway |
| TRBV4-3 | TCRBV4S3|TCRBV7S2A1N4T|TRBV43 | 7 | TCRsignalingPathway |
| TRBV5-1 | TCRBV5S1|TCRBV5S1A1T|TRBV51 | 7 | TCRsignalingPathway |
| TRBV5-4 | TCRBV5S4|TCRBV5S6A3N2T|TRBV54 | 7 | TCRsignalingPathway |
| TRBV5-5 | TCRBV5S3A2T|TCRBV5S5|TRBV55 | 7 | TCRsignalingPathway |
| TRBV5-6 | TCRBV5S2|TCRBV5S6|TRBV56 | 7 | TCRsignalingPathway |
| TRBV5-7 | TCRBV5S7|TCRBV5S7P|TRBV57 | 7 | TCRsignalingPathway |
| TRBV5-8 | TCRBV5S4A2T|TCRBV5S8|TRBV58 | 7 | TCRsignalingPathway |
| TRBV6-1 | TCRBV13S3|TCRBV6S1|TRBV61 | 7 | TCRsignalingPathway |
| TRBV6-2 | TCRBV13S2A1T|TCRBV6S2|TRBV62 | 7 | TCRsignalingPathway |
| TRBV6-3 | TCRBV13S9/13S2A1T|TCRBV6S3|TRBV63 | 7 | TCRsignalingPathway |
| TRBV6-4 | TCRBV13S5|TCRBV6S4|TRBV64 | 7 | TCRsignalingPathway |
| TRBV6-5 | TCRBV13S1|TCRBV6S5|TRBV65 | 7 | TCRsignalingPathway |
| TRBV6-6 | TCRBV13S6A2T|TCRBV6S6|TRBV66 | 7 | TCRsignalingPathway |
| TRBV6-7 | TCRBV13S8P|TCRBV6S7|TRBV67 | 7 | TCRsignalingPathway |
| TRBV6-8 | TCRBV13S7P|TCRBV6S8|TRBV68 | 7 | TCRsignalingPathway |
| TRBV6-9 | TCRBV13S4|TCRBV6S9|TRBV69 | 7 | TCRsignalingPathway |
| TRBV7-2 | MGC117435|TCRBV6S5A1N1|TCRBV7S2|TRBV72 | 7 | TCRsignalingPathway |
| TRBV7-3 | TCRBV6S1A1N1|TCRBV7S3|TRBV73 | 7 | TCRsignalingPathway |
| TRBV7-4 | TCRBV6S8A2T|TCRBV7S4|TRBV74 | 7 | TCRsignalingPathway |
| TRBV7-6 | TCRBV6S3A1N1T|TCRBV7S6|TRBV76 | 7 | TCRsignalingPathway |
| TRBV7-7 | TCRBV6S6A2T|TCRBV7S7|TRBV77 | 7 | TCRsignalingPathway |
| TRBV7-8 | TCRBV6S2A1N1T|TCRBV7S8|TRBV78 | 7 | TCRsignalingPathway |
| TRBV7-9 | TCRBV6S4A1|TCRBV7S9|TRBV79 | 7 | TCRsignalingPathway |
| TRBV9 | TCRBV1S1A1N1|TCRBV9S1 | 7 | TCRsignalingPathway |
| TRBV10-1 | TCRBV10S1|TCRBV12S2|TCRBV12S2A1T|TRBV101 | 7 | TCRsignalingPathway |
| TRBV10-2 | TCRBV10S2|TCRBV12S3|TRBV102 | 7 | TCRsignalingPathway |
| TRBV10-3 | TCRBV10S3|TCRBV12S1A1N2|TRBV103 | 7 | TCRsignalingPathway |
| TRBV11-1 | TCRBV11S1|TCRBV21S1|TRBV111 | 7 | TCRsignalingPathway |
| TRBV11-2 | TCRBV11S2|TCRBV21S3A2N2T|TRBV112 | 7 | TCRsignalingPathway |
| TRBV11-3 | TCRBV11S3|TCRBV21S2A2|TRBV113 | 7 | TCRsignalingPathway |
| TRBV12-3 | TCRBV12S3|TCRBV8S1|TRBV123 | 7 | TCRsignalingPathway |
| TRBV12-4 | TCRBV12S4|TCRBV8S2A1T|TRBV124 | 7 | TCRsignalingPathway |
| TRBV12-5 | TCRBV12S5|TCRBV8S3|TRBV125 | 7 | TCRsignalingPathway |
| TRBV13 | TCRBV13S1|TCRBV23S1A2T | 7 | TCRsignalingPathway |
| TRBV14 | TCRBV14S1|TCRBV16S1A1N1 | 7 | TCRsignalingPathway |
| TRBV15 | TCRBV15S1|TCRBV24S1A3T | 7 | TCRsignalingPathway |
| TRBV16 | TCRBV16S1|TCRBV25S1A2PT | 7 | TCRsignalingPathway |
| TRBV17 | TCRBV17S1|TCRBV26S1P | 7 | TCRsignalingPathway |
| TRBV18 | TCRBV18S1 | 7 | TCRsignalingPathway |
| TRBV19 | TCRBV17S1A1T|TCRBV19S1 | 7 | TCRsignalingPathway |
| TRBV20-1 | TCRBV20S1|TCRBV2S1|TRBV201 | 7 | TCRsignalingPathway |
| TRBV24-1 | TCRBV15S1|TCRBV24S1|TRBV241 | 7 | TCRsignalingPathway |
| TRBV25-1 | TCRBV11S1A1T|TCRBV25S1|TRBV251 | 7 | TCRsignalingPathway |
| TRBV27 | FLJ35984|TCRBV14S1|TCRBV27S1 | 7 | TCRsignalingPathway |
| TRBV28 | TCRBV28S1|TCRBV3S1 | 7 | TCRsignalingPathway |
| TRBV29-1 | TCRBV29S1|TCRBV4S1A1T|TRBV291 | 7 | TCRsignalingPathway |
| TRBV30 | TCRBV20S1A1N2|TCRBV30S1 | 7 | TCRsignalingPathway |
| TRDC | - | 14 | TCRsignalingPathway |
| TRDD1 | - | 14 | TCRsignalingPathway |
| TRDD2 | - | 14 | TCRsignalingPathway |
| TRDD3 | TCRD | 14 | TCRsignalingPathway |
| TRDJ1 | TCRD | 14 | TCRsignalingPathway |
| TRDJ2 | - | 14 | TCRsignalingPathway |
| TRDJ3 | - | 14 | TCRsignalingPathway |
| TRDJ4 | - | 14 | TCRsignalingPathway |
| TRDV1 | hDV101S1 | 14 | TCRsignalingPathway |
| TRDV2 | MGC117421|hDV102S1 | 14 | TCRsignalingPathway |
| TRDV3 | hDV103S1 | 14 | TCRsignalingPathway |
| TRGV9 | MGC47828|TCRGV9|V2 | 7 | TCRsignalingPathway |
| TRGV8 | TCRGV8|V1S8 | 7 | TCRsignalingPathway |
| TRGV5 | TCRGV5|V1S5 | 7 | TCRsignalingPathway |
| TRGV4 | TCRGV4|V1S4 | 7 | TCRsignalingPathway |
| TRGV3 | TCRGV3|V1S3 | 7 | TCRsignalingPathway |
| TRGV2 | MGC42817|TCRGV2|VIS2 | 7 | TCRsignalingPathway |
| TRGJP2 | JP2|TCRGJP2 | 7 | TCRsignalingPathway |
| TRGJP1 | JP1|TCRGJP1 | 7 | TCRsignalingPathway |
| TRGJP | JP|TCRGJP | 7 | TCRsignalingPathway |
| TRGJ2 | J2|TCRGJ2 | 7 | TCRsignalingPathway |
| TRGJ1 | J1|TCRGJ1 | 7 | TCRsignalingPathway |
| TRGC2 | TCRGC2|TRGC2(2X)|TRGC2(3X) | 7 | TCRsignalingPathway |
| TRGC1 | C1|TCRGC1 | 7 | TCRsignalingPathway |
| TRAV6 | TCRAV5S1|TCRAV6S1 | 14 | TCRsignalingPathway |
| BMP1 | FLJ44432|PCOLC|PCP|TLD|pCP-2 | 8 | TGFb_Family_Member |
| BMP10 | MGC126783 | 2 | TGFb_Family_Member |
| BMP15 | GDF9B|ODG2|POF4 | X | TGFb_Family_Member |
| BMP2 | BMP2A | 20 | TGFb_Family_Member |
| BMP3 | BMP-3A | 4 | TGFb_Family_Member |
| BMP4 | BMP2B|BMP2B1|MCOPS6|OFC11|ZYME | 14 | TGFb_Family_Member |
| BMP5 | MGC34244 | 6 | TGFb_Family_Member |
| BMP6 | VGR|VGR1 | 6 | TGFb_Family_Member |
| BMP7 | OP-1 | 20 | TGFb_Family_Member |
| BMP8A | FLJ14351|FLJ45264 | 1 | TGFb_Family_Member |
| BMP8B | BMP8|MGC131757|OP2 | 1 | TGFb_Family_Member |
| GDF1 | - | 19 | TGFb_Family_Member |
| GDF10 | BMP-3b|BMP3B | 10 | TGFb_Family_Member |
| GDF11 | BMP-11|BMP11 | 12 | TGFb_Family_Member |
| GDF15 | GDF-15|MIC-1|MIC1|NAG-1|PDF|PLAB|PTGFB | 19 | TGFb_Family_Member |
| GDF2 | BMP-9|BMP9 | 10 | TGFb_Family_Member |
| GDF3 | - | 12 | TGFb_Family_Member |
| GDF5 | BMP14|CDMP1|LAP4|OS5|SYNS2 | 20 | TGFb_Family_Member |
| GDF6 | BMP13|CDMP2|KFS|KFSL|MGC158100|MGC158101|SGM1 | 8 | TGFb_Family_Member |
| GDF7 | BMP12 | 2 | TGFb_Family_Member |
| GDF9 | - | 5 | TGFb_Family_Member |
| GDNF | ATF1|ATF2|HFB1-GDNF | 5 | TGFb_Family_Member |
| INHA | - | 2 | TGFb_Family_Member |
| INHBA | EDF|FRP | 7 | TGFb_Family_Member |
| INHBB | MGC157939 | 2 | TGFb_Family_Member |
| INHBC | IHBC | 12 | TGFb_Family_Member |
| INHBE | MGC4638 | 12 | TGFb_Family_Member |
| LEFTY1 | LEFTB|LEFTYB | 1 | TGFb_Family_Member |
| LEFTY2 | EBAF|LEFTA|LEFTYA|MGC46222|TGFB4 | 1 | TGFb_Family_Member |
| NODAL | MGC138230 | 10 | TGFb_Family_Member |
| TGFB1 | CED|DPD1|TGFB|TGFbeta | 19 | TGFb_Family_Member |
| TGFB2 | MGC116892|TGF-beta2 | 1 | TGFb_Family_Member |
| TGFB3 | ARVD|FLJ16571|TGF-beta3 | 14 | TGFb_Family_Member |
| ACVR1B | ACTRIB|ACVRLK4|ALK4|SKR2 | 12 | TGFb_Family_Member_Receptor |
| ACVR1C | ACVRLK7|ALK7 | 2 | TGFb_Family_Member_Receptor |
| ACVR2A | ACTRII|ACVR2 | 2 | TGFb_Family_Member_Receptor |
| ACVR2B | ACTRIIB|ActR-IIB|MGC116908 | 3 | TGFb_Family_Member_Receptor |
| ACVRL1 | ACVRLK1|ALK-1|ALK1|HHT|HHT2|ORW2|SKR3|TSR-I | 12 | TGFb_Family_Member_Receptor |
| AMHR2 | AMHR|MISR2|MISRII | 12 | TGFb_Family_Member_Receptor |
| BMPR1A | 10q23del|ACVRLK3|ALK3|CD292|SKR5 | 10 | TGFb_Family_Member_Receptor |
| BMPR1B | ALK-6|ALK6|CDw293 | 4 | TGFb_Family_Member_Receptor |
| BMPR2 | BMPR-II|BMPR3|BMR2|BRK-3|FLJ41585|FLJ76945|PPH1|T-ALK | 2 | TGFb_Family_Member_Receptor |
| TGFBR1 | AAT5|ACVRLK4|ALK-5|ALK5|LDS1A|LDS2A|SKR4|TGFR-1 | 9 | TGFb_Family_Member_Receptor |
| TGFBR2 | AAT3|FAA3|LDS1B|LDS2B|MFS2|RIIC|TAAD2|TGFR-2|TGFbeta-RII | 3 | TGFb_Family_Member_Receptor |
| TGFBR3 | BGCAN|betaglycan | 1 | TGFb_Family_Member_Receptor |
| TNFRSF11B | MGC29565|OCIF|OPG|TR1 | 8 | TNF_Family_Members |
| TNFSF10 | APO2L|Apo-2L|CD253|TL2|TRAIL | 3 | TNF_Family_Members |
| TNFSF11 | CD254|ODF|OPGL|OPTB2|RANKL|TRANCE|hRANKL2|sOdf | 13 | TNF_Family_Members |
| TNFSF12 | APO3L|DR3LG|MGC129581|MGC20669|TWEAK | 17 | TNF_Family_Members |
| TNFSF13 | APRIL|CD256|TALL2|TRDL-1|UNQ383/PRO715|ligand | 17 | TNF_Family_Members |
| TNFSF13B | BAFF|BLYS|CD257|DTL|TALL-1|TALL1|THANK|TNFSF20|ZTNF4 | 13 | TNF_Family_Members |
| TNFSF14 | CD258|HVEML|LIGHT|LTg|TR2 | 19 | TNF_Family_Members |
| TNFSF15 | MGC129934|MGC129935|TL1|TL1A|VEGI|VEGI192A | 9 | TNF_Family_Members |
| TNFSF18 | AITRL|GITRL|MGC138237|TL6|hGITRL | 1 | TNF_Family_Members |
| TNFSF4 | CD134L|CD252|GP34|OX-40L|OX4OL|TXGP1 | 1 | TNF_Family_Members |
| TNFSF8 | CD153|CD30L|CD30LG|MGC138144 | 9 | TNF_Family_Members |
| TNFSF9 | 4-1BB-L|CD137L | 19 | TNF_Family_Members |
| TNFRSF10B | CD262|DR5|KILLER|KILLER/DR5|TRAIL-R2|TRAILR2|TRICK2|TRICK2A|TRICK2B|TRICKB|ZTNFR9 | 8 | TNF_Family_Members_Receptors |
| TNFRSF10C | CD263|DCR1|LIT|MGC149501|MGC149502|TRAILR3|TRID | 8 | TNF_Family_Members_Receptors |
| TNFRSF10D | CD264|DCR2|TRAILR4|TRUNDD | 8 | TNF_Family_Members_Receptors |
| TNFRSF11A | CD265|FEO|LOH18CR1|ODFR|OFE|OPTB7|OSTS|PDB2|RANK|TRANCER | 18 | TNF_Family_Members_Receptors |
| TNFRSF12A | CD266|FN14|TWEAKR | 16 | TNF_Family_Members_Receptors |
| TNFRSF13B | CD267|CVID|FLJ39942|MGC133214|MGC39952|TACI|TNFRSF14B | 17 | TNF_Family_Members_Receptors |
| TNFRSF13C | BAFF-R|BAFFR|CD268|MGC138235 | 22 | TNF_Family_Members_Receptors |
| TNFRSF14 | ATAR|HVEA|HVEM|LIGHTR|TR2 | 1 | TNF_Family_Members_Receptors |
| TNFRSF17 | BCM|BCMA|CD269 | 16 | TNF_Family_Members_Receptors |
| TNFRSF18 | AITR|GITR|GITR-D | 1 | TNF_Family_Members_Receptors |
| TNFRSF19 | TAJ|TAJ-alpha|TRADE|TROY | 13 | TNF_Family_Members_Receptors |
| TNFRSF1A | CD120a|FPF|MGC19588|TBP1|TNF-R|TNF-R-I|TNF-R55|TNFAR|TNFR1|TNFR55|TNFR60|p55|p55-R|p60 | 12 | TNF_Family_Members_Receptors |
| TNFRSF1B | CD120b|TBPII|TNF-R-II|TNF-R75|TNFBR|TNFR1B|TNFR2|TNFR80|p75|p75TNFR | 1 | TNF_Family_Members_Receptors |
| TNFRSF21 | BM-018|DR6|MGC31965 | 6 | TNF_Family_Members_Receptors |
| TNFRSF25 | APO-3|DDR3|DR3|LARD|TNFRSF12|TR3|TRAMP|WSL-1|WSL-LR | 1 | TNF_Family_Members_Receptors |
| TNFRSF4 | ACT35|CD134|OX40|TXGP1L | 1 | TNF_Family_Members_Receptors |
| TNFRSF6B | DCR3|DJ583P15.1.1|M68|TR6 | 20 | TNF_Family_Members_Receptors |
| TNFRSF8 | CD30|D1S166E|Ki-1 | 1 | TNF_Family_Members_Receptors |
| TNFRSF9 | 4-1BB|CD137|CDw137|ILA|MGC2172 | 1 | TNF_Family_Members_Receptors |
|  |  |  |  |
