# Supplementary material for: A novel immune-related prognostic signature in epithelial ovarian carcinoma
Source: Aging (Albany NY). 2021 Apr 4;13(7):10289–311. doi: 10.18632/aging.202792 (PMC8064207; doi:10.18632/aging.202792)
Supplement: Supplementary Table 2 [file aging-13-202792-s003.doc]

Supplementary Table 2. The table of transcription factors from cistrome cancer database.

| ADNP |
| --- |
| AFF4 |
| AR |
| ARID3A |
| ARNT |
| ARNTL |
| ASCL1 |
| ASH2L |
| ATF1 |
| ATF2 |
| ATF3 |
| ATF4 |
| ATF7 |
| BACH1 |
| BACH2 |
| BATF |
| BCL11A |
| BCL3 |
| BCL6 |
| BDP1 |
| BHLHE40 |
| BMI1 |
| BRCA1 |
| BRD1 |
| BRD2 |
| BRD3 |
| BRD4 |
| BRF1 |
| BRF2 |
| C17orf96 |
| CBFB |
| CBX2 |
| CBX3 |
| CBX5 |
| CBX7 |
| CBX8 |
| CDK2 |
| CDK7 |
| CDK8 |
| CDK9 |
| CDX2 |
| CEBPA |
| CEBPB |
| CENPA |
| CHD1 |
| CHD2 |
| CHD7 |
| CHD8 |
| CIITA |
| CPSF3L |
| CREBBP |
| CTNNB1 |
| CUX1 |
| DNMT1 |
| DNMT3A |
| DYRK1A |
| E2F1 |
| E2F3 |
| E2F4 |
| E2F6 |
| E2F7 |
| EBF1 |
| EED |
| EGR1 |
| EGR2 |
| EHF |
| EHMT2 |
| ELF1 |
| ELF5 |
| ELK1 |
| ELK4 |
| ELL2 |
| EMX1 |
| EOMES |
| EP400 |
| EPAS1 |
| EPO |
| ERCC6 |
| ERG |
| ESR1 |
| ESRRA |
| ETS1 |
| ETV1 |
| EZH1 |
| EZH2 |
| FLI1 |
| FOS |
| FOSL1 |
| FOSL2 |
| FOXA1 |
| FOXA2 |
| FOXK1 |
| FOXM1 |
| FOXO1 |
| FOXO3 |
| FOXP1 |
| FOXP2 |
| FOXP3 |
| GABPA |
| GATA2 |
| GATA3 |
| GATA4 |
| GATA6 |
| GATAD1 |
| GREB1 |
| GRHL2 |
| GTF2B |
| GTF2F1 |
| GTF2I |
| H2AFX |
| HCFC1 |
| HDAC1 |
| HDAC2 |
| HDAC3 |
| HDAC6 |
| HEY1 |
| HIF1A |
| HIRA |
| HNF1B |
| HNF4A |
| HNF4G |
| HOXA9 |
| HOXB13 |
| HOXB7 |
| HOXC11 |
| HOXC9 |
| HSF1 |
| HSF2 |
| IKZF1 |
| IRF1 |
| IRF3 |
| IRF4 |
| IRF5 |
| JARID2 |
| JMJD1C |
| JMJD6 |
| JUN |
| JUNB |
| JUND |
| KAT2B |
| KAT5 |
| KDM1A |
| KDM2B |
| KDM3A |
| KDM4C |
| KDM5A |
| KDM5B |
| KDM5C |
| KDM6B |
| KLF11 |
| KLF4 |
| KLF5 |
| LEF1 |
| LHX2 |
| LIN9 |
| LMNA |
| LMNB1 |
| LMO2 |
| LYL1 |
| MAF |
| MAFF |
| MAFK |
| MAX |
| MAZ |
| MBD2 |
| MBD3 |
| MECP2 |
| MED12 |
| MEF2A |
| MEF2B |
| MEF2C |
| MEIS1 |
| MITF |
| MXI1 |
| MYB |
| MYBL2 |
| MYC |
| MYH11 |
| NANOG |
| NCAPG |
| NCOR2 |
| NFATC1 |
| NFE2 |
| NFIC |
| NFYA |
| NFYB |
| NIPBL |
| NOTCH1 |
| NR1H2 |
| NR2C2 |
| NR2F1 |
| NR2F2 |
| NR3C1 |
| NR4A1 |
| NR5A2 |
| NRF1 |
| OGT |
| PAF1 |
| PAX3 |
| PAX5 |
| PAX6 |
| PBX1 |
| PBX3 |
| PDX1 |
| PHF8 |
| PIAS1 |
| PML |
| POLR2B |
| POLR3A |
| POLR3D |
| POLR3G |
| POU2F1 |
| POU5F1 |
| PPARD |
| PPARG |
| PRDM1 |
| PRKDC |
| RAG1 |
| RARA |
| RARG |
| RB1 |
| RBBP5 |
| RBL2 |
| RBP2 |
| RBPJ |
| RCOR1 |
| RELA |
| RFX2 |
| RFX5 |
| RING1 |
| RNF2 |
| RUNX1 |
| RUNX1T1 |
| RXRA |
| RXRG |
| RYBP |
| SALL4 |
| SAP30 |
| SCML2 |
| SETDB1 |
| SF1 |
| SFMBT1 |
| SFPQ |
| SIN3A |
| SIRT6 |
| SIX5 |
| SMAD1 |
| SMAD2 |
| SMAD3 |
| SMAD4 |
| SMARCA4 |
| SMARCB1 |
| SMARCC1 |
| SMARCC2 |
| SMC1A |
| SMC3 |
| SNAI2 |
| SNAPC2 |
| SNAPC4 |
| SOX17 |
| SOX2 |
| SOX4 |
| SOX9 |
| SP2 |
| SPDEF |
| SPIB |
| SRC |
| SREBF1 |
| SREBF2 |
| SRF |
| SSRP1 |
| STAT1 |
| STAT2 |
| STAT3 |
| STAT4 |
| STAT5A |
| STAT5B |
| STAT6 |
| SUMO1 |
| SUMO2 |
| SUPT5H |
| TAF1 |
| TAL1 |
| TAT |
| TBL1XR1 |
| TBP |
| TCF12 |
| TCF21 |
| TCF7 |
| TCF7L1 |
| TCF7L2 |
| TEAD1 |
| TEAD4 |
| TERF1 |
| TERF2 |
| TET2 |
| TFAP2A |
| TFAP2C |
| THAP11 |
| TP53 |
| TP63 |
| TP73 |
| TRIM28 |
| TTF2 |
| UBTF |
| USF1 |
| USF2 |
| VDR |
| VEZF1 |
| WDR5 |
| WHSC1 |
| WWTR1 |
| XBP1 |
| XRN2 |
| YAP1 |
| YY1 |
| ZBTB17 |
| ZBTB33 |
|  |
