# Supplementary material for: A novel immune-related prognostic signature in epithelial ovarian carcinoma
Source: Aging (Albany NY). 2021 Apr 4;13(7):10289–311. doi: 10.18632/aging.202792 (PMC8064207; doi:10.18632/aging.202792)
Supplement: Supplementary Table 3 [file aging-13-202792-s004.doc]

Supplementary Table 3. The table of differentially expressed immune-related genes.

| Symbol | Description | logFC | P.Value | Category |
| --- | --- | --- | --- | --- |
| CD1A | T-cell surface glycoprotein CD1a | 5.51655819 | 3.93E-37 | Antigen_Processing_and_Presentation |
| CTSE | Cathepsin E | 4.98939942 | 1.59E-50 | Antigen_Processing_and_Presentation |
| HLA-DQA2 | HLA class II histocompatibility antigen, DQ alpha 2 chain | 4.21770922 | 9.90E-29 | Antigen_Processing_and_Presentation |
| RAET1L | UL16-binding protein 6 | 4.21203753 | 9.59E-32 | Antigen_Processing_and_Presentation |
| CD1E | T-cell surface glycoprotein CD1e, membrane-associated | 4.04369791 | 8.40E-25 | Antigen_Processing_and_Presentation |
| ULBP2 | UL16-binding protein 2 | 3.91757135 | 8.20E-42 | Antigen_Processing_and_Presentation |
| HLA-DRA | HLA class II histocompatibility antigen, DR alpha chain | 3.48590857 | 7.73E-55 | Antigen_Processing_and_Presentation |
| IFI30 | Gamma-interferon-inducible lysosomal thiol reductase | 3.46682685 | 1.32E-74 | Antigen_Processing_and_Presentation |
| HLA-DQA1 | Major histocompatibility complex, class II, DQ alpha 1 | 3.42977149 | 2.66E-34 | Antigen_Processing_and_Presentation |
| CD74 | HLA class II histocompatibility antigen gamma chain | 3.22459532 | 2.46E-53 | Antigen_Processing_and_Presentation |
| IFNG | Interferon gamma | 3.1390561 | 4.50E-17 | Antigen_Processing_and_Presentation |
| HLA-DMB | HLA class II histocompatibility antigen, DM beta chain | 3.08584607 | 3.63E-53 | Antigen_Processing_and_Presentation |
| HLA-DQB1 | HLA class II histocompatibility antigen, DQ beta 1 chain | 3.08557572 | 1.35E-31 | Antigen_Processing_and_Presentation |
| HLA-DPA1 | HLA class II histocompatibility antigen, DP alpha 1 chain | 3.0173932 | 1.31E-40 | Antigen_Processing_and_Presentation |
| CD1B | T-cell surface glycoprotein CD1b | 2.88053453 | 2.65E-13 | Antigen_Processing_and_Presentation |
| FCER1G | High affinity immunoglobulin epsilon receptor subunit gamma | 2.82018798 | 1.20E-48 | Antigen_Processing_and_Presentation |
| HLA-DRB1 | HLA class II histocompatibility antigen, DRB1-15 beta chain | 2.7949143 | 4.82E-39 | Antigen_Processing_and_Presentation |
| KIR2DL4 | Killer cell immunoglobulin-like receptor 2DL4 | 2.76843284 | 9.97E-18 | Antigen_Processing_and_Presentation |
| CD1C | T-cell surface glycoprotein CD1c | 2.747088 | 5.37E-18 | Antigen_Processing_and_Presentation |
| HLA-DOA | HLA class II histocompatibility antigen, DO alpha chain | 2.60558235 | 3.89E-29 | Antigen_Processing_and_Presentation |
| CD8B | T-cell surface glycoprotein CD8 beta chain | 2.59512493 | 5.38E-22 | Antigen_Processing_and_Presentation |
| HLA-DRB5 | HLA class II histocompatibility antigen, DR beta 5 chain | 2.54825192 | 1.61E-17 | Antigen_Processing_and_Presentation |
| ULBP1 | UL16-binding protein 1 | 2.54589223 | 4.17E-23 | Antigen_Processing_and_Presentation |
| KIR2DL3 | Killer cell immunoglobulin-like receptor 2DL3 | 2.42061523 | 6.21E-12 | Antigen_Processing_and_Presentation |
| HLA-DMA | HLA class II histocompatibility antigen, DM alpha chain | 2.40226138 | 3.00E-37 | Antigen_Processing_and_Presentation |
| KIR3DL2 | Killer cell immunoglobulin-like receptor 3DL2 | 2.39632139 | 4.38E-12 | Antigen_Processing_and_Presentation |
| ULBP3 | UL16-binding protein 3 | 2.35570942 | 1.14E-30 | Antigen_Processing_and_Presentation |
| HLA-G | HLA class I histocompatibility antigen, alpha chain G | 2.26043865 | 1.81E-19 | Antigen_Processing_and_Presentation |
| CTSS | Cathepsin S | 2.18461394 | 9.35E-27 | Antigen_Processing_and_Presentation |
| HLA-DPB1 | HLA class II histocompatibility antigen, DP beta 1 chain | 2.07433024 | 1.34E-27 | Antigen_Processing_and_Presentation |
| THBS1 | Thrombospondin-1 | -2.0984231 | 2.80E-26 | Antigen_Processing_and_Presentation |
| RAET1G | UL-16 binding protein 5 | -2.4068092 | 2.37E-27 | Antigen_Processing_and_Presentation |
| MICA | MHC class I polypeptide-related sequence A | -2.438817 | 7.75E-61 | Antigen_Processing_and_Presentation |
| CLEC4M | C-type lectin domain family 4 member M | -6.0271735 | 1.17E-45 | Antigen_Processing_and_Presentation |
| CRABP1 | Cellular retinoic acid-binding protein 1 | 10.6518257 | 3.97E-94 | Antimicrobials |
| LCN2 | Neutrophil gelatinase-associated lipocalin | 8.94954823 | 2.15E-132 | Antimicrobials |
| CXCL11 | C-X-C motif chemokine 11 | 8.83368701 | 9.81E-114 | Antimicrobials |
| DEFB1 | Beta-defensin 1 | 8.81819743 | 2.40E-91 | Antimicrobials |
| S100A5 | Protein S100-A5 | 8.78751987 | 2.58E-129 | Antimicrobials |
| CLDN4 | Claudin-4 | 8.73739606 | 1.50E-227 | Antimicrobials |
| OBP2A | Odorant-binding protein 2a | 8.71185429 | 2.97E-62 | Antimicrobials |
| WFDC2 | WAP four-disulfide core domain protein 2 | 8.51402194 | 6.97E-178 | Antimicrobials |
| SLPI | Antileukoproteinase | 8.47160065 | 2.64E-152 | Antimicrobials |
| PAEP | Glycodelin | 8.39980846 | 2.83E-70 | Antimicrobials |
| S100A1 | Protein S100-A1 | 8.21782461 | 3.70E-137 | Antimicrobials |
| MMP12 | Macrophage metalloelastase | 7.77570689 | 1.37E-68 | Antimicrobials |
| CCL20 | C-C motif chemokine 20 | 7.54246 | 1.88E-81 | Antimicrobials |
| CXCL10 | C-X-C motif chemokine 10 | 7.53567243 | 2.05E-96 | Antimicrobials |
| CCL7 | C-C motif chemokine 7 | 7.19831108 | 3.70E-75 | Antimicrobials |
| CST4 | Cystatin-S | 6.80079701 | 1.96E-51 | Antimicrobials |
| FABP6 | Gastrotropin | 6.78102677 | 1.16E-44 | Antimicrobials |
| PI3 | Elafin | 6.76341102 | 5.12E-77 | Antimicrobials |
| LCN1 | Lipocalin-1 | 6.74248106 | 4.39E-49 | Antimicrobials |
| CRABP2 | Cellular retinoic acid-binding protein 2 | 6.71830067 | 5.53E-144 | Antimicrobials |
| TNF | Tumor necrosis factor | 6.65018093 | 1.89E-96 | Antimicrobials |
| DEFB126 | Beta-defensin 126 | 6.4381229 | 1.40E-31 | Antimicrobials |
| CCL17 | C-C motif chemokine 17 | 6.26603686 | 8.87E-56 | Antimicrobials |
| CCR8 | C-C chemokine receptor type 8 | 6.18565755 | 1.07E-73 | Antimicrobials |
| BIRC5 | Baculoviral IAP repeat containing 5 | 6.09730511 | 2.49E-163 | Antimicrobials |
| PDYN | Proenkephalin-B | 6.08915543 | 5.97E-40 | Antimicrobials |
| CCL25 | C-C motif chemokine 25 | 5.88417463 | 7.70E-58 | Antimicrobials |
| CXCL9 | C-X-C motif chemokine 9 | 5.81708271 | 4.66E-57 | Antimicrobials |
| S100A14 | Protein S100-A14 | 5.50920952 | 6.99E-118 | Antimicrobials |
| CCL18 | C-C motif chemokine 18 | 5.27297929 | 4.55E-40 | Antimicrobials |
| OLR1 | Oxidized low-density lipoprotein receptor 1 | 5.23787401 | 3.13E-75 | Antimicrobials |
| MMP9 | Matrix metalloproteinase-9 | 5.13743864 | 8.35E-67 | Antimicrobials |
| IDO1 | Indoleamine 2,3-dioxygenase 1 | 5.12103362 | 1.31E-62 | Antimicrobials |
| TNFSF11 | Tumor necrosis factor ligand superfamily member 11 | 5.09881557 | 4.66E-50 | Antimicrobials |
| COLEC10 | Collectin-10 | 5.08103367 | 6.83E-62 | Antimicrobials |
| F2RL1 | Proteinase-activated receptor 2 | 4.9582157 | 8.72E-122 | Antimicrobials |
| XCL2 | Cytokine SCM-1 beta | 4.90766343 | 2.91E-39 | Antimicrobials |
| CDH1 | Cadherin-1 | 4.86708844 | 1.06E-119 | Antimicrobials |
| OASL | 2'-5'-oligoadenylate synthase-like protein | 4.86271861 | 1.50E-80 | Antimicrobials |
| IL1A | Interleukin-1 alpha | 4.85686357 | 1.95E-63 | Antimicrobials |
| UMODL1 | Uromodulin like 1 | 4.81035964 | 1.35E-49 | Antimicrobials |
| CXCL5 | C-X-C motif chemokine 5 | 4.61241948 | 2.83E-37 | Antimicrobials |
| CCL8 | C-C motif chemokine 8 | 4.55658094 | 2.73E-59 | Antimicrobials |
| OBP2B | Odorant-binding protein 2b | 4.40687639 | 3.95E-23 | Antimicrobials |
| PGLYRP4 | Peptidoglycan recognition protein 4 | 4.38806109 | 1.51E-26 | Antimicrobials |
| S100A2 | Protein S100-A2 | 4.34465346 | 2.27E-64 | Antimicrobials |
| SPAG11B | Sperm-associated antigen 11A | 4.31835722 | 2.49E-15 | Antimicrobials |
| CXCL6 | C-X-C motif chemokine 6 | 4.1766771 | 3.84E-33 | Antimicrobials |
| CCL11 | Eotaxin | 4.0965963 | 7.59E-21 | Antimicrobials |
| FASLG | Tumor necrosis factor ligand superfamily member 6 | 4.04605905 | 6.44E-37 | Antimicrobials |
| CXCL13 | C-X-C motif chemokine 13 | 4.01619318 | 7.54E-27 | Antimicrobials |
| ISG15 | Ubiquitin-like protein ISG15 | 4.01612916 | 3.61E-65 | Antimicrobials |
| PLAU | Urokinase-type plasminogen activator | 4.00813837 | 1.57E-62 | Antimicrobials |
| IL1B | Interleukin-1 beta | 3.9039891 | 2.21E-55 | Antimicrobials |
| PF4V1 | Platelet factor 4 variant | 3.84381198 | 1.66E-18 | Antimicrobials |
| CCL13 | C-C motif chemokine 13 | 3.82697354 | 1.95E-23 | Antimicrobials |
| CCR6 | C-C Motif Chemokine Receptor 6 | 3.75727727 | 3.60E-25 | Antimicrobials |
| PGLYRP2 | N-acetylmuramoyl-L-alanine amidase | 3.69077407 | 1.98E-36 | Antimicrobials |
| TLR7 | Toll-like receptor 7 | 3.67749204 | 4.99E-53 | Antimicrobials |
| XCL1 | Lymphotactin | 3.66314679 | 5.48E-47 | Antimicrobials |
| CCR3 | C-C chemokine receptor type 3 | 3.63049758 | 3.21E-30 | Antimicrobials |
| CCL22 | C-C motif chemokine 22 | 3.50908887 | 6.30E-39 | Antimicrobials |
| CCL3 | C-C motif chemokine 3 | 3.39132498 | 5.23E-50 | Antimicrobials |
| VDR | Vitamin D3 receptor | 3.37110259 | 1.77E-69 | Antimicrobials |
| AQP9 | Aquaporin-9 | 3.32805475 | 9.82E-40 | Antimicrobials |
| IFNB1 | Interferon beta | 3.32404866 | 4.88E-19 | Antimicrobials |
| CCL28 | C-C motif chemokine 28 | 3.31035993 | 3.57E-60 | Antimicrobials |
| CXCR4 | C-X-C chemokine receptor type 4 | 3.30538366 | 1.88E-69 | Antimicrobials |
| PF4 | Platelet factor 4 | 3.30360169 | 6.84E-16 | Antimicrobials |
| SYTL1 | Synaptotagmin-like protein 1 | 3.22170001 | 1.19E-78 | Antimicrobials |
| KAL1 | Anosmin-1 | 3.21670835 | 3.65E-46 | Antimicrobials |
| S100A11 | Protein S100-A11 | 3.1606426 | 1.58E-72 | Antimicrobials |
| IL18 | Interleukin-18 | 3.06717585 | 3.42E-59 | Antimicrobials |
| CXCL1 | Growth-regulated alpha protein | 3.05685452 | 6.44E-26 | Antimicrobials |
| IL12B | Interleukin-12 subunit beta | 3.05282494 | 2.70E-30 | Antimicrobials |
| SPAG11A | Sperm associated antigen 11A | 2.97341147 | 9.63E-11 | Antimicrobials |
| DMBT1 | Deleted in malignant brain tumors 1 protein | 2.96350803 | 1.16E-13 | Antimicrobials |
| LYZ | Lysozyme C | 2.93638732 | 4.23E-32 | Antimicrobials |
| CHIT1 | Chitotriosidase-1 | 2.93132653 | 1.68E-19 | Antimicrobials |
| CCL5 | C-C motif chemokine 5 | 2.91630226 | 1.47E-36 | Antimicrobials |
| CD86 | T-lymphocyte activation antigen CD86 | 2.88042714 | 1.23E-44 | Antimicrobials |
| UNC93B1 | Protein unc-93 homolog B1 | 2.82609154 | 1.37E-76 | Antimicrobials |
| S100A7A | Protein S100-A7A | 2.81849268 | 3.64E-11 | Antimicrobials |
| CXCL16 | C-X-C motif chemokine 16 | 2.81504907 | 9.16E-68 | Antimicrobials |
| IL27 | Interleukin-27 subunit alpha | 2.79610437 | 1.27E-18 | Antimicrobials |
| TFR2 | Transferrin receptor protein 2 | 2.67721683 | 8.72E-39 | Antimicrobials |
| DEFB123 | Beta-defensin 123 | 2.58923202 | 1.12E-09 | Antimicrobials |
| CCL2 | C-C motif chemokine 2 | 2.56057703 | 4.40E-37 | Antimicrobials |
| IKBKE | Inhibitor of nuclear factor kappa-B kinase subunit epsilon | 2.55741222 | 1.33E-65 | Antimicrobials |
| OAS1 | 2'-5'-oligoadenylate synthase 1 | 2.54054131 | 1.27E-37 | Antimicrobials |
| CCR4 | C-C chemokine receptor type 4 | 2.48767192 | 7.18E-18 | Antimicrobials |
| CCL1 | C-C motif chemokine 1 | 2.46454534 | 1.79E-12 | Antimicrobials |
| CCL4L1 | C-C motif chemokine 4-like | 2.44145762 | 8.23E-17 | Antimicrobials |
| IRF5 | Interferon regulatory factor 5 | 2.43893146 | 9.25E-53 | Antimicrobials |
| CCL4 | C-C motif chemokine 4 | 2.41011747 | 6.49E-34 | Antimicrobials |
| LTF | Lactotransferrin | 2.38477228 | 1.73E-17 | Antimicrobials |
| MSR1 | Macrophage scavenger receptor types I and II | 2.37183957 | 2.07E-35 | Antimicrobials |
| CCR1 | C-C chemokine receptor type 1 | 2.35447539 | 2.01E-32 | Antimicrobials |
| TLR8 | Toll-like receptor 8 | 2.34036613 | 3.00E-19 | Antimicrobials |
| PPBP | Platelet basic protein | 2.33797155 | 7.33E-09 | Antimicrobials |
| MIF | Macrophage migration inhibitory factor | 2.30898382 | 1.13E-54 | Antimicrobials |
| TMPRSS6 | Transmembrane protease serine 6 | 2.24589696 | 1.14E-15 | Antimicrobials |
| SFTPD | Pulmonary surfactant-associated protein D | 2.19875547 | 2.49E-23 | Antimicrobials |
| FABP5 | Fatty acid-binding protein, epidermal | 2.14733519 | 2.81E-43 | Antimicrobials |
| PTGDS | Prostaglandin-H2 D-isomerase | 2.14086406 | 9.01E-15 | Antimicrobials |
| CYBB | Cytochrome b-245 heavy chain | 2.11569737 | 4.71E-23 | Antimicrobials |
| MX2 | Interferon-induced GTP-binding protein Mx2 | 2.08129085 | 1.97E-28 | Antimicrobials |
| CCR5 | C-C chemokine receptor type 5 | 2.0208644 | 4.11E-20 | Antimicrobials |
| ADIPOQ | Adiponectin | -2.0122019 | 0.0000159 | Antimicrobials |
| DUOX2 | Dual oxidase 2 | -2.0458803 | 1.31E-16 | Antimicrobials |
| ORM1 | Alpha-1-acid glycoprotein 1 | -2.1106134 | 0.00000758 | Antimicrobials |
| LTB4R | Leukotriene B4 receptor 1 | -2.1367456 | 7.22E-46 | Antimicrobials |
| TRIM22 | E3 ubiquitin-protein ligase TRIM22 | -2.1381307 | 2.06E-34 | Antimicrobials |
| VCAM1 | Vascular cell adhesion protein 1 | -2.1537769 | 7.53E-23 | Antimicrobials |
| HMOX1 | Heme oxygenase 1 | -2.1960741 | 4.34E-37 | Antimicrobials |
| APOH | Beta-2-glycoprotein 1 | -2.209584 | 1.67E-07 | Antimicrobials |
| ELN | Elastin | -2.2322138 | 9.82E-28 | Antimicrobials |
| WFIKKN1 | WAP, Kazal, immunoglobulin, Kunitz and NTR domain-containing protein 1 | -2.2929556 | 1.19E-31 | Antimicrobials |
| TPM2 | Tropomyosin beta chain | -2.3408297 | 5.00E-47 | Antimicrobials |
| ANXA6 | Annexin A6 | -2.3516882 | 1.18E-60 | Antimicrobials |
| LEAP2 | Liver-expressed antimicrobial peptide 2 | -2.3603766 | 4.48E-45 | Antimicrobials |
| RNASEL | 2-5A-dependent ribonuclease | -2.3744395 | 8.49E-66 | Antimicrobials |
| FAM19A4 | Protein FAM19A4 | -2.3836007 | 1.79E-11 | Antimicrobials |
| BACH2 | Transcription regulator protein BACH2 | -2.4654603 | 4.72E-34 | Antimicrobials |
| CCL23 | C-C motif chemokine ligand 23 | -2.5064842 | 2.00E-11 | Antimicrobials |
| NFKBIZ | NF-kappa-B inhibitor zeta | -2.5293267 | 1.55E-47 | Antimicrobials |
| JAK2 | Tyrosine-protein kinase JAK2 | -2.5915709 | 2.44E-77 | Antimicrobials |
| DEFA6 | Defensin-6 | -2.6078358 | 2.87E-22 | Antimicrobials |
| DDX17 | Probable ATP-dependent RNA helicase DDX17 | -2.6340349 | 1.27E-80 | Antimicrobials |
| NOS2 | Nitric oxide synthase, inducible | -2.6647695 | 1.11E-33 | Antimicrobials |
| SLC22A17 | Solute carrier family 22 member 17 | -2.6713252 | 1.02E-41 | Antimicrobials |
| PTGDR | Prostaglandin D2 receptor | -2.6820554 | 2.58E-30 | Antimicrobials |
| TNFAIP3 | Tumor necrosis factor alpha-induced protein 3 | -2.7639825 | 2.12E-57 | Antimicrobials |
| LMBR1L | Protein LMBR1L | -2.7642462 | 5.31E-91 | Antimicrobials |
| NOD1 | Nucleotide-binding oligomerization domain-containing protein 1 | -2.8033046 | 1.67E-92 | Antimicrobials |
| VTN | Vitronectin | -2.8062605 | 1.32E-42 | Antimicrobials |
| VIM | Vimentin | -2.8321235 | 2.00E-58 | Antimicrobials |
| CCR10 | C-C chemokine receptor type 10 | -2.8543679 | 1.08E-61 | Antimicrobials |
| RBP1 | Retinol-binding protein 1 | -2.8972899 | 9.15E-54 | Antimicrobials |
| IL13 | Interleukin-13 | -2.994224 | 1.05E-19 | Antimicrobials |
| FAM19A5 | Protein FAM19A5 | -3.0055033 | 1.20E-34 | Antimicrobials |
| MASP2 | Mannan-binding lectin serine protease 2 | -3.084328 | 5.07E-47 | Antimicrobials |
| KLKB1 | Plasma kallikrein | -3.1075609 | 2.84E-31 | Antimicrobials |
| CCL27 | C-C motif chemokine 27 | -3.1126481 | 1.00E-15 | Antimicrobials |
| LRP1 | Prolow-density lipoprotein receptor-related protein 1 | -3.1259265 | 1.19E-78 | Antimicrobials |
| DEFB124 | Beta-defensin 124 | -3.1564111 | 5.10E-11 | Antimicrobials |
| SFTPA2 | Pulmonary surfactant-associated protein A2 | -3.1851342 | 5.26E-26 | Antimicrobials |
| DEFA5 | Defensin-5 | -3.2847675 | 1.17E-22 | Antimicrobials |
| ALB | Serum albumin | -3.4545841 | 4.35E-33 | Antimicrobials |
| TMSB15B | Thymosin Beta 15B | -3.5287146 | 6.81E-54 | Antimicrobials |
| APOD | Apolipoprotein D | -3.5603861 | 4.97E-70 | Antimicrobials |
| PPARG | Peroxisome proliferator-activated receptor gamma | -3.5616458 | 4.64E-80 | Antimicrobials |
| PDGFRB | Platelet-derived growth factor receptor beta | -3.585465 | 1.87E-78 | Antimicrobials |
| ELANE | Neutrophil elastase | -3.6823321 | 1.71E-23 | Antimicrobials |
| CCL14 | C-C motif chemokine ligand 14 | -3.6838545 | 2.69E-38 | Antimicrobials |
| MASP1 | Mannan binding lectin serine peptidase 1 | -3.7994185 | 1.70E-44 | Antimicrobials |
| LCN10 | Epididymal-specific lipocalin-10 | -3.8021706 | 1.42E-36 | Antimicrobials |
| ROBO3 | Roundabout homolog 3 | -4.0192953 | 6.74E-88 | Antimicrobials |
| SERPINA3 | Serpin Family A Member 3 | -4.0295506 | 3.47E-37 | Antimicrobials |
| CCL21 | C-C motif chemokine 21 | -4.0834378 | 1.53E-18 | Antimicrobials |
| DES | Desmin | -4.3307311 | 4.08E-56 | Antimicrobials |
| FABP4 | Fatty acid-binding protein, adipocyte | -4.3433443 | 2.94E-19 | Antimicrobials |
| STAB2 | Stabilin-2 | -4.4563406 | 1.33E-35 | Antimicrobials |
| SERPIND1 | Heparin cofactor 2 | -4.5134559 | 1.27E-62 | Antimicrobials |
| DEFA3 | Neutrophil defensin 3 | -4.5178617 | 8.76E-27 | Antimicrobials |
| PI15 | Peptidase inhibitor 15 | -4.6837322 | 7.26E-52 | Antimicrobials |
| PMP2 | Myelin P2 protein | -4.6982867 | 2.38E-59 | Antimicrobials |
| FCN2 | Ficolin-2 | -4.8490855 | 3.89E-34 | Antimicrobials |
| ACTA1 | Actin, alpha skeletal muscle | -4.8950775 | 6.30E-54 | Antimicrobials |
| SFTPA1 | Pulmonary surfactant-associated protein A1 | -4.918212 | 5.99E-56 | Antimicrobials |
| FGF2 | Fibroblast growth factor 2 | -4.9669906 | 3.17E-120 | Antimicrobials |
| HTR1A | 5-hydroxytryptamine receptor 1A | -4.9836426 | 1.45E-60 | Antimicrobials |
| PCSK2 | Neuroendocrine convertase 2 | -5.1627822 | 2.47E-32 | Antimicrobials |
| DEFA1 | Neutrophil defensin 1 | -5.270574 | 6.02E-32 | Antimicrobials |
| CCL16 | C-C motif chemokine 16 | -5.3468791 | 3.48E-64 | Antimicrobials |
| PDGFRA | Platelet-derived growth factor receptor alpha | -5.82848 | 2.68E-108 | Antimicrobials |
| CCL26 | C-C motif chemokine 26 | -5.8808452 | 2.58E-71 | Antimicrobials |
| CRP | C-reactive protein | -6.2962747 | 1.84E-70 | Antimicrobials |
| DCD | Dermcidin | -6.5291075 | 2.32E-92 | Antimicrobials |
| FGA | Fibrinogen alpha chain | -6.716732 | 2.85E-78 | Antimicrobials |
| BPI | Bactericidal permeability-increasing protein | -6.7446953 | 1.87E-62 | Antimicrobials |
| PENK | Proenkephalin-A | -7.4554317 | 6.87E-59 | Antimicrobials |
| PLA2G2A | Phospholipase A2, membrane associated | -7.4619069 | 4.09E-65 | Antimicrobials |
| PGC | Gastricsin | -7.5393646 | 9.79E-78 | Antimicrobials |
| LCN6 | Epididymal-specific lipocalin-6 | -8.0128627 | 7.73E-79 | Antimicrobials |
| SYK | Tyrosine-protein kinase SYK | 4.52042183 | 1.90E-116 | BCRSignalingPathway |
| CR2 | Complement receptor type 2 | 3.94427764 | 5.71E-36 | BCRSignalingPathway |
| VAV3 | Guanine nucleotide exchange factor VAV3 | 3.37700743 | 2.18E-32 | BCRSignalingPathway |
| PTPN6 | Tyrosine-protein phosphatase non-receptor type 6 | 2.81918212 | 3.43E-96 | BCRSignalingPathway |
| BLNK | B-cell linker protein | 2.52977702 | 1.23E-47 | BCRSignalingPathway |
| FCGR2B | Low affinity immunoglobulin gamma Fc region receptor II-b | 2.48981732 | 7.44E-29 | BCRSignalingPathway |
| VAV1 | Proto-oncogene vav | 2.42274829 | 2.70E-35 | BCRSignalingPathway |
| RAC3 | Ras-related C3 botulinum toxin substrate 3 | 2.18120758 | 2.02E-42 | BCRSignalingPathway |
| LYN | Tyrosine-protein kinase Lyn | 2.17233184 | 1.17E-42 | BCRSignalingPathway |
| NFKBIE | NF-kappa-B inhibitor epsilon | 2.13798368 | 2.00E-59 | BCRSignalingPathway |
| CARD11 | Caspase recruitment domain-containing protein 11 | 2.06299854 | 2.46E-22 | BCRSignalingPathway |
| CHP2 | Calcineurin B homologous protein 2 | 2.05070532 | 1.33E-07 | BCRSignalingPathway |
| AKT3 | RAC-gamma serine/threonine-protein kinase | -3.9025358 | 1.27E-75 | BCRSignalingPathway |
| NFATC4 | Nuclear factor of activated T-cells, cytoplasmic 4 | -4.1747017 | 9.06E-115 | BCRSignalingPathway |
| CD22 | B-cell receptor CD22 | -5.2150434 | 6.37E-78 | BCRSignalingPathway |
| CXCR3 | C-X-C chemokine receptor type 3 | 5.16955403 | 1.46E-66 | Chemokine_Receptors |
| CX3CR1 | CX3C chemokine receptor 1 | 4.08010249 | 1.56E-61 | Chemokine_Receptors |
| PTAFR | Platelet-activating factor receptor | 3.37357538 | 6.40E-63 | Chemokine_Receptors |
| CYSLTR1 | Cysteinyl leukotriene receptor 1 | 2.42698108 | 1.21E-28 | Chemokine_Receptors |
| ROBO1 | Roundabout homolog 1 | -2.0570723 | 9.47E-32 | Chemokine_Receptors |
| ROBO2 | Roundabout homolog 2 | -2.5700264 | 1.35E-29 | Chemokine_Receptors |
| LTB4R2 | Leukotriene B4 receptor 2 | -2.612556 | 5.54E-52 | Chemokine_Receptors |
| PLXNA4 | Plexin-A4 | -2.6274627 | 1.72E-21 | Chemokine_Receptors |
| CYSLTR2 | Cysteinyl leukotriene receptor 2 | -2.638899 | 5.67E-30 | Chemokine_Receptors |
| GPR17 | Uracil nucleotide/cysteinyl leukotriene receptor | -2.7401644 | 4.25E-22 | Chemokine_Receptors |
| PLXNA2 | Plexin-A2 | -2.8781809 | 3.73E-63 | Chemokine_Receptors |
| EDNRA | Endothelin-1 receptor | -3.3617454 | 9.87E-61 | Chemokine_Receptors |
| CXCL17 | C-X-C motif chemokine 17 | 9.18820006 | 4.22E-94 | Chemokines |
| EDN2 | Endothelin-2 | 6.39796793 | 7.10E-86 | Chemokines |
| SEMA3E | Semaphorin-3E | 4.10286066 | 3.10E-27 | Chemokines |
| SEMA3F | Semaphorin-3F | 3.6010182 | 1.28E-79 | Chemokines |
| SEMA3A | Semaphorin-3A | 2.72937016 | 5.34E-30 | Chemokines |
| TYMP | Thymidine phosphorylase | 2.36201416 | 6.83E-35 | Chemokines |
| TNC | Tenascin | 2.27907725 | 1.89E-20 | Chemokines |
| SAA1 | Serum amyloid A-1 protein | 2.27010545 | 1.69E-11 | Chemokines |
| SAA2 | Serum amyloid A-2 protein | 2.00078326 | 3.37E-07 | Chemokines |
| SEMA6C | Semaphorin-6C | -2.0027193 | 1.29E-34 | Chemokines |
| SEMA6D | Semaphorin-6D | -2.2188632 | 4.34E-38 | Chemokines |
| SEMA4G | Semaphorin-4G | -2.2253723 | 1.72E-45 | Chemokines |
| C5 | Complement C5 | -3.0975145 | 8.33E-71 | Chemokines |
| SEMA6A | Semaphorin-6A | -3.1684126 | 1.91E-54 | Chemokines |
| FGF10 | Fibroblast growth factor 10 | -8.8943018 | 2.53E-93 | Chemokines |
| HTR3A | 5-hydroxytryptamine receptor 3A | 9.90601077 | 4.49E-110 | Cytokine_Receptors |
| PTH2R | Parathyroid hormone 2 receptor | 7.46531917 | 5.95E-63 | Cytokine_Receptors |
| TNFRSF9 | Tumor necrosis factor receptor superfamily member 9 | 6.72078367 | 1.51E-117 | Cytokine_Receptors |
| PTGER1 | Prostaglandin E2 receptor EP1 subtype | 6.13289149 | 1.49E-79 | Cytokine_Receptors |
| AVPR1B | Vasopressin V1b receptor | 5.42097391 | 1.04E-63 | Cytokine_Receptors |
| IL22RA2 | Interleukin-22 receptor subunit alpha-2 | 4.69569072 | 9.90E-44 | Cytokine_Receptors |
| RORC | Nuclear receptor ROR-gamma | 4.57576083 | 8.76E-85 | Cytokine_Receptors |
| HNF4G | Hepatocyte nuclear factor 4-gamma | 4.45415522 | 7.56E-30 | Cytokine_Receptors |
| GLP1R | Glucagon-like peptide 1 receptor | 4.28027626 | 1.70E-35 | Cytokine_Receptors |
| GALR2 | Galanin receptor type 2 | 4.04050517 | 7.26E-39 | Cytokine_Receptors |
| TNFRSF12A | Tumor necrosis factor receptor superfamily member 12A | 3.99824633 | 2.36E-104 | Cytokine_Receptors |
| SDC1 | Syndecan-1 | 3.81919912 | 2.44E-64 | Cytokine_Receptors |
| IL2RA | Interleukin-2 receptor subunit alpha | 3.66526772 | 1.19E-45 | Cytokine_Receptors |
| MC4R | Melanocortin receptor 4 | 3.63945095 | 6.59E-22 | Cytokine_Receptors |
| HTR3E | 5-hydroxytryptamine receptor 3E | 3.60022536 | 2.43E-33 | Cytokine_Receptors |
| TNFRSF18 | Tumor necrosis factor receptor superfamily member 18 | 3.54309523 | 6.28E-37 | Cytokine_Receptors |
| HTR3B | 5-hydroxytryptamine receptor 3B | 3.4822484 | 4.40E-22 | Cytokine_Receptors |
| LGR6 | Leucine-rich repeat-containing G-protein coupled receptor 6 | 3.42033572 | 3.69E-37 | Cytokine_Receptors |
| TUBB3 | Tubulin Beta 3 Class III | 3.26623606 | 1.06E-42 | Cytokine_Receptors |
| APLNR | Apelin receptor | 3.26524565 | 9.43E-47 | Cytokine_Receptors |
| IL21R | Interleukin-21 receptor | 3.19468653 | 9.18E-30 | Cytokine_Receptors |
| IL2RB | Interleukin-2 receptor subunit beta | 3.17794201 | 5.56E-41 | Cytokine_Receptors |
| SORT1 | Sortilin | 3.14988557 | 6.52E-77 | Cytokine_Receptors |
| NR2F6 | Nuclear receptor subfamily 2 group F member 6 | 3.08477666 | 1.82E-84 | Cytokine_Receptors |
| OPRK1 | Kappa-type opioid receptor | 3.07836039 | 1.69E-17 | Cytokine_Receptors |
| NR2E3 | Photoreceptor-specific nuclear receptor | 2.88970336 | 3.27E-27 | Cytokine_Receptors |
| IL2RG | Cytokine receptor common subunit gamma | 2.82881125 | 4.58E-41 | Cytokine_Receptors |
| CALCR | Calcitonin receptor | 2.74615627 | 1.80E-24 | Cytokine_Receptors |
| MET | Hepatocyte growth factor receptor | 2.67885246 | 1.26E-39 | Cytokine_Receptors |
| IL5RA | Interleukin-5 receptor subunit alpha | 2.54462933 | 5.92E-14 | Cytokine_Receptors |
| MLNR | Motilin receptor | 2.51924343 | 2.68E-13 | Cytokine_Receptors |
| ESRRG | Estrogen-related receptor gamma | 2.51213004 | 2.20E-29 | Cytokine_Receptors |
| IL1RAP | Interleukin 1 receptor accessory protein | 2.37671651 | 1.92E-50 | Cytokine_Receptors |
| LGR5 | Leucine-rich repeat-containing G-protein coupled receptor 5 | 2.35561852 | 8.06E-14 | Cytokine_Receptors |
| ESRRB | Steroid hormone receptor ERR2 | 2.31903529 | 1.70E-14 | Cytokine_Receptors |
| TNFRSF13C | Tumor necrosis factor receptor superfamily member 13C | 2.31533442 | 7.93E-36 | Cytokine_Receptors |
| GALR3 | Galanin receptor type 3 | 2.31262892 | 4.35E-12 | Cytokine_Receptors |
| IL27RA | Interleukin-27 receptor subunit alpha | 2.26164869 | 9.44E-36 | Cytokine_Receptors |
| FGFR3 | Fibroblast growth factor receptor 3 | 2.10693693 | 1.38E-16 | Cytokine_Receptors |
| SDC4 | Syndecan-4 | 2.01096339 | 3.75E-42 | Cytokine_Receptors |
| SDC3 | Syndecan-3 | 2.01006346 | 8.46E-36 | Cytokine_Receptors |
| MTNR1A | Melatonin receptor type 1A | 2.00758751 | 1.56E-09 | Cytokine_Receptors |
| RORB | Nuclear receptor ROR-beta | -2.0038892 | 2.40E-13 | Cytokine_Receptors |
| THRA | Thyroid hormone receptor alpha | -2.1423317 | 2.38E-55 | Cytokine_Receptors |
| LIFR | Leukemia inhibitory factor receptor | -2.3101733 | 7.29E-58 | Cytokine_Receptors |
| MC1R | Melanocyte-stimulating hormone receptor | -2.3284124 | 8.28E-51 | Cytokine_Receptors |
| TNFRSF14 | Tumor necrosis factor receptor superfamily member 14 | -2.3494268 | 9.75E-52 | Cytokine_Receptors |
| GNRHR | Gonadotropin-releasing hormone receptor | -2.4694676 | 4.82E-19 | Cytokine_Receptors |
| IL1RL1 | Interleukin-1 receptor-like 1 | -2.4824387 | 3.06E-20 | Cytokine_Receptors |
| INSR | Insulin receptor | -2.5080581 | 1.08E-66 | Cytokine_Receptors |
| RORA | Nuclear receptor ROR-alpha | -2.5088977 | 1.23E-61 | Cytokine_Receptors |
| TNFRSF10D | Tumor necrosis factor receptor superfamily member 10D | -2.5483188 | 2.90E-47 | Cytokine_Receptors |
| ANGPTL2 | Angiopoietin-related protein 2 | -2.5485794 | 1.15E-49 | Cytokine_Receptors |
| HNF4A | Hepatocyte nuclear factor 4-alpha | -2.6186227 | 3.39E-15 | Cytokine_Receptors |
| IL11RA | Interleukin-11 receptor subunit alpha | -2.6408278 | 1.76E-45 | Cytokine_Receptors |
| CRHR2 | Corticotropin-releasing factor receptor 2 | -2.6764159 | 2.40E-20 | Cytokine_Receptors |
| ENG | Endoglin | -2.8377806 | 2.48E-81 | Cytokine_Receptors |
| NR1D1 | Nuclear receptor subfamily 1 group D member 1 | -2.8654554 | 5.71E-75 | Cytokine_Receptors |
| NR3C2 | Mineralocorticoid receptor | -2.913871 | 2.45E-61 | Cytokine_Receptors |
| ANGPT1 | Angiopoietin-1 | -2.9778167 | 4.77E-52 | Cytokine_Receptors |
| GHR | Growth hormone receptor | -3.027742 | 2.59E-48 | Cytokine_Receptors |
| SDC2 | Syndecan-2 | -3.0317489 | 3.78E-51 | Cytokine_Receptors |
| LEPR | Leptin receptor | -3.1513663 | 1.81E-76 | Cytokine_Receptors |
| FGFR4 | Fibroblast growth factor receptor 4 | -3.1524512 | 5.11E-63 | Cytokine_Receptors |
| PRLR | Prolactin receptor | -3.2481203 | 1.32E-44 | Cytokine_Receptors |
| NR2F2 | COUP transcription factor 2 | -3.3109531 | 4.12E-84 | Cytokine_Receptors |
| NR4A2 | Nuclear receptor subfamily 4 group A member 2 | -3.5819544 | 3.17E-63 | Cytokine_Receptors |
| ACVR1C | Activin receptor type-1C | -3.7124081 | 3.09E-57 | Cytokine_Receptors |
| NR2F1 | COUP transcription factor 1 | -3.8268425 | 2.34E-72 | Cytokine_Receptors |
| AGTR1 | Type-1 angiotensin II receptor | -3.9466368 | 1.36E-48 | Cytokine_Receptors |
| SSTR1 | Somatostatin receptor type 1 | -3.9905657 | 3.00E-49 | Cytokine_Receptors |
| TGFBR3 | Transforming growth factor beta receptor type 3 | -4.0317409 | 2.46E-111 | Cytokine_Receptors |
| ANGPTL3 | Angiopoietin-related protein 3 | -4.112014 | 9.31E-41 | Cytokine_Receptors |
| MCHR1 | Melanin-concentrating hormone receptor 1 | -4.1874297 | 3.20E-48 | Cytokine_Receptors |
| ESR2 | Estrogen receptor beta | -4.4986252 | 2.87E-95 | Cytokine_Receptors |
| GLP2R | Glucagon-like peptide 2 receptor | -4.6082259 | 3.32E-48 | Cytokine_Receptors |
| SCTR | Secretin receptor | -4.8234514 | 3.79E-32 | Cytokine_Receptors |
| PGR | Progesterone receptor | -4.9159098 | 5.04E-58 | Cytokine_Receptors |
| NR4A3 | Nuclear receptor subfamily 4 group A member 3 | -4.9749334 | 2.06E-75 | Cytokine_Receptors |
| PTH1R | Parathyroid hormone/parathyroid hormone-related peptide receptor | -5.0793759 | 6.72E-94 | Cytokine_Receptors |
| VIPR2 | Vasoactive intestinal polypeptide receptor 2 | -5.1341458 | 1.03E-74 | Cytokine_Receptors |
| NR4A1 | Nuclear receptor subfamily 4 group A member 1 | -5.3825448 | 3.98E-102 | Cytokine_Receptors |
| NR0B1 | Nuclear receptor subfamily 0 group B member 1 | -5.4407632 | 1.36E-39 | Cytokine_Receptors |
| ANGPTL1 | Angiopoietin-related protein 1 | -5.7794063 | 2.48E-86 | Cytokine_Receptors |
| FSHR | Follicle-stimulating hormone receptor | -5.8154199 | 9.76E-57 | Cytokine_Receptors |
| PTGFR | Prostaglandin F2-alpha receptor | -5.8532386 | 4.34E-62 | Cytokine_Receptors |
| LHCGR | Lutropin-choriogonadotropic hormone receptor | -6.0123274 | 2.60E-66 | Cytokine_Receptors |
| ANGPT4 | Angiopoietin-4 | -6.2913911 | 3.74E-97 | Cytokine_Receptors |
| NR5A1 | Steroidogenic factor 1 | -6.9926936 | 1.35E-44 | Cytokine_Receptors |
| AMHR2 | Anti-Muellerian hormone type-2 receptor | -7.8063421 | 5.90E-58 | Cytokine_Receptors |
| NR1H4 | Bile acid receptor | -9.3138863 | 5.70E-89 | Cytokine_Receptors |
| PNOC | Prepronociceptin | 7.344733 | 1.02E-76 | Cytokines |
| BMP7 | Bone morphogenetic protein 7 | 7.01973001 | 1.92E-83 | Cytokines |
| GRP | Gastrin-releasing peptide | 6.41219635 | 8.02E-42 | Cytokines |
| CGB2 | Chorionic gonadotropin beta subunit 2 | 6.1031874 | 3.11E-29 | Cytokines |
| RLN1 | Prorelaxin H1 | 6.08087984 | 2.26E-79 | Cytokines |
| FGF18 | Fibroblast growth factor 18 | 6.01772115 | 3.74E-110 | Cytokines |
| CD70 | CD70 antigen | 5.97058365 | 2.95E-63 | Cytokines |
| FAM3B | Protein FAM3B | 5.9626692 | 3.14E-87 | Cytokines |
| ADM2 | ADM2 | 5.8961805 | 9.65E-116 | Cytokines |
| SPP1 | Osteopontin | 5.84433561 | 1.88E-105 | Cytokines |
| NRTN | Neurturin | 5.65495431 | 2.12E-84 | Cytokines |
| RLN2 | Prorelaxin H2 | 5.64039572 | 1.45E-69 | Cytokines |
| SST | Somatostatin | 5.47038618 | 1.53E-34 | Cytokines |
| GAL | Galanin peptides | 5.42947066 | 1.38E-53 | Cytokines |
| FGF19 | Fibroblast growth factor 19 | 5.38216118 | 7.28E-32 | Cytokines |
| NRG3 | Pro-neuregulin-3, membrane-bound isoform | 5.31686893 | 6.90E-36 | Cytokines |
| IL19 | Interleukin-19 | 4.8774931 | 3.18E-44 | Cytokines |
| TNFSF15 | Tumor necrosis factor ligand superfamily member 15 | 4.75806633 | 1.26E-102 | Cytokines |
| GAST | Gastrin | 4.73764864 | 7.55E-24 | Cytokines |
| FGF3 | Fibroblast growth factor 3 | 4.4338309 | 1.51E-18 | Cytokines |
| CSPG5 | Chondroitin sulfate proteoglycan 5 | 4.31950868 | 6.78E-94 | Cytokines |
| TNFSF18 | Tumor necrosis factor ligand superfamily member 18 | 4.13907049 | 2.12E-51 | Cytokines |
| TGFA | Protransforming growth factor alpha | 4.13883131 | 1.48E-68 | Cytokines |
| CMTM8 | CKLF like MARVEL transmembrane domain containing 8 | 4.10303118 | 5.81E-135 | Cytokines |
| GALP | Galanin-like peptide | 4.04224891 | 9.62E-21 | Cytokines |
| CSF2 | Granulocyte-macrophage colony-stimulating factor | 3.96488612 | 1.78E-27 | Cytokines |
| IL1RN | Interleukin-1 receptor antagonist protein | 3.96403065 | 3.32E-82 | Cytokines |
| LTB | Lymphotoxin-beta | 3.95148813 | 2.28E-54 | Cytokines |
| ESM1 | Endothelial cell-specific molecule 1 | 3.89606769 | 1.28E-67 | Cytokines |
| CMTM7 | CKLF like MARVEL transmembrane domain containing 7 | 3.87327229 | 1.47E-100 | Cytokines |
| NTF4 | Neurotrophin-4 | 3.84964232 | 5.72E-41 | Cytokines |
| STC2 | Stanniocalcin-2 | 3.78113672 | 2.46E-70 | Cytokines |
| IL17C | Interleukin-17C | 3.64861478 | 2.60E-22 | Cytokines |
| TNFRSF11B | Tumor necrosis factor receptor superfamily member 11B | 3.43738202 | 1.88E-43 | Cytokines |
| GDF5 | Growth/differentiation factor 5 | 3.40606817 | 9.34E-31 | Cytokines |
| UCN3 | Urocortin-3 | 3.30836136 | 4.82E-18 | Cytokines |
| UTS2 | Urotensin 2 | 3.25935449 | 5.78E-14 | Cytokines |
| PYY | Peptide YY | 3.25288668 | 1.34E-16 | Cytokines |
| FGF21 | Fibroblast growth factor 21 | 3.12600842 | 9.83E-16 | Cytokines |
| GIP | Gastric inhibitory polypeptide | 3.10918352 | 4.23E-17 | Cytokines |
| FAM3D | Protein FAM3D | 3.0118803 | 1.44E-15 | Cytokines |
| CSH2 | Chorionic somatomammotropin hormone 2 | 2.9660753 | 2.50E-16 | Cytokines |
| GDF3 | Growth/differentiation factor 3 | 2.87769984 | 7.93E-18 | Cytokines |
| CCK | Cholecystokinin | 2.8035783 | 3.02E-11 | Cytokines |
| INHBB | Inhibin beta B chain | 2.80103374 | 5.55E-35 | Cytokines |
| IL32 | Interleukin-32 | 2.73969793 | 1.59E-44 | Cytokines |
| CGB5 | Chorionic gonadotropin beta subunit 5 | 2.72226594 | 2.55E-10 | Cytokines |
| SCGB3A1 | Secretoglobin family 3A member 1 | 2.72039896 | 1.74E-17 | Cytokines |
| IL11 | Interleukin-11 | 2.5837261 | 1.43E-27 | Cytokines |
| FGF16 | Fibroblast growth factor 16 | 2.56992385 | 3.15E-15 | Cytokines |
| NTS | Neurotensin/neuromedin N | 2.56028915 | 4.50E-12 | Cytokines |
| PMCH | Pro-MCH | 2.55815018 | 4.86E-13 | Cytokines |
| TNFSF8 | Tumor necrosis factor ligand superfamily member 8 | 2.44544981 | 1.70E-22 | Cytokines |
| CGB | Chorionic gonadotropin, beta polypeptide | 2.4098829 | 2.39E-09 | Cytokines |
| BTC | Probetacellulin | 2.39998341 | 4.04E-32 | Cytokines |
| CMTM6 | CKLF like MARVEL transmembrane domain containing 6 | 2.31433858 | 6.48E-54 | Cytokines |
| TNFSF13B | Tumor necrosis factor ligand superfamily member 13B | 2.21128257 | 4.10E-34 | Cytokines |
| FGF8 | Fibroblast growth factor 8 | 2.1992981 | 8.98E-09 | Cytokines |
| SCT | Secretin | 2.19524385 | 1.34E-08 | Cytokines |
| CGB1 | Chorionic gonadotropin beta subunit 1 | 2.16829905 | 0.00000169 | Cytokines |
| VGF | Neurosecretory protein VGF | 2.09649608 | 6.98E-18 | Cytokines |
| NOV | Protein NOV homolog | 2.04515389 | 2.52E-26 | Cytokines |
| LIF | Leukemia inhibitory factor | 2.04435897 | 9.37E-23 | Cytokines |
| ARTN | Artemin | 2.030058 | 3.04E-35 | Cytokines |
| INHBC | Inhibin beta C chain | 2.02625084 | 1.18E-16 | Cytokines |
| FGF12 | Fibroblast growth factor 12 | 2.01781716 | 5.14E-23 | Cytokines |
| AGT | Angiotensinogen | -2.1350975 | 3.64E-16 | Cytokines |
| TG | Thyroglobulin | -2.1539945 | 1.03E-25 | Cytokines |
| UCN | Urocortin | -2.1659894 | 6.17E-35 | Cytokines |
| NODAL | Nodal homolog | -2.2317308 | 4.87E-16 | Cytokines |
| LEFTY1 | Left-right determination factor 1 | -2.2746299 | 2.55E-14 | Cytokines |
| BMP10 | Bone morphogenetic protein 10 | -2.278025 | 4.98E-32 | Cytokines |
| DKK1 | Dickkopf-related protein 1 | -2.2816413 | 2.68E-10 | Cytokines |
| BMP6 | Bone morphogenetic protein 6 | -2.3079212 | 1.93E-26 | Cytokines |
| TSLP | Thymic stromal lymphopoietin | -2.3340385 | 1.61E-19 | Cytokines |
| TSHB | Thyrotropin subunit beta | -2.4664784 | 7.54E-20 | Cytokines |
| BMP2 | Bone morphogenetic protein 2 | -2.4699777 | 1.19E-33 | Cytokines |
| GCG | Glucagon | -2.4981106 | 3.43E-25 | Cytokines |
| POMC | Pro-opiomelanocortin | -2.6303756 | 8.04E-39 | Cytokines |
| MIA | Melanoma-derived growth regulatory protein | -2.6649024 | 7.98E-22 | Cytokines |
| NTF3 | Neurotrophin-3 | -2.6718401 | 2.34E-19 | Cytokines |
| EPO | Erythropoietin | -2.7349345 | 3.44E-21 | Cytokines |
| IL25 | Interleukin-25 | -2.7429645 | 2.39E-19 | Cytokines |
| IFNK | Interferon kappa | -2.77878 | 2.48E-21 | Cytokines |
| NDP | Norrin | -2.8885279 | 2.61E-25 | Cytokines |
| FGF20 | Fibroblast growth factor 20 | -2.9153502 | 3.10E-15 | Cytokines |
| FGF17 | Fibroblast growth factor 17 | -2.9192045 | 4.80E-16 | Cytokines |
| GDF7 | Growth/differentiation factor 7 | -2.9564099 | 3.28E-37 | Cytokines |
| CSF3 | Granulocyte colony-stimulating factor | -3.0573549 | 3.47E-14 | Cytokines |
| GDNF | Glial cell line-derived neurotrophic factor | -3.0592306 | 5.55E-29 | Cytokines |
| LTBP4 | Latent-transforming growth factor beta-binding protein 4 | -3.1019619 | 9.30E-57 | Cytokines |
| REG1A | Lithostathine-1-alpha | -3.1139324 | 1.16E-09 | Cytokines |
| NPPB | Natriuretic peptides B | -3.3297892 | 7.46E-15 | Cytokines |
| ANGPTL7 | Angiopoietin-related protein 7 | -3.3821657 | 2.59E-26 | Cytokines |
| IL5 | Interleukin-5 | -3.4148258 | 3.02E-21 | Cytokines |
| GNRH2 | Progonadoliberin-2 | -3.5930442 | 4.97E-31 | Cytokines |
| NRG2 | Pro-neuregulin-2, membrane-bound isoform | -3.5983954 | 1.65E-63 | Cytokines |
| BMP3 | Bone morphogenetic protein 3 | -3.6218133 | 3.08E-25 | Cytokines |
| CMTM5 | CKLF like MARVEL transmembrane domain containing 5 | -3.665293 | 6.63E-18 | Cytokines |
| MSTN | Growth/differentiation factor 8 | -3.6719766 | 8.69E-46 | Cytokines |
| GNRH1 | Progonadoliberin-1 | -3.81615 | 2.91E-79 | Cytokines |
| CGA | Glycoprotein hormones, alpha polypeptide | -3.9631538 | 2.92E-24 | Cytokines |
| PDGFD | Platelet-derived growth factor D | -3.9855085 | 1.32E-96 | Cytokines |
| NGF | Beta-nerve growth factor | -4.7156116 | 1.63E-86 | Cytokines |
| TDGF1 | Teratocarcinoma-derived growth factor 1 | -4.7806913 | 3.51E-60 | Cytokines |
| THPO | Thrombopoietin | -4.8857681 | 9.05E-71 | Cytokines |
| FGF7 | Fibroblast growth factor 7 | -5.0035644 | 1.56E-66 | Cytokines |
| GH1 | Somatotropin | -5.0928904 | 7.38E-30 | Cytokines |
| VIP | VIP peptides | -5.2082226 | 8.77E-59 | Cytokines |
| PRL | Prolactin | -5.2479071 | 1.88E-34 | Cytokines |
| TRH | Pro-thyrotropin-releasing hormone | -5.3302302 | 5.17E-56 | Cytokines |
| GREM2 | Gremlin-2 | -5.385698 | 2.23E-65 | Cytokines |
| NPPA | Natriuretic peptides A | -5.3950244 | 1.43E-50 | Cytokines |
| OGN | Mimecan | -5.657355 | 1.06E-77 | Cytokines |
| INHA | Inhibin alpha chain | -5.6960109 | 4.61E-114 | Cytokines |
| CHGB | Secretogranin-1 | -5.7289395 | 1.30E-69 | Cytokines |
| FIGF | Vascular endothelial growth factor D | -5.8954307 | 1.33E-84 | Cytokines |
| INS | Insulin | -6.0836732 | 1.19E-90 | Cytokines |
| LEFTY2 | Left-right determination factor 2 | -6.2850828 | 1.66E-68 | Cytokines |
| GKN1 | Gastrokine-1 | -6.3088755 | 3.48E-96 | Cytokines |
| FGF22 | Fibroblast growth factor 22 | -6.4564033 | 3.04E-90 | Cytokines |
| ANGPTL5 | Angiopoietin-related protein 5 | -10.075502 | 6.03E-131 | Cytokines |
| PROK1 | Prokineticin-1 | -10.185791 | 4.54E-122 | Cytokines |
| GZMB | Granzyme B | 4.15638462 | 5.86E-42 | NaturalKiller_Cell_Cytotoxicity |
| NCR2 | Natural cytotoxicity triggering receptor 2 | 3.50380013 | 6.47E-22 | NaturalKiller_Cell_Cytotoxicity |
| CD48 | CD48 antigen | 3.24649282 | 6.91E-50 | NaturalKiller_Cell_Cytotoxicity |
| NCR3 | Natural cytotoxicity triggering receptor 3 | 3.20555512 | 1.47E-27 | NaturalKiller_Cell_Cytotoxicity |
| TYROBP | TYRO protein tyrosine kinase-binding protein | 3.19553092 | 7.08E-61 | NaturalKiller_Cell_Cytotoxicity |
| SH2D1B | SH2 domain-containing protein 1B | 3.16892024 | 1.90E-33 | NaturalKiller_Cell_Cytotoxicity |
| PRKCG | Protein kinase C gamma type | 3.11149354 | 1.05E-19 | NaturalKiller_Cell_Cytotoxicity |
| FCGR3A | Low affinity immunoglobulin gamma Fc region receptor III-A | 2.7106135 | 2.07E-33 | NaturalKiller_Cell_Cytotoxicity |
| BID | BH3-interacting domain death agonist | 2.6207495 | 1.19E-76 | NaturalKiller_Cell_Cytotoxicity |
| NCR1 | Natural cytotoxicity triggering receptor 1 | 2.58829589 | 6.38E-15 | NaturalKiller_Cell_Cytotoxicity |
| HCST | Hematopoietic cell signal transducer | 2.08339769 | 7.07E-41 | NaturalKiller_Cell_Cytotoxicity |
| ITGB2 | Integrin beta-2 | 2.02568283 | 6.09E-24 | NaturalKiller_Cell_Cytotoxicity |
| FAS | Tumor necrosis factor receptor superfamily member 6 | -2.4344634 | 8.31E-57 | NaturalKiller_Cell_Cytotoxicity |
| CD244 | Natural killer cell receptor 2B4 | -2.7266623 | 1.73E-33 | NaturalKiller_Cell_Cytotoxicity |
| PLCG1 | 1-phosphatidylinositol 4,5-bisphosphate phosphodiesterase gamma-1 | -2.759166 | 2.38E-59 | NaturalKiller_Cell_Cytotoxicity |
| SHC2 | SHC-transforming protein 2 | -3.6841005 | 4.76E-68 | NaturalKiller_Cell_Cytotoxicity |
| CBLC | E3 ubiquitin-protein ligase CBL-C | 9.15815714 | 3.32E-161 | TCRsignalingPathway |
| PAK6 | P21 (RAC1) Activated Kinase 6 | 3.86990307 | 1.80E-71 | TCRsignalingPathway |
| ICOS | Inducible T-cell costimulator | 3.4334078 | 3.31E-28 | TCRsignalingPathway |
| CD3G | T-cell surface glycoprotein CD3 gamma chain | 3.31345099 | 2.73E-34 | TCRsignalingPathway |
| CD3D | T-cell surface glycoprotein CD3 delta chain | 2.35289896 | 2.34E-16 | TCRsignalingPathway |
| PRKCQ | Protein kinase C theta type | 2.18755366 | 2.86E-37 | TCRsignalingPathway |
| CD3E | T-cell surface glycoprotein CD3 epsilon chain | 2.12524866 | 1.19E-19 | TCRsignalingPathway |
| PAK7 | Serine/threonine-protein kinase PAK 5 | 2.05640509 | 1.99E-09 | TCRsignalingPathway |
| RASGRP1 | RAS guanyl-releasing protein 1 | 2.05581322 | 3.12E-35 | TCRsignalingPathway |
| PAK3 | Serine/threonine-protein kinase PAK 3 | -3.7086029 | 1.17E-40 | TCRsignalingPathway |
|  |  |  |  |  |
