# Supplementary material for: A novel immune-related prognostic signature in epithelial ovarian carcinoma
Source: Aging (Albany NY). 2021 Apr 4;13(7):10289–311. doi: 10.18632/aging.202792 (PMC8064207; doi:10.18632/aging.202792)
Supplement: Supplementary Table 4 [file aging-13-202792-s005.doc]

Supplementary Table 4. The table of differentially expressed transcription factors.

| Symbol | Description | logFC | P.Value |
| --- | --- | --- | --- |
| MYBL2 | Myb-related protein B | 8.18070865 | 2.881E-164 |
| EHF | ETS homologous factor | 8.14857616 | 6.115E-164 |
| FOXA2 | Hepatocyte nuclear factor 3-beta | 7.99160889 | 7.7641E-82 |
| GRHL2 | Grainyhead-like protein 2 homolog | 7.47975056 | 9.96E-169 |
| CENPA | Histone H3-like centromeric protein A | 7.30928242 | 3.128E-147 |
| SOX9 | Transcription factor SOX-9 | 7.03677387 | 3.219E-154 |
| SPDEF | SAM pointed domain-containing Ets transcription factor | 6.01778698 | 2.0153E-72 |
| MYB | Transcriptional activator Myb | 5.67763024 | 1.186E-109 |
| TFAP2C | Transcription factor AP-2 gamma | 5.40386978 | 1.957E-99 |
| BCL11A | B-cell lymphoma/leukemia 11A | 5.39161423 | 5.661E-100 |
| SOX17 | Transcription factor SOX-17 | 4.91512269 | 2.578E-128 |
| NCAPG | Condensin complex subunit 3 | 4.90933632 | 2.415E-130 |
| HOXB7 | Homeobox protein Hox-B7 | 4.86927095 | 6.5145E-92 |
| FOXM1 | Forkhead box protein M1 | 4.81651888 | 2.929E-131 |
| TFAP2A | Transcription factor AP-2-alpha | 4.66946733 | 1.031E-61 |
| NFE2 | Transcription factor NF-E2 45 kDa subunit | 4.53426994 | 7.8893E-66 |
| HNF4G | Hepatocyte nuclear factor 4-gamma | 4.45415522 | 7.5583E-30 |
| SPIB | Transcription factor Spi-B | 4.1850875 | 8.2312E-49 |
| E2F7 | Transcription factor E2F7 | 4.08997002 | 6.9821E-84 |
| KLF5 | Krueppel-like factor 5 | 3.76515164 | 5.871E-100 |
| HNF1B | Hepatocyte nuclear factor 1-beta | 3.46374529 | 1.2741E-15 |
| VDR | Vitamin D3 receptor | 3.37110259 | 1.7742E-69 |
| POU5F1 | POU domain, class 5, transcription factor 1 | 3.09918441 | 7.4778E-42 |
| CEBPA | CCAAT/enhancer-binding protein alpha | 2.98414136 | 9.5828E-59 |
| GATA3 | Trans-acting T-cell-specific transcription factor GATA-3 | 2.9233568 | 1.3366E-36 |
| LMNB1 | Lamin-B1 | 2.75365173 | 4.7947E-66 |
| ELF5 | ETS-related transcription factor Elf-5 | 2.70363837 | 3.3455E-14 |
| TEAD4 | Transcriptional enhancer factor TEF-3 | 2.68831753 | 9.6769E-71 |
| PAX5 | Paired box protein Pax-5 | 2.66871793 | 2.9129E-18 |
| FOXP3 | Forkhead box protein P3 | 2.55643863 | 2.3299E-50 |
| LHX2 | LIM/homeobox protein Lhx2 | 2.53613276 | 4.5216E-16 |
| E2F3 | Transcription factor E2F3 | 2.50885277 | 6.0568E-68 |
| SALL4 | Sal-like protein 4 | 2.508747 | 1.7282E-24 |
| CHD7 | Chromodomain-helicase-DNA-binding protein 7 | 2.50044645 | 9.3339E-55 |
| IRF5 | Interferon regulatory factor 5 | 2.43893146 | 9.2523E-53 |
| TP73 | Tumor protein p73 | 2.37462971 | 5.8224E-30 |
| C17orf96 | Elongin BC and Polycomb repressive complex 2-associated protein | 2.26141848 | 8.6947E-39 |
| LEF1 | Lymphoid enhancer-binding factor 1 | 2.2337132 | 1.2496E-29 |
| E2F1 | Transcription factor E2F1 | 2.22250112 | 5.7468E-52 |
| TCF7 | Transcription factor 7 | 2.14822701 | 7.7201E-40 |
| H2AFX | Histone H2AX | 2.0527152 | 3.8223E-49 |
| TCF7L1 | Transcription factor 7-like 1 | -2.0516782 | 2.731E-26 |
| MAFF | Transcription factor MafF | -2.0924935 | 2.0779E-40 |
| HOXC9 | Homeobox protein Hox-C9 | -2.1495546 | 3.8044E-15 |
| KAT2B | Histone acetyltransferase KAT2B | -2.1780739 | 1.4511E-55 |
| SNAI2 | Zinc finger protein SNAI2 | -2.2397877 | 5.1793E-28 |
| BACH2 | Transcription regulator protein BACH2 | -2.4654603 | 4.723E-34 |
| MEF2C | Myocyte-specific enhancer factor 2C | -2.4830963 | 1.7032E-64 |
| OGT | UDP-N-acetylglucosamine--peptide N-acetylglucosaminyltransferase 110 kDa subunit | -2.5403693 | 8.1849E-63 |
| CHD1 | Chromodomain-helicase-DNA-binding protein 1 | -2.57656 | 1.3299E-75 |
| ARNTL | Aryl hydrocarbon receptor nuclear translocator-like protein 1 | -2.5792693 | 2.1128E-80 |
| HNF4A | Hepatocyte nuclear factor 4-alpha | -2.6186227 | 3.3949E-15 |
| EPO | Erythropoietin | -2.7349345 | 3.4383E-21 |
| MITF | Microphthalmia-associated transcription factor | -2.8680151 | 2.0881E-67 |
| GATA6 | Transcription factor GATA-6 | -2.8965347 | 3.9346E-39 |
| FOXO1 | Forkhead box protein O1 | -3.000742 | 6.4135E-83 |
| MAF | Transcription factor Maf | -3.0818641 | 5.7854E-67 |
| EBF1 | Transcription factor COE1 | -3.0866715 | 3.6018E-68 |
| RUNX1T1 | Protein CBFA2T1 | -3.1078366 | 1.84E-45 |
| ELL2 | RNA polymerase II elongation factor ELL2 | -3.1612919 | 1.4731E-66 |
| EZH1 | Histone-lysine N-methyltransferase EZH1 | -3.2746384 | 1.61E-107 |
| TAL1 | T-cell acute lymphocytic leukemia protein 1 | -3.2802166 | 1.8915E-65 |
| NR2F2 | COUP transcription factor 2 | -3.3109531 | 4.1235E-84 |
| STAT5B | Signal transducer and activator of transcription 5B | -3.3170922 | 8.796E-112 |
| KLF4 | Krueppel-like factor 4 | -3.5188262 | 1.1657E-74 |
| ASCL1 | Achaete-scute homolog 1 | -3.5360956 | 5.6886E-22 |
| PPARG | Peroxisome proliferator-activated receptor gamma | -3.5616458 | 4.64E-80 |
| PBX3 | Pre-B-cell leukemia transcription factor 3 | -3.5917208 | 4.6053E-89 |
| CBX7 | Chromobox protein homolog 7 | -3.634989 | 2.868E-106 |
| NR2F1 | COUP transcription factor 1 | -3.8268425 | 2.3443E-72 |
| GREB1 | Protein GREB1 | -5.2865199 | 1.0962E-96 |
| NR4A1 | Nuclear receptor subfamily 4 group A member 1 | -5.3825448 | 3.984E-102 |
| MYH11 | Myosin-11 | -5.6028311 | 6.1508E-94 |
| TCF21 | Transcription factor 21 | -5.7839722 | 5.8022E-73 |
| FOXP2 | Forkhead box protein P2 | -6.2785445 | 1.0557E-73 |
| GATA4 | Transcription factor GATA-4 | -7.9468744 | 2.2196E-62 |
|  |  |  |  |
